# *FMOPhore* for hotspot identification and efficient fragment-to-lead growth strategies

**DOI:** 10.1038/s41467-026-72384-x

**Published:** 2026-04-28

**Authors:** Peter E. G. F. Ibrahim, Simone Altmann, Ulrich Zachariae, David Horn, Ian H. Gilbert, Michael J. Bodkin

**Affiliations:** 1 Drug Discovery Unit, Wellcome Centre for Anti-Infectives Research, School of Life Sciences, University of Dundee, Dundee, UK; 2 School of Life Sciences, University of Dundee, Dundee, UK; 3 Division of Biological Chemistry and Drug Discovery, School of Life Sciences, University of Dundee, Dundee, UK

**Keywords:** Computational models, Structure-based drug design, Lead optimization, Computational biology and bioinformatics, Computational chemistry

## Abstract

Fragment based drug design is like a chess game in that a good or a bad move can dramatically influence the outcome. In the design process, it is important to identify the key binding site residues (hotspots) that can have a substantial impact on ligand potency and efficiency. Here, we introduce FMOPhore algorithm represented with a scoring function named FP-score, which combines Quantum Mechanics Fragment Molecular Orbital calculations with 3D-protein-ligand pharmacophore models. FP-score accurately classifies binding site residues in two classes: 1) Hotspot residues (Delineated into three categories; Anchor, Transient, and Accessible) and 2) non-hotspot residues. We apply our algorithm in two different scenarios: holo-complex and apo-structure, testing its robustness on 46 different protein targets including an experimental case study on drug-resistance hotspots across 829 protein-ligand complexes. We handle protein binding site flexibility using Dy-FMOPhore which improves the detection of hotspots. FMOPhore provides valuable insight for efficient, selective fragment growing and lead optimization strategies.

## Abstract (web summary)

The study introduces FMOPhore, a computational tool that identifies key binding site residues to improve drug design by guiding efficient fragment growth and lead optimization across diverse protein targets.

## Introduction

Protein binding sites are typically composed of small regions (sub-pockets) that can contribute significantly to ligand’s binding free energy and affinity^1^. Identifying the key interacting residues “hotspots” on these sub-pockets is important for efficient drug design^2^. Hotspots are known to be the areas that are more likely to bind small drug-like compounds with higher affinity compared to other parts of the binding site, and targeting such ‘hotspots’ is a foundation of fragment-based drug discovery (FBDD).

Fragment growing is one of the most applied FBDD approaches^3^. It is the addition of functional groups and substituents on an identified hit fragment to increase its interaction and affinity for the target protein. Growth of fragment hits into lead compounds, with appropriate physicochemical and pharmacokinetic (PK) properties, is a key challenge in FBDD. Hence, efficient fragment growing strategies must be employed to transform a fragment hit typically obeying the rule-of-three (Ro3) into a lead-like compound with optimal properties^4–6^.

The addition of functional moieties onto a hit fragment requires the determination of proper directionality which are defined as “growth vectors”. These are synthetically amenable positions on the core-scaffold where functional groups or building blocks can be attached. This can be facilitated by pre-determination of binding site hotspots (size and geometry), allowing to design the substituent features and its complementary functional group^7^. The challenge is to obtain growth vectors with optimal interaction energy, and appropriate 3D-spatial geometry.

Experimentally hotspots are defined as regions on the protein surface where crystallographic fragments aggregate^8^. This was reinforced when consensus sites of solvent molecules were identified using mixed solvent crystallography (MSCS)^9,10^. Alongside crystallography and NMR, other biophysical approaches, such as Surface Plasmon Resonance (SPR) or Thermal Shift Analysis, are commonly used for fragment screening^11–13^. Although druggability indices have been created from NMR data, it is challenging to deduce the strength of specific key interactions from experimental approaches^10^. Molecular simulation approaches such as SILCS^14^ or MixMD^15^ can be used but are computationally very demanding.

Several computational algorithms have been developed for hotspot identification that map the binding site using a variety of molecular probes at the protein surface. Commonly used programs such as MCSS^16^ or GRID^17^ have now been superseded by FTMap (E-FTMap)^18,19^ PLImap^20^ and Hotspots API^21^. These methods broadly differ in their approach to fragment placement, sampling, clustering, and scoring. For example, Hotspots API uses SuperStar propensities for MIF (molecular interaction field) generation rather than a GRID-like forcefield. FTMap uses fast Fourier transform docking for probe sampling. PLImap uses the PLIff protein ligand interaction forcefield for scoring^22^.

However, identifying hotspots and quantitatively determining the strength of interaction between a protein binding site and the functional group (pharmacophore) of a complementary ligand has been challenging and remains a noticeable limitation in many approaches^23,24^.

In this work we introduce FMOPhore algorithm that combines quantum mechanics (QM) using fragment molecular orbital (FMO) calculations with protein-ligand pharmacophore models in a scoring function named FP-score. Our approach assesses each binding site residue qualitatively and quantitatively in terms of energy and accessibility. FMOPhore highlights the critical interactions in a binding site defined as favourable interactions (that drive binding), and unfavourable interactions (clashes). The algorithm offers valuable direction for lead optimization strategies i.e., by pointing out which features on a hit candidate must be kept and are vital for the structure activity relationship (SAR), and which can be modified to increase potency, affinity, and selectivity. We demonstrate the general utility of our approach across 46 different protein systems, with 840 protein-ligand complexes including direct fragment-based design studies. Firstly, we illustrate the utility of FP-score to provides insights on multiple fragment-to-lead growing strategies from literature. Secondly, we assess the quality of the predictions and introduce the application of dynamic hotspot prediction named Dy-FMOPhore for flexible protein systems. Thirdly, we took advantage of a recent proteasome β5 subunit (PSMB5) saturation mutagenesis study to evaluate the generalizability of the FMOPhore algorithm in identifying drug resistance hotspots. Finally, we demonstrate FMOPhore algorithm in different modes, static or dynamic to assess protein-ligand flexibility and its influence on hotspots classification.

## Results

### Main-protease protein

**Fig. 1:**
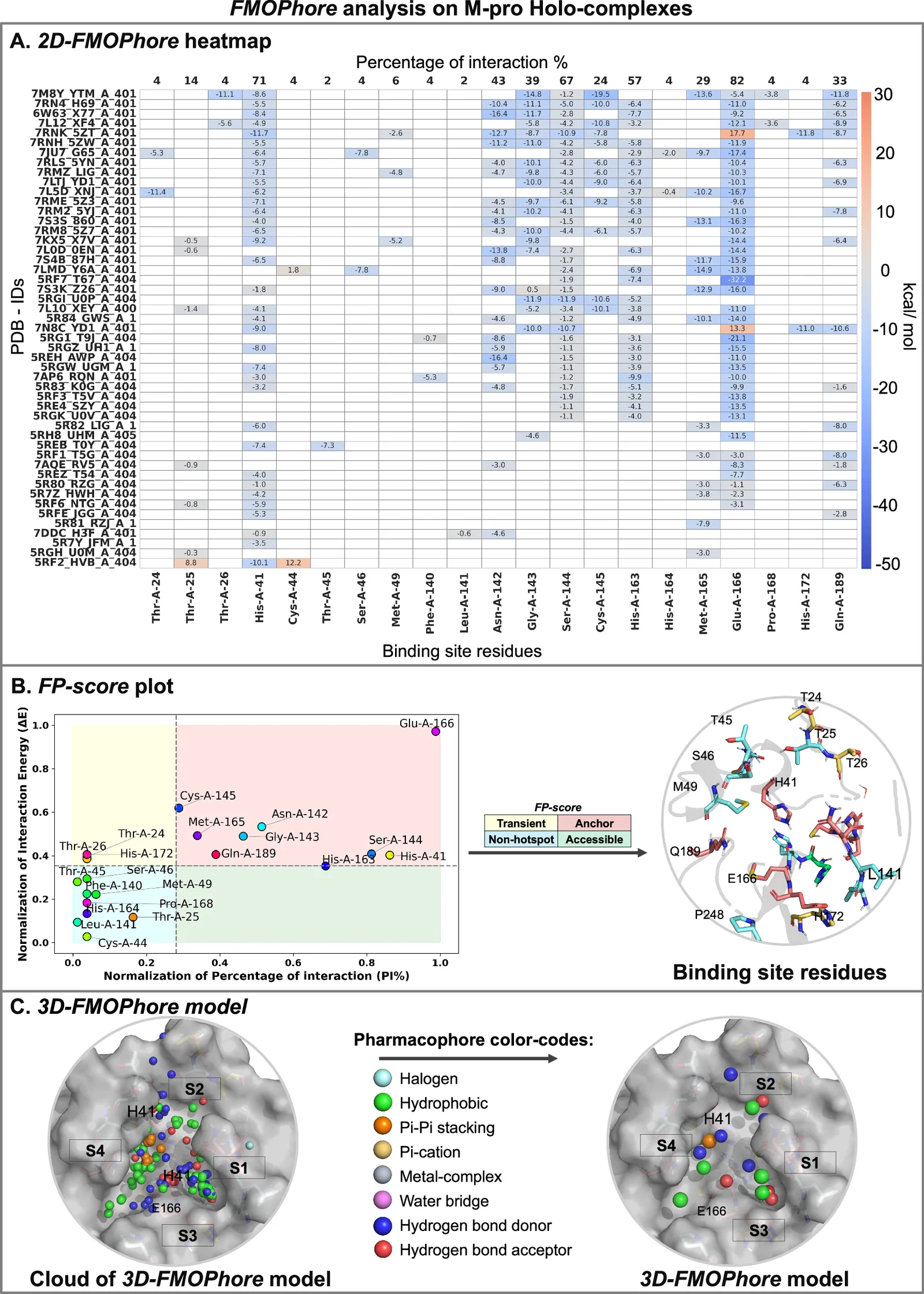
*FMOPhore* analysis on M-pro Holo-complexes. **A** 2D-FMOPhore heatmap. The binding site residues on the bottom *x*-axis, and the percentage of interaction (%) on the top *x*-axis. PDB-IDs representing the ligands of the complex (PDB-D_Ligand-name_Chain-ID_Ligand-Number) on the *y*-axis (left side), and the color code of each cell corresponds to the interaction energy values *y*-axis (right side), (kcal/mol) between the function group on the ligands and the binding site residue. **B** FP-score plot categorizing the binding site residues, normalization of Interaction energies on the *y*-axis, and normalization of percentage of interaction on the *x*-axis. **C** M-pro 3D-FMOPhore model based on Holo-complexes. Sub-pockets (S1, S2, S3, and S4). Binding site residues are coloured according to FP-score categories by FMOPhore analysis on the holo-complexes.

The Main-protease protein is an antiviral target for SAR-COV-2^25^. We analysed 50 different holo-complexes of noncovalent protein-ligands complexes against main-protease protein collected from crystallographic PDB-IDs. In case of multiple co-crystalized ligands against the same target, 2D-FMOPhore heatmaps and 3D-FMOPhore models are constructed representing a quantitative-structure activity relationship model (QSAR). The FP-score quadrant matrix plot is used to classify binding site residues. The 2D-FMOPhore heatmap (Fig. 1A.) shows that more than 80% of ligands are binding to Glu-A-166 consistently with favourable interaction energy values which is represented as a key hotspot class; Anchor-category on the FP-score plot (Fig. 1B.).

The robustness of FMOPhore is in showing that certain residues exhibit trends across different ligands, highlighting these hotspots (residue-ligand interactions) can provide medicinal chemists with critical insights for developing active inhibitors against the protein targets. This can be observed on the FP-score plot representing those prioritized Anchor-category residues (hotspots), (Fig. 1B), (e.g., His-A-41, Asn-A-142, Gly-A-143, Ser-A-144, Cys-A-145, Met-A-165, Glu-A-166, and Gln-A-189). The 2D-FMOPhore heatmap can then be translated into a cloud of 3D-FMOPhore model (Pharmacophore model combined with interaction energy (ΔE^FMO^)), that is minimized into a single 3D-FMOPhore model based on the FP-score prioritized hotspots, (Fig. 1C).

The 3D-FMOPhore model represents the optimal 3D-spatial geometry for pharmacophore features towards the key hotspot residues, based on the lowest interaction energy and accessibility to form a specific type of interaction (bond type). The cloud of 3D-FMOPhore features represent the critical accessibility of the 3D-geometric space around a certain function group of binding site residue. For example, the cloud of hydrogen bond acceptor around the Glu-A-166 (Sub-pockets; S3), and the cloud of π–π-stacking and hydrophobic bonds around the His-A-41(Sub-pocket; S2 and S4), (Fig. 1C). The 3D-FMOPhore model can be used to drive the rational of designing active compounds.

For this we compare between two co-crystalized ligands (PDB-IDs: 7N8C and 7S3S). On the 2D-FMOPhore heatmap (Fig. 1A), the co-crystalized ligand (PDB-ID: 7N8C), is found to have an unfavourable interaction with a Glu-A-166, (Anchor-category residue). The interaction is driven by the carboxylic group (side chain) of the Glu-A-166, with the NH-group on the uracil ring of the ligand, (Supplementary Fig. 1). However, having two electronegative oxygen moieties (on the uracil ring) leads to electrostatic repulsion and instability of the interaction. In comparison to the co-crystalized ligand (PDB-ID: 7S3S), which developed a strong electrostatic interaction (hydrogen bond) with the amide group of the backbone of the Glu-A-166 aligning on 3D-spatial geometry of the 3D-FMOPhore model. The iso-quinoline ring maintains a hydrogen bond acceptor and hydrophobic interaction which aligns on the 3D-FMOhore model features. This is translated in a better IC_50_ value (1.6 μM) of the PDB-ID: 7S3S over PDB-ID: 7N8C (IC_50_ > 20 μM).

To validate the stability of the 3D-geometric positions of the 3D-FMOPhore features, we performed a short molecular dynamic simulation of the complex PDB-ID: 7S3S (50-75 nanoseconds, 3 replicates). Ligand PDB-ID: 7S3S, kept a constant electrostatic hydrogen bond with the Glu-A-166, (percentage of interaction along the molecular dynamic simulation; 100%), 60% and 80% electrostatic hydrogen bond with the Ser-A-144 and His-A-163 respectively, as shown in Supplementary Fig. 1 and Supplementary Movie 1.

### Ephrin A2 receptor

**Fig. 2:**
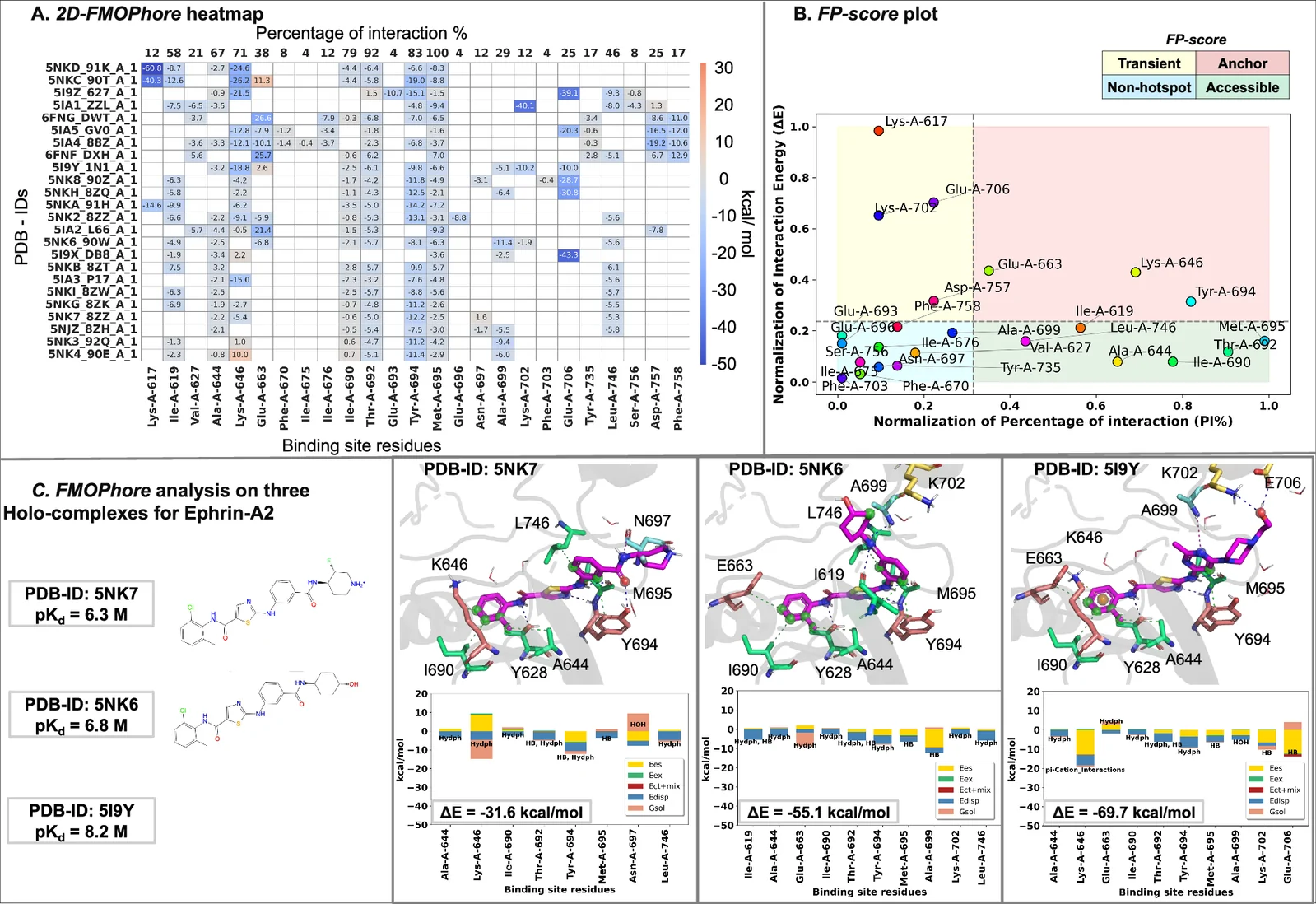
*FMOPhore* analysis on Ephrin-A2 Holo-complexes. **A** 2D-FMOPhore heatmap. The binding site residues on the bottom *x*-axis, and the percentage of interaction (%) on the top *x*-axis. PDB-IDs representing the ligands of the complex (PDB-D_Ligand-name_Chain-ID_Ligand-Number) on the *y*-axis (left side), and the color code of each cell corresponds to the interaction energy values *y*-axis (right side), (kcal/mol) between the function group on the ligands and the binding site residue. **B** FP-score plot categorizing the binding site residues, normalization of Interaction energies on the *y*-axis, and normalization of percentage of interaction on the *x*-axis. **C** FMOPhore analysis for three Ephrin-A2 Holo-complex structures, PDB-IDs: 5NK7, 5NK6, and 5I9Y. Bar chart showing interaction bond type and energy. Binding site residues are colored according to FP-score categories by FMOPhore analysis on the holo-complexes, and the ligands colored magenta.

The Tyrosine kinase EPHA2 (Ephrin type-A receptor 2) receptor plays an important role in cancer development and treatment^26^. We performed FMOPhore analysis on twenty-four different ligands binding to the EPHA2 receptor to rationalize the key hotspots of its binding site. All co-crystalized ligands analysed are found to bind with moderate interaction energy to Met-A-695 (2D-FMOPhore heatmap, Fig. 2A.) which is categorized as Accessible-category residue on the FP-score plot, (Fig. 2B).

Three co-crystalized ligands were used as examples to rationalize the hotspot classes, PDB-IDs: 5NK7, 5NK6, and 5I9Y, (Fig. 2C). The co-crystalized ligand (PDB-ID: 5NK7; pK_d_ = 6.3) is found to bind to two key Anchor-category residues, Lys-A-646 and Tyr-A-694, and multiple Accessible-category residues. The co-crystalized ligand (PDB-ID: 5NK6; pK_d_ = 6.8), is found to gain a weak hydrophobic interaction with Transient-category interaction (Lys-A-702). However, with better orientation of the hydroxyl moiety between two Transient-category residues Lys-A-702 and Glu-A-706, led to significant enhancement in the pK_d_ value of 8.2 of co-crystalized ligand (PDB-ID: 5I9Y), along with gaining interaction with three Anchor-category residues. This shows growing the reference hit molecule with growth vector towards developing interactions with Anchor and Transient-category residues leads to better dissociation constant.

### Protein Kinase B

**Fig. 3:**
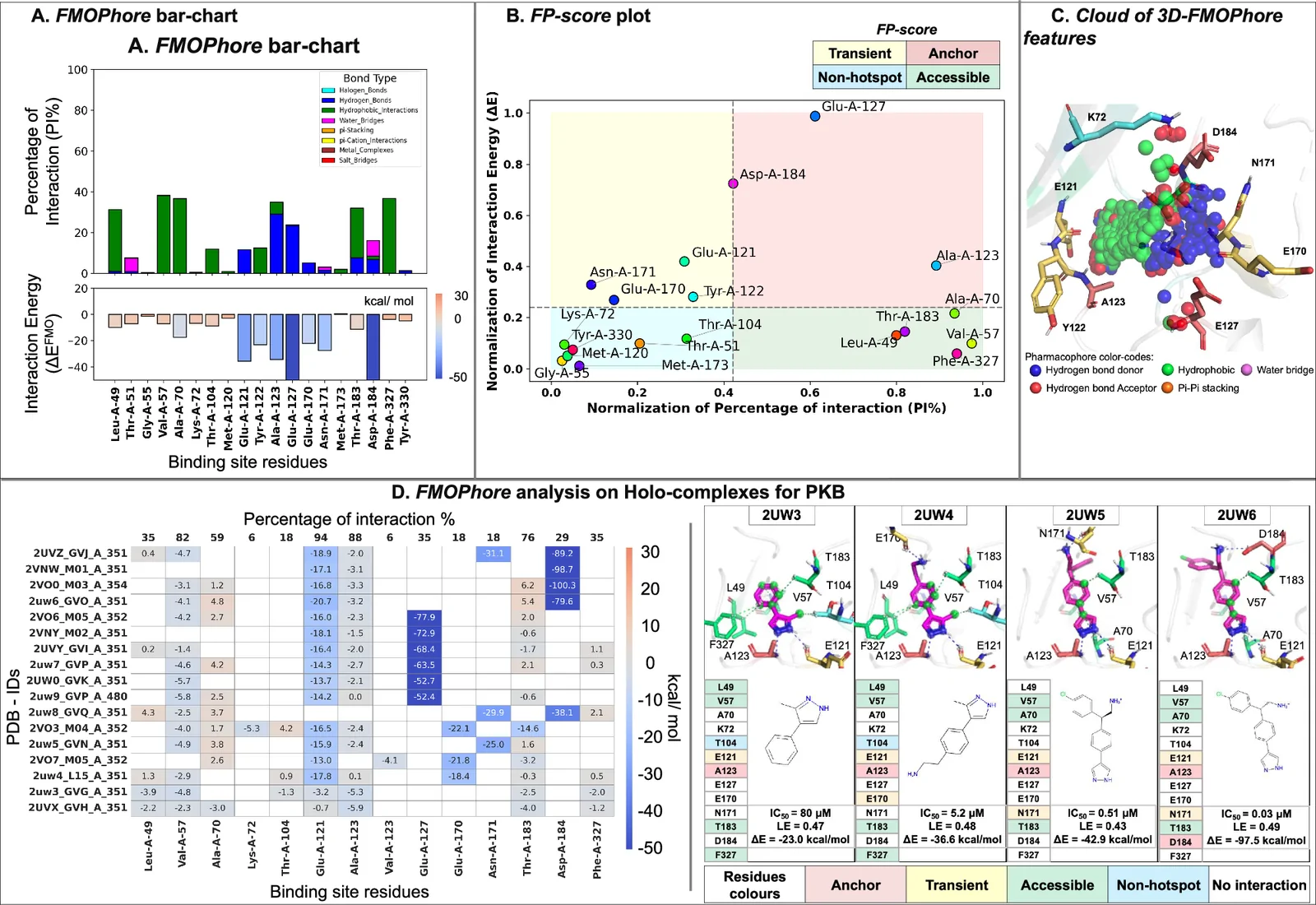
Static-*FMOPhore* scan on apo-structure for PKB receptor*.* **A** FMOPhore bar-chart showing the percentage of interaction between residues and probes, with interaction energy and type of bonds to be formed. **B** FP-score plot categorizing the binding site residues, normalization of Interaction energies on the *y*-axis, and normalization of percentage of interaction on the *x*-axis. **C** Cloud of 3D-FMOPhore features, with FP-score prioritize hotspot residues. **D** 2D-FMOPhore heatmap with 3D-visualization of binding interactions. Binding site residues are colored according to FP-score categories by FMOPhore scanning on the apo-structure. The bottom *x*-axis shows binding site residues, and the percentage of interaction observed on the top *x*-axis. PDB-IDs represent the ligands of the complex on the *y*-axis, and the color code of each cell corresponds to the interaction energy values (kcal/mol) between ligands and the binding site residue.

Protein Kinase B plays a pivotal role in regulating various signal transduction pathways which are crucial for cell growth, differentiation, and division. Inhibition of PKB, is considered a promising strategy for cancer treatment for its potential mechanism in tumour cell survival and progression^27,28^. The FP-score plot was generated by scanning the static-FMOPhore mode on an apo-structure of the Protein-Kinase B, (PDB-ID: 2UW3, without ligand bound) (Fig. 3A,B). An electronegative binding pocket formed between the Glu-A-127, Glu-A170, Asn-A-171 and Asp-A184 which are observed as Anchor-category and Transient-category residues with a cloud of 3D-FMOPhore features (Blue spheres: Hydrogen bond donor), (Fig. 3C).

Fragment elaborations by Saxty et al.^29^, started from two fragment scaffolds, PDB-IDs: 2UW3 and 2UVX, (Fig. 3D), and showed that upon targeting the electronegative binding pocket (between the Glu-A-127, Glu-A170, and Asn-A-171) gave an increase of 30-fold in affinity for the lead compound. The analysis of the holo-complexes using FMOPhore suggest that the fragment (PDB-ID: 2UW3) grew to compounds with PDB-IDs: 2UW4, 2UW5, 2UW6, 2UW7, and 2UW9, by keeping the starting scaffold of the primary hit fragment (2UW3), while reaching to the electronegative binding pocket which was reflected with a better interaction energy.

The fragment 2UW3 (IC_50_ = 80 μM, LE = 0.47, ΔE^FMO^ = −22.99 kcal/mol), maintaining key critical interactions with Ala-A-123, and Glu-A-121 (Anchor-category and Transient-category residues), (Fig. 3D). The first elaboration was the ligand in 2UW4, which gained a key interaction with a Transient-category residue Glu-A-170, (IC_50_ = 5.2 μM, LE = 0.48, ΔE^FMO^ = −36.6 kcal/mol). Binding to Transient-category residue, had a significant influence on the inhibitor affinity and gaining LE. That is the same for the lead compounds 2UW6, (IC_50_ = 0.03 μM, LE = 0.49, ΔE^FMO^ = −97.5 kcal/mol) which gained interaction with an Anchor-category residue Asp-A-184. This shows an efficient fragment growing strategy, by gaining interaction with Anchor-category residues, achieves both better IC_50_ and LE values. The electronegative pocket was predicted in the FMOPhore apo-structure scanning (using the probes library) as the key Hotspot-category residues (Fig. 3C), suggesting that this is a critical binding site sub-pocket to gain affinity, potency and for inhibitors targeting PKB.

### *FP-Score* for efficient fragment growing strategies

#### Biotin carboxylase target

Biotin carboxylase is a validated target for inhibition of bacterial fatty acid biosynthesis^30^. By scanning the apo structure of the active binding site of Biotin carboxylase target (using PDB ID: 2W71, without bound ligand), (Supplementary Fig. 2). The FP-score classifies Lys-A-159, Glu-A-201, Leu-A-204, and Glu-A-288, as Anchor-category binding site residues. This means that they are prioritized residues to gain potency and efficacy in growing strategy. Ile-A-157, Tyr-A-203, Pro-A-207, Leu-A-278 and Ile-437, are accessible with moderately favourable interaction energy, falling in the Accessible-category residues. This suggests they provide an accessible vector for growing strategies yet won’t have a significant impact on the LE as Anchor-category residues. Transient-category residues are the Lys-A-202, Lys-A-238, and His-A-438, with lower accessibility yet favourable interaction energy. The rest of the binding site residues were less prioritized for development of a high affinity lead compound with maximum ligand efficacy.

**Fig. 4:**
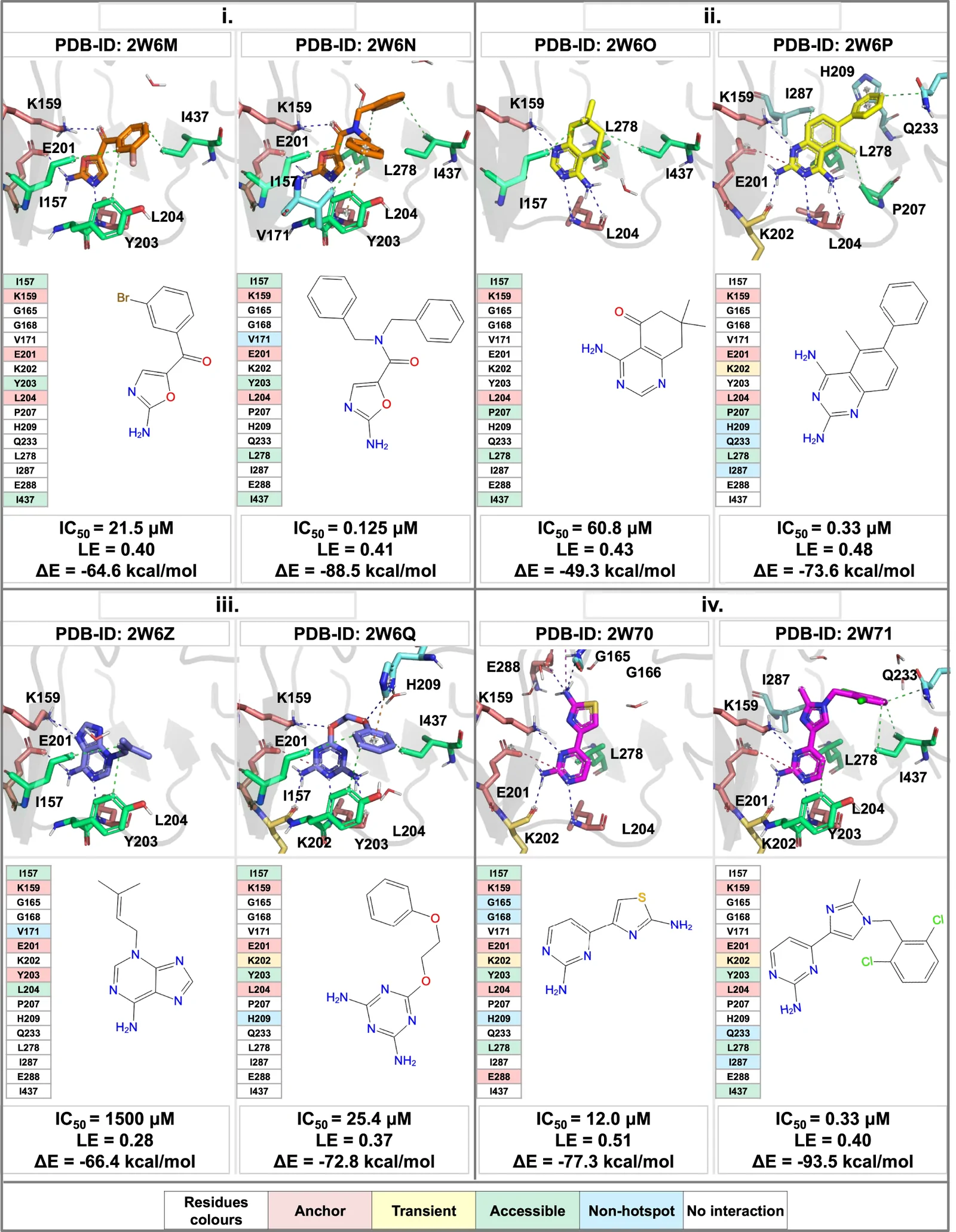
FMOPhore biotin carboxylase. 3D-visualization of protein–ligand complexes. Ligands (orange, yellow, violet, and magenta). Binding site residues are colored according to FP-score categories by FMOPhore scanning on the apo-structure.

Mochalkin et al.^28^, performed a fragment growing approach to deliver nanomolar range Biotin carboxylase inhibitors and reported eight different holo-complex crystal structures. FMOPhore was used to assess the binding of the crystalized ligands, represented in FP-score plot and 2D-FMOPhore heatmap (Supplementary Fig. 2) shows that 100% of the Biotin carboxylase co-crystalized ligands are binding to the Lys-A-159, and Leu-204, which are defined as key Anchor-category residues in the FMOPhore apo-structure scan. The authors started from four millimolar to micromolar range fragments to grow into lead compounds, (Fig. 4).

**The first fragment growing strategy** (Fig. 4i) for the PDB-ID: 2W6M (IC_50_ = 21.5 μM, LE = 0.40, ΔE^FMO^ = −64.6 kcal/mol), was developed to grow into PDB-ID: 2W6N (IC_50_ = 0.125 μM, LE = 0.41, ΔE^FMO^ = −88.5 kcal/mol). The main difference was in gaining interaction with the Leu-A-278 (Accessible-category) residue that was identified as highly accessible with moderate interaction energy in the apo-structure FMOPhore scanning and Val-A-171 (non-hotspot category). Although this growing strategy led to a better IC_50_ value, however the experimental LE shows minor improvement for the lead compound, which suggests that this fragment growing strategy could have been improved if targeted to other hotspot categories other than the non-hotspot residues.

**The second fragment growing strategy** (Fig. 4ii) was the core scaffold fragment, PDB-ID: 2W6O (IC_50_ = 60.8 μM, LE = 0.43, ΔE^FMO^ = −73.6 kcal/mol), that was found to bind to the Lys-A-159, and Leu-A-204. This emerged into the lead compound, PDB-ID: 2W6P (IC_50_ = 0.33 μM, LE = 0.48, ΔE^FMO^ = −88.5 kcal/mol), and maintained the same interaction with Lys-A-159, and Leu-A-204, yet gained another Anchor-category residue interaction with Glu-A-201 and a Transient-category residue interaction with Lys-A-202. This resulted in a better IC_50_ value and total experimental LE. However, the molecule (PDB-ID: 2W6P), also gained multiple non-hotspot-category interaction residues; His-A-209, Gln-A-233, and Ile-A-287. This implies that the ligand grew substantially in size with moderate influence on the efficiency of interaction. This is shown in the LE with 0.05 difference from the starting fragment.

**The third fragment growing strategy** (Fig. 4iii) started from fragment, PDB-ID: 2W6Z (IC_50_ = 1500 μM, LE = 0.28, ΔE^FMO^ = −66.4 kcal/mol), and was found to bind to three Anchor-category residues Lys-A-159, Glu-A-201, and Leu-A-204 and two Accessible-category residues, Ile-A-157 and Tyr-A-203. A core-scaffold modification morphed into compound (PDB-ID: 2W6Q, IC_50_ = 25.4 μM, LE = 0.37, ΔE^FMO^ = −72.8 kcal/mol), which maintained the same Anchor-category and Accessible-category interactions. The compound showed a noticeable increase in LE and total better IC_50_ value. This is due to a Transient-category interaction with the Lys-A-202, which was followed by growing the molecule into a more hydrophobic sub-pocket found between residues, His-A-209 (non-hotspot-category) and Ile-A-437 (Accessible-category).

**The fourth fragment growing strategy** (Fig. 4iv) in this study involves the core scaffold fragment PDB-ID: 2W70 (IC_50_ = 12.0 μM, LE = 0.51, ΔE^FMO^ = −77.3 kcal/mol), which was found to interact with all Anchor-category residues Lys-A-159, Leu-A-204 and Glu-A-288, through a strong electrostatic interaction forming a hydrogen bond, as well as with the Transient-category Lys-A-202. This should the best fragment to grow with LE 0.51, as a starting point across the other fragments. However, the fragment was grown into the compound (PDB-ID: 2W71, IC_50_ = 0.33 μM, LE = 0.40, ΔE^FMO^ = −93.5 kcal/mol) by maintaining the same interaction with Anchor-category residues except the Glu-A-288. This was a result of substituting the amine group with a methyl and hence lost a critical Anchor-category interaction. The compound gained other hydrophobic interactions with Tyr-A-203, Leu-A-278, and Ile-A-437, and weaker interactions with non-hotspot-category residues, Ile-A-287 and Gln-A-233. Although, the lead compound is found with better IC_50_ (0.33 μM) than the fragment (IC_50_ 12 μM). However, the starting fragment shows a much higher LE over the lead compound, 0.51 and 0.40 respectively. This suggests that keeping the amine group in the vicinity of the Glu-A-288, may have delivered a better ligand efficiency and potency, for the developed inhibitor.

#### Beta-secretase target

Another fragment screening study done by Murray et al., and Congreve et al., explores the aspartyl protease enzyme β-secretase (BACE-1) utilizing high throughput X-ray crystallography^31,32^. The screening process identified distinct chemotypes that bind to the catalytic aspartates: amino-heterocycles such as 2-aminoquinoline, aminopyridine, piperidine, and aliphatic hydroxyl groups. Although these fragment hits demonstrated weak inhibitory activity against BACE-1, with inhibition observed in the millimolar range, they were significant due to their relatively high ligand efficiencies (LE). Further progression from these initial fragments to the development of sub-micromolar inhibitors were carried out.

**Fig. 5:**
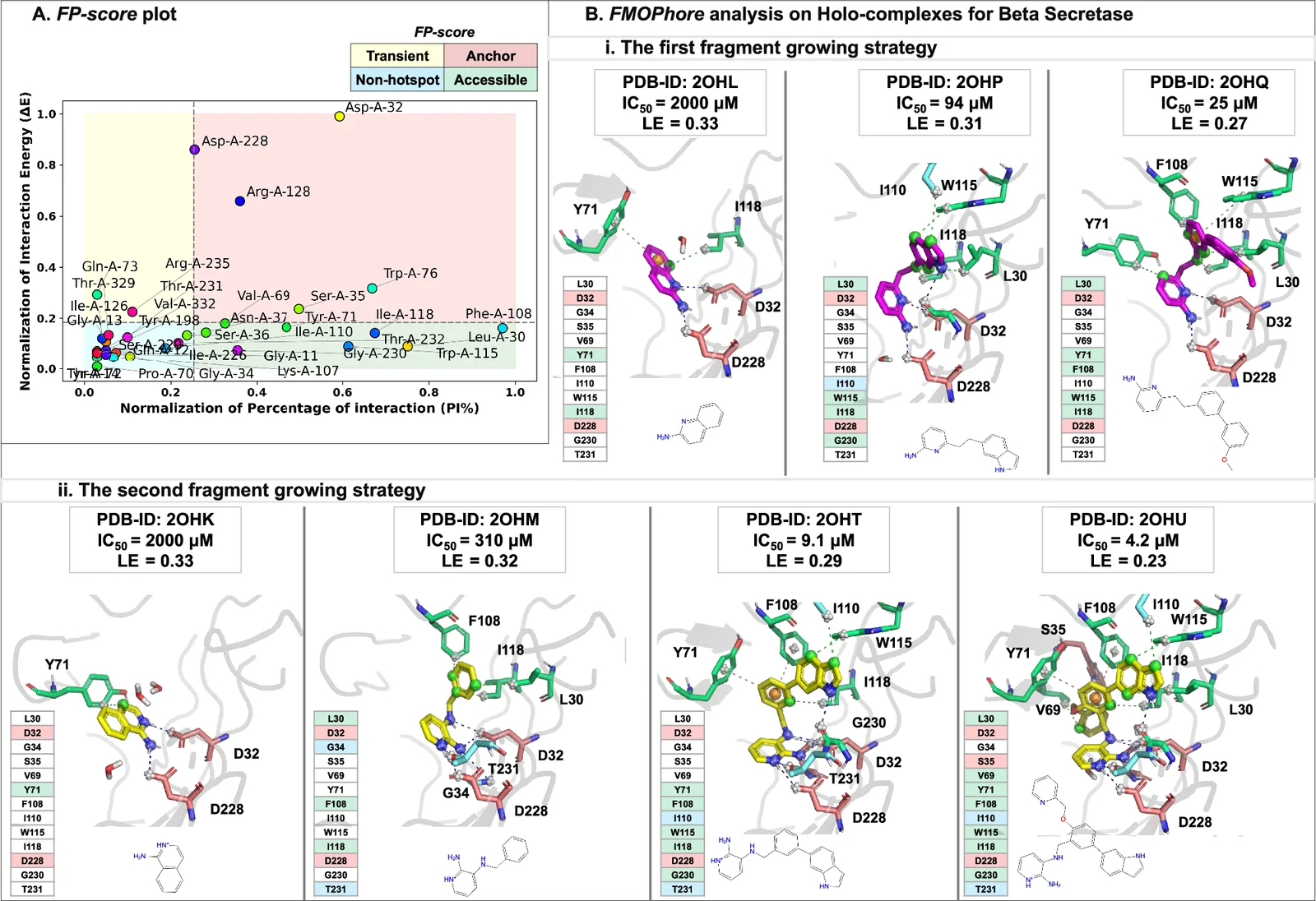
Static-FMOPhore Beta-secretase. **A** FP-score plot for the Apo-structure of Beta-secretase receptor. categorizing the binding site residues, normalization of Interaction energies on the *y*-axis, and normalization of percentage of interaction on the *x*-axis. **B** 3D-visualization of protein–ligand complexes. Ligands (magenta, yellow, and orange), binding site residues. Binding site residues are colored according to FP-score categories by FMOPhore analysis on the apo-structure scan. (i) The first fragment growing strategy. (ii) The second fragment growing strategy.

FMOPhore scan on the BACE-1 apo-structure suggests that Anchor-category residues are, Asp-A-32, Ser-A-35, Trp-A-76, Arg-A-128, Asp-A-228, interacting with these residues with a complementary functional group, is predicted to increase potency and affinity with ultimate LE, (Fig. 5A.). Other residues that fall in the Transient-category; Gln-A-73, and Arg-A-235, are also predicted to increase LE but they show less accessibility than the latter category. However, Leu-A-30, Asn-A-37, Val-A-69, Tyr-A-71, Phe-A-108, Trp-A-115, Ile-A-118, and Glu-A-230, these residues fall in the Accessible-category, which means binding to these residues is easily accessible, but they might show less improvement in LE if chosen as growth vectors, (Fig. 5A). Two fragments were used as a starting point for growing into lead compounds in this study.

**The first fragment growing strategy** (Fig. 5B. i) PDB-ID: 2OHL binds to Asp-A-32, Tyr-A-71, Ile-A-118, and Asp-A-228, (IC_50_ = 2000 μM, LE = 0.33, ΔE^FMO^ = −99.5 kcal/mol). This core scaffold was grown into the lead compounds PDB-IDs: 2OHP, and 2OHQ, with a better IC_50_ of 94 and 25 μM and interaction energies, −134.8 and −142.8 kcal/mol respectively. The lead compounds maintained similar interaction as the starting fragment but gained more interactions with Accessible-category residues. Although both; 2OHP, and 2OHQ, accomplished better overall IC_50_ values their LE was found to be lower than that of the starting fragment, 0.31 and 0.27, respectively.

**The second fragment growing strategy** (Fig. 5B. ii.) PDB-ID: 2OHK, is also found to have a common binding to Asp-A-32, and Asp-A-228, (IC_50_ = 2000 μM, LE = 0.33, ΔE^FMO^ = −124.8 kcal/mol). On progression to lead compounds PDB-IDs: 2OHM, 2OHT, and 2OHU, it gained interactions with accessible residues such as Phe-A-108, Ile-A-110, Gly-A-230, and Trp-A-115, along with some unfavourable interactions with non-hotspot residues Ile-A-118 and Thr-A-231, which lead to a much lower LE of 0.32, 0.29, and 0.23 respectively.

#### Multiple case studies

**Fig. 6:**
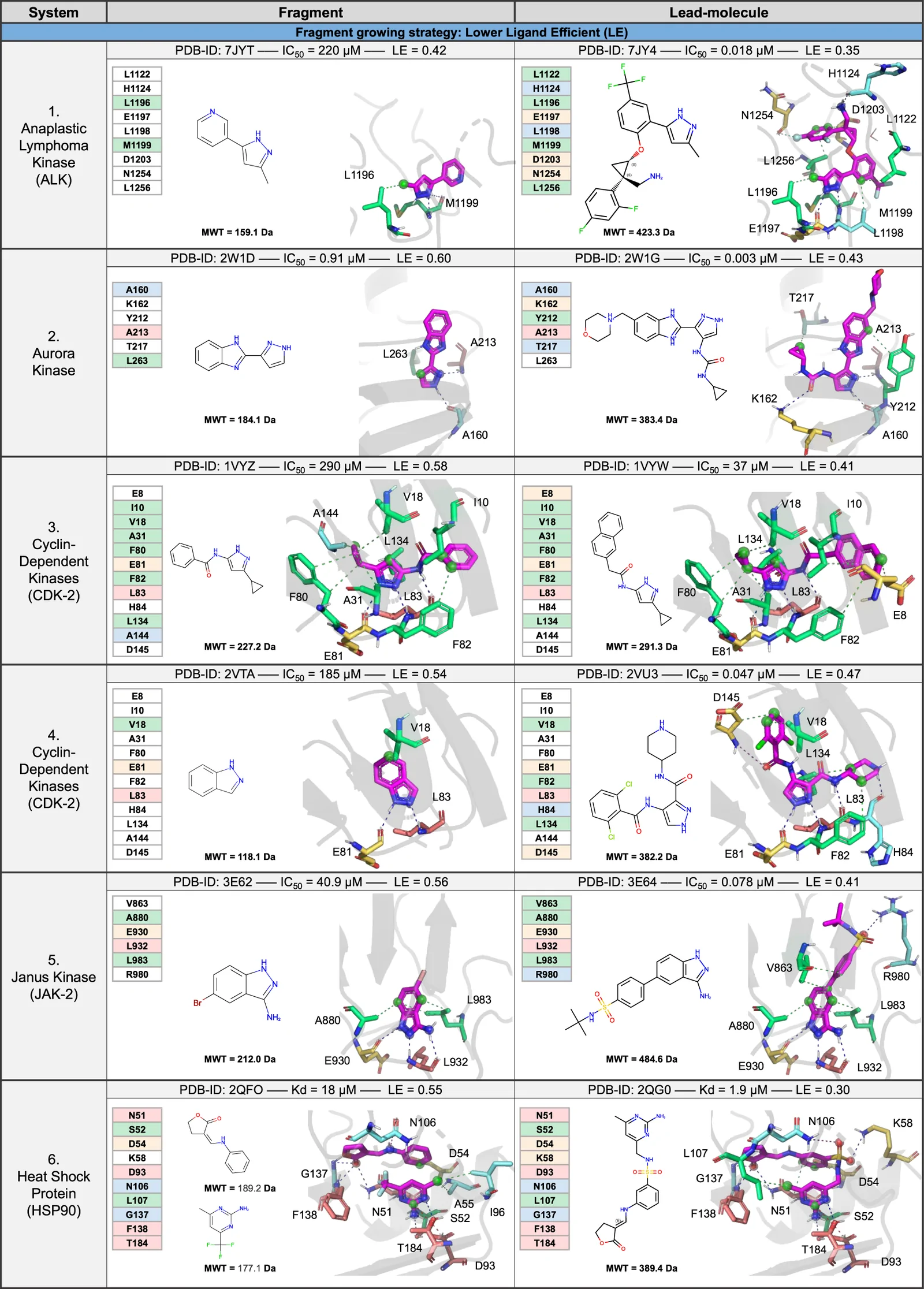

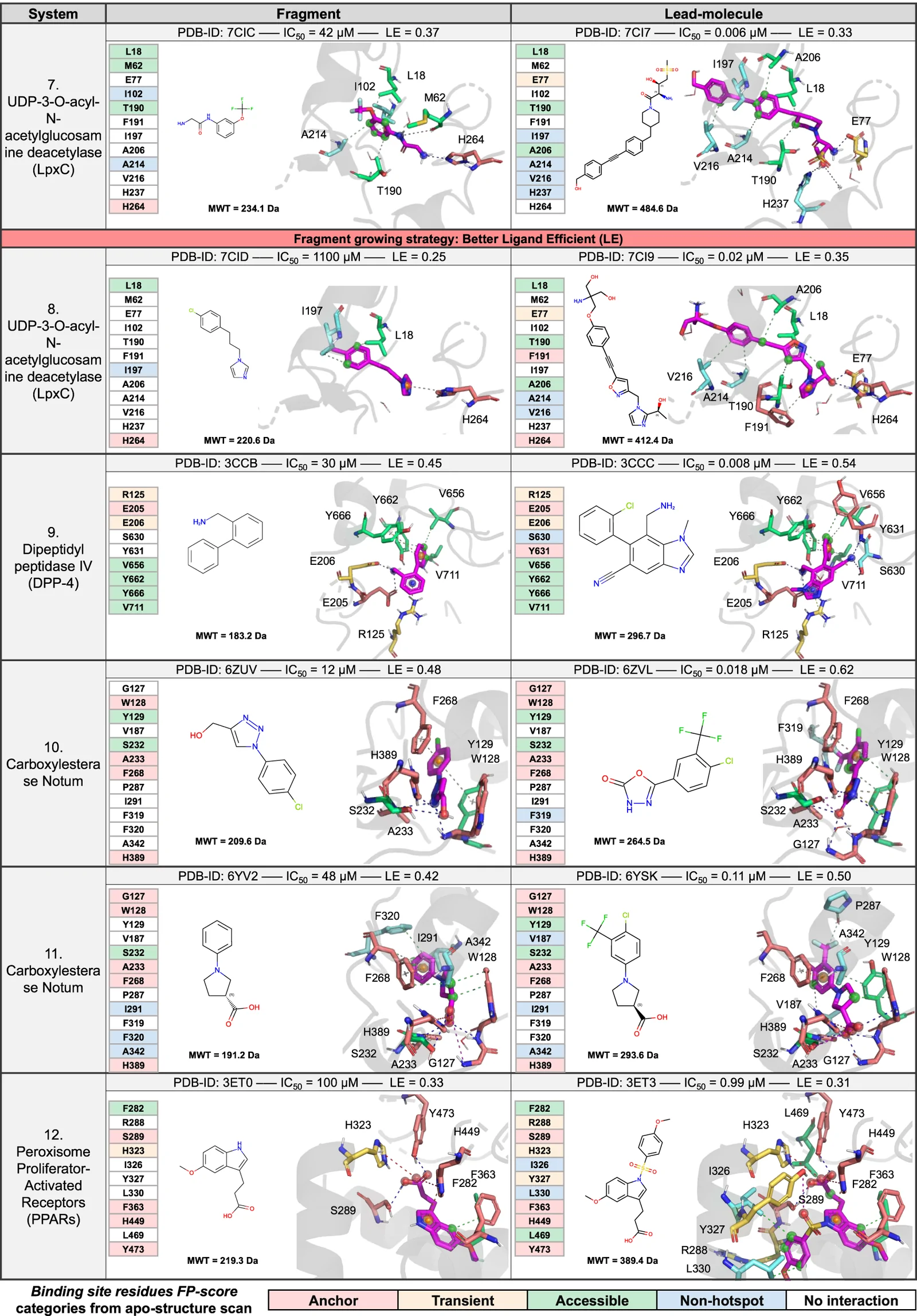
Comparison between fragment growing strategies.

Multiple fragments growing case studies have been assessed and reported, (Fig. 6). FMOPhore scan using the probes library on apo-structures of each system and FP-score was used to categorize the binding site residues (colour-coded) in different categories.

In several examples (Fig. 6, Systems 1-7) an increase in the molecular size delivers a concomitant increase in potency. However, the LE can decrease dramatically in comparison to the starting fragment indicating a less-than-optimal design strategy. In such cases an FMOPhore analysis on the fragment and lead molecules suggested that lead-molecules are either not gaining or losing critical Anchor-category residues (Classified by FMOPhore apo-structure scanning).

Conversely, those systems that show a major improvement in IC_50_ values while improving or maintaining LE values, gain a critical Hotspot-category interaction (Fig. 6, Systems 8-12). This shows that FMOPhore scans on the apo-structures were able to accurately quantify and classify the hotspot residues that can be critical for gaining potency and delivering an efficient design.

By establishing interactions with key Hotspot residues while avoiding those non-hotspot-category residues, better strategies can be implemented beforehand for designing or growing fragments into lead compounds.

### Proteasome β5 subunit case study on drug-resistance hotspots

A recent drug discovery programme targeting the T. cruzi parasite, led to the invention of GSK3494245/DDD01305143, (PDB-ID 6QM7) (Supplementary Fig. 3), which selectively binds to a unique binding site between the β4 and the β5 subunit of the Leishmania proteasome, as revealed by structural (cryo-EM) studies^33^. These structural studies revealed that this is a unique binding site, distinct from the human site, explaining the high levels of selectivity. This compound has a potent activity against L. dononvani and is active in animal models of VL and has been advanced to clinical studies^34^.

One of the compounds produced during the lead optimisation programme for GSK3494245/DDD01305143 was compound 7a (Supplementary Fig. 3)^35^. This compound showed potent inhibition of the T. cruzi proteasome and of intracellular T. cruzi amastigotes.

Given the high similarity of genomic sequence between these parasites, it is hypothesized that this mechanism could potentially serve in treatment of the three diseases. Multiple preclinical and clinically advanced candidates exhibit a promising inhibitory mechanism against the parasite proteasome demonstrating distinct selectivity between humans and kinetoplastid parasites. Therefore, it is imperative to highlight the critical hotspot residues of the kinetoplastid proteasome which can aid medicinal chemists in designing highly selective inhibitors against targeted Kinetoplastid parasite proteasome.

### *FMOPhore* on the Proteasome β5 subunit

A validation case study was performed using FMOPhore scanning with the wild type of apo-structure of 20S Proteasome binding site located between the β4 and β5 subunits.

The key Anchors-category residues identified were Phe-24, Ile-27, and Ile-29, in the β5 subunit, and Tyr-113, Phe-126, Val-128, Gly-129, Ser-132, and Tyr-136, which shows high accessibility and favourable interaction energy residues with scanned probes. Ser-127 is within the Accessible-category residue, whilst the remainder of residues in the binding site were classified as non-hotspots-category, (Supplementary Fig. 3).

A recent mutagenesis study (Altmann et al.)^36^, presented an excellent opportunity to compare our results with empirical data for all possible mutations around the compound 7a drug-binding pocket. Remarkably, the saturation mutagenesis study also identified Gly-98, Tyr-113, Val-128, Ser-132, and Tyr-136 as compound 7a resistance hotspots, suggesting their critical role in maintaining ligand interaction and stability. All these residues were predicted to be Anchor-category residues using FMOPhore scanning, except for the Gly-98. Conversely, mutations in other residues e.g., Thr-1, Ala-46, Gly-47, Asp-115, and Ser-130 as per suggested in the study (Altmann et al.) did not show resistance to compound 7a, indicating minimal to no impact on ligand binding within the binding site. All these residues were predicted to be Non-hotspots.

Among other hotspot residues that were detected by FMOPhore as key hotspots is the Phe-24, which was not included in the experimental mutation scan, yet it has been identified as a key binding residue in previous studies, as mentioned in (Khare et al.)^37^. However, Gly-98 was identified as a hotspot residue in the experimental scan but was not detected by the FMOPhore analysis. Conversely, Gly-129 was predicted as a hotspot by FMOPhore but was not confirmed by the experimental results. To investigate this discrepancy, the binding pose of compound 7a was evaluated.

### *P-score* Protocol and *Dy-FMOPhore* on compound 7a

For evaluation of the binding pose of the 7a ligand, the P-score protocol^38^ was performed. Upon analysis of the MD-trajectory using Dy-FMOPhore, protein ligand interactions showed that the ligand forms a constant hydrophobic interaction with Phe-24, Phe-126, and Val-128, while maintaining a stable electrostatic interaction with Gly-129, and Ser-132, (Supplementary Fig. 3). The PIEDA analysis of the most representative pose, shows that the carboxylic moiety on the ligand is forming a strong hydrogen bond (electrostatic interaction) with the Gly-129 amine group-backbone interaction. This confirmed several of the hotspot predicted residues by the FMOPhore hypothesis above.

Accordingly, upon mutation of the Gly-129, to any other binding site residue, the parasite shows no resistance simply as it is a backbone interaction, so the binding pose of the carboxylic moiety on the ligand will maintain the same backbone interaction with any other amino acid substituting the Gly-129. Yet this is the contrary for the Gly-98, where upon mutation to any other residue, the fluoride atom is in the vicinity to the Gly-98, therefore, longer side chains from other amino acids will act as a stick that may clash with the fluoride group disrupting ligand binding.

### *FP-Score* for selective hotspot strategies

Another way of applying the FMOPhore algorithm is in identifying hotspots for selectivity studies. By being able to classify residues, FMOPhore can be used to develop selective fragment growing strategies by identifying the key residues that fall into different hotspot categories. A good example to demonstrate its utility is the Janus Kinases JAK2 and JAK3^39,40^. The Janus Kinase family is a small group of receptor-associated signalling molecules crucial for the signal cascade. The inhibition of tyrosine kinases’ enzymatic activity with small molecules can play a key role in the treatment of different forms of malignancy. Here the FP-score plots shows that residues that fall in the same vicinity of the binding site such as Asp-A-994 (JAK2), and Asp-A-967 (JAK3) are classified as Anchor-category residues, (Supplementary Fig. 4).

However, FMOPhore identifies that Gly-A-993 (JAK2) as an Accessible-category residue, while in the same position in JAK3 it is substituted with Ala-A-966, that falls in non-hotspot-category residues. This is mainly because of the carbonyl moiety of the Gly-A-993 that is projected towards the binding site while it is muted with the methyl group of the Ala-A-966.

### Recall percentage and comparison between *FMOPhore* modes

**Fig. 7:**
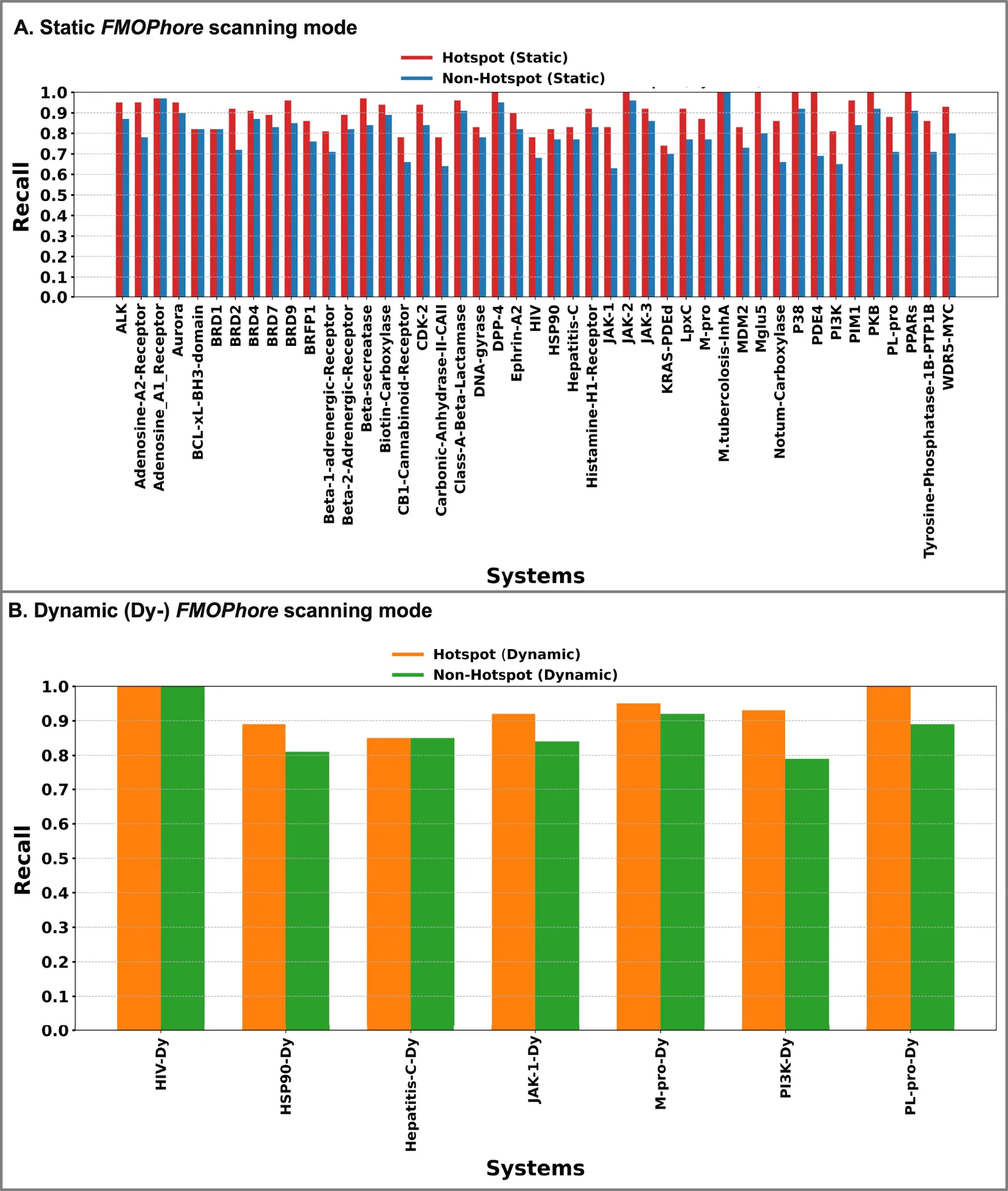

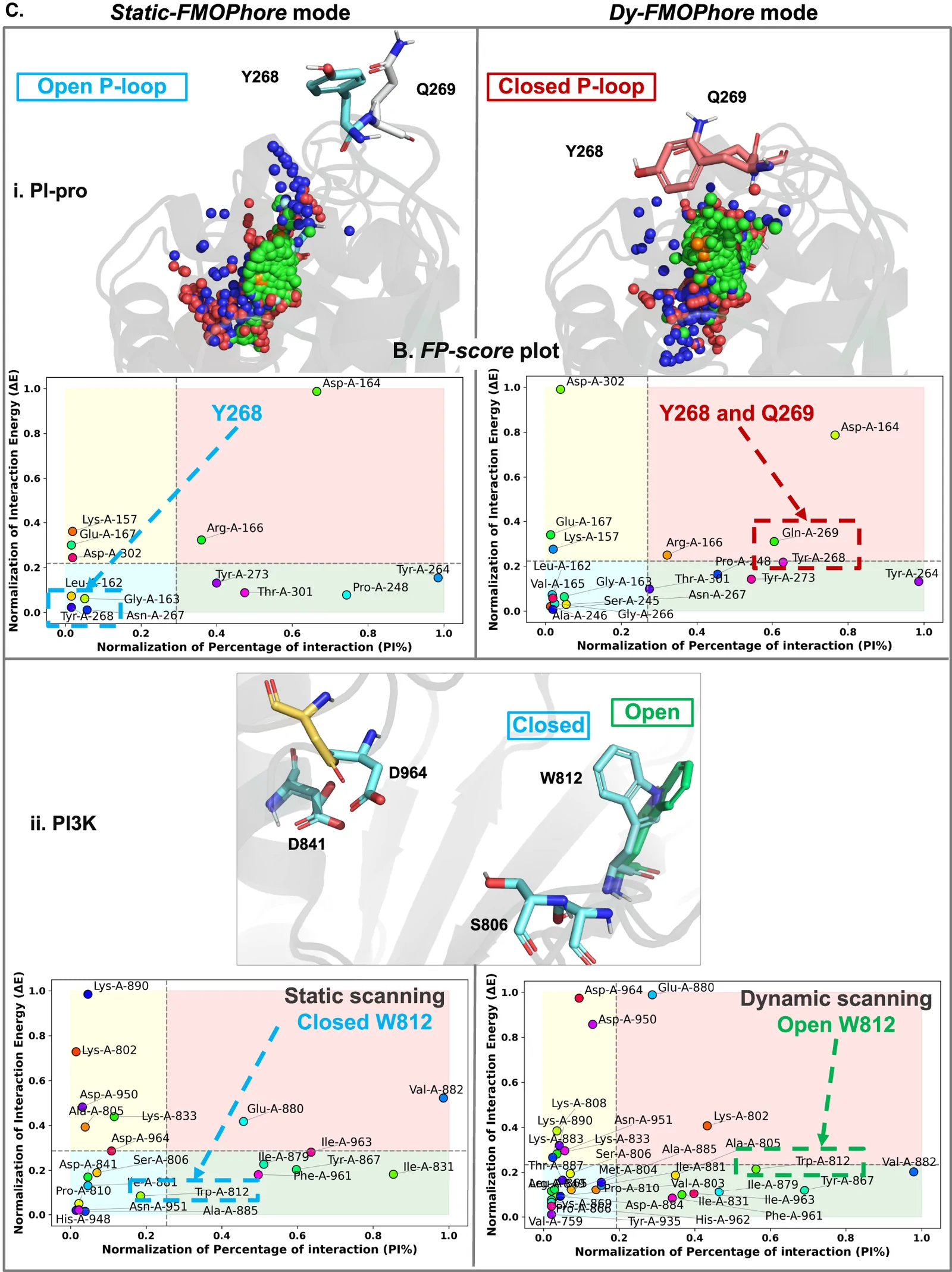
Recall percentage between Holo-complex and Apo-scan FMOPhore scenarios on static and dynamic modes. **A** Recall percentage of the hotspots and non-hotspots for the 45 different systems using static-FMOPhore. **B** A Dy-FMOPhore mode scan for 7 systems (System*-Dy). **C** Comparison between static and dynamic FMOPhore apo-scanning mode. Binding site residues are coloured according to the FP-score categories by FMOPhore scan on apo-structures. (i) PL-pro. (ii) PI3K.

The recall of the hotspots and non-hotspots for the 45 different systems are illustrated in a bar-chart, (Fig. 7A.). A recall value of 1.0 means 100% accurate match between the hotspots categorized by FMOPhore on apo-structure to hotspots categorized on the holo-complexes of the same protein target.

In most systems the recall for hotspots is found to be above 0.8 (80% of the binding site residues are correctly categorized as hotspots using the apo-structure scanning) except for few systems; HIV, CB1-Cannabinoid receptor, Carbonic-anhydrase-II and KRAS-PDEd. Some systems showing a recall of 1.0, e.g., JAK-2, PKB, PPARs, and p38. Other systems, such as Aurora-A, Beta-secretase, Biotin, CDK, and Ephrin-A2, demonstrate high recall values for hotspots of more than 0.9 accuracy.

We performed Dy-FMOPhore on 7 different systems that reported a recall lower than 0.9 in the static-FMOPhore scanning mode (Fig. 7A). Dy-FMOPhore delivers a significant improvement of hotspot and non-hotspot recall accuracy in the seven systems studied (Fig. 7B). All systems showed an improvement in the recall accuracy where some prediction improved between 90% to 100%, e.g., HIV, JAK-1, M-pro, PI3K, and PL-pro.

A good example that demonstrates the success of Dy-FMOPhore is the PL-pro. PL-pro binding site is known to be formed by a P-loop (Tyr-A-268, and Gln-A-269) that is highly flexible, we observed that the binding site is formed upon the induced-fit binding event that happened between the binding site residues and the ligands^41–43^.

By scanning the PL-pro apo-structure (PB-ID: 6W9C) (Fig. 7C. i.), using static-FMOPhore mode, it was found that FP-score categorizes Asp-A-164, and Arg-A-166 as the key Anchor-category residues, while Tyr-A-268 was classified as non-hotspot. However, by scanning the closed binding site conformation of the P-loop, the resulted shows a shift of the Tyr-A-268 from the non-hotspot category (static-FMOPhore mode) to the hotspot-category (Dy-FMOPhore mode). Also, on the open conformation of the P-loop the Gln-A-269, was not detected in the static-FMOPhore scan yet appeared in the Hotspot-category on the closed P-loop conformation.

Another application of Dy-FMOPhore on the Phosphoinositide 3-kinase receptor (PI3K)^44^ (Fig. 7C. ii.), highlights that in the static scanning of the binding site the Trp-A-812 was in a closed conformation and makes the orientation of the indole ring not in favourable 3D-geometry to form a π–π interaction with the probes. By applying Dy-FMOPhore, the ensemble of the simulation allowed the rotation of the side chain that led to opening of the indole ring. This is shown on the FP-score plot, where the Trp-A-812 was categorized in the non-hotspot category and shifted to the Accessible-category using the Dy-FMOPhore. The scanning of different conformations of the binding site and taking the average FP-score per residue, results in the increase in the accuracy of classifying hotspots.

## Discussion

FMOPhore algorithm integrate two descriptors in FP-score to define and categorize binding site residues with regards to their accessibility (Percentage of Interaction; PI%) and interaction energy (ΔE^FMO^). It categorizes binding site residues into four distinct categories: Anchor, Transient, Accessible, and Non-hotspots. By scanning an apo-structure with a small library of probes we can accurately replicate QSAR models represented by the holo-complex ligands and active hits. This is demonstrated in the consistency of high recall rates for hotspots across the 46 different systems.

This FMOPhore protocol delivers an insight into the quantitative structure-activity relationship (QSAR) between bound molecules and the protein complex. It can also provide defined strategies for fragment growing approaches to maximize ligand efficiency (LE) by targeting prioritized Hotspot residues. We have demonstrated that the growing of small molecules in the direction of key hotspot residues in the binding site can lead to better LE and potency of lead compounds with respect to growing molecules without a predetermined strategy. Similarly, we have highlighted how FMOPhore can be used to drive selective design in kinases and prediction of drug resistance hotspots in the Proteasome β5 subunit case study.

The FP-score quadrant matrix along with the 2D and 3D-FMOPhore heatmaps can provide medicinal chemists with a comprehensive visual representation of binding site interactions, combining quantitative interaction percentages with qualitative interaction energy assessments to facilitate the identification of key binding hotspots and guide future design efforts. By focusing on residues with high interaction percentages and strongly negative free energy values, researchers can streamline the identification of promising binding features. By integrating dynamics, we have demonstrated that Dy-FMOPhore, can increase the accuracy of categorizing binding site residues into hotspots and non-hotspots, for systems with high flexibility or large binding pocket. One of the key strengths of FMOPhore is that it allows an in-silico prediction of the effect of modifications to a fragment / compound on the interactions with the protein recalling multiple fragments growing strategies.

In summary, we have introduced and validated our approach to identifying protein ligand hotspots using quantum mechanics and accessibility that can be used to drive efficient and selective design strategies for drug discovery.

## Methods

FMOPhore algorithm integrates FMO using GAMESS to calculate interaction energies described by the inter-fragment interaction energy (IFIE) and its pairwise interaction energy decomposition analysis (PIEDA)^45–48^.

This allows the estimation of protein–ligand interactions with quantum (electronic) effects using a low level of computational resources. By means of PIEDA, the FMO interaction energy; (ΔE^FMO^), is calculated as the sum of five energy terms: electrostatic (ΔE^ES^), exchange repulsion (ΔE^EX^), dispersion (ΔE^DI^), charge transfer with higher-order mixed terms (ΔE^CT + mix^), and solvation energy, as shown in Eq. (1). Accordingly, the chemical nature of the interaction (electrostatic or hydrophobic) can be qualitatively determined, which offers a detailed information regarding protein–ligand interactions in implicit water solvent Polarizable Continuum Model (PCM)^49^.

1 $${{{\mathbf{\Delta }}}{{\bf{E}}}}^{{{\bf{FMO}}}}\,={{{\mathbf{\Delta }}}{{\bf{E}}}}_{{{\bf{ij}}}}^{{{\bf{es}}}}+\,{{{\mathbf{\Delta }}}{{\bf{E}}}}_{{{\bf{ij}}}}^{{{\bf{ex}}}}+\,{{{\mathbf{\Delta }}}{{\bf{E}}}}_{{{\bf{ij}}}}^{{{\bf{ct}}}+{{\bf{mix}}}}+\,{{{\mathbf{\Delta }}}{{\bf{E}}}}_{{{\bf{ij}}}}^{{{\bf{DI}}}}+\,{{{\mathbf{\Delta }}}{{\bf{E}}}}^{{{\bf{Gsol}}}}$$

Fragmentation of protein-ligand complexes is carried out according to a well-established fragmentation strategy by Facio^50^. For high speed with optimal accuracy FMO2-DFTB3 (Density Function Tight-Binding) theory level is used^38,51–54^.

FMOPhore quantify protein-ligand interactions using PLIP^55^ which identifies various bond types including hydrophobic interactions, hydrogen bonds (both donors and acceptors), salt bridges, π–cation interactions, π–π stacking, water bridges, halogen bonds, and metal complexes. These interaction types are used to generate protein-ligand (PL) fingerprints. The proportion of ligands that form interactions with a given residue is then calculated and expressed as the Percentage of Interaction (PI%), providing a quantitative measure of residue accessibility, this is formalized in Eq. (2).

2 $${{\boldsymbol{PI}}}\,\left(\%\right)=\frac{{{\boldsymbol{Frequency}}}\,{{\boldsymbol{of}}}\,{{\boldsymbol{Ligand}}}\,{{\boldsymbol{interactions}}}\,{{\boldsymbol{per}}}\,{{\boldsymbol{residue}}}}{{{\boldsymbol{Total}}}\,{{\boldsymbol{number}}}\,{{\boldsymbol{of}}}\,{{\boldsymbol{interactions}}}}*{{\bf{100}}}$$

The Percentage of interaction with a binding site residue represents the frequency of ligands interactions per residue is the number of ligands that interact with the residue. Total number of interaction opportunities is the total number of ligands scanned for that protein.

To measure how frequently each binding site residue interacts across different ligands, we calculate the Percentage of Interaction (PI%), which reflects the residue’s accessibility and involvement in binding across the ligand dataset. The percentage of interaction with a binding site residue is calculated as the proportion of protein-ligand interactions observed for a specific residue within the binding site, relative to the total number of sampling points (or ligands) analysed. This metric quantifies how frequently a particular residue interacts with ligands during the sampling process, providing insight into the residue’s significance in ligand binding. A higher percentage indicates that the residue plays a more prominent role in ligand interactions across the dataset.

FMOPhore is based on a scoring function named FP-score that classifies binding site residues in two classes: 1) Hotspot residues (further detailed into three categories; Anchor, Transient, and Accessible) and 2) non-hotspot residues. The FMOPhore methodology can be found in the Supplementary Methods.

### *FP-score* theory for hotspot classification

**Fig. 8:**
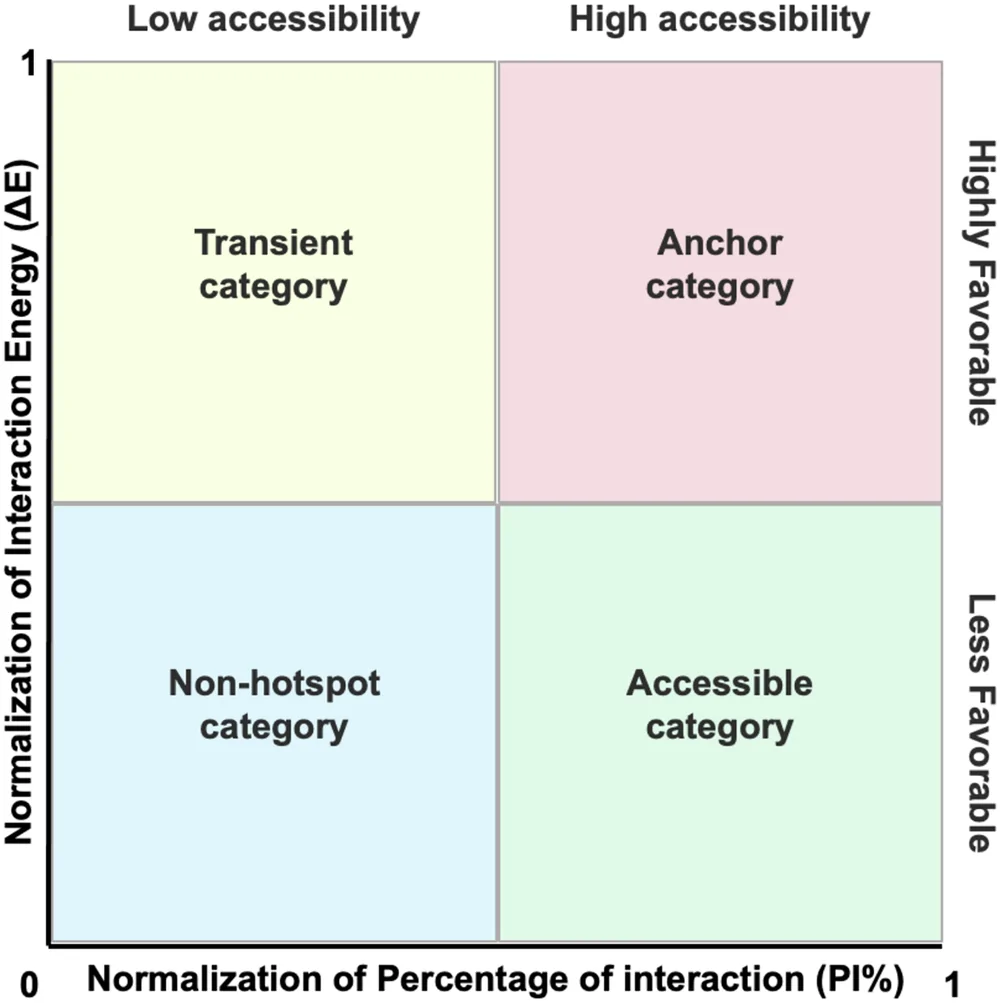
*FP-score* plot (quadrant matrix): four binding site residue categories. Normalization of Percentage of Interaction (PI%) (*x*-axis) and Normalization of Interaction Energy (*E*) (*y*-axis). Vertical line intersecting the *x*-axis is the mean of percentage of interaction energy (PI mean) values, and horizontal line intersecting the *y*-axis is the mean of interaction energy (*E* mean). Top-right red box ➔ Highly prioritized binding site residue. Top-left yellow box ➔ Binding site residue with highly favourable interaction energy but low frequency of interaction. Bottom-right green box ➔ Binding site residue with high frequency of interaction but less binding affinity. Bottom-left blue box ➔ Least prioritized binding site residue.

To be able to accurately classify and score binding site residues at a molecular level, each residue is assigned with the two descriptors mentioned earlier, (1) Percentage of interaction (quantitative term for accessibility), and (2) Interaction energy (qualitative term for the strength of interaction, ΔE^FMO^), and presented in FP-score quadrant matrix plot (Fig. 8) and Eq. (3).

The FP-score as a scoring function, was inspired from the P-score theory that we introduced to accurately predict protein-ligand binding pose in a dynamic environment^41^.

3 $${{{\boldsymbol{FP}}}}_{{{\boldsymbol{score}}}}=	 \left(\frac{\left({{\bf{1}}}+{{\max }}\left({{{\boldsymbol{E}}}}^{{{\boldsymbol{FMO}}}}\right)\right)-\left({{{\boldsymbol{E}}}}_{{{\boldsymbol{a}}}}^{{{\boldsymbol{FMO}}}}\right)}{\left({{\bf{1}}}+{{\max }}\left({{{\boldsymbol{E}}}}^{{{\boldsymbol{FMO}}}}\right)\right)-\left({{\min }}\left({{{\boldsymbol{E}}}}^{{{\boldsymbol{FMO}}}}\right)-{{\bf{1}}}\right)}\right) \\ 	*\left(\frac{\left({{\boldsymbol{PI}}}\right)-\left({{\min }}\left({{\boldsymbol{PI}}}\right)-{{\bf{1}}}\right)}{\left({{\bf{1}}}+{{\max }}\left({{\boldsymbol{PI}}}\right)\right)-\left({{\min }}\left({{\boldsymbol{PI}}}\right)-{{\bf{1}}}\right)}\right)$$

Percentage of Interaction (PI) represents the percentage of interaction with a binding site residue. min(PI) and max(PI) indicate the lowest and highest percentage of interactions in the compared hotspots, respectively. ΔE_a_^FMO^ represents the interaction energy with a binding site residue. min(ΔE^FMO^) and max(ΔE^FMO^): indicate the lowest and highest interaction energy in the compared hotspots, respectively.

1. ***Hotspot class***: Key binding site residues that can potentially be targeted for fragment-growing strategies. Interactions with this class of residues allow the growth of hit fragments while retaining or improving ligand efficiency (LE), lipophilicity, potency, or selectivity^56,57^. It is categorized in three categories:
  - ***Anchor category***: Binding site residues that show high percentage of interaction and highly favourable interaction energy (≤ −5.0 kcal/mol; typically forming hydrogen bonds or strong electrostatic interactions).
  - ***Transient category***: Binding site residues that show low percentage of interaction and highly favourable interaction energy.
  - ***Accessible category***: Binding site residues that show high percentage of interaction and low to moderately favourable interaction energy (−5.0 to −2.5 kcal/mol; Weaker interactions, such as hydrophobic contacts or weak electrostatic attractions).
2. ***Non-hotspot class***: Binding site residues that show a low percentage of interaction and low to unfavourable interaction energy (> 0 kcal/mol; Destabilizing interactions, indicating repulsion or steric clashes).

The FP-score is an evaluation matrix for each binding site residue, obtained by the normalizing the ensemble of two descriptors: percentage of interaction (PI%) and interaction energy (ΔE^FMO^) with a score range between 0 and 1. An FP-score value closer to 1 indicates a highly prioritized hotspot, whereas a value closer to 0 suggests lower priority. A low FP-score may result from low accessibility, unfavourable interaction energy, or a combination of both.

The Anchor category represents a top priority hotspot that is easily accessible. This means that the vicinity surrounding its chemical feature can form highly favourable interactions (≤ −5.0 kcal/mol) with complementary functional groups from the ligand, provided the proper 3D-geometry. Targeting Anchor residues in a fragment-growing study ensures both efficiency and potency during the growing step.

The Transient category consists of binding site residues that can form highly favourable interaction energies; however, they exhibit a moderate to low percentage of interaction (i.e., they are less accessible). This limited accessibility is primarily due to the small 3D geometric volume surrounding the residue’s chemical features, which may expand or contract during molecular dynamics simulations (e.g., the opening or closing of cryptic pockets). If targeted with a properly complementary functional group, Transient residues can play a key role in increasing binding affinity while positively influencing ligand efficiency.

The Accessible category consists of binding site residues that exhibit a high percentage of interaction but low to moderately favourable interaction energy (−5.0 to −2.5 kcal/mol). Targeting an Accessible residue is generally easier than targeting a Transient-category residue due to its high accessibility; however, it may contribute only marginally to improving ligand binding affinity. This is primarily because Accessible residues often engage more lipophilic interactions rather than electrostatic ones.

Finally, the non-hotspot category refers to binding site residues that exhibit low to unfavourable interaction energy (> 0 kcal/mol) and a low percentage of interaction. Non-hotspots should be the least prioritized, as targeting them may result in minimal improvement or even a decrease in binding affinity due to steric clashes.

However, any binding site residue that falls into one of these four categories might still be essential for selectivity purposes.

### Validation dataset for the *FP-score* theory

Forty-six different targets were selected from literature for validation of our *FP-score* theory including the application for fragment growing case studies, the systems are: 1.) Anaplastic Lymphoma Kinase (ALK), 2.) Aurora-A Kinase, 3./4./5./6./7.) Bromodomain-containing protein (BRD-1/−2/−4/−7/−9) 8.) Bromodomain and plant homeodomain (PHD) finger containing protein 1 (BRPF1), 9.) Beta-secretase receptor, 10.) Biotin carboxylase, 11.) Carboxylesterase Notum, 12.) Cyclin-dependent Kinase-2 (CDK-2), 13.) Dipeptidyl peptidase IV (DPP-4), 14.) DNA-gyrase, 15.) Tyrosine kinase EPHA2 (Ephrin type-A receptor 2), 16.) Heat shock protein (HSP90), 17.) Hepatitis-C virus (HCV NS5b RNA polymerase), 18.) Human Immunodeficiency Virus protease (HIV-1), 19./20./21.) Janus Kinase family (JAK-1/−2/−3), 22.) Main-protease protein (M-pro), 23.) M. Tuberculosis-InhA, 24.) Phosphodiesterase 4 (PDE4), 25.) Papain-like protease (PL-pro), 26.) Proto-oncogene serine/threonine-protein kinase (Pim-1) 27.) Peroxisome proliferator-activated receptor (PPARs) gamma, 28.) Phosphoinositide 3-kinase R (PI3KR), 29.) Protein Kinase B (PKB), 30.) p38α MAP kinase, 31.) 2UDP-3-O-acyl-N-acetylglucosamine deacetylase (LpxC), and 32.) WD repeat-containing protein 5 (WDR5), 33.) Tyrosine Phosphatase 1B (PTP1B), 34.) Metabotropic Glutamate Receptor 5 (mGlu5), 35.) Mouse Double Minute 2 homologue (MDM2), 36.) KRAS–Phosphodiesterase delta complex (KRAS_PDEδ), 37.) Human Carbonic Anhydrase II (hCAII), 38.) Histamine H1 Receptor (H1R), 39.) Class A Beta-Lactamase, 40.) Cannabinoid Receptor 1 (CB1), 41.) Beta-1 Adrenergic Receptor (β1AR), 42.) Beta-2 Adrenergic Receptor (β2AR), 43.) BCL-xL BH3 Domain Complex, 44.) Adenosine A2A Receptor (A2AR), 45.) Adenosine A1 Receptor (A1R), 46.) proteasome β5 subunit (PSMB5) as listed in Supplementary Table 1.

### *FMOPhore* application scenarios

Our approach was designed to identify the key interacting residues that drive binding affinity and stability of ligands and fragments in the binding site. Hence, for this study, we considered the experimentally validated binding sites as the focus for apo-structures for each protein target.

#### *Holo*-complexes scenario

The holo-complex scenario is performed by analysing multiple co-crystalized holo-complexes for each system, using FP-score to classify the binding site residues (Hotspots and Non-hotspots). In this work, we provide FMOPhore analysis on two holo-complexes case studies out of the 46 systems. The FMOPhore holo-complex analysis of the other systems is reported in the Supplementary Note 1.

#### *Apo*-structure scenario

**Fig. 9:**
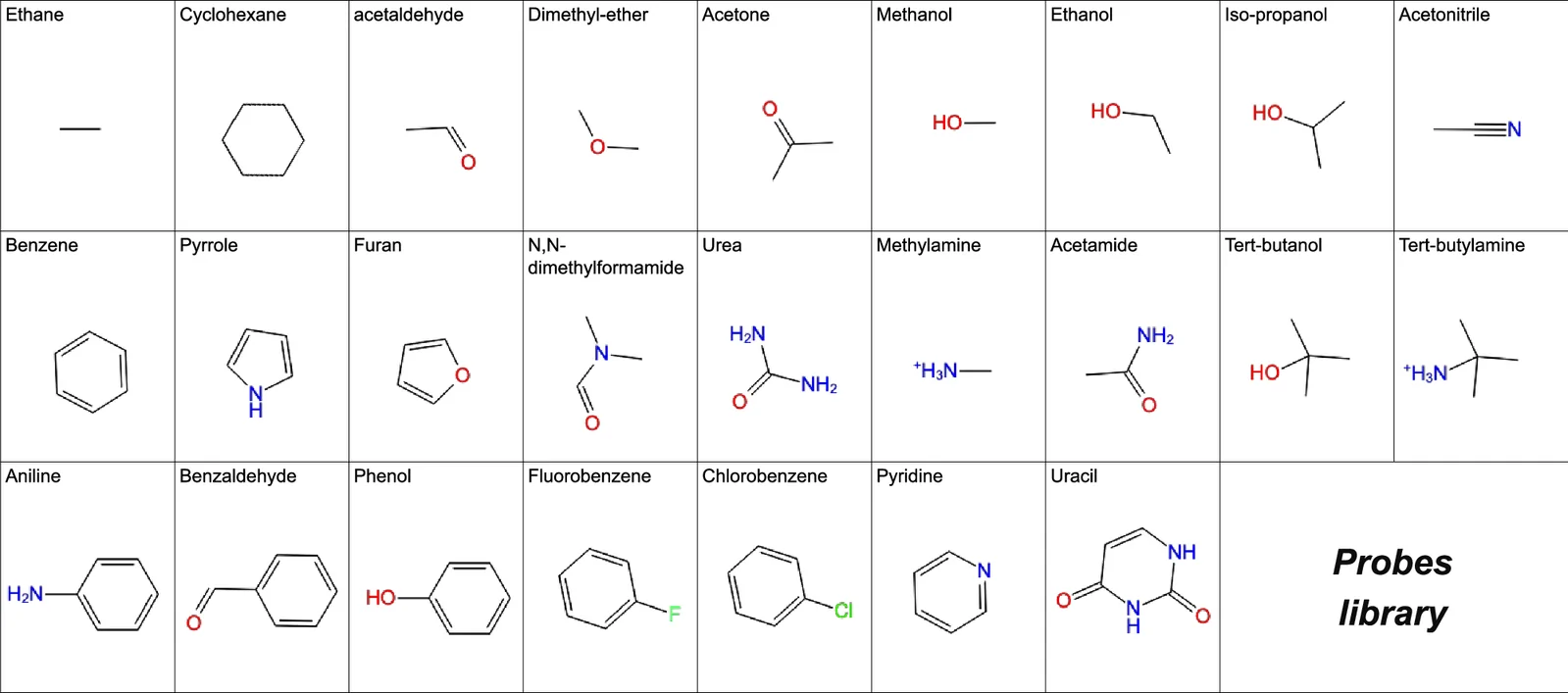
Probes library for scanning the binding site in apo-structures.

In the apo-scenario is used to classify the active binding site residues without prior knowledge of bound ligands. We demonstrate the potential of our algorithm in predicting and classifying hotspots on the apo-structure per system using a small library of twenty-five functional probes (Fig. 9). The probes represent a diverse set of pharmacophore features. The rationale behind the small library of probes is to have a good coverage of possible interaction bond types that are relevant to drug design; hydrogen bond acceptor, hydrogen bond donor, hydrophobic, π–π-stacking, π-cation stacking, salt-bridges, halogen bond, and water-bridges.

To test the robustness of the FP-score in classifying binding site residues (either Hotspots or Non-hotspot), the percentage of recall is calculated by comparing and quantifying the apo-structure classification output for each system to the actual hotspots identified from the holo-complex crystal structures. We wanted to see if the hotspots identified in the apo-structure were confirmed in the holo-complex. We defined this with the recall; Eq. (4), represented in true positive: if a hotspot is detected in apo-structure scanning and confirmed by holo-complex analysis, and false negative: if a hotspot residue is not detected by apo-structure scanning:

4 $${Recall}=\frac{{True}\,{positive}}{{True}\,{positive}+{False}\,{negative}}$$

### FMOPhore modes

In both FMOPhore application scenarios, analysis of co-crystalized ligands (holo) or the binding site scanning (apo), FMOPhore can be performed in a static or dynamic mode to include protein flexibility.

#### Static-FMOPhore mode

For comparison between the holo-complex and apo-structure scenarios, we run a static FMOPhore scanning using an apo-structure for each system. To conduct static hotspot scanning, each system was prepared using the ‘protein preparation wizard’ in Maestro (Schrodinger, LLC)^58^. This involved adding bond orders and formal charges to hetero groups and hydrogens to all atoms. The structures are then refined, and the hydrogen bond network optimized using the OPLSe force field. For the apo-structure scenario, docking of the probes library is performed on the protein structure grid for each system using the standard precision (SP) mode as per the Glide docking modules of the Maestro 12.6.144 software (Schrodinger, LLC, New York, NY, USA)^59–61^, and Ligprep (Schrodinger, LLC)^58^.

FMOPhore analysis is conducted on the docked probes to evaluate the interaction energy and accessibility of the binding site residues with the probes. This is represented in an FP-score plot (quadrant matrix), a FP-score bar-chart, and a 3D-FMOPhore model.

#### Dynamic-FMOPhore mode

The consideration of protein’s dynamic nature is critical in classifying hotspots. This can lead to the opening and closing of cryptic pockets and the exposure of different accessibility of binding site residues. Static-FMOPhore allows binding site scanning on a single conformation of the binding site, while Dynamic-FMOPhore (Dy-FMOPhore) takes the average of the FP-score of each binding site residue throughout the ensemble of the different binding site conformations, describing protein-ligand interactions from different snapshots along the trajectory.

With the Dy-FMOPhore, it is possible to assess interaction throughout a full MD simulation trajectory, wherein the probes were allowed to scan different binding site conformations. To perform dynamic scanning “Dy-FMOPhore”, protein structures are prepared with the protein preparation wizard as implemented in Maestro^59^. Hydrogen atoms are added to the complex, and missing atoms in protein side chains were built according to the AMBER16 force field topology^62^, and supervised molecular dynamics (SuMD) was used^63–65^. Details on the Dy-FMOPhore protocol are reported in the Supplementary Note 2.

### Reporting summary

Further information on research design is available in the Nature Portfolio Reporting Summary linked to this article.

## Supplementary information

Supplementary Information

Description of Additional Supplementary Files

Supplementary Dataset 1

Supplementary Movie 1

Reporting Summary

Transparent Peer Review file

## Source data

Source Data

## Data Availability

All data supporting the findings of this study are available within the paper and its Supplementary Information file. All protein structures and associated ligands were obtained from Protein Data Bank (PDB; https://rcsb.org) and are listed in Supplementary Table 1. The Initial and final snapshots for the molecular dynamics’ simulations are provided as Supplementary Dataset 1. A dynamic visualization of the Dy-FMOPhore analysis for the PDB-ID: 7S3S Mpro-protein system is available as Supplementary Movie 1. All additional data supporting the figures in the main manuscript are provided as Source Data. Source data supporting the analyses described in Supplementary Notes are also provided as Source Data. A detailed description of all supplementary datasets and files is available in the document titled “Description of Additional Supplementary Files”. Source data are provided with this paper.

## Code availability

The code used in this study is publicly available and has been deposited in a GitHub repository at https://github.com/DDU-CDD/fmophore, and Zenodo https://zenodo.org/records/23080112 under GPL, CC-BY 4.0.
