# Supplementary material for: FMOPhore for hotspot identification and efficient fragment-to-lead growth strategies
Source: Nat Commun. 2026 Apr 28;17:5813. doi: 10.1038/s41467-026-72384-x (PMC13328607; doi:10.1038/s41467-026-72384-x)
Supplement: Supplementary file 1 — Supplementary Information [file 41467_2026_72384_MOESM1_ESM.pdf]

Supplementary information

FMOPhore for hotspot identification and efficient fragment-to-lead growth strategies

Peter E.G.F. Ibrahim, *[1,2], Simone Altmann,[2] Ulrich Zachariae,[2,3] David Horn,[2] Ian H. Gilbert[1,2] and Michael J. Bodkin*[1,2]

[1] *Drug Discovery Unit, Wellcome Centre for Anti-Infectives Research, School of Life Sciences, University of Dundee, Dow Street, Dundee. DD1 5EH, UK.*
[2] *School of Life Sciences, University of Dundee, Dow Street, Dundee DD1 5EH, UK.*
[3] *Division of Biological Chemistry and Drug Discovery, School of Life Sciences, University of Dundee, DD1 5EH, UK.*
For correspondence, please contact email: peteregfi@gmail.com

Table of Contents

Supplementary information 1

Supplementary Methods 4

FMOPhore Methodology: 4

Protein–Ligand Complex Preparation 4

Input Generation for FMOPhore 4

Fragmentation Strategy and FMO 4

FMO Settings (via GAMESS) 6

Pharmacophore Detection 6

FP-score Analysis 6

Supplementary Table 1 8

PDB-IDs per system and ligands SMILES 8

Supplementary Notes 39

Note 1: FMOPhore analysis per system 39

Anaplastic Lymphoma Kinase (ALK) 40

Aurora-A Kinase 41

Bromodomain-containing protein (BRD-1) 42

Bromodomain-containing protein (BRD-2) 43

Bromodomain-containing protein (BRD-4) 44

Bromodomain-containing protein (BRD-7) 49

Bromodomain-containing protein (BRD-9) 50

Bromodomain and plant homeodomain (PHD) finger containing protein 1 (BRPF1) 51

Beta-secretase receptor 52

Biotin carboxylase 53

Carboxylesterase Notum 54

Cyclin-dependent Kinase-2 (CDK-2) 55

Dipeptidyl peptidase IV (DPP-4) 56

DNA-gyrase 57

Tyrosine kinase EPHA2 (Ephrin type-A receptor 2) 58

Heat shock protein (HSP90) 59

Hepatitis-C virus (HCV NS5b RNA polymerase) 60

Human Immunodeficiency Virus protease (HIV-1) 61

Janus Kinase family (JAK-1) 62

Janus Kinase family (JAK-2) 63

Janus Kinase family (JAK-3) 64

Main Protease Protein (Mpro) 65

M. Tuberculosis-InhA 66

Phosphodiesterase 4 (PDE4) 67

Papain-like protease (PL-pro) 68

Proto-oncogene serine/threonine-protein kinase (Pim-1)....69
Peroxisome proliferator-activated receptor (PPARs) gamma....70
Phosphoinositide 3-kinase R (PI3KR)....71
Protein Kinase B....72
p38α MAP kinase....73
2UDP-3-O-acyl-N-acetylglucosamine deacetylase (LpxC)....74
WD repeat-containing protein 5 (WDR5)....75
Tyrosine Phosphatase 1B (PTP1B)....76
Metabotropic Glutamate Receptor 5 (mGlu5)....77
Mouse Double Minute 2 homolog (MDM2)....78
KRAS–Phosphodiesterase delta complex (KRAS_PDEδ)....79
Human Carbonic Anhydrase II (hCAII)....80
Histamine H1 Receptor (H1R)....81
Class A Beta-Lactamase....82
Cannabinoid Receptor 1 (CB1)....83
Beta-1 Adrenergic Receptor (β1AR)....84
Beta-2 Adrenergic Receptor (β2AR)....85
BCL-xL BH3 Domain Complex....86
Adenosine A2A Receptor (A2AR)....87
Adenosine A1 Receptor (A1R)....88
Proteasome β5 subunit (PSMB5)....89

Note 2: Molecular dynamics simulations protocol for Dy-FMOPhore....90

Dy-FMOPhore analysis of PDB-ID: 7S3S....92
Proteasome β5 subunit....94
Dy-FMOPhore analysis Systems....96

Supplementary Figures....103

Figure 1: FMOPhore analysis on two M-pro Holo-complex structures, PDB-IDs: 7N8C and 7S3S....103
Figure 2: FMOPhore analysis of Biotin carboxylase....104
Figure 3: FMOPhore for Hotspot identification in the Proteasome β5 subunit....105
Figure 4: FMOPhore analysis of Janus Kinases....106

Supplementary References....107

Supplementary Methods

FMOPhore Methodology:

Protein–Ligand Complex Preparation

- Input protein–ligand complexes were downloaded from the Protein Data Bank (PDB).
- Structural integrity was checked using PDBFixer to add missing atoms, and correct residue naming.
- Final refinement was carried out in Maestro's Protein Preparation Wizard:
    - Assign protonation states at pH 7.4
    - Optimize H-bond network
    - Minimize heavy atoms (restrained)

Input Generation for FMOPhore

- The prepared .pdb file is directly fed into the FMOPhore pipeline.
- The code:
    - Automatically detects the ligand (HETATM ligand name except the HOH presented in the code: https://github.com/DDU-CDD/fmophore).
    - Defines residues within a default 5 Å radius
    - Splits the complex into FMO fragments
    - Generates GAMESS-compatible input files

Fragmentation Strategy and FMO

- Fragmentation is implemented via the Facio system.
- Protein residues are treated as individual fragments.
- The ligand is preserved as a single fragment.
- Interactions across fragments are preserved to support accurate IFIE calculations.

The Fragment Molecular Orbital (QM-FMO) approach is a QM method that segments proteins or nucleic acids into smaller substructures such as 3-body (or more components), performs the QM calculation then recombines the outcome. The approach is considerably faster than a conventional multibody QM approach with little cost in accuracy. Since its introduction by *Kitaura et al*. in 1999, the QM-FMO method has gained widespread recognition and application in the drug discovery field.[1]

QM-FMO evaluates the binding interaction energies described by the inter-fragment interaction energy (IFIE) and its pair interaction energy decomposition analysis (PIEDA).[2] Inter-fragment interaction energy (IFIE) analysis estimates protein–ligand interactions with quantum (electronic) effects using a modest level of computational resources. By means of pair interaction energy decomposition analysis (PIEDA), the FMO interaction energy, ΔE^{FMO}, is calculated as the sum of five energy terms: electrostatic (ΔE^{ES}), exchange repulsion (ΔE^{EX}), dispersion (ΔE^{DI}), charge transfer with higher-order mixed terms ($\Delta E^{CT + mix}$), and solvation energy (ΔE^{Gsol}) (Equation 1).

QM-FMO offers a detailed estimation of protein-ligand interactions in an implicit water, polarizable continuum model (PCM), that has been of great value in a range of drug discovery projects.

$$\Delta \mathbf{E}^{\mathbf{FMO}} = \Delta \mathbf{E}_{\mathbf{ij}}^{\mathbf{es}} + \Delta \mathbf{E}_{\mathbf{ij}}^{\mathbf{ex}} + \Delta \mathbf{E}_{\mathbf{ij}}^{\mathbf{ct+mix}} + \Delta \mathbf{E}_{\mathbf{ij}}^{\mathbf{DI}} + \Delta \mathbf{E}^{\mathbf{Gsol}} \qquad \mathbf{(1)}$$

The workflows perform:

1- Protein-ligand complex fragmentation strategy
2- Interaction energy calculation (IFIE)
3- QM calculation at DFTB-3
4- PIEDA analysis

Fragmentation of protein-ligand complexes is carried out according to a well-established fragmentation strategy by Facio, where a given amino acid, along with its side chain, C-alpha, and backbone NH, and the carbonyl group of the adjacent amino acid, define each FMO fragment.[3]

The workflows have been developed with full integration of a pharmacophore modelling using PLIP, in the algorithm "*FMOPhore*" and its associated dynamic version named "*Dy-FMOPhore*".

The protein-ligand complex is partitioned into smaller fragments. For binding site residues, fragmentation is performed at the level of individual amino acids, with the covalent backbone broken between the Cα and C=O bond of the preceding residue. To preserve the electronic environment and avoid artificial termini effects, covalent bond cuts are capped with hydrogen atoms (the “link atom” approach), positioned along the original bond vector at an optimal distance based on standard covalent radii. This ensures that the capped fragments mimic the natural bonding and electrostatic environment of the intact protein, allowing accurate quantum mechanical energy decomposition analysis. In ligand-protein complexes, the ligand is typically treated as a separate fragment, enabling direct calculation of inter-fragment interaction energies between the ligand and individual binding site residues.

FMO Settings (via GAMESS)

- Method: DFTB3
- Basis set: Minimal basis set (internally specified via DFTB parameters)
- Charge/spin: Neutral, singlet
- Solvation model: Polarizable continuum model (PCM)
- SCF convergence: Tight (default GAMESS thresholds)

Pharmacophore Detection

- After FMO calculation, the PLIP tool is used to extract:
    - H-bond donors/acceptors
    - Hydrophobic contacts
    - π-π and cation-π interactions
    - Salt bridges
    - Water bridges and metal coordination

FP-score Analysis

- A scoring function computes per-residue FP-score using:
    - Interaction energy (ΔE) from FMO output

  - PI% – frequency of ligand-residue interactions
- Binding site residues are categorized as:
  - Anchor (Red shaded): High ΔE and high PI%
  - Transient (Yellow shaded): High ΔE or low PI%
  - Accessible (Green shaded): Low ΔE and high PI%
  - Non-hotspot (cyan shaded): Low ΔE and low PI%

Supplementary Table 1

PDB-IDs per system and ligands SMILES

Table 1: All PDB-IDs with ligands; in SMILES format, used for holo-complex structures analysis.

Aurora Kinase A				
PDB ID	Ligand Name	Ligand MW	Ligand-smiles	URL
3H0Z	45B	581.26	CC(=O)N1CCN(C(=O)Cc2ccc(Nc3ncc(F)c(Nc4ccc(C(=O)Nc5ccccc5C)cc4)n3)cc2)CC1	https://www.rcsb.org/structure/3H0Z
3UOH	0C4	318.13	Bc1ccccc1Nc1ccnc(Nc2ccc(C(=O)O)cc2)n1	https://www.rcsb.org/structure/3UOH
3UO4	0C0	382.14	O=C(O)c1ccc(Nc2nccc(Nc3ccccc3-c3ccccc3)n2)cc1	https://www.rcsb.org/structure/3UO4
4DED	NHU	348.13	NC(=O)c1ccc(Nc2nccc(Nc3ccccc3C(N)=O)n2)cc1	https://www.rcsb.org/structure/4DED
3UO6	0BY	320.13	Cc1ccccc1Nc1ccnc(Nc2ccc(C(=O)O)cc2)n1	https://www.rcsb.org/structure/3UO6
3UOD	0C3	374.1	O=C(O)c1ccc(Nc2nccc(Nc3ccccc3C(F)(F)F)n2)cc1	https://www.rcsb.org/structure/3UOD
5AAD	5GX	385.2	CC1=C(c2cnn(Cc3ccccc3)c2)C2NC(c3cn(C)nc3C)N=C2N=C1	https://www.rcsb.org/structure/5AAD
3UP7	0C9	350.1	O=C(O)c1ccc(Nc2nccc(Nc3ccccc3C(=O)O)n2)cc1	https://www.rcsb.org/structure/3UP7
5AAE	7HD	388.18	Cc1cc(Cn2cc(-c3c(C)cnc4nc(-c5cn(C)nc5C)[nH]c34)cn2)no1	https://www.rcsb.org/structure/5AAE
4DEB	NHJ	373.12	NC(=O)c1ccc(Nc2nccc(Nc3cccc(C(F)(F)F)c3)n2)cc1	https://www.rcsb.org/structure/4DEB
5AAF	NL4	454.22	Cc1cnc2nc(-c3cn(C)nc3C)[nH]c2c1-c1cnn(Cc2cccc(C(=O)N(C)C)c2)c1	https://www.rcsb.org/structure/5AAF
3UP2	0C8	390.09	O=C(O)c1ccc(Nc2nccc(Nc3ccccc3OC(F)(F)F)n2)cc1	https://www.rcsb.org/structure/3UP2
2W1G	L0G	381.19	O=C(Nc1c[nH]nc1-c1nc2cc(CN3CCOCC3)ccc2[nH]1)NC1CC1	https://www.rcsb.org/structure/2W1G
3COH	83H	348.2	CCN1C(=O)C(C)(C)c2cc3[nH]c4c(c3cc21)CCCc1c-4n[nH]c1C	https://www.rcsb.org/structure/3COH
4JBO	WPH	536.6	CN(C)CCOc1ccc(cc1)c2cc3c(ncnc3o2)NCCc4ccc(cc4)NC(=O)Nc5ccccc5	https://www.rcsb.org/structure/4JBO
4JBP	YPH	592.6	c1ccc(cc1)NC(=O)Nc2ccc(cc2)CCNc3c4cc(oc4ncn3)c5ccc(cc5)OCCN6CCC(CC6)O	https://www.rcsb.org/structure/4JBP
2W1D	L0D	184.07	c1ccc2[nH]c(-c3cc[nH]n3)nc2c1	https://www.rcsb.org/structure/2W1D
4JBQ	VX6	466.23	Cc1cc(NC2=CC(N3CCN(C)CC3)NC(Sc3ccc(NC(=O)C4CC4)cc3)=N2)n[nH]1	https://www.rcsb.org/structure/4JBQ
3UOK	0C6	338.12	Cc1ccccc1Nc1nc(Nc2ccc(C(=O)O)cc2)ncc1F	https://www.rcsb.org/structure/3UOK
3UOL	0C7	344.15	Cc1ccccc1Nc1ccnc(Nc2ccc(-c3nn[nH]n3)cc2)n1	https://www.rcsb.org/structure/3UOL
3UNZ	0BZ	324.1	O=C(O)c1ccc(Nc2nccc(Nc3ccccc3F)n2)cc1	https://www.rcsb.org/structure/3UNZ

Beta-Secretase				
PDB ID	Ligand Name	Ligand MW	Ligand-smiles	URL
2OF0	CMZ	265.17	Cc1ccc(C)c(OC[C@@H](O)CN2CCOCC2)c1	https://www.rcsb.org/structure/2OF0

2OHS	9IP	306.15	COc1cncc(-c2cccc(CNc3cccnc3N)c2)c1	https://www.rcsb.org/structure/2OHS
2OHT	IP6	316.17	NC1NC=CC=C1NCc1cccc(-c2ccc3cc[nH]c3c2)c1	https://www.rcsb.org/structure/2OHT
2OHU	IP7	423.21	NC1NC=CC=C1NCc1cc(-c2ccc3cc[nH]c3c2)ccc1OCc1ccccn1	https://www.rcsb.org/structure/2OHU
2OHP	6IP	237.13	Nc1cccc(CCc2ccc3cc[nH]c3c2)n1	https://www.rcsb.org/structure/2OHP
2OHQ	7IP	304.16	COc1cccc(-c2cccc(CCc3cccc(N)n3)c2)c1	https://www.rcsb.org/structure/2OHQ
2OHR	8IP	278.15	NC1NC=CC=C1NCc1cccc(-c2cccnc2)c1	https://www.rcsb.org/structure/2OHR
2OHK	1SQ	146.08	NC1=C2CC=CC=C2C=CN1	https://www.rcsb.org/structure/2OHK
2OHL	2AQ	144.07	Nc1ccc2ccccc2n1	https://www.rcsb.org/structure/2OHL
2OHM	8AP	201.13	NC1NC=CC=C1NCc1ccccc1	https://www.rcsb.org/structure/2OHM
2OHN	4FP	193.13	Fc1ccc(CC2CCNCC2)cc1	https://www.rcsb.org/structure/2OHN
**Biotin Carboxylase**				
**PDB ID**	**Ligand Name**	**Ligand MW**	**Ligand-smiles**	**URL**
2W6P	OA4	252.14	Cc1c(-c2ccccc2)ccc2c1C(N)N=C(N)N2	https://www.rcsb.org/structure/2W6P
2W6Q	OA5	247.25	c1ccc(cc1)OCCOc2nc(nc(n2)N)N	https://www.rcsb.org/structure/2W6Q
2W71	L23	293.16	Cc1cccc(C)c1Cn1cc(-c2ccnc(N)n2)nc1C	https://www.rcsb.org/structure/2W71
2W6M	OA1	200.08	Bc1cccc(C(=O)c2cnc(N)o2)c1	https://www.rcsb.org/structure/2W6M
2W6N	OA2	307.13	Nc1ncc(C(=O)N(Cc2ccccc2)Cc2ccccc2)o1	https://www.rcsb.org/structure/2W6N
2W6O	OA3	191.11	CC1(C)CC(=O)c2c(N)ncnc2C1	https://www.rcsb.org/structure/2W6O
2W70	L22	193.04	Nc1nccc(-c2csc(N)n2)n1	https://www.rcsb.org/structure/2W70
2W6Z	L21	217.23	C.C.C.C.CN1CN=C(N)c2[nH]cnc21.[HH]	https://www.rcsb.org/structure/2W6Z
**CDK-2**				
**PDB ID**	**Ligand Name**	**Ligand MW**	**Ligand-smiles**	**URL**
4FKR	45K	383.04	O=C1Nc2ccc3ncsc3c2C1/C=N/c1ccc2c(c1)CS(=O)(=O)C2	https://www.rcsb.org/structure/4FKR
4FKS	46K	422.11	C.CC(=O)NS(=O)(=O)c1ccc(N)cc1.Cc1c(NC=O)ccc2ncsc12	https://www.rcsb.org/structure/4FKS
2VTA	LZ1	118.05	c1ccc2[nH]ncc2c1	https://www.rcsb.org/structure/2VTA
1VYW	292	291.14	O=C(Cc1ccc2ccccc2c1)Nc1cc(C2CC2)[nH]n1	https://www.rcsb.org/structure/1VYW
2VU3	LZE	382.2	c1cc(c(c(c1)Cl)C(=O)Nc2c[nH]nc2C(=O)NC3CCNCC3)Cl	https://www.rcsb.org/structure/2VU3
4FKQ	42K	357.06	NS(=O)(=O)c1ccc(N/N=C2\C(=O)Nc3ccc4[nH]nnc4c32)cc1	https://www.rcsb.org/structure/4FKQ
4FKL	CK2	206.06	Cc1nc(C)c(-c2ccnc(N)n2)s1	https://www.rcsb.org/structure/4FKL
4FKG	4CK	271.1	O=C(O)c1ccc(C(=O)Nc2cc(C3CC3)[nH]n2)cc1	https://www.rcsb.org/structure/4FKG
4FKI	09K	325.1	O=C(Cc1ccc(OC(F)(F)F)cc1)Nc1cc(C2CC2)[nH]n1	https://www.rcsb.org/structure/4FKI
4FKW	62K	416.12	CC(C)COC(=O)c1ccc2c(c1)/C(=N/Nc1ccc(S(N)(=O)=O)cc1)C(=O)N2	https://www.rcsb.org/structure/4FKW

1VYZ	N5B	227.11	O=C(Nc1cc(C2CC2)[nH]n1)c1ccccc1	https://www.rcsb.org/structure/1VYZ

Ephrin-A2				
PDB ID	Ligand Name	Ligand MW	Ligand-smiles	URL
6FNF	DXH	505.18	Cc1ccc(C(=O)Nc2cccc(C(F)(F)F)c2)cc1NC1N=C(c2cccnc2)Nc2c1cnn2C	https://www.rcsb.org/structure/6FNF
5I9Z	627	474.5	CN1CCN(CC1)c2ccc(cc2)C(=O)Nc3c4c([nH]n3)CN(C4)C(=O)[C@@H](c5ccccc5)OC	https://www.rcsb.org/structure/5I9Z
6FNG	DWT	505.18	Cc1ccc(C(=O)Nc2cccc(C(F)(F)F)c2)cc1NC1=C2CN(C)N=C2NC(c2cccnc2)=N1	https://www.rcsb.org/structure/6FNG
5NJZ	8ZH	469.98	Cc1cccc(c1NC(=O)c2cnc(s2)Nc3cccc(c3)C(=O)NC4CCNCC4)Cl	https://www.rcsb.org/structure/5NJZ
5I9X	DB8	530.4	CN1CCN(CC1)CCCOc2cc3c(cc2OC)c(c(cn3)C#N)Nc4cc(c(cc4Cl)Cl)OC	https://www.rcsb.org/structure/5I9X
5NKG	8ZK	498	CCc1cc(cc(c1)Nc2ncc(s2)C(=O)Nc3c(cccc3Cl)C)C(=O)NC4CCNCC4	https://www.rcsb.org/structure/5NKG
5I9Y	1N1	453.19	Cc1cccc(c1NC(=O)c2cnc(s2)Nc3cc(nc(n3)C)N4CCN(CC4)CCO)Cl	https://www.rcsb.org/structure/5I9Y
5NKH	8ZQ	561	Cc1cccc(c1NC(=O)c2cnc(s2)Nc3cc(cc(c3)C(=O)NC4CCNCC4)c5ccccc5N)Cl	https://www.rcsb.org/structure/5NKH
5NKI	8ZW	472.12	Cc1ccccc1NC(=O)c1cnc(Nc2cc(N3CCOCC3)cc(S(C)(=O)=O)c2)s1	https://www.rcsb.org/structure/5NKI
5IA3	P17	374.12	CSc1cccc(Nc2ncc3cc(-c4ccccc4)c(=O)n(C)c3n2)c1	https://www.rcsb.org/structure/5IA3
5NKB	8ZT	479.2	Cc1ccccc1NC(=O)c1cnc(Nc2cc(N3CCOCC3)cc(N3CCOCC3)c2)s1	https://www.rcsb.org/structure/5NKB
5IA4	88Z	632.6	COc1cc2c(ccnc2cc1OCCCN3CCOCC3)Oc4ccc(cc4F)NC(=O)C5(CC5)C(=O)Nc6ccc(cc6)F	https://www.rcsb.org/structure/5IA4
5NK6	90W	450.17	Cc1cccc(c1NC(=O)c2cnc(s2)Nc3cccc(c3)C(=O)NC4CCC(CC4)O)Cl	https://www.rcsb.org/structure/5NK6
5NKC	90T	450.14	Cc1ccccc1NC(=O)c1cnc(Nc2cccc(C(=O)N3CC[C@H](C(=O)O)C3)c2)s1	https://www.rcsb.org/structure/5NKC
5IA5	GV0	633.6	CN1CCN(CC1)C2CCN(CC2)C(=O)Nc3cc(ccn3)Oc4ccc(c(c4)F)NC(=O)C5(CC5)C(=O)Nc6ccc(cc6)F	https://www.rcsb.org/structure/5IA5
5NK7	8ZZ	487.9	Cc1cccc(c1NC(=O)c2cnc(s2)Nc3cccc(c3)C(=O)N[C@@H]4CCNC[C@H]4F)Cl	https://www.rcsb.org/structure/5NK7
5NKD	91K	464.15	Cc1ccccc1NC(=O)c1cnc(Nc2cccc(C(=O)N3CC[C@H](CC(=O)O)C3)c2)s1	https://www.rcsb.org/structure/5NKD
5NK8	90Z	507.23	Cc1ccccc1NC(=O)c1cnc(Nc2cccc(C(=O)NC3CC(C)(C)N(O)C(C)(C)C3)c2)s1	https://www.rcsb.org/structure/5NK8
5NK2	8ZZ	487.9	Cc1cccc(c1NC(=O)c2cnc(s2)Nc3cccc(c3)C(=O)N[C@@H]4CCNC[C@H]4F)Cl	https://www.rcsb.org/structure/5NK2
5IA1	ZZL	442.12	O=C(O)c1ccc(Nc2ncc3c(n2)-c2ccccc2C(c2c(F)cccc2F)=NC3)cc1	https://www.rcsb.org/structure/5IA1
5NK3	92Q	455.9	Cc1cccc(c1NC(=O)c2cnc(s2)Nc3cccc(c3)C(=O)N[C@H]4CCNC4)Cl	https://www.rcsb.org/structure/5NK3
5IA2	L66	417.14	COc1ccccc1-n1c(-c2cc(O)ccc2C)cn2c3c(=O)[nH]c(=O)n(C)c3nc12	https://www.rcsb.org/structure/5IA2
5NK4	90E	505.9	Cc1cccc(c1NC(=O)c2cnc(s2)Nc3cccc(c3)C(=O)N[C@@H]4CCNCC4(F)F)Cl	https://www.rcsb.org/structure/5NK4
5NKA	91H	478.17	Cc1ccccc1NC(=O)c1cnc(Nc2cccc(C(=O)N[C@H]3CC[C@H](C(=O)O)CC3)c2)s1	https://www.rcsb.org/structure/5NKA

Hepatitis-C RNA polymerase ns5b				
PDB ID	Ligand Name	Ligand MW	Ligand-smiles	URL
3CJ4	SX5	344.19	Bc1ccc(NC(=O)CCC(=O)O)c(C(=O)N2C[C@H](C)C[C@H](C)C2)c1	https://www.rcsb.org/structure/3CJ4
3CJ5	SX6	413.25	Bc1ccc(NC(=O)CCC(=O)N2CCOCC2)c(C(=O)N2C[C@H](C)C[C@H](C)C2)c1	https://www.rcsb.org/structure/3CJ5

3CJ0	SX2	206.09	Bc1ccc(NC(=O)CCC(=O)O)nc1	https://www.rcsb.org/structure/3CJ0
3CIZ	SX1	149.06	Bc1ccc(N)c(C(=O)O)c1	https://www.rcsb.org/structure/3CIZ
3CJ2	SX3	244.17	Bc1ccc(N)c(C(=O)N2C[C@H](C)C[C@H](C)C2)c1	https://www.rcsb.org/structure/3CJ2
3CJ3	SX4	306.19	Bc1ccc(N)c(C(=O)N2CCC[C@@H]2Cc2ccccc2C)c1	https://www.rcsb.org/structure/3CJ3
HIV-1 protease				
PDB ID	Ligand Name	Ligand MW	Ligand-smiles	URL
2UXZ	HI1	642.36	Bc1ccc(CN(CCC[C@@](O)(Cc2ccccc2)C(=O)N[C@H]2c3ccccc3C[C@H]2O)NC(=O)[C@@H](NC(=O)OC)C(C)(C)C)cc1	https://www.rcsb.org/structure/2UXZ
1EC1	BEE	778.31	CNC(=O)[C@@H](NC(=O)[C@H](OCc1ccc(-c2ccsc2)cc1)[C@H](O)[C@@H](O)[C@@H](OCc1ccc(-c2ccsc2)cc1)C(=O)N[C@H](C(=O)NC)C(C)C)C(C)C	https://www.rcsb.org/structure/1EC1
2QNP	QN2	813.5	c1ccc(cc1)S(=O)(=O)N(Cc2ccc(cc2)I)[C@H]3CNC[C@@H]3N(Cc4ccc(cc4)I)S(=O)(=O)c5ccccc5	https://www.rcsb.org/structure/2QNP
1AJV	NMB	574.21	O=S1(=O)N(Cc2ccccc2)[C@H](COc2ccccc2)[C@H](O)[C@@H](O)[C@@H](COc2ccccc2)N1Cc1ccccc1	https://www.rcsb.org/structure/1AJV
1EC2	BEJ	768.38	CNC(=O)[C@@H](NC(=O)[C@H](OCc1ccc(-c2cccnc2)cc1)[C@H](O)[C@@H](O)[C@@H](OCc1ccc(-c2cccnc2)cc1)C(=O)N[C@H](C(=O)NC)C(C)C)C(C)C	https://www.rcsb.org/structure/1EC2
2QNQ	QN3	630.6	c1ccc(cc1)CN([C@H]2CNC[C@@H]2N(Cc3ccccc3)S(=O)(=O)c4ccccc4Cl)S(=O)(=O)c5ccccc5Cl	https://www.rcsb.org/structure/2QNQ
4I8Z	G08	575.23	CC(C)CN(C[C@@H](O)[C@H](Cc1ccccc1)NC(=O)O[C@H]1CO[C@H]2OCC[C@H]21)S(=O)(=O)c1ccc(C(N)=O)cc1	https://www.rcsb.org/structure/4I8Z
1HVJ	A78	778.45	CC(C)[C@H](NC(=O)N(C)Cc1ccccn1)C(=O)N[C@@H](Cc1ccccc1)C[C@H](O)[C@H](Cc1ccccc1)NC(=O)[C@@H](NC(=O)N(C)Cc1ccccn1)C(C)C	https://www.rcsb.org/structure/1HVJ
1D4H	BEH	610.27	O=C(NCc1ccccc1)[C@H](OCc1ccccc1)[C@H](O)[C@@H](O)[C@@H](OCc1ccccc1)C(=O)N[C@H]1c2ccccc2C[C@H]1O	https://www.rcsb.org/structure/1D4H
1HVK	A79	794.45	CC(C)[C@H](NC(=O)N(C)Cc1ccccn1)C(=O)N[C@@H](Cc1ccccc1)[C@H](O)[C@@H](O)[C@H](Cc1ccccc1)NC(=O)[C@@H](NC(=O)N(C)Cc1ccccn1)C(C)C	https://www.rcsb.org/structure/1HVK
2PWR	G4G	647.7	c1ccc(cc1)CN([C@H]2CNC[C@@H]2N(Cc3ccccc3)S(=O)(=O)c4ccc(cc4)C(=O)N)S(=O)(=O)c5ccc(cc5)C(=O)N	https://www.rcsb.org/structure/2PWR
1D4I	BEG	636.28	O=C(N[C@H]1c2ccccc2C[C@H]1O)[C@@H](C[C@@H](O)[C@@H](OCc1ccccc1)C(=O)N[C@H]1c2ccccc2C[C@H]1O)OCc1ccccc1	https://www.rcsb.org/structure/1D4I
1HVL	A76	794.45	CC(C)[C@H](NC(=O)N(C)Cc1ccccn1)C(=O)N[C@@H](Cc1ccccc1)[C@@H](O)[C@H](O)[C@H](Cc1ccccc1)NC(=O)[C@@H](NC(=O)N(C)Cc1ccccn1)C(C)C	https://www.rcsb.org/structure/1HVL
1D4J	MSC	628.26	O=C(NCc1ccccc1F)[C@H](OCc1ccccc1)[C@H](O)[C@@H](O)[C@@H](OCc1ccccc1)C(=O)N[C@H]1c2ccccc2C[C@H]1O	https://www.rcsb.org/structure/1D4J
2AQU	DR7	704.39	COC(=O)N[C@H](C(=O)N[C@@H](Cc1ccccc1)[C@@H](O)CN(Cc1ccc(-c2ccccn2)cc1)NC(=O)[C@@H](NC(=O)OC)C(C)(C)C)C(C)(C)C	https://www.rcsb.org/structure/2AQU

4I8W	G07	576.21	CC(C)CN(C[C@@H](O)[C@H](Cc1ccccc1)NC(=O)O[C@H]1CO[C@H]2OCC[C@H]21)S(=O)(=O)c1ccc(C(=O)O)cc1	https://www.rcsb.org/structure/4I8W
1HPX	KNI	667.25	CSC[C@H](NC(=O)COc1cccc2cnccc12)C(=O)N[C@@H](Cc1ccccc1)[C@H](O)C(=O)N1CSC[C@H]1C(=O)NC(C)(C)C	https://www.rcsb.org/structure/1HPX
7UPJ	INU	479.18	O=c1oc2c(c(O)c1[C@H](c1cccc(NS(=O)(=O)c3ccccc3)c1)C1CC1)CCCCCC2	https://www.rcsb.org/structure/7UPJ
2CEN	4AH	679.34	COC(=O)N[C@H](C(=O)NN(Cc1ccc(-c2cccnc2)cc1)C[C@@](O)(Cc1ccccc1)C(=O)N[C@H]1c2ccccc2C[C@H]1O)C(C)(C)C	https://www.rcsb.org/structure/2CEN
1G35	AHF	664.25	CO[C@H](O)c1ccc(CN2[C@H](COc3ccccc3)[C@H](O)[C@@H](O)[C@@H](COc3ccccc3)N(Cc3ccc(CO)cc3)S2(=O)=O)cc1	https://www.rcsb.org/structure/1G35
2BPV	1IN	621.8	C[C@H]1CC[C@H]([C@H]1NC(=O)[C@H](Cc2ccccc2)C[C@@H](C[N@@]3CCN(C[C@H]3C(=O)NC(C)(C)C)C(=O)CCc4cccnc4)O)O	https://www.rcsb.org/structure/2BPV
2PQZ	G0G	561.7	c1ccc(cc1)CN([C@H]2CNC[C@@H]2N(Cc3ccccc3)S(=O)(=O)c4ccccc4)S(=O)(=O)c5ccccc5	https://www.rcsb.org/structure/2PQZ
3BGB	LJG	525.7	CC(C)CN(CCNCCN(CC(C)C)S(=O)(=O)c1ccc(cc1)N)S(=O)(=O)c2ccc(cc2)N	https://www.rcsb.org/structure/3BGB
4HLA	017	547.24	CC(C)CN(C[C@@H](O)[C@H](Cc1ccccc1)NC(=O)O[C@H]1CO[C@H]2OCC[C@H]21)S(=O)(=O)c1ccc(N)cc1	https://www.rcsb.org/structure/4HLA
3BGC	LJH	593.7	c1ccc(cc1)CN(CCNCCN(Cc2ccccc2)S(=O)(=O)c3ccc(cc3)N)S(=O)(=O)c4ccc(cc4)N	https://www.rcsb.org/structure/3BGC
1OHR	1UN	567.7	Cc1c(cccc1O)C(=O)N[C@@H](CSc2ccccc2)[C@@H](C[N@@]3C[C@H]4CCCC[C@H]4C[C@H]3C(=O)NC(C)(C)C)O	https://www.rcsb.org/structure/1OHR
2PWC	G3G	727.3	C[C@H]1CC[C@H]([C@H]1NC(=O)[C@H](Cc2ccccc2)C[C@@H](C[N@@]3CCN(C[C@H]3C(=O)NC(C)(C)C)C(=O)c4cnc(c(n4)Cl)N5CCN(CC5)C)O)O	https://www.rcsb.org/structure/2PWC
2BPY	3IN	727.3	C[C@H]1CC[C@H]([C@H]1NC(=O)[C@H](Cc2ccccc2)C[C@@H](C[N@@]3CCN(C[C@H]3C(=O)NC(C)(C)C)C(=O)c4cnc(c(n4)Cl)N5CCN(CC5)C)O)O	https://www.rcsb.org/structure/2BPY
1G2K	NM1	631.24	CNC(=O)c1cccc(CN2[C@H](COc3ccccc3)[C@H](O)[C@@H](O)[C@@H](COc3ccccc3)N(Cc3ccccc3)S2(=O)=O)c1	https://www.rcsb.org/structure/1G2K
2CEJ	1AH	614.33	Bc1ccc(CN(C[C@@](O)(Cc2ccccc2)C(=O)N[C@H]2c3ccccc3C[C@H]2O)NC(=O)[C@@H](NC(=O)OC)C(C)(C)C)cc1	https://www.rcsb.org/structure/2CEJ
1HPV	478	505.22	CC(C)CN(C[C@@H](O)[C@H](Cc1ccccc1)NC(=O)O[C@H]1CCOC1)S(=O)(=O)c1ccc(N)cc1	https://www.rcsb.org/structure/1HPV
2UY0	HV1	656.34	Bc1ccc(CN(NC(=O)[C@@H](NC(=O)OC)C(C)(C)C)C(=O)CC[C@@](O)(Cc2ccccc2)C(=O)N[C@H]2c3ccccc3C[C@H]2O)cc1	https://www.rcsb.org/structure/2UY0
2QNN	QN1	783.76	c1cc(ccc1CN([C@H]2CNC[C@@H]2N(Cc3ccc(cc3)C(F)(F)F)S(=O)(=O)c4ccc(cc4)C(=O)N)S(=O)(=O)c5ccc(cc5)C(=O)N)C(F)(F)F	https://www.rcsb.org/structure/2QNN
1C70	L75	652.8	CC(C)(C)NC(=O)[C@@H]1C[N@@](CC[N@]1C[C@H](C[C@@H](Cc2ccccc2)C(=O)N[C@H]3c4ccccc4C[C@H]3O)O)Cc5cc6ccccc6o5	https://www.rcsb.org/structure/1C70
Heat shock protein HSP-90				
PDB ID	Ligand Name	Ligand MW	Ligand-smiles	URL
5J20	6FJ	424.12	CN(Cc1ccco1)C(=O)c1cc(-c2n[nH]c(=O)n2-c2ccccc2F)c(O)cc1O	https://www.rcsb.org/structure/5J20
5ODX	9RZ	493.23	CO[C@H]1CCCN(C(=O)c2n[nH]c3cc(O)c(C(=O)N(C)c4ccc(N5CCOCC5)cc4)cc23)C1	https://www.rcsb.org/structure/5ODX
5J82	6GV	422.11	CC(C)N(C)S(=O)(=O)c1cc(-c2n[nH]c(=O)n2-c2ccccc2F)c(O)cc1O	https://www.rcsb.org/structure/5J82

4NH8	2LC	399.2	Cc1cn(-c2cc(F)c(C(N)=O)c(N[C@H]3CCOC3)c2)c2c1C(=O)CC(C)(C)C2	https://www.rcsb.org/structure/4NH8
6EYA	C4K	415.15	Cc1cccc(Cc2n[nH]c3cc(O)c(C(=O)N(C)c4ccc5c(c4)OCO5)cc23)c1	https://www.rcsb.org/structure/6EYA
2UWD	2GG	354.12	CCNC(=O)c1noc(-c2ccc(O)cc2O)c1-c1ccc(OC)cc1	https://www.rcsb.org/structure/2UWD
6EYB	C3Z	366.15	Oc1cc2n[nH]c(Cc3ccccc3)c2cc1-c1ccnn1-c1ccccc1	https://www.rcsb.org/structure/6EYB
4EGK	RDC	340.19	C.C.CC[C@H]1O[C@@H]1C[C@@H](C)OC(=O)c1c(O)cc(O)cc1CC=O	https://www.rcsb.org/structure/4EGK
5J9X	6GC	400.15	CCCCN(C)C(=O)c1cc(-c2n[nH]c(=O)n2-c2ccccc2F)c(O)cc1O	https://www.rcsb.org/structure/5J9X
4CWF	H05	229.13	CCCC1=NC2C=CC=CC2=C2N=C(N)NN12	https://www.rcsb.org/structure/4CWF
5FND	IQ5	221.06	CC(=O)Nc1cc2nc(N)sc2cc1C	https://www.rcsb.org/structure/5FND
5J8M	6DL	299.09	Bc1cc(-c2n[nH]c(=O)n2-c2ccccc2F)c(O)cc1O	https://www.rcsb.org/structure/5J8M
4BQG	50Q	202.06	Oc1cc(O)cc(Oc2ccccc2)c1	https://www.rcsb.org/structure/4BQG
1YET	GDM	582.45	C.C.C.C.C.CC.CC[C@@H](O)[C@H](C[C@H](C)CC1=C(OC)C(=O)C=C(NC(C)=O)C1=O)OC.COCCOC(N)=O	https://www.rcsb.org/structure/1YET
5FNF	TQL	230.07	O/N=C(\c1cccnc1)c1ccc(O)cc1O	https://www.rcsb.org/structure/5FNF
6F1N	C8W	441.11	COc1ccc(-c2c(C#N)c(N)nc3sc(C(N)=O)c(N)c23)cc1OCCCC(=O)O	https://www.rcsb.org/structure/6F1N
3D0B	SNX	377.17	COCCNc1cc(-n2c3c(c4ccccc42)C(=O)CCC3)ccc1C(N)=O	https://www.rcsb.org/structure/3D0B
5J64	6G7	287.07	O=c1[nH]nc(-c2ccc(O)cc2O)n1-c1ccccc1F	https://www.rcsb.org/structure/5J64
5NYH	9EK	449.21	CN(C(=O)c1cc2c(C(=O)N3CCCC3)[nH]nc2cc1O)c1ccc(N2CCOCC2)cc1	https://www.rcsb.org/structure/5NYH
6FCJ	D4W	252.09	Oc1ccc(-c2ccnn2-c2ccccc2)c(O)c1	https://www.rcsb.org/structure/6FCJ
6ELP	BA8	357.15	Oc1cc(O)c(-c2ccnn2-c2ccccc2)cc1CCc1ccccn1	https://www.rcsb.org/structure/6ELP
1YC4	43P	270.11	CCc1cc(-c2n[nH]cc2-c2c[nH]cn2)c(O)cc1O	https://www.rcsb.org/structure/1YC4
5J27	6FF	422.11	CCCN(C)S(=O)(=O)c1cc(-c2n[nH]c(=O)n2-c2ccccc2F)c(O)cc1O	https://www.rcsb.org/structure/5J27
6HHR	G5E	303.05	Oc1ccc(-c2nnc(S)n2-c2ccccc2F)c(O)c1	https://www.rcsb.org/structure/6HHR
2YKI	YKI	444.17	O=C(N[C@@H]1c2ccccc2-c2c(-c3nc4c([nH]3)=CNCC=4)cccc21)c1ccnc2[nH]ccc12	https://www.rcsb.org/structure/2YKI
5J2X	6DL	299.09	Bc1cc(-c2n[nH]c(=O)n2-c2ccccc2F)c(O)cc1O	https://www.rcsb.org/structure/5J2X
4CWT	IK9	398.15	NC1=NN2C(=c3c(NCCO)cc(F)cc3=N[C@@H]2Cc2ccc3c(c2)OCO3)N1	https://www.rcsb.org/structure/4CWT
4FCQ	2N6	296.11	CSC1=NC(c2ccc(C)cc2C)c2c(C#N)c[nH]c2N1	https://www.rcsb.org/structure/4FCQ
1YC1	4BC	368.1	CCc1cc(-c2n[nH]c(C(=O)O)c2-c2ccc3c(c2)OCO3)c(O)cc1O	https://www.rcsb.org/structure/1YC1
4CWO	T62	309.12	NC1=NN2C(=c3ccccc3=N[C@@H]2Cc2cc(O)cc(O)c2)N1	https://www.rcsb.org/structure/4CWO
6ELO	BAW	312.13	CCc1cc(-c2n[nH]c(C)c2-c2ccccc2F)c(O)cc1O	https://www.rcsb.org/structure/6ELO
5J86	6GW	436.12	Cc1ccccc1-n1c(-c2cc(C(=O)N(C)Cc3cccs3)c(O)cc2O)n[nH]c1=O	https://www.rcsb.org/structure/5J86
5J6L	6GC	400.15	CCCCN(C)C(=O)c1cc(-c2n[nH]c(=O)n2-c2ccccc2F)c(O)cc1O	https://www.rcsb.org/structure/5J6L
6EY8	C4T	371.16	CN(Cc1ccccc1)C(=O)c1cc2c(Cc3ccccc3)n[nH]c2cc1O	https://www.rcsb.org/structure/6EY8

5J6M	6FJ	424.12	CN(Cc1ccco1)C(=O)c1cc(-c2n[nH]c(=O)n2-c2ccccc2F)c(O)cc1O	https://www.rcsb.org/structure/5J6M
6EY9	C4N	385.18	Cc1cccc(Cc2n[nH]c3cc(O)c(C(=O)N(C)Cc4ccccc4)cc23)c1	https://www.rcsb.org/structure/6EY9
4B7P	9UN	454.4	CN1CCC(CC1)NC(=O)c2cc(on2)c3c(cc(cc3Oc4ccc(cc4)[N+](=O)[O-])O)O	https://www.rcsb.org/structure/4B7P
5J6N	6FF	422.11	CCCN(C)S(=O)(=O)c1cc(-c2n[nH]c(=O)n2-c2ccccc2F)c(O)cc1O	https://www.rcsb.org/structure/5J6N
3FT5	MO8	181.07	Cc1nc(N)nc2c1CSCC2	https://www.rcsb.org/structure/3FT5
2W12	WR2	455.22	CNC(=O)[C@@H](NC(=O)[C@H](CC(C)C)[C@H](CNC(=O)c1nccs1)C(=O)NO)C(C)(C)C	https://www.rcsb.org/structure/2W12
2QFO	A51	199.16	C.C.COC(C)=O.Nc1ccccc1	https://www.rcsb.org/structure/2QFO
2QFO	A13	177.05	Cc1cc(C(F)(F)F)nc(N)n1	https://www.rcsb.org/structure/2QFO
2QG0	A94	399.19	C.C.COC(C)=O.Cc1cc(CNS(=O)(=O)c2cccc(N)c2)nc(N)n1	https://www.rcsb.org/structure/2QG0
2QG2	A91	325.15	Cc1cc(CCc2ccccc2CNn2ccoc2=O)nc(N)n1	https://www.rcsb.org/structure/2QG2
2QF6	A56	316.1	c1ccc2cc(c(cc2c1)c3nc(nc(n3)N)N)Br	https://www.rcsb.org/structure/2QF6
**JAK1-kinase**				
**PDB ID**	**Ligand Name**	**Ligand MW**	**Ligand-smiles**	**URL**
6N79	KES	370.1	O=C(Nc1cn[nH]c1-c1ccccc1OC(F)F)c1cnn2cccnc12	https://www.rcsb.org/structure/6N79
4EI4	0Q2	270.15	Cc1nc2cnc3[nH]ccc3c2n1[C@@H]1CCC[C@@H](O)C1	https://www.rcsb.org/structure/4EI4
4K77	1Q4	244.13	O=c1[nH]ccc2c(NC3CCCCC3)ncnc12	https://www.rcsb.org/structure/4K77
4E5W	0NT	380.4	CC(C)N1CCC[C@H]1C(=O)N2CCC(CC2)n3cnc4c3c5cc[nH]c5nc4	https://www.rcsb.org/structure/4E5W
4E4N	0NL	341.19	CC(C)(C)OC(=O)N[C@@H]1CC[C@@H](n2cnc3cnc4[nH]ccc4c32)C1	https://www.rcsb.org/structure/4E4N
4I5C	C5I	309.13	N#CCC(=O)N1CCC[C@@H](n2nnc3cnc4[nH]ccc4c32)C1	https://www.rcsb.org/structure/4I5C
4EHZ	LIG	255.3	Cc1nc2cnc3c(c2n1C4CCNCC4)cc[nH]3	https://www.rcsb.org/structure/4EHZ
4FK6	0UJ	359.14	CS(=O)(=O)NCc1nc2cnc3[nH]ccc3c2n1[C@@H]1C[C@H]2CC[C@@H]1C2	https://www.rcsb.org/structure/4FK6
4IVB	1J5	309.16	C[C@@H](O)c1nc2cnc3[nH]ccc3c2n1[C@H]1CC[C@H](C#N)CC1	https://www.rcsb.org/structure/4IVB
4IVC	1J6	323.17	C[C@@H](O)c1nc2cnc3[nH]ccc3c2n1[C@H]1CC[C@H](CC#N)CC1	https://www.rcsb.org/structure/4IVC
4IVD	15T	337.19	C[C@@H](O)c1nc2cnc3[nH]ccc3c2n1[C@H]1CC[C@H](CCC#N)CC1	https://www.rcsb.org/structure/4IVD
**JAK2- Kinases**				
**PDB ID**	**Ligand Name**	**Ligand MW**	**Ligand-smiles**	**URL**
4ZIM	4OK	399.16	NC(=O)c1cc(-c2ccccc2)cc2c1[nH]c1ccc(C(=O)N3CCOCC3)cc12	https://www.rcsb.org/structure/4ZIM
3E62	5B1	145.08	Bc1ccc2[nH]nc(N)c2c1	https://www.rcsb.org/structure/3E62
3E64	5B3	344.13	CC(C)(C)NS(=O)(=O)c1ccc(-c2ccc3[nH]nc(N)c3c2)cc1	https://www.rcsb.org/structure/3E64
**JAK3- Kinases**				
**PDB ID**	**Ligand Name**	**Ligand MW**	**Ligand-smiles**	**URL**

3PJC	PJC	455.3	c1ccc2c(c1)c(c[nH]2)C3=C(C(=O)NC3=O)c4c(cnc(n4)N5CCC(=O)CC5)C(F)(F)F	https://www.rcsb.org/structure/3PJC
7C3N	FHX	310.3	C[C@H]1CN([C@]12CCN(C2)c3c4cc[nH]c4ncn3)C(=O)CC#N	https://www.rcsb.org/structure/7C3N
6AAK	9T6	326.3	c1c[nH]c2c1c(c(cn2)C(=O)N)NC3[C@@H]4CC5C[C@H]3CC(C4)(C5)O	https://www.rcsb.org/structure/6AAK
3LXK	MI1	312.3	C[C@@H]1CCN(C[C@@H]1N(C)c2c3cc[nH]c3ncn2)C(=O)CC#N	https://www.rcsb.org/structure/3LXK
**Main Protease Protein M-pro**				
**PDB ID**	**Ligand Name**	**Ligand MW**	**Ligand-smiles**	**URL**
7DDC	H3F	463.4	Cc1cc(nc2c1c(c(cc2N[C@@H](C)CCCN)OC)Oc3cccc(c3)C(F)(F)F)OC	https://www.rcsb.org/structure/7DDC
5R81	RZJ	226.08	CN1CCCc2ccc(S(N)(=O)=O)cc21	https://www.rcsb.org/structure/5R81
7L0D	0EN	433.24	CC(C)(C)NC(=O)[C@@H](c1cccnc1)N(C(=O)c1ccco1)c1ccc(C(C)(C)C)cc1	https://www.rcsb.org/structure/7L0D
5RGK	U0V	244.1	O=C(NCCc1ccncc1)c1ccccc1F	https://www.rcsb.org/structure/5RGK
5REB	T0Y	197.2	c1cscc1CN2CCC(CC2)O	https://www.rcsb.org/structure/5REB
5R82	LIG	147.08	CCNc1ccc(C#N)cn1	https://www.rcsb.org/structure/5R82
7JU7	G65	498.6	Cc1ccc(cc1Nc2nc(cs2)c3cccnc3)NC(=O)c4ccc(cc4)CN5CCN(CC5)C	https://www.rcsb.org/structure/7JU7
5R83	K0G	213.09	O=C(Nc1ccccc1)Nc1cccnc1	https://www.rcsb.org/structure/5R83
5R84	GWS	218.14	O=C(CC1CCCCC1)Nc1cccnc1	https://www.rcsb.org/structure/5R84
7L5D	XNJ	484.6	Cc1ccc(cc1Nc2nc(cs2)c3cccnc3)NC(=O)c4ccc(cc4)CN5CCNCC5	https://www.rcsb.org/structure/7L5D
7RNK	5ZT	330.13	O=C1CC(C(=O)N2CCN(c3ccc(CO)cc3)CC2)=NC(=O)N1	https://www.rcsb.org/structure/7RNK
5RF6	NTG	203.11	N#Cc1ccc(N2CCCOCC2)cn1	https://www.rcsb.org/structure/5RF6
5RGZ	UH1	237.09	N#Cc1cccc(CC(=O)Nc2cccnc2)c1	https://www.rcsb.org/structure/5RGZ
5RGH	U0M	215.09	Cc1nnc(CN2CCC[C@@H](F)C2)s1	https://www.rcsb.org/structure/5RGH
5RF7	T67	258.3	CN1CCN(CC1)C(=O)Cc2c[nH]c3c2cccn3	https://www.rcsb.org/structure/5RF7
7RMZ	LIG	368.11	O=C(c1cc(=O)[nH]c(=O)[nH]1)N1CCN(c2ccc(C(F)(F)F)cc2)CC1	https://www.rcsb.org/structure/7RMZ
5RGI	U0P	209.12	Cc1cc(CN(C)C(=O)NC2CC2)no1	https://www.rcsb.org/structure/5RGI
5RFE	JGG	245.12	N#Cc1ccc(CNC(=O)N2CCOCC2)cc1	https://www.rcsb.org/structure/5RFE
5R80	RZG	215.03	COC(=O)c1ccc(S(N)(=O)=O)cc1	https://www.rcsb.org/structure/5R80
5RE4	SZY	150.08	CC(=O)Nc1cnccc1C	https://www.rcsb.org/structure/5RE4
5RF2	HVB	78.12	C.C.NCN	https://www.rcsb.org/structure/5RF2
7S3S	860	262.11	c1ccc2c(c1)cncc2NC(=O)Cc3cccc(c3)Cl	https://www.rcsb.org/structure/7S3S
7M8Y	YTM	478.16	O=C1N=C[C@@H](c2cc(-c3cccc(OCCc4ccccc4)c3)c(=O)n(-c3cccnc3)c2)C(=O)N1	https://www.rcsb.org/structure/7M8Y
7AQE	RV5	319.11	O=C(NCC(=O)N1CCCCC1)Nc1ccc2nnsc2c1	https://www.rcsb.org/structure/7AQE
7RNH	5ZW	300.12	O=C(c1cc(=O)[nH]c(=O)[nH]1)N1CCN(c2ccccc2)CC1	https://www.rcsb.org/structure/7RNH
5RF3	T5V	95.05	Nc1cncnc1	https://www.rcsb.org/structure/5RF3

5RGW	UGM	238.09	N#Cc1cncc(CC(=O)Nc2cccnc2)c1	https://www.rcsb.org/structure/5RGW
7KX5	X7V	501.21	C[C@H](NC(=O)[C@@H](c1cccnc1)N(C(=O)c1ccco1)c1ccc(-c2ccccc2)cc1)c1ccccc1	https://www.rcsb.org/structure/7KX5
5R7Y	JFM	199.07	CS(=O)(=O)NCCc1ccccc1	https://www.rcsb.org/structure/5R7Y
7RM8	5Z7	312.14	Bc1ccc(N2CCN(C(=O)c3cc(=O)[nH]c(=O)[nH]3)CC2)cc1	https://www.rcsb.org/structure/7RM8
7RME	5Z3	368.11	O=C(c1cc(=O)[nH]c(=O)[nH]1)N1CCN(c2cccc(C(F)(F)F)c2)CC1	https://www.rcsb.org/structure/7RME
7RLS	5YN	300.12	O=C(c1cc(=O)[nH]c(=O)[nH]1)N1CCN(c2ccccc2)CC1	https://www.rcsb.org/structure/7RLS
7AP6	RQN	293.09	CCc1cc(O)c(Oc2ccc(C(N)=O)cc2F)cc1F	https://www.rcsb.org/structure/7AP6
5R7Z	HWH	220.1	CC(=O)NCCc1c[nH]c2ccc(F)cc12	https://www.rcsb.org/structure/5R7Z
7L10	XEY	349.12	N#Cc1ccccc1-c1cc(-c2ccccc2)c(=O)n(-c2cccnc2)c1	https://www.rcsb.org/structure/7L10
7RM2	5YJ	314.14	Cc1ccc(N2CCN(C(=O)c3cc(=O)[nH]c(=O)[nH]3)CC2)cc1	https://www.rcsb.org/structure/7RM2
5RH8	UHM	273.06	N#CCOc1ccccc1C(=O)NCc1cnsc1	https://www.rcsb.org/structure/5RH8
7S4B	87H	294.12	C[C@@H](C(=O)Nc1cncc2ccccc12)c1cccc(F)c1	https://www.rcsb.org/structure/7S4B
6W63	X77	459.26	CC(C)(C)c1ccc(N(C(=O)c2c[nH]cn2)[C@@H](C(=O)NC2CCCCC2)c2cccnc2)cc1	https://www.rcsb.org/structure/6W63
7L12	XF4	464.15	O=c1[nH]cc(-c2cc(-c3cccc(OCc4ccccc4)c3)c(=O)n(-c3cccnc3)c2)c(=O)[nH]1	https://www.rcsb.org/structure/7L12
5RF1	T5G	169.04	Bc1ccc(S(N)(=O)=O)cc1	https://www.rcsb.org/structure/5RF1
7S3K	Z26	292.12	COc1ccccc1CC(=O)Nc1cncc2ccccc12	https://www.rcsb.org/structure/7S3K
7LTJ	YD1	300.12	O=C(c1cc(=O)[nH]c(=O)[nH]1)N1CCN(c2ccccc2)CC1	https://www.rcsb.org/structure/7LTJ
7LMD	Y6A	414.13	O=C(Cn1nnc2ccccc21)N(Cc1ccsc1)c1ccc(-c2cn[nH]c2)cc1	https://www.rcsb.org/structure/7LMD
7RN4	H69	299.13	O=C(c1cc(=O)[nH]c(=O)[nH]1)N1CCC(c2ccccc2)CC1	https://www.rcsb.org/structure/7RN4
5RG1	T9J	276.16	BCCCNC(=O)[C@H](Cc1ccc(O)cc1)NC(C)=O	https://www.rcsb.org/structure/5RG1
5REZ	T54	195.07	NC(=O)[C@H]1CCC[C@H]1c1ccsc1	https://www.rcsb.org/structure/5REZ
7N8C	YD1	300.12	c1cc(c(cc1N2CCN(CC2)C(=O)C3=CC(=O)NC(=O)N3)Cl)Cl	https://www.rcsb.org/structure/7N8C
5REH	AWP	247.17	O=C(NCCc1ccncc1)NC1CCCCC1	https://www.rcsb.org/structure/5REH
p38 Kinase				
PDB ID	Ligand Name	Ligand MW	Ligand-smiles	URL
1WBT	WBT	444.2	O=C(Nc1ccc2ccn(CCc3ccncc3)c2c1)c1cc(F)cc(N2CCOCC2)c1	https://www.rcsb.org/structure/1WBT
1W7H	3IP	200.09	Nc1ncccc1OCc1ccccc1	https://www.rcsb.org/structure/1W7H
1WBV	LI3	339.14	O=C(Nc1ccc2[nH]ccc2c1)c1cc(F)cc(N2CCOCC2)c1	https://www.rcsb.org/structure/1WBV
1WBW	LI4	250.11	Nc1ncccc1OCc1cccc2ccccc12	https://www.rcsb.org/structure/1WBW
1WBS	LI2	444.2	O=C(Nc1ccc2[nH]cc(CCc3ccncc3)c2c1)c1cc(F)cc(N2CCOCC2)c1	https://www.rcsb.org/structure/1WBS
1WBN	L09	379.2	Cc1ccc(NC(=O)Nc2cc(C(C)(C)C)[nH]n2)cc1COc1cccnc1	https://www.rcsb.org/structure/1WBN
1W82	L10	348.2	Cc1ccc(NC(=O)Nc2cc(C(C)(C)C)nn2-c2ccccc2)cc1	https://www.rcsb.org/structure/1W82

1WBO	2CH	108.06	Cc1ccccc1O	https://www.rcsb.org/structure/1WBO
1W83	L11	435.29	C.C.C[C@@H](CCF)N1CCOCC1.Cc1ccc(NC=O)cc1COc1cccnc1	https://www.rcsb.org/structure/1W83
1W84	L12	222.12	c1ccc2c(CCc3ccncc3)c[nH]c2c1	https://www.rcsb.org/structure/1W84
PI3K-gamma				
PDB ID	Ligand Name	Ligand MW	Ligand-smiles	URL
4PS3	2WH	438.18	CCCN1C=C(CCNC(=O)Nc2nc3ccc(-c4cncc(OC)c4)cc3s2)NC1	https://www.rcsb.org/structure/4PS3
4G11	0W7	314.14	O=C(Cc1nc(N2CCOCC2)cc(=O)[nH]1)Nc1ccccc1	https://www.rcsb.org/structure/4G11
3QK0	QK0	442.06	CC(=O)Nc1nc2ccc(-c3cncc(NS(=O)(=O)c4ccc(F)cc4)c3)cc2s1	https://www.rcsb.org/structure/3QK0
3DBS	GD9	513.16	CS(=O)(=O)N1CCN(Cc2cc3nc(-c4cccc5[nH]ncc45)nc(N4CCOCC4)c3s2)CC1	https://www.rcsb.org/structure/3DBS
2A5U	QYT	259.04	O=C1NC(=O)C(Cc2ccc3nccnc3c2)S1	https://www.rcsb.org/structure/2A5U
4F1S	F1S	490.9	Cc1nc(nc(n1)N)c2cc(cnc2Nc3cc(c(nc3)Cl)NS(=O)(=O)C)C4CCOCC4	https://www.rcsb.org/structure/4F1S
3ZW3	ZW3	304.06	CC(=O)Nc1cn2cc(CC3SC(=O)NC3=O)ccc2n1	https://www.rcsb.org/structure/3ZW3
3R7Q	FAV	321.08	CN(C(=O)c1cc2c(s1)-c1ccccc1OC2)c1ccccc1	https://www.rcsb.org/structure/3R7Q
5T23	74F	408.21	C[C@@H](C1N=C(c2cnc(N)c(-n3nnc4ccccc43)n2)NN1C)N1CCOCC1	https://www.rcsb.org/structure/5T23
3R7R	FAZ	392.12	CC(=O)Nc1ccc2c(c1)OCCc1cc(C(=O)N(C)c3ccccc3)sc1-2	https://www.rcsb.org/structure/3R7R
6C1S	EFV	376.13	COc1cc(-c2ccc3c(n2)CN(c2cnn(CC#N)c2)C3=O)cnc1OC	https://www.rcsb.org/structure/6C1S
4WWP	3VE	381.17	Cc1ncccc1-c1nc2ccccc2cc1[C@H](C)Nc1ncnc2[nH]cnc12	https://www.rcsb.org/structure/4WWP
4URK	A82	410.2	CC1=CC([C@@H](C)Nc2ccccc2C(=O)O)=C2NC(N3CCOCC3)=CC(=O)N2C1	https://www.rcsb.org/structure/4URK
3NZS	NZS	388.13	COc1cccc(Nc2nc(N3CCS(=O)(=O)CC3)nc3c2cnn3C)c1	https://www.rcsb.org/structure/3NZS
3NZU	NZU	387.15	Cn1ncc2c1NC(c1cccc3[nH]ncc13)=NC2NCCCS(C)(=O)=O	https://www.rcsb.org/structure/3NZU
3S2A	2NQ	464.13	O=S(=O)(Nc1cncc(-c2ccc3nccc(N4CCOCC4)c3c2)c1)c1ccc(F)cc1	https://www.rcsb.org/structure/3S2A
2CHZ	093	355.07	CC(=O)Nc1nc(C)c(-c2cccc(S(=O)(=O)NCCO)c2)s1	https://www.rcsb.org/structure/2CHZ
4GB9	0WR	533.6	CC(C)(C1CCN(CC1)Cc2nc3c(n2C)nc(nc3N4CCOCC4)n5c6ccccc6nc5N(C)C)O	https://www.rcsb.org/structure/4GB9
4FJZ	4FJ	513.25	Cc1c(-c2ccccn2)nc2cc(F)ccc2c1N1CC2(CCOCC2)C2NC=C(N3CCOCC3)C=C21	https://www.rcsb.org/structure/4FJZ
4WWN	3VC	387.16	C[C@H](NC1N=CNc2[nH]cnc21)c1cc2ccc(F)cc2nc1-c1ccccn1	https://www.rcsb.org/structure/4WWN
6AUD	BWY	482.6	CC(C)n1c(ncn1)c2cn3c(n2)-c4cc(ccc4OCC3)S(=O)C5CCN(CC5)C(C)(C)C	https://www.rcsb.org/structure/6AUD
4HVB	19P	446.21	Cc1ccc(-c2ccc3ncc4c(c3n2)n(C2CCN(C(=O)[C@H](C)O)CC2)c(=O)n4C)cn1	https://www.rcsb.org/structure/4HVB
3L08	ZIG	505.1	COc1ncc(-c2ccc3nccc(-c4ccnnc4)c3c2)cc1NS(=O)(=O)c1ccc(F)cc1F	https://www.rcsb.org/structure/3L08
3PRZ	3RZ	270.12	CC1=NC(N)c2cccc(C(=O)Nc3cc[nH]n3)c2N1	https://www.rcsb.org/structure/3PRZ

3L17	JZY	506.19	Cc1nc(N)ncc1C1=N[C@@H](N2CCOCC2)c2sc(CN3CCN(S(C)(=O)=O)CC3)cc2N1	https://www.rcsb.org/structure/3L17
2CHX	090	349.12	COc1ccc2c(nc(NC(=O)c3cccnc3)n3ccnc23)c1OC	https://www.rcsb.org/structure/2CHX
4DK5	0KO	485.5	Cc1nc(nc(n1)N)c2cc(cnc2Nc3ccc(nc3)OC)CN4CCN(CC4)S(=O)(=O)C	https://www.rcsb.org/structure/4DK5
3OAW	OAW	285.13	Cc1nc(N)nc2c1nc(-c1cn[nH]c1)c(=O)n2C(C)C	https://www.rcsb.org/structure/3OAW
3DPD	41A	266.11	CC1(C)CC(=O)c2sc(N3CCOCC3)nc2C1	https://www.rcsb.org/structure/3DPD
5EDS	5MT	451.12	C[C@H](Nc1ncnc(N)c1C#N)c1nc2ccc(F)cc2n1-c1cccc(S(C)(=O)=O)c1	https://www.rcsb.org/structure/5EDS
5JHA	6K7	424.18	CC1(CO)CN(c2nc(-c3cnc(N)cc3C(F)(F)F)cc(N3CCOCC3)n2)C1	https://www.rcsb.org/structure/5JHA
4PS7	2WJ	269.06	CC(=O)Nc1nc2ccc(-c3cccnc3)cc2s1	https://www.rcsb.org/structure/4PS7
4FLH	14K	517.5	Cc1nc(nc(n1)N)c2cc(cnc2Nc3cc(c(nc3)OC)F)[C@@H](C)N4CCN(CC4)S(=O)(=O)C	https://www.rcsb.org/structure/4FLH
3L13	JZW	503.17	CS(=O)(=O)N1CCN(Cc2cc3nc(-c4cccc(CO)c4)nc(N4CCOCC4)c3s2)CC1	https://www.rcsb.org/structure/3L13
4PS8	2WK	329.08	COc1cc(-c2ccc3nc(NC(C)=O)sc3c2)cnc1OC	https://www.rcsb.org/structure/4PS8
3PS6	3PS	311.14	COc1ccc(NC(=O)c2cccc3c2NC(C)=NC3N)cn1	https://www.rcsb.org/structure/3PS6
3L54	LXX	323.12	c1cc(-c2ccnc3ccc(-c4cnc5[nH]ncc5c4)cc23)ccn1	https://www.rcsb.org/structure/3L54
5OQ4	A3W	411.3	c1c(c(cnc1N)c2nc(nc(n2)N3CCOCC3)N4CCOCC4)C(F)(F)F	https://www.rcsb.org/structure/5OQ4
**Protein Kinase B**				
**PDB ID**	**Ligand Name**	**Ligand MW**	**Ligand-smiles**	**URL**
2UW6	GVO	297.7	c1cc(ccc1c2c[nH]nc2)[C@H](CN)c3ccc(cc3)Cl	https://www.rcsb.org/structure/2UW6
2UW7	GVP	337.8	c1cc(ccc1c2c[nH]nc2)C3(CCNCC3)c4ccc(cc4)Cl	https://www.rcsb.org/structure/2UW7
2VO6	M05	342.8	c1cc(ccc1CC2(CCN(CC2)c3c4cc[nH]c4ncn3)[NH3+])Cl	https://www.rcsb.org/structure/2VO6
2UW8	GVQ	231.7	c1ccc(cc1)[C@@H](CN)c2ccc(cc2)Cl	https://www.rcsb.org/structure/2UW8
2VO7	M05	342.84	c1cc(ccc1CC2(CCN(CC2)c3c4cc[nH]c4ncn3)[NH3+])Cl	https://www.rcsb.org/structure/2VO7
2VO0	M03	341.8	c1cc(ccc1C2(CCN(CC2)c3c4cc[nH]c4ncn3)CN)Cl	https://www.rcsb.org/structure/2VO0
2UW3	GVG	158.08	Cc1[nH]ncc1-c1ccccc1	https://www.rcsb.org/structure/2UW3
2UW4	L15	201.2	Cc1c(c[nH]n1)c2ccc(cc2)CCN	https://www.rcsb.org/structure/2UW4
2VO3	M04	355.8	c1cc(ccc1CC2(CCN(CC2)c3c4cc[nH]c4ncn3)CN)Cl	https://www.rcsb.org/structure/2VO3
2UW0	GVK	389.8	c1cc(ccc1c2c3c([nH]cn3)ncn2)C4(CCNCC4)c5ccc(cc5)Cl	https://www.rcsb.org/structure/2UW0
2UVX	GVH	118.05	c1cnc2[nH]ccc2c1	https://www.rcsb.org/structure/2UVX
2VNW	M01	232.2	c1[nH]c2c(n1)c(ncn2)N3CCC(CC3)CN	https://www.rcsb.org/structure/2VNW
2UVY	GVI	239.2	CNCc1ccc(cc1)c2c3c([nH]cn3)ncn2	https://www.rcsb.org/structure/2UVY
2UVZ	GVJ	301.34	c1ccc(cc1)[C@@H](c2ccc(cc2)c3c4c([nH]cn4)ncn3)N	https://www.rcsb.org/structure/2UVZ
2VNY	M02	218.2	c1[nH]c2c(n1)c(ncn2)N3CCC(CC3)N	https://www.rcsb.org/structure/2VNY
2UW9	GVP	337.8	c1cc(ccc1c2c[nH]nc2)C3(CCNCC3)c4ccc(cc4)Cl	https://www.rcsb.org/structure/2UW9
2UW5	GVN	297.7	c1cc(ccc1c2c[nH]nc2)[C@@H](CN)c3ccc(cc3)Cl	https://www.rcsb.org/structure/2UW5

Papain Protease Protein PL-pro				
PDB ID	Ligand Name	Ligand MW	Ligand-smiles	URL
7TZJ	S88	390.4	C[C@H](c1cccc2c1cccc2)N3CCC(CC3)C(=O)NCc4cccc(c4)F	https://www.rcsb.org/structure/7TZJ
7RZC	JWX	314.4	C[C@H](c1cccc2c1cccc2)N(C)Cc3c[nH]c4c3cccc4	https://www.rcsb.org/structure/7RZC
7LOS	Y97	490.6	Cc1ccc(cc1C(=O)N[C@H](C)c2cccc(c2)c3ccc(s3)CN[C@@H]4CCOC4)NC5CNC5	https://www.rcsb.org/structure/7LOS
7LLF	Y54	504.6	Cc1ccc(cc1C(=O)N[C@H](C)c2cccc(c2)c3ccc(s3)CN[C@@H]4CC[C@@H](C4)O)NC5CNC5	https://www.rcsb.org/structure/7LLF
7SDR	JW9	291.3	C[C@H](c1cccc2c1cccc2)N(C)Cc3ccc(cc3)O	https://www.rcsb.org/structure/7SDR
7LBS	XR8	474.6	Cc1ccc(cc1C(=O)N[C@H](C)c2cccc(c2)c3ccc(s3)CN4CCCC4)NC5CNC5	https://www.rcsb.org/structure/7LBS
7JIT	Y95	390.17	Cc1ccc(NC(=O)NC(N)=O)cc1C(=O)N[C@H](C)c1cccc2ccccc12	https://www.rcsb.org/structure/7JIT
7JIV	VBY	364.22	C.C.Cc1ccc(NC=O)cc1C(=O)N[C@H](C)c1cccc2ccccc12	https://www.rcsb.org/structure/7JIV
7JRN	TTT	304.16	Cc1ccc(N)cc1C(=O)N[C@H](C)c1cccc2ccccc12	https://www.rcsb.org/structure/7JRN

PPARgamma				
PDB ID	Ligand Name	Ligand MW	Ligand-smiles	URL
3ET3	ET1	389.09	COc1ccc(S(=O)(=O)n2cc(CCC(=O)O)c3cc(OC)ccc32)cc1	https://www.rcsb.org/structure/3ET3
3ET0	ET0	219.09	COc1ccc2[nH]cc(CCC(=O)O)c2c1	https://www.rcsb.org/structure/3ET0

BRD1				
PDB ID	Ligand Name	Ligand MW	Ligand-smiles	URL
5POU	8U7	247.3	CNC(=O)NC1CCN(CC1)Cc2ccccc2	https://www.rcsb.org/structure/5POU
5PO6	8SS	171.1	Bc1ccc(-c2c[nH]nc2N)cc1	https://www.rcsb.org/structure/5PO6
5POC	8TG	217.08	Bc1cc2c(ccc(=O)n2C)cc1[N+](=O)O	https://www.rcsb.org/structure/5POC
5PO0	8S7	166.07	Cc1cc(NC(=O)C2CC2)on1	https://www.rcsb.org/structure/5PO0
5POW	8UA	150.08	CC(=O)NCc1ccccn1	https://www.rcsb.org/structure/5POW
3RCW	MB3	99.07	CN1CCCC1=O	https://www.rcsb.org/structure/3RCW
5POQ	8TY	206.07	CC(=O)N1CCN(S(C)(=O)=O)CC1	https://www.rcsb.org/structure/5POQ
5POR	PW3	142.07	CC(=O)N1CCNC(=O)C1	https://www.rcsb.org/structure/5POR
5PP0	8UD	151.07	CNC(=O)c1cccnc1N	https://www.rcsb.org/structure/5PP0
5POS	8U1	179.09	COc1ccc(CNC(C)=O)cc1	https://www.rcsb.org/structure/5POS
5PON	8RA	205.12	CC(=O)N1CCN(c2ccccn2)CC1	https://www.rcsb.org/structure/5PON
5POO	8TS	230.12	CC(=O)N1CCN(c2ccc(C#N)cn2)CC1	https://www.rcsb.org/structure/5POO
5POH	8TP	178.11	CC(=O)NCCNc1ccccc1	https://www.rcsb.org/structure/5POH
5POI	8RY	137.06	CNC(=O)c1ncccn1	https://www.rcsb.org/structure/5POI

5POJ	8RV	232.16	CC(=O)NC1CCN(Cc2ccccc2)CC1	https://www.rcsb.org/structure/5POJ
5PO7	8SV	237.1	CNc1cc2c(cc1[N+](=O)O)n(C)c(=O)n2C	https://www.rcsb.org/structure/5PO7
5PO8	8SY	176.09	CN1C(=O)CCc2cc(N)ccc21	https://www.rcsb.org/structure/5PO8
5POE	8T1	174.08	Cn1c(=O)ccc2cc(N)ccc21	https://www.rcsb.org/structure/5POE
5AMF	TWL	194.11	CCOC(=O)[C@@H]1CCc2[nH]ncc2C1	https://www.rcsb.org/structure/5AMF
5POX	8UG	194.11	CCOC(=O)[C@H]1CCc2n[nH]cc2C1	https://www.rcsb.org/structure/5POX
5PO9	8T7	241.08	Cn1nc(C(F)(F)F)c(-c2ccccc2)c1N	https://www.rcsb.org/structure/5PO9
5POT	8U4	163.1	CC(=O)NCc1cccc(C)c1	https://www.rcsb.org/structure/5POT
5POB	8TD	177.09	Cn1c(=O)n(C)c2cc(N)ccc21	https://www.rcsb.org/structure/5POB

BRD2

PDB ID	Ligand Name	Ligand MW	Ligand-smiles	URL
3ONI	JQ1	436.19	Cc1ccc(C2=N[C@@H](CC(=O)OC(C)(C)C)c3nnc(C)n3-c3sc(C)c(C)c32)cc1	https://www.rcsb.org/structure/3ONI
5BT5	2LO	488.28	COc1ccc(CCc2nc3cc(-c4c(C)noc4C)ccc3n2C[C@H](C)N2CCOCC2)cc1C	https://www.rcsb.org/structure/5BT5
5EK9	5P4	356.17	CC(=O)N1c2ccc(-c3ccco3)cc2[C@H](NC(=O)OC(C)C)C[C@@H]1C	https://www.rcsb.org/structure/5EK9
5UEW	87D	400.11	COc1cc(=O)n(C)cc1-c1cc(NS(C)(=O)=O)ccc1Oc1ccccc1	https://www.rcsb.org/structure/5UEW
6I81	H7E	613.1	Cc1nnc2n1-c3ccc(cc3C(=N[C@H]2CC(=O)Nc4ccc(cc4)N5CCC(CC5)[N+](C)(C)C)c6ccc(cc6)Cl)OC	https://www.rcsb.org/structure/6I81
5XHK	2ME	60.06	CCOC	https://www.rcsb.org/structure/5XHK
5XHK	LDR	195.07	O=c1[nH]c2ccccc2c2ccccc12	https://www.rcsb.org/structure/5XHK
5U6V	7WY	382.19	Cc1ccc(N2CCn3nnc(C)c3-c3ccc(-c4ccc(N)nc4)cc32)cc1	https://www.rcsb.org/structure/5U6V
7NQJ	UME	335.17	CCNC(=O)c1cc(-c2cn(C)nn2)nc([C@@H](C)c2ccccc2)c1	https://www.rcsb.org/structure/7NQJ
6DBC	G3J	360.2	CC(=O)N1c2ccc(-c3cc[nH]n3)cc2[C@H](Nc2ccccc2C)C[C@@H]1C	https://www.rcsb.org/structure/6DBC
5DW1	5GD	423.5	Cc1cc(cc(c1OCCN2CCCC2)C)C3=Nc4cc(cc(c4C(=O)N3)OC)OC	https://www.rcsb.org/structure/5DW1
6VIY	QYY	507.18	CCNC(=O)c1cc2c(-c3cc(S(=O)(=O)CC)ccc3Oc3c(C)cccc3C)cn(C)c(=O)c2[nH]1	https://www.rcsb.org/structure/6VIY
6FFE	D7Q	350.16	CC(=O)N1c2ccccc2N(Cc2ccccc2C(=O)O)C[C@@H]1C1CC1	https://www.rcsb.org/structure/6FFE
6FFF	D7H	457.21	CC(=O)N1c2ccc(-c3cnc(C(N)=O)nc3)cc2N(Cc2ccccc2CO)C[C@@H]1C1CC1	https://www.rcsb.org/structure/6FFF
5N2L	8J2	423.22	CC(=O)Nc1cccc(-c2ccc3c(c2)[C@H](NC(=O)OC(C)C)C[C@H](C)N3C(C)=O)c1	https://www.rcsb.org/structure/5N2L
6E6J	HWV	491.22	CCNC(=O)c1cc2c(-c3cc(C(C)(C)O)ccc3Oc3c(C)cc(F)cc3C)cn(C)c(=O)c2[nH]1	https://www.rcsb.org/structure/6E6J
7NQI	UM8	333.16	Cn1cc(-c2cc(C(=O)NC3CC3)cc(Cc3ccccc3)n2)nn1	https://www.rcsb.org/structure/7NQI
4J1P	1K0	370.15	COc1cc(OC)c2c(=O)[nH]c(-c3cc(C)c(OCCO)c(C)c3)nc2c1	https://www.rcsb.org/structure/4J1P

5IG6	6B3	434.13	O=C(O)c1ccccc1-c1ccccc1C(=O)Nc1ccc2c(c1)[nH]c(=O)c1ccccc12	https://www.rcsb.org/structure/5IG6
6K04	CQF	384.21	Cc1ccc(N[C@@H]2CCc3nnc(C)n3-c3ccc(-c4cnn(C)c4)cc32)cc1	https://www.rcsb.org/structure/6K04
6DDJ	G7V	371.17	CC(=O)N1c2ccc(-c3cc[nH]n3)cc2[C@H](Nc2ccc(C#N)cc2)C[C@@H]1C	https://www.rcsb.org/structure/6DDJ
6MOA	JW4	394.18	Cc1ccc2ncccc2c1-c1cc(-c2c(C)noc2C)cc2[nH]c(C3CC3)nc12	https://www.rcsb.org/structure/6MOA
7NQ8	UM5	337.15	CCNC(=O)c1ccc(OCc2ccccn2)c(-c2cn(C)nn2)c1	https://www.rcsb.org/structure/7NQ8
4UYG	73B	414.19	CC(=O)N1c2ccc(-c3ccc(C(=O)O)cc3)cc2[C@H](Nc2ccc(C)cc2)C[C@@H]1C	https://www.rcsb.org/structure/4UYG
4MR5	1K0	326.13	COc1cc(OC)c2c(=O)[nH]c(-c3cc(C)c(O)c(C)c3)nc2c1	https://www.rcsb.org/structure/4MR5

BRD4

PDB ID	Ligand Name	Ligand MW	Ligand-smiles	URL
6CZV	FOY	280.12	Cc1noc(C)c1-c1ccc(=O)n(Cc2ccccc2)c1	https://www.rcsb.org/structure/6CZV
4XYA	43S	263.1	Bc1cc(-c2nc(N)nc3[nH]cnc23)c2occc2c1	https://www.rcsb.org/structure/4XYA
3U5L	08K	303.15	Cc1ccc2c(c1)C(c1ccccc1)=NN(C)c1nnc(C)n1-2	https://www.rcsb.org/structure/3U5L
4C67	L5S	280.08	Cc1nnc2n1-c1sccc1C(c1ccccc1)=NC2	https://www.rcsb.org/structure/4C67
4O72	2R4	413.11	O=c1cc(N2CCOCC2)oc2c(-c3cccc4c3sc3ccccc34)cccc12	https://www.rcsb.org/structure/4O72
5Y8Y	8PX	360.1	Bc1ccc(OC)c(S(=O)(=O)Nc2cc3c(C)noc3cc2OC)c1	https://www.rcsb.org/structure/5Y8Y
6JJ5	C6X	386.17	C[C@@H]1c2c(cccc2N(Cc2cccc(C(N)=O)c2)c2ccccn2)C(=O)N1C	https://www.rcsb.org/structure/6JJ5
5VZS	9U4	525.27	CNC(=O)N1CCc2c(c(N3CCCc4cc(-c5cnn(C)c5)c(C(F)F)cc43)nn2C2CCOCC2)C1	https://www.rcsb.org/structure/5VZS
5FBX	5W4	318.14	Cc1noc(C)c1-c1ccc2c(c1)[C@H](c1ccccc1)N(C)C2=O	https://www.rcsb.org/structure/5FBX
5F62	5W1	562.1	Cc1cnc(nc1Nc2ccc(c(c2)NS(=O)(=O)C(C)(C)C)Cl)Nc3ccc(c(c3)F)N4CCN(CC4)C	https://www.rcsb.org/structure/5F62
6I7X	H7B	507.02	Cc1nnc2n1-c3ccc(cc3C(=N[C@H]2CC(=O)N4CCC(CC4)N(C)C)c5ccc(cc5)Cl)OC	https://www.rcsb.org/structure/6I7X
6JJB	BT0	368.08	COc1ccccc1S(=O)(=O)Nc1ccc2c3c(cccc13)C(=O)N2C	https://www.rcsb.org/structure/6JJB
6DJC	CF6	896.43	Cc1ccc(C2=N[C@@H](CC(=O)NCCCCCCCCCCNC(=O)C[C@@H]3N=C(c4ccc(C)cc4)c4c(sc(C)c4C)-n4c(C)nnc43)c3nnc(C)n3-c3sc(C)c(C)c32)cc1	https://www.rcsb.org/structure/6DJC
5OWW	B0Q	332.14	Cn1nnc2ccc(NC(=O)c3nccn3Cc3ccccc3)cc21	https://www.rcsb.org/structure/5OWW
5VOM	9GY	417.17	CNC(=O)c1cccc(-c2ccc3c(c2)N(C(=O)c2ccco2)C[C@H](C)N3C(C)=O)c1	https://www.rcsb.org/structure/5VOM
5O97	9OE	252.06	COc1ccccc1O.[H]/C=C1/SC(=O)N=C1N	https://www.rcsb.org/structure/5O97
6S25	KSZ	534.3	COc1ccc2c(c1)[C@@H](c1ccc(C)cc1)N[C@@H](CC(=O)NCCCNC(=O)OC(C)(C)C)c1nnc(C)n1-2	https://www.rcsb.org/structure/6S25
6XUZ	O1W	479.28	COC[C@H](C)n1c(-c2cn3c(C)nnc3c(NC(C)C)n2)nc2cnc(N3CCOC[C@@H]3C)cc21	https://www.rcsb.org/structure/6XUZ
5Y8Z	8Q3	344.1	Bc1ccc(OC)c(S(=O)(=O)Nc2cc3c(C)noc3cc2C)c1	https://www.rcsb.org/structure/5Y8Z

4CL9	IES	552.29	CC(=O)N1c2ccc(-c3ccc(NC(=O)CCCCCCC(=O)NO)cc3)cc2[C@H](NC(=O)OC(C)C)C[C@@H]1C	https://www.rcsb.org/structure/4CL9
5KDH	6RX	339.12	CCn1c2c(c(=O)[nH]c1=O)[C@H](c1ccc(C)cc1)C1=C(COC1=O)N2	https://www.rcsb.org/structure/5KDH
4BJX	73B	414.19	CC(=O)N1c2ccc(-c3ccc(C(=O)O)cc3)cc2[C@H](Nc2ccc(C)cc2)C[C@@H]1C	https://www.rcsb.org/structure/4BJX
6JJ6	C89	465.5	CN1CCN(CC1)C(=O)c2cccc(c2)CN3c4cccc5c4C(=Cc6c3nccc6)N(C5=O)C	https://www.rcsb.org/structure/6JJ6
5MLI	82I	153.09	CNc1cnn(C)c(=O)c1C	https://www.rcsb.org/structure/5MLI
5F63	5W2	604.1	Cc1cnc(nc1Nc2ccc(c(c2)NS(=O)(=O)C(C)(C)C)Cl)Nc3ccc(c(c3)F)C(=O)NC4CCN(CC4)C	https://www.rcsb.org/structure/5F63
5TI7	7CQ	360.13	Bc1ccc(OC)c(S(O)(O)Nc2cccc(N3CCCC3=O)c2)c1	https://www.rcsb.org/structure/5TI7
4PCI	2NJ	252.13	CN1C(=O)C[C@@H](c2ccccc2)Nc2ccccc21	https://www.rcsb.org/structure/4PCI
5HCL	5Y9	87.07	CC(=O)N(C)C	https://www.rcsb.org/structure/5HCL
5DW2	5GD	423.5	Cc1cc(cc(c1OCCN2CCCC2)C)C3=Nc4cc(cc(c4C(=O)N3)OC)OC	https://www.rcsb.org/structure/5DW2
6Q3Y	HG5	514.6	CC[C@@H]1C(=O)N(c2cnc(nc2N1Cc3ccccc3)Nc4ccc(cc4OCC)C5CCN(CC5)C)C	https://www.rcsb.org/structure/6Q3Y
4O74	R78	521.6	CC[C@@H]1C(=O)N(c2cnc(nc2N1C3CCCC3)Nc4ccc(cc4OC)C(=O)NC5CCN(CC5)C)C	https://www.rcsb.org/structure/4O74
6VIZ	QYY	507.18	CCNC(=O)c1cc2c(-c3cc(S(=O)(=O)CC)ccc3Oc3c(C)cccc3C)cn(C)c(=O)c2[nH]1	https://www.rcsb.org/structure/6VIZ
6CKR	F5V	408.13	Cn1cc(-c2ccc3c(=O)n(C)cc(-c4cccc(NS(C)(=O)=O)c4)c3c2)cn1	https://www.rcsb.org/structure/6CKR
5A85	78J	370.4	CC1=Cc2ccnc(c2NC1=O)N[C@@H]3CCNC[C@H]3OCC4CCCCC4	https://www.rcsb.org/structure/5A85
4NUC	435	382.11	Cc1cc(/N=N/c2ccc(S(=O)(=O)Nc3ccccn3)cc2)cc(C)c1O	https://www.rcsb.org/structure/4NUC
5CP5	EB0	370.08	CCN1C(=O)c2cccc3c(S(=O)(=O)Nc4ccc(F)cc4)ccc1c23	https://www.rcsb.org/structure/5CP5
4UYD	V1T	205.09	Cn1c(=O)n(C)c2cc(C(N)=O)ccc21	https://www.rcsb.org/structure/4UYD
4WHW	3OT	495.44	C.C.C.C.C.C.C.C=O.CO.[H][H].c1ccc(CCc2nc3ccccc3n2CCN2CCOCC2)cc1	https://www.rcsb.org/structure/4WHW
4HXR	1A4	337.99	O=c1[nH]c(-c2cccc(NS(=O)(=O)c3cccs3)c2)cs1	https://www.rcsb.org/structure/4HXR
6FNX	DYZ	270.11	CCn1cnc2c1c(=O)[nH]c(=O)n2Cc1ccccc1	https://www.rcsb.org/structure/6FNX
4HBX	14X	295.1	CN1Cc2cc(S(=O)(=O)N3CCCC3)ccc2NC1=O	https://www.rcsb.org/structure/4HBX
4A9L	P9L	325.11	CN1Cc2cc(S(=O)(=O)N3CCOCC3)ccc2N(C)C1=O	https://www.rcsb.org/structure/4A9L
4E96	0NS	347.09	COc1ccccc1S(=O)(=O)Nc1ccc2c(c1)CN(C)C(=O)N2	https://www.rcsb.org/structure/4E96
6KEI	D6U	390.16	COc1ccc2c(c1Oc1ccc(OCc3ccccn3)cc1C)CN(C)C2=O	https://www.rcsb.org/structure/6KEI
6Q3Z	HG8	534.7	CC[C@@H]1C(=O)N(c2cnc(nc2N1Cc3cc(cs3)C)Nc4ccc(cc4OCC)C5CCN(CC5)C)C	https://www.rcsb.org/structure/6Q3Z
6G0D	LY2	307.12	O=c1cc(N2CCOCC2)oc2c(-c3ccccc3)cccc12	https://www.rcsb.org/structure/6G0D
3SVF	WDR	317.14	Cc1noc(C)c1-c1ccc2c(c1)[C@H](OCCO)N(C)C(=O)N2	https://www.rcsb.org/structure/3SVF
6CKS	F5Y	437.14	CCS(=O)(=O)c1ccc(OC)c(-c2cn(C)c(=O)c3ccc(-c4cnn(C)c4)cc23)c1	https://www.rcsb.org/structure/6CKS
4HXS	1A3	346.04	O=c1[nH]c(-c2cccc(NS(=O)(=O)Cc3ccccc3)c2)cs1	https://www.rcsb.org/structure/4HXS
4HBY	13F	317.08	CN1Cc2cc(S(=O)(=O)Nc3ccccc3)ccc2NC1=O	https://www.rcsb.org/structure/4HBY
5KU3	6XH	424.2	CC(=O)N1CCc2c(c(Nc3ccc(-c4cnn(C)c4)cc3F)nn2[C@H]2CCOC2)C1	https://www.rcsb.org/structure/5KU3

4QR4	BNK	338.08	Cc1ccc(-c2csc(=O)[nH]2)cc1S(=O)(=O)NC1CCCC1	https://www.rcsb.org/structure/4QR4
6KEJ	D7F	394.4	CCN(CC)CCOc1ccc2c(c1)C=C3c4c(ccc(c4O2)OC)C(=O)N3C	https://www.rcsb.org/structure/6KEJ
6G0E	EGN	414.5	CCN1c2ccccc2C(=CC1=O)SCC(=O)N3CC[NH+](CC3)C4CCCCC4	https://www.rcsb.org/structure/6G0E
3SVG	ODR	261.14	CCOc1cc(-c2c(C)noc2C)cc([C@@H](C)O)c1	https://www.rcsb.org/structure/3SVG
4GPJ	0Q1	305.14	Cc1noc(C)c1-c1ccc2c(c1)[C@](O)(c1ccccc1)CC2	https://www.rcsb.org/structure/4GPJ
4HXL	1A9	491.1	O=C(CCC1CCCCC1)Nc1cc(NS(=O)(=O)c2cccs2)cc(-c2csc(=O)[nH]2)c1	https://www.rcsb.org/structure/4HXL
5D0C	E0B	415.16	CCN1C(=O)c2cccc3c(S(=O)(=O)NCC4CCN(C(C)=O)CC4)ccc1c23	https://www.rcsb.org/structure/5D0C
4BW1	S5B	417.21	Cc1noc(C)c1C1=CC2NC=C(C(=O)O)C(Nc3ccccc3C(C)(C)C)=C2C=C1	https://www.rcsb.org/structure/4BW1
6X7B	ZVP	504.5	c1cc(cnc1)CNC(=O)c2cc(cc3c2OCCO3)c4csc5c4OC(=CC5=O)N6CCNCC6	https://www.rcsb.org/structure/6X7B
5TI2	7CJ	334.08	O=C1CCCN1c1cccc(NS(=O)(=O)c2cccc(F)c2)c1	https://www.rcsb.org/structure/5TI2
5Y8C	8P9	362.09	COc1cc2onc(C)c2cc1NS(=O)(=O)c1cc(C)ccc1OC	https://www.rcsb.org/structure/5Y8C
4WIV	3P2	361.19	Cc1noc(C)c1-c1ccc(-c2nc3cnccn3c2NC(C)(C)C)cc1	https://www.rcsb.org/structure/4WIV
4QR5	BNM	407.1	O=C(Nc1cc(-c2csc(=O)[nH]2)cc(S(=O)(=O)NC2CCCC2)c1)C1CC1	https://www.rcsb.org/structure/4QR5
5MKZ	RNK	249.09	Cc1ccsc1CNc1cnn(C)c(=O)c1C	https://www.rcsb.org/structure/5MKZ
6V1K	5SW	353.4	CN1C=C(c2ccncc2C1=O)c3cc(c(c(c3)OC)CN(C)C)OC	https://www.rcsb.org/structure/6V1K
6KEK	D7L	303.15	C.COc1ccc(C(=O)N(C)C)cc1Oc1ccc(O)cc1	https://www.rcsb.org/structure/6KEK
4HXM	1A8	423.04	CCCC(=O)Nc1cc(NS(=O)(=O)c2cccs2)cc(-c2csc(=O)[nH]2)c1	https://www.rcsb.org/structure/4HXM
4ZW1	4V1	341.4	Cc1cc(cc(c1O)C)C2=Nc3c(c4c(s3)CN(CC4)C)C(=O)N2	https://www.rcsb.org/structure/4ZW1
5CQT	EB3	358.14	CCN1C(=O)c2cccc3c(S(=O)(=O)NC4CCCC4)ccc1c23	https://www.rcsb.org/structure/5CQT
5D3R	57C	445.2	CCCc1c(C(=O)Nc2cccc(S(=O)(=O)N3CCCCCC3)c2)[nH]c(C)c1C(C)=O	https://www.rcsb.org/structure/5D3R
5HQ5	64Q	239.12	Cc1cc(Nc2ccccc2C)n2cnnc2n1	https://www.rcsb.org/structure/5HQ5
4CLB	83T	409.5	C[C@H]1C[C@H](c2cc(ccc2N1C(=O)C)c3ccc(cc3)CNC)NC(=O)OC(C)C	https://www.rcsb.org/structure/4CLB
5TI3	7CG	340.12	Bc1ccc(B)c(S(=O)(=O)Nc2cccc(N3CCCC3=O)c2)c1	https://www.rcsb.org/structure/5TI3
4PCE	2N0	253.15	CCc1cc2c(n1Cc1ccccc1)CCCC2=O	https://www.rcsb.org/structure/4PCE
5I88	69G	345.24	C.C.C.CN(C)C(=O)c1cccc(-c2cn(C)c(=O)c3[nH]ccc23)c1.[HH]	https://www.rcsb.org/structure/5I88
4MEO	25V	294.14	CC(=O)Nc1cccc(C2=C3C=CC=CC3N(O)C(C)=C2)c1	https://www.rcsb.org/structure/4MEO
4YH4	Y81	329.12	O=C1CN(C(=O)c2cncc(-c3ccccc3)c2)c2ccccc2N1	https://www.rcsb.org/structure/4YH4
4X2I	3X0	304.17	Cc1ccc(N2C[C@@H](C)c3nnc(C)n3-c3ccccc32)cc1	https://www.rcsb.org/structure/4X2I
4O70	1QK	397.23	CCc1cnn2c(NCc3ccc[n+](O)c3)cc(N3CCCC[C@H]3CCO)nc12	https://www.rcsb.org/structure/4O70
6V1L	5U6	353.4	CN1C=C(c2ccncc2C1=O)c3cc(c(cc3OC)CN(C)C)OC	https://www.rcsb.org/structure/6V1L
4LR6	1XA	174.08	Cc1noc(N)c1-c1ccccc1	https://www.rcsb.org/structure/4LR6
6YQN	P8T	379.15	Cc1ccc(C2=N[C@@H](CC(N)=O)c3nnc(C)n3-c3sc(C)c(C)c32)cc1	https://www.rcsb.org/structure/6YQN
5Y8W	8PU	346.08	Bc1ccc(OC)c(S(=O)(=O)Nc2cc3c(C)noc3cc2O)c1	https://www.rcsb.org/structure/5Y8W

4QB3	30M	299.16	CC(=O)NCCCCn1c2c(c3ccccc31)C(=O)NCC2	https://www.rcsb.org/structure/4QB3
4HXN	1A7	195.02	O=c1[nH]c(-c2ccccc2F)cs1	https://www.rcsb.org/structure/4HXN
5U28	SCN	61	[H]/N=C\S	https://www.rcsb.org/structure/5U28
5U28	82V	373.1	O=C1C[C@@H](N2CCOCC2)Oc2c(-c3ccc4c(c3)OCCO4)csc21	https://www.rcsb.org/structure/5U28
6JJ3	BS6	467.5	CN1CCN(CC1)c2cc3c4c(ccc(c4n2)NS(=O)(=O)c5ccccc5OC)N(C3=O)C	https://www.rcsb.org/structure/6JJ3
5HQ6	64R	177.08	Cc1ccc2c(c1)NC(=O)[C@@H](C)O2	https://www.rcsb.org/structure/5HQ6
6X7D	UT4	370.4	c1cc2c(cc1c3csc4c3OC(=CC4=O)N5CCNCC5)OCCO2	https://www.rcsb.org/structure/6X7D
5F60	5VZ	544.11	Cc1cnc(nc1Nc2ccc(c(c2)NS(=O)(=O)C(C)(C)C)Cl)Nc3ccc(cc3)N4CCN(CC4)C	https://www.rcsb.org/structure/5F60
5TI4	7CM	348.09	Cc1cc(S(=O)(=O)Nc2cccc(N3CCCC3=O)c2)ccc1F	https://www.rcsb.org/structure/5TI4
5U2E	837	393.16	C.CCOC(=O)c1ccc(-c2csc(C=O)c2OCN2CCOCC2)cc1.[HH]	https://www.rcsb.org/structure/5U2E
6MAU	JBS	526.27	Cc1noc(C)c1-c1ccc2c(c1)C(N1CCOC[C@@H]1c1ccccc1)N=C(c1cnn(CC(C)(C)O)c1)N2	https://www.rcsb.org/structure/6MAU
6FSY	E5Q	296.12	Cc1noc(C)c1-c1cc(O)cc([C@@H](O)c2cccnc2)c1	https://www.rcsb.org/structure/6FSY
5CRZ	EB7	384.09	CCN1C(=O)c2cccc3c(NS(=O)(=O)c4ccc(F)cc4C)ccc1c23	https://www.rcsb.org/structure/5CRZ
6KEE	D7U	445.09	Cc1cc(-c2cc(CS(C)(=O)=O)cnc2Oc2ccc(F)cc2F)n2cc[nH]c(=O)c12	https://www.rcsb.org/structure/6KEE
5IGK	BMF	404.13	CCOC(=O)Nc1cc(-c2ccc(C)c(NS(C)(=O)=O)c2)nn2c(C)nnc12	https://www.rcsb.org/structure/5IGK
4MEP	24Y	345.16	Cc1cccnc1N1NC(c2ccccc2)=NC1c1c[nH]c(=O)c(C)c1	https://www.rcsb.org/structure/4MEP
6CD4	EX1	598.7	CC(C)N1c2ccccc2C(=O)N(c3c1nc(nc3)Nc4ccc(cc4OC)C(=O)N5CCC(CC5)N6CCN(CC6)C)C	https://www.rcsb.org/structure/6CD4
5LRQ	4WG	474.24	CCOc1cc(N2CCC(O)CC2)ccc1Nc1ncc2c(n1)N(C)c1ccccc1C(=O)N2C	https://www.rcsb.org/structure/5LRQ
6VIW	QYV	501.12	CCNC(=O)c1cc2c(-c3cc(S(C)(=O)=O)ccc3Oc3ccc(F)cc3F)cn(C)c(=O)c2[nH]1	https://www.rcsb.org/structure/6VIW
5CS8	EB8	410.09	CCN1C(=O)c2cccc3c(NS(=O)(=O)c4ccccc4C(=O)OC)ccc1c23	https://www.rcsb.org/structure/5CS8
6YQO	P8Q	470.19	Cc1ccc(C2=N[C@@H](CC(=O)Nc3ccc(N)cc3)c3nnc(C)n3-c3sc(C)c(C)c32)cc1	https://www.rcsb.org/structure/6YQO
6LG9	ECU	238.1	Bc1ccc2c(-n3ccnc3)cc(N)nc2c1O	https://www.rcsb.org/structure/6LG9
5W55	X30	584.7	CCN1c2ccccc2C(=O)N(c3c1nc(nc3)Nc4ccc(cc4OC)C(=O)N5CCC(CC5)N6CCN(CC6)C)C	https://www.rcsb.org/structure/5W55
4HXO	1A6	246.06	Cc1cc(CSc2nnc3ccccn23)on1	https://www.rcsb.org/structure/4HXO
5EGU	5NQ	484.24	CCCCc1c(C)nc2nc(SCc3nc4c(c(=O)[nH]c(=O)n4CCCC)n3CC)nn2c1C	https://www.rcsb.org/structure/5EGU
5D3T	56Y	341.17	CCc1c(C(=O)N(C)Cc2cccc(C(N)=O)c2)[nH]c(C)c1C(C)=O	https://www.rcsb.org/structure/5D3T
5HQ7	64S	235.13	CCNC1N=CNc2cc(OC)c(OC)cc21	https://www.rcsb.org/structure/5HQ7
5Z5T	96R	226.09	Nc1cc(-n2ccnc2)c2cccc(O)c2n1	https://www.rcsb.org/structure/5Z5T
5TI5	7CN	326.09	Bc1cccc(S(=O)(=O)Nc2cccc(N3CC=CC3=O)c2)c1	https://www.rcsb.org/structure/5TI5
6S6K	KXK	473.2	COc1ccc(S(=O)(=O)N2CCCCCC2)cc1NC(=O)c1[nH]c(C)c2c1CCCCC2=O	https://www.rcsb.org/structure/6S6K
6XVC	O32	387.17	Cc1nnc2ccc(-c3cc(O[C@H](C)[C@H]4CNC(=O)C4)c4cccnc4c3)cn12	https://www.rcsb.org/structure/6XVC

6WGX	U0D	563.6	Cc1cc(cc(c1)Nc2nccc(n2)c3c(ncn3C4CCN(CC4)CCN(C)C)c5ccc(cc5)C(F)(F)F)C	https://www.rcsb.org/structure/6WGX
6KEF	D7X	409.11	Cc1cc(-c2cc(NS(C)(=O)=O)ccc2Oc2ccccc2)n2cc[nH]c(=O)c12	https://www.rcsb.org/structure/6KEF
4MEQ	25O	227.12	CC1=C[C@H](c2ccccc2)N2NC(N)=NC2=N1	https://www.rcsb.org/structure/4MEQ
6CD5	R4L	624.7	CN1CCN(CC1)C2CCN(CC2)C(=O)c3ccc(c(c3)OC)Nc4ncc5c(n4)N(c6ccccc6C(=O)N5C)C7CCCC7	https://www.rcsb.org/structure/6CD5
6S4B	KUH	517.38	C.CCN(CC)S(=O)(=O)c1ccc(OC)c(NC(=O)c2cc(C(C)=O)c[nH]2)c1.C[C@@H](O)[C@@H](C)O.[HH].[HH].[HH].[HH].[HH].[HH].[H][H].[H][H].[H][H]	https://www.rcsb.org/structure/6S4B
5XHY	8FC	399.16	Cc1ccc(S(=O)(=O)Nc2ccc3c(c2)N(C2CC2)C(C)(C)C(=O)N3C)cc1	https://www.rcsb.org/structure/5XHY
6CIY	F3J	610.7	CN1CCN(CC1)C2CCN(CC2)C(=O)c3ccc(c(c3)OC)Nc4ncc5c(n4)N(c6ccccc6C(=O)N5C)C7CCC7	https://www.rcsb.org/structure/6CIY
5BT4	2LO	488.28	COc1ccc(CCc2nc3cc(-c4c(C)noc4C)ccc3n2C[C@H](C)N2CCOCC2)cc1C	https://www.rcsb.org/structure/5BT4
3P5O	EAM	403.2	CCNC(=O)C[C@@H]1N=C(c2ccc(C)cc2)c2cc(OC)ccc2-n2c(C)nnc21	https://www.rcsb.org/structure/3P5O
5AD2	ETU	483.9	C[C@@H]1C(=O)N(CCN1CCOc2ccc(cc2)C3CCN(CC3)c4ccc5nnc(n5n4)Cl)C	https://www.rcsb.org/structure/5AD2
5WA5	4K4	570.6	CN1CCN(CC1)C2CCN(CC2)C(=O)c3ccc(c(c3)OC)Nc4ncc5c(n4)N(c6ccccc6C(=O)N5C)C	https://www.rcsb.org/structure/5WA5
4HBV	15E	174.1	Bc1ccc2c(c1)CN(C)C(=O)N2	https://www.rcsb.org/structure/4HBV
6XV7	O2Q	289.11	Cc1nnc2ccc(N(C)Cc3ccc(F)c(F)c3)nn12	https://www.rcsb.org/structure/6XV7
5Z5U	96U	242.08	Nc1cc(-n2ccnc2)c2cc(O)cc(O)c2n1	https://www.rcsb.org/structure/5Z5U
6KEG	D89	459.11	CCS(=O)(=O)Cc1cnc(Oc2ccc(F)cc2F)c(-c2cc(C)c3n2CC=NC3=O)c1	https://www.rcsb.org/structure/6KEG
5HLS	62G	345.15	Cc1ccc(C2=N[C@@H](CC(N)=O)c3onc(C)c3-c3ccccc32)cc1	https://www.rcsb.org/structure/5HLS
6RWJ	KLK	220.12	CNC(=O)c1[nH]c(C)c2c1CCCCC2=O	https://www.rcsb.org/structure/6RWJ
5AD3	K6K	534.28	COC1=NNC2=CC=C(N3CCC(c4ccc(OCCN(C)C5=NN6C(OC)=NN[C@H]6C=C5)cc4)CC3)NN21	https://www.rcsb.org/structure/5AD3
5D3N	L40	377.14	CCc1c(C(=O)Nc2cc(S(=O)(=O)NC)ccc2C)[nH]c(C)c1C(C)=O	https://www.rcsb.org/structure/5D3N
4LYW	21Q	421.17	CCc1c(C(=O)Nc2cc(S(=O)(=O)N(CC)CC)ccc2O)[nH]c(C)c1C(C)=O	https://www.rcsb.org/structure/4LYW
5Z5V	96X	284.13	CC(=O)Nc1cc(CN2C=CNC2)c2cccc(O)c2n1	https://www.rcsb.org/structure/5Z5V
6DMJ	53W	460.25	COc1ccc(CCc2nc3cc(-c4c(C)noc4C)ccc3n2CCN2CCOCC2)cc1	https://www.rcsb.org/structure/6DMJ
5ULA	5MJ	350.16	COc1ccc(NC(=O)c2ccc(NC3=CCC(=O)C3C)c(C)c2)cc1	https://www.rcsb.org/structure/5ULA
4NQM	SIN	118.03	O=C(O)CCC(=O)O	https://www.rcsb.org/structure/4NQM
4NQM	Y1Z	514.18	Cc1ccccc1S(=O)(=O)Nc1cc(-c2nn3c(C)nnc3c3ccccc23)ccc1N1CCOCC1	https://www.rcsb.org/structure/4NQM
6CIS	X26	652.8	CC(C)Oc1cc(ccc1Nc2ncc3c(n2)N(c4ccccc4C(=O)N3C)C5CCCC5)C(=O)N6CCC(CC6)N7CCN(CC7)C	https://www.rcsb.org/structure/6CIS
6FO5	DZH	522.17	CCn1cnc2c1c(=O)[nH]c(=O)n2Cc1ccc(CNS(=O)(=O)c2ccc3c(c2)CCCC(=O)N3)cc1	https://www.rcsb.org/structure/6FO5
6KEH	D6R	317.16	C.COc1ccc(Oc2cc(C(=O)N(C)C)ccc2OC)cc1	https://www.rcsb.org/structure/6KEH
2YEL	WSH	423.17	Cc1nnc2n1-c1ccccc1C(c1ccccc1)=N[C@H]2NC(=O)OCc1ccccc1	https://www.rcsb.org/structure/2YEL

5M39	7EA	270.11	COc1ccc(-c2ccc3nnc(C)n3n2)cc1OC	https://www.rcsb.org/structure/5M39
6SAH	L2W	459.18	COc1ccc(S(=O)(=O)N2CCCCC2)cc1NC(=O)c1[nH]c(C)c2c1CCCCC2=O	https://www.rcsb.org/structure/6SAH
5Z90	99U	330.1	CCN1C(=O)c2cccc3c(S(=O)(=O)N4CCCC4)ccc1c23	https://www.rcsb.org/structure/5Z90
4QR3	BNJ	324.06	O=c1[nH]c(-c2cccc(S(=O)(=O)NC3CCCC3)c2)cs1	https://www.rcsb.org/structure/4QR3
6LG5	EC3	256.12	Cc1cc(CN2CCCC2=O)c(O)c2ncccc12	https://www.rcsb.org/structure/6LG5
4HXK	1AJ	233.06	O=C(N1CCc2sccc2C1)n1ccnc1	https://www.rcsb.org/structure/4HXK
5D3P	57E	387.13	CCc1c(C(=O)n2ccc3ccc(S(=O)(=O)NC)cc32)[nH]c(C)c1C(C)=O	https://www.rcsb.org/structure/5D3P
6MH1	JQP	442.53	Cc1cc(cc(c1)Nc2nccc(n2)c3c(ncn3C4CCNCC4)c5ccc(cc5)F)C	https://www.rcsb.org/structure/6MH1
5F5Z	5VY	509.6	Cc1cnc(nc1Nc2cccc(c2)NS(=O)(=O)C(C)(C)C)Nc3ccc(cc3)N4CCN(CC4)C	https://www.rcsb.org/structure/5F5Z
3ZYU	1GH	415.16	COc1cc2c(cc1-c1c(C)noc1C)ncc1[nH]c(=O)n([C@H](C)c3ccccn3)c12	https://www.rcsb.org/structure/3ZYU
5UVW	8NG	459.11	CCS(=O)(=O)Nc1ccc(Oc2ccc(F)cc2F)c(-c2cn(C)c(=O)c3[nH]ccc23)c1	https://www.rcsb.org/structure/5UVW
5Y1Y	HNQ	191.05	O=[N+](O)c1ccc(O)c2ncccc12	https://www.rcsb.org/structure/5Y1Y
5COI	55K	402.12	CCN1C(=O)c2cccc3c(S(=O)(=O)NCC4(C(=O)O)CCCC4)ccc1c23	https://www.rcsb.org/structure/5COI
6FT4	E5W	322	Cc1c(c(on1)C)c2cc(cc(c2)O)CN3CCC(CC3)(F)F	https://www.rcsb.org/structure/6FT4
6LG6	EC9	296.13	CC(=O)NC1=CC(Nc2ccccn2)=C2CC=CC(=O)C2N1	https://www.rcsb.org/structure/6LG6
5A5S	NP8	365.429	CC1=Cc2c(cnc(c2NC1=O)NC3CCNCC3)c4cc(cnc4)OC	https://www.rcsb.org/structure/5A5S
6SAJ	L2Z	473.16	COc1ccc(S(=O)(=O)N2CC3(COC3)C2)cc1NC(=O)c1[nH]c(C)c2c1CCCCC2=O	https://www.rcsb.org/structure/6SAJ
4J0R	1H2	295.12	Cc1noc(C)c1-c1cc(O)cc([C@H](O)c2ccccc2)c1	https://www.rcsb.org/structure/4J0R
6XV3	O2B	511.28	Cc1nnc2c(NC(C)C)nc(-c3nc4cnc(N5CCOC[C@@H]5C)cc4n3[C@@H](C)c3ccccc3)cn12	https://www.rcsb.org/structure/6XV3
5Y93	8Q9	516.19	Bc1ccc(OC)c(S(=O)(=O)Nc2cc3c(C)noc3cc2OCC(=O)NCCN2CCOCC2)c1	https://www.rcsb.org/structure/5Y93
4O7F	2RQ	445.4	COc1ccccc1Oc2nccc(n2)c3c(ncn3C4CCNCC4)c5ccc(cc5)F	https://www.rcsb.org/structure/4O7F
5DLX	5D2	497	Cc1nnc2n1nc(cc2)N3CCC(CC3)C(=O)NCCCN4CCN(CC4)c5cccc(c5)Cl	https://www.rcsb.org/structure/5DLX
5N2M	8J2	423.22	CC(=O)Nc1cccc(-c2ccc3c(c2)[C@H](NC(=O)OC(C)C)C[C@H](C)N3C(C)=O)c1	https://www.rcsb.org/structure/5N2M
6AFR	9E3	243.08	Oc1ccc(Cn2cnc(F)c2)c2cccnc12	https://www.rcsb.org/structure/6AFR
6KEC	D9C	379.33	C.C.C.C.CC.CCO.CO.COc1ccc2c(c1OC)CN(C)C2=O	https://www.rcsb.org/structure/6KEC
5LUU	77X	255.14	CCC(=O)N1CCc2[nH]nc(-c3ccccc3)c2C1	https://www.rcsb.org/structure/5LUU
4MEN	25K	269.16	CC1=CC(N(C)Cc2ccc(C)cc2)N2NC=NC2=N1	https://www.rcsb.org/structure/4MEN
7KHL	WEM	514.11	COC(=O)c1cc2c(cc1CS(C)(=O)=O)-c1cn(C)c(=O)c3[nH]cc(c13)CN2c1ncc(F)cc1F	https://www.rcsb.org/structure/7KHL
6UWX	QKD	271.07	CCOC(=O)N1CCS[C@H](c2cccs2)CC1	https://www.rcsb.org/structure/6UWX
6LIM	EE9	438.22	CC(C)N1c2cc(Nc3nccc(-c4cncc5ccccc45)n3)ccc2N(C)C(=O)[C@H]1C	https://www.rcsb.org/structure/6LIM
6LG7	ECF	244.08	Nc1cc(-n2ccnc2)c2cc(F)cc(O)c2n1	https://www.rcsb.org/structure/6LG7

5D3J	L33	405.17	CCc1c(C(=O)Nc2cccc(S(=O)(=O)N(CC)CC)c2)[nH]c(C)c1C(C)=O	https://www.rcsb.org/structure/5D3J
5XI4	8F0	349.22	Cc1ccc([C@H](C)Nc2ccc3c(c2)N(C2CC2)[C@@H](C)C(=O)N3C)cc1	https://www.rcsb.org/structure/5XI4
6VUF	RLV	296.12	CCCNS(=O)(=O)c1ccc([C@H]2CC(=O)N(C)C2)cc1	https://www.rcsb.org/structure/6VUF
5M3A	7E7	306.12	Cc1nnc2ccc(-c3cnn(C)c3Oc3ccccc3)nn12	https://www.rcsb.org/structure/5M3A
5Y94	8QC	359.11	Bc1ccc(OC)c(S(=O)(=O)Nc2cc3c(C)noc3cc2NC)c1	https://www.rcsb.org/structure/5Y94
5I80	67B	295.13	CN(C)C(=O)c1cccc(-c2cn(C)c(=O)c3[nH]ccc23)c1	https://www.rcsb.org/structure/5I80
5Z1R	EFL	374.11	Bc1ccc(OC)c(S(=O)(=O)Nc2ccc3c(c2)OC(C)(C)C(=O)N3)c1	https://www.rcsb.org/structure/5Z1R
6E4A	HRY	532	CN1C=C(C=CC1=O)c2ccc3c(c2)c(nc(n3)N4CCN(CC4)CCN(C)C)NCc5cccc(c5)Cl	https://www.rcsb.org/structure/6E4A
6PS9	Y17	311.16	CN1C[C@H](CCn2c3c(c4ccccc42)C(=O)NCC3)CC1=O	https://www.rcsb.org/structure/6PS9
5WUU	7UU	344.12	CN(CCCNc1cc(=O)oc2ccccc12)C(=O)C1=CCCS1	https://www.rcsb.org/structure/5WUU
4ZC9	4MW	764.27	Cc1ccc(C2=N[C@@H](CC(=O)NCCCCNC(=O)COc3cccc4c3C(=O)N([C@H]3CCC(=O)NC3=O)C4=O)c3nnc(C)n3-c3sc(C)c(C)c32)cc1	https://www.rcsb.org/structure/4ZC9
6PRT	OWA	171.09	COC(=O)C[C@H]1CC(=O)N(C)C1	https://www.rcsb.org/structure/6PRT
6LG8	ECR	244.08	Nc1cc(-n2ccnc2)c2c(F)ccc(O)c2n1	https://www.rcsb.org/structure/6LG8
5Z9K	99X	360.11	CCN1C(=O)c2cccc3c(S(=O)(=O)N4CCC[C@@H]4CO)ccc1c23	https://www.rcsb.org/structure/5Z9K
6YIN	OS8	422.23	COc1ccc2c(c1)N(CCCNC(=O)c1cccc3c1N[C@H](C)CC(=O)N3)CCC2	https://www.rcsb.org/structure/6YIN
4CFL	8DQ	306.3	c1ccc(cc1)c2cccc3c2OC(=CC3=O)N4CCNCC4	https://www.rcsb.org/structure/4CFL
5LJ1	6XX	527.6	Cc1cc(cnc1)c2cnc(c3c2C=C(C(=O)N3)C)N[C@@H]4[C@H](CNC[C@H]4OCC5CCC(CC5)(F)F)OC	https://www.rcsb.org/structure/5LJ1
4UIZ	N1D	491.12	COc1ccc(-c2cn(C)c(=O)c3cc(C(=O)N4CCN(S(C)(=O)=O)CC4)sc23)cc1OC	https://www.rcsb.org/structure/4UIZ
5CY9	E0A	332.12	CCCCS(=O)(=O)Nc1ccc2c3c(cccc13)C(=O)N2CC	https://www.rcsb.org/structure/5CY9
5DLZ	5D1	461.5	Cc1cccc(c1)CN2CCC(CC2)CNC(=O)CCCOC3=CC(=O)N(c4c3cccc4)C	https://www.rcsb.org/structure/5DLZ
5YOU	8XX	363.19	Cc1ccc(N(C)C(=O)c2ccc3c(c2)N(C2CC2)[C@H](C)C(=O)N3C)cc1	https://www.rcsb.org/structure/5YOU
5Z1S	EFM	404.12	Bc1ccc(OC)c(S(=O)(=O)Nc2cc3c(cc2OC)NC(=O)C(C)(C)O3)c1	https://www.rcsb.org/structure/5Z1S
6I7Y	H7E	613.1	Cc1nnc2n1-c3ccc(cc3C(=N[C@H]2CC(=O)Nc4ccc(cc4)N5CCC(CC5)[N+](C)(C)C)c6ccc(cc6)Cl)OC	https://www.rcsb.org/structure/6I7Y
5Z8G	99L	360.11	CCN1C(=O)c2cccc3c(S(=O)(=O)N4CCC[C@@H](O)C4)ccc1c23	https://www.rcsb.org/structure/5Z8G
4PS5	2TA	524.6	Cc1cnc(nc1Nc2cccc(c2)S(=O)(=O)NC(C)(C)C)Nc3ccc(cc3)OCCN4CCCC4	https://www.rcsb.org/structure/4PS5
5H21	RMR	323.12	COc1cc(C(=O)NCC[C@H]2C=CCS2)cc(OC)c1OC	https://www.rcsb.org/structure/5H21
5D3L	57F	435.18	CCc1c(C(=O)Nc2cc(S(=O)(=O)N(CC)CC)c(C)cc2O)[nH]c(C)c1C(C)=O	https://www.rcsb.org/structure/5D3L
6P05	YF2	552.19	CCS(=O)(=O)Nc1cc(-c2cn(C)c3c(=O)[nH]ccc23)cc2c1ccn2C(C)(c1ccccn1)c1ccccn1	https://www.rcsb.org/structure/6P05
5WMD	6JE	471.17	Cc1ccc(C2=N[C@@H](CC(=O)Nc3ccc(O)cc3)c3nnc(C)n3-c3sc(C)c(C)c32)cc1	https://www.rcsb.org/structure/5WMD
4O7A	2RF	308.12	Cc1cccc(C2=C(Nc3ccc(O)c(C)c3)C(=O)NC2=O)c1	https://www.rcsb.org/structure/4O7A
5Z8Z	99R	359.4	CCN1c2ccc(c3c2c(ccc3)C1=O)S(=O)(=O)N4CCC[C@@H](C4)N	https://www.rcsb.org/structure/5Z8Z

5LJ2	6XW	512.62	CC1=Cc2c(cnc(c2NC1=O)N[C@@H]3CCNC[C@H]3OCC4CCS(=O)(=O)CC4)c5cc(cnc5)N	https://www.rcsb.org/structure/5LJ2
4LRG	1XB	379.14	Cc1ccc(C2=N[C@@H](CC(N)=O)c3onc(C)c3-c3sc(C)c(C)c32)cc1	https://www.rcsb.org/structure/4LRG
6YQZ	P8W	305.15	Cc1c(NCc2ccccc2-c2ccccc2)cnn(C)c1=O	https://www.rcsb.org/structure/6YQZ
5YOV	8XR	391.23	Cc1ccc(NC(=O)c2ccc3c(c2)N(C2CCCC2)[C@H](C)C(=O)N3C)c(C)c1	https://www.rcsb.org/structure/5YOV
5Z1T	EFN	390.11	Bc1ccc(OC)c(S(=O)(=O)Nc2cc3c(cc2O)NC(=O)C(C)(C)O3)c1	https://www.rcsb.org/structure/5Z1T
5OVB	AY2	429.17	CCOc1ccc(C(C)=O)cc1-c1cc(NC(=O)c2ccco2)cc(-c2ccn(C)n2)c1	https://www.rcsb.org/structure/5OVB
6LIH	EDF	387.21	CC(C)N1c2cc(Nc3nccc(-c4ccccc4)n3)ccc2N(C)C(=O)[C@H]1C	https://www.rcsb.org/structure/6LIH
5YQX	E0K	298.13	Cc1noc(C)c1-c1ccc2c(c1)O[C@H](CC1CC1)C(=O)N2	https://www.rcsb.org/structure/5YQX
4NUD	NUD	383.11	Cc1cc(/N=N/c2ccc(S(=O)(=O)Nc3ccccn3)cc2)c(N)cc1O	https://www.rcsb.org/structure/4NUD
5HM0	62V	288.13	Cc1ccc(C2=NCc3onc(C)c3-c3ccccc32)cc1	https://www.rcsb.org/structure/5HM0
4O7B	2RJ	443.5	Cc1cccc(c1C)Oc2nccc(n2)c3c(ncn3C4CCNCC4)c5ccc(cc5)F	https://www.rcsb.org/structure/4O7B
5D24	L26	343.15	CCc1c(C(=O)Nc2cccc(OCC(N)=O)c2)[nH]c(C)c1C(C)=O	https://www.rcsb.org/structure/5D24
4LZS	L46	208.12	CCc1c(C(=O)NC)[nH]c(C)c1C(C)=O	https://www.rcsb.org/structure/4LZS
3MXF	JQ1	436.19	Cc1ccc(C2=N[C@@H](CC(=O)OC(C)(C)C)c3nnc(C)n3-c3sc(C)c(C)c32)cc1	https://www.rcsb.org/structure/3MXF
4MR3	1K0	326.13	COc1cc(OC)c2c(=O)[nH]c(-c3cc(C)c(O)c(C)c3)nc2c1	https://www.rcsb.org/structure/4MR3
6JI4	BOF	387.21	Cc1ccc(-n2c(C)nnc2-c2ccc3c(c2)N(C2CC2)[C@H](C)C(=O)N3C)cc1	https://www.rcsb.org/structure/6JI4
6VUB	RLG	175.1	CN1C[C@@H](c2ccccc2)CC1=O	https://www.rcsb.org/structure/6VUB
4Z1S	559	396.21	Cc1ccc(N2C[C@H](C)c3nnc(C)n3-c3ccc(-c4ccc(N)nc4)cc32)cc1	https://www.rcsb.org/structure/4Z1S
4NUE	NUE	397.12	Cc1cc(/N=N/c2ccc(S(=O)(=O)Nc3ccccn3)cc2)c(N)c(C)c1O	https://www.rcsb.org/structure/4NUE
5CRM	EB5	304.09	CCNS(=O)(=O)c1ccc2c3c(cccc13)C(=O)N2CC	https://www.rcsb.org/structure/5CRM
5D25	56M	479.27	CCN(CC)S(=O)(=O)c1ccc(O)c(NC=O)c1.C[C@@H](O)[C@@H](C)O.[H]/C=C(C(C)=O)/C(C)=N/[H].[H][H].[H][H].[H][H]	https://www.rcsb.org/structure/5D25
4DON	3PF	162.08	CN1Cc2ccccc2NC1=O	https://www.rcsb.org/structure/4DON
4XY9	43U	253.11	Bc1ccc(OC)c(-c2nc(N)nc3[nH]cnc23)c1	https://www.rcsb.org/structure/4XY9
5ACY	9S3	453.6	C[C@H]1C[C@H](c2cc(ccc2N1C(=O)C)c3ccc(cc3)CN4CCCCC4)Nc5ccccc5	https://www.rcsb.org/structure/5ACY
6UWU	QKP	482.56	Cc1cc(cc(c1OC[C@@H](CN2CCN(CC2)C)O)C)C3=CC(=O)c4c(cc(cc4OC)OC)O3	https://www.rcsb.org/structure/6UWU
6LG4	EC0	205.06	Cc1cc([N+](=O)O)c2cccnc2c1O	https://www.rcsb.org/structure/6LG4
4MR4	1K0	370.15	COc1cc(OC)c2c(=O)[nH]c(-c3cc(C)c(OCCO)c(C)c3)nc2c1	https://www.rcsb.org/structure/4MR4
6G0F	EGH	343.14	Bc1ccc(F)c([C@@H]2CC(=O)Nc3cc(OC)c(OC)c(OC)c32)c1	https://www.rcsb.org/structure/6G0F
4O77	2RE	331.11	Oc1ccc(-c2nc(-c3ccc(F)cc3)c(-c3ccncc3)[nH]2)cc1	https://www.rcsb.org/structure/4O77
6SB8	L45	523.24	CCN(CC)S(=O)(=O)c1ccc(O)c(NC(=O)c2[nH]c(C)c3c2CCCCC3=O)c1.C[C@@H](O)[C@@H](C)O	https://www.rcsb.org/structure/6SB8
6JI5	BQ0	545.35	Cc1ccc(N2NC(C(=O)N3CCN(C)CC3)=NC2c2ccc3c(c2)N(C2CCCC2)[C@H](C)[C@H](O)N3C)c(C)c1	https://www.rcsb.org/structure/6JI5

6VUC	RLS	322.14	CN1C[C@@H](c2ccc(S(=O)(=O)N3CCCCC3)cc2)CC1=O	https://www.rcsb.org/structure/6VUC
4BW2	UTH	416.18	Cc1noc(C)c1-c1ccc2c(Nc3ccccc3C(C)(C)C)c(C(=O)O)cnc2n1	https://www.rcsb.org/structure/4BW2
4IOO	BAE	115.1	CNC(=O)C(C)(C)C	https://www.rcsb.org/structure/4IOO
5CPE	EB2	372.15	CCN1C(=O)c2cccc3c(S(=O)(=O)NC4CCCCC4)ccc1c23	https://www.rcsb.org/structure/5CPE
6DL2	GUJ	311.11	Cc1sc2c(c1Cc1ccccc1)COCc1nnc(C)n1-2	https://www.rcsb.org/structure/6DL2
3U5J	08H	290.15	Cc1ccc2c(c1)[C@@H](c1ccccc1)NCc1nnc(C)n1-2	https://www.rcsb.org/structure/3U5J
5D3H	57G	437.16	CCc1c(C(=O)Nc2cc(S(=O)(=O)N(CC)CC)ccc2O)[nH]c(C)c1C(=O)CO	https://www.rcsb.org/structure/5D3H
6G0G	SAS	398.07	O=C(O)c1cc(/N=N/c2ccc(S(=O)(=O)Nc3ccccn3)cc2)ccc1O	https://www.rcsb.org/structure/6G0G
6CJ2	X27	487.5	CC(C)N1c2ccccc2C(=O)N(c3c1nc(nc3)Nc4ccc(c(c4)OC)N5CCN(CC5)C)C	https://www.rcsb.org/structure/6CJ2
5V67	IBI	618.8	CC[C@@H]1C(=O)N(c2cnc(nc2N1C(C)C)Nc3ccc(cc3OC)C(=O)NC4CCC(CC4)N5CCN(CC5)CC6CC6)C	https://www.rcsb.org/structure/5V67
4BW3	9BM	446.2	COc1nc2c(Nc3ccccc3C(C)(C)C)c(C(=O)O)cnc2cc1-c1c(C)noc1C	https://www.rcsb.org/structure/4BW3
5KHM	XNH	479.57	C[C@@H]1C(=O)N(CCN1CCOc2ccc(cc2)C3CCN(CC3)c4ccc5nnc(n5n4)OC)C	https://www.rcsb.org/structure/5KHM
6UVJ	QJ1	257.05	COC(=O)N1CCS[C@H](c2cccs2)CC1	https://www.rcsb.org/structure/6UVJ
6WW8	YR0	584.22	CN(C)C(=O)c1cc2cnc(Nc3ccc(-c4csc5c(=O)cc(N6CCOCC6)oc45)cc3)nc2n1C1CCCC1	https://www.rcsb.org/structure/6WW8
5EIS	5OU	284.13	CCn1cnc2c1c(=O)[nH]c(=O)n2Cc1ccc(C)cc1	https://www.rcsb.org/structure/5EIS
3U5K	08J	305.13	Cc1ccc2c(c1)C(c1ccccc1F)=NCc1cnc(C)n1-2	https://www.rcsb.org/structure/3U5K
4C66	H4C	322.13	CCc1cc2c(s1)-n1c(C)nnc1CN=C2c1ccccc1C	https://www.rcsb.org/structure/4C66
5CFW	53W	460.25	COc1ccc(CCc2nc3cc(-c4c(C)noc4C)ccc3n2CCN2CCOCC2)cc1	https://www.rcsb.org/structure/5CFW
4O71	CPB	401.8	C[N@]1CC[C@@H]([C@@H](C1)O)c2c(cc(c3c2OC(=CC3=O)c4ccccc4Cl)O)O	https://www.rcsb.org/structure/4O71
5KJ0	6TB	535.6	CC[C@@H]1C(=O)N(c2cnc(nc2N1C3CCCC3)N(C)c4ccc(cc4OC)C(=O)NC5CCN(CC5)C)C	https://www.rcsb.org/structure/5KJ0
5XI3	8F6	335.2	C[C@@H]1C(=O)N(C)c2ccc(N[C@H](C)c3ccccc3)cc2N1C1CC1	https://www.rcsb.org/structure/5XI3
6G0H	EGE	413.08	Nc1cc(O)c(C(=O)O)cc1N=Nc1ccc(S(=O)(=O)Nc2ccccn2)cc1	https://www.rcsb.org/structure/6G0H
5WMA	6JC	213.09	Cc1noc(C)c1-c1cnc2[nH]ccc2c1	https://www.rcsb.org/structure/5WMA
4UIX	TVU	476.11	COc1ccc(-c2cn(C)c(=O)c3cc(C(=O)NC4CCS(=O)(=O)CC4)sc23)cc1OC	https://www.rcsb.org/structure/4UIX
6HDQ	FZE	521.64	Cc1cc(cnc1)c2cnc(c3c2C=C(C(=O)N3)C)N[C@@H]4C[C@H]5CC[C@@H]([C@H]4CCC6CCC(CC6)(F)F)N5	https://www.rcsb.org/structure/6HDQ
4BW4	9B6	471.12	COc1nc2c(cc1-c1c(C)noc1C)ncc1[nH]c(=O)n(-c3ccccc3OC(F)(F)F)c12	https://www.rcsb.org/structure/4BW4
4IOQ	BAQ	85.05	O=C1CCCN1	https://www.rcsb.org/structure/4IOQ
5F61	5W0	527.6	Cc1cnc(nc1Nc2cccc(c2)NS(=O)(=O)C(C)(C)C)Nc3ccc(c(c3)F)N4CCN(CC4)C	https://www.rcsb.org/structure/5F61
BRD7				
PDB ID	Ligand Name	Ligand MW	Ligand-smiles	URL

5E9V	5L0	360.16	CC(=O)c1cc(-c2cccc3nccn23)c2cc(N3CCOCC3)ccn12	https://www.rcsb.org/structure/5E9V
4UIT	N1D	491.12	COc1ccc(-c2cn(C)c(=O)c3cc(C(=O)N4CCN(S(C)(=O)=O)CC4)sc23)cc1OC	https://www.rcsb.org/structure/4UIT
4XY8	43U	255.13	B.COc1ccccc1-c1nc(N)nc2[nH]cnc12	https://www.rcsb.org/structure/4XY8
5F25	5TU	242.11	Cc1cc(-c2ccc(C(N)=O)cc2)cn(C)c1=O	https://www.rcsb.org/structure/5F25
5MKY	I0D	304.7	CN1CCc2c(cccc2NC3=C(C(=O)N(N=C3)C)Cl)C1	https://www.rcsb.org/structure/5MKY
4Z6H	4L2	270.14	Cc1cc(=O)n(C)c2cc(N3CCCCC3=O)ccc12	https://www.rcsb.org/structure/4Z6H
4UIU	TVU	476.11	COc1ccc(-c2cn(C)c(=O)c3cc(C(=O)NC4CCS(=O)(=O)CC4)sc23)cc1OC	https://www.rcsb.org/structure/4UIU
4Z6I	4L3	461.23	Cc1cc(=O)n(C)c2cc(N3C(=O)CC[C@H](NC(=O)OC(C)(C)C)[C@H]3c3ccccc3)ccc12	https://www.rcsb.org/structure/4Z6I
5F2P	5TY	204.1	CN(C)c1ncc2c(=O)n(C)ccc2n1	https://www.rcsb.org/structure/5F2P
5F1L	5U2	344.4	CC1=CC(=CN(C1=O)C)c2cc(c(c(c2)OC)CN3CC(C3)O)OC	https://www.rcsb.org/structure/5F1L
5EU1	5SW	353.4	CN1C=C(c2ccncc2C1=O)c3cc(c(c(c3)OC)CN(C)C)OC	https://www.rcsb.org/structure/5EU1
5IGM	BMF	404.13	CCOC(=O)Nc1cc(-c2ccc(C)c(NS(C)(=O)=O)c2)nn2c(C)nnc12	https://www.rcsb.org/structure/5IGM
6BQA	67C	341.21	C.C.CCn1cc(-c2cccc(C(=O)N(C)C)c2)c2cc[nH]c2c1=O	https://www.rcsb.org/structure/6BQA
6Y7I	OF8	237.09	CC(=O)c1cc(-c2cccn2)c2ncccn12	https://www.rcsb.org/structure/6Y7I
5IGN	6B2	495.22	Cc1ccc([C@@H]2[C@@H](NS(=O)(=O)CC(C)C)CCC(=O)N2c2ccc3c(C)cc(=O)n(C)c3c2)cc1	https://www.rcsb.org/structure/5IGN
4NQN	Y1Z	514.18	Cc1ccccc1S(=O)(=O)Nc1cc(-c2nn3c(C)nnc3c3ccccc23)ccc1N1CCOCC1	https://www.rcsb.org/structure/4NQN
6Y7J	OF5	296.12	COc1ccc(OC)c(-c2cc(C(C)=O)n3cccnc23)c1	https://www.rcsb.org/structure/6Y7J
6Y7K	OEZ	363.16	COc1ccc(C(=O)NC2CCC2)cc1-c1cc(C(C)=O)n2cccnc12	https://www.rcsb.org/structure/6Y7K
5F1H	5U6	353.4	CN1C=C(c2ccncc2C1=O)c3cc(c(cc3OC)CN(C)C)OC	https://www.rcsb.org/structure/5F1H
4YYG	BTK	200.15	CCCC(=O)NCCCC[C@H](N)C=O	https://www.rcsb.org/structure/4YYG
6HM0	GBW	953.1	Cc1c(scn1)c2ccc(cc2)CNC(=O)[C@@H]3C[C@@H](CN3C(=O)[C@H](C(C)(C)C)NC(=O)COCCOCCN4CCN(CC4)Cc5c(cc(cc5OC)C6=CN(C(=O)c7c6ccnc7)C)OC)O	https://www.rcsb.org/structure/6HM0
6V0S	EAE	251.1	CCN1c2cc(OC)ccc2SC1CC(C)=O	https://www.rcsb.org/structure/6V0S
6Y7H	OFK	236.09	CC(=O)c1cc(-c2ccccc2)c2ncccn12	https://www.rcsb.org/structure/6Y7H
4UIV	XZB	483.5	CN1C=C(c2c(cc(s2)/C(=N/C3CCS(=O)(=O)CC3)/N)C1=O)c4cccc(c4)C(F)(F)F	https://www.rcsb.org/structure/4UIV
4UIW	H1B	497.5	CCN1C=C(c2c(cc(s2)/C(=N/C3CCS(=O)(=O)CC3)/N)C1=O)c4cccc(c4)C(F)(F)F	https://www.rcsb.org/structure/4UIW
6V1B	H1B	497.5	CCN1C=C(c2c(cc(s2)/C(=N/C3CCS(=O)(=O)CC3)/N)C1=O)c4cccc(c4)C(F)(F)F	https://www.rcsb.org/structure/6V1B
6YQW	82I	153.09	CNc1cnn(C)c(=O)c1C	https://www.rcsb.org/structure/6YQW
BRD9				
PDB ID	Ligand Name	Ligand MW	Ligand-smiles	URL
6V0Q	EAE	251.1	CCN1c2cc(OC)ccc2SC1CC(C)=O	https://www.rcsb.org/structure/6V0Q
6V1H	BMF	404.13	CCOC(=O)Nc1cc(-c2ccc(C)c(NS(C)(=O)=O)c2)nn2c(C)nnc12	https://www.rcsb.org/structure/6V1H

6V17	H1B	497.5	CCN1C=C(c2c(cc(s2)/C(=N/C3CCS(=O)(=O)CC3)/N)C1=O)c4cccc(c4)C(F)(F)F	https://www.rcsb.org/structure/6V17
6V1E	5SW	353.4	CN1C=C(c2ccncc2C1=O)c3cc(c(c(c3)OC)CN(C)C)OC	https://www.rcsb.org/structure/6V1E
5MQ1	5U6	353.4	CN1C=C(c2ccncc2C1=O)c3cc(c(cc3OC)CN(C)C)OC	https://www.rcsb.org/structure/5MQ1
6V16	LIG	333.15	CC(=O)c1cc(-c2cc(C(=O)NC3CC3)ccc2C)c2ncccn12	https://www.rcsb.org/structure/6V16

BRPF1

PDB ID	Ligand Name	Ligand MW	Ligand-smiles	URL
5ETB	5RO	173.08	CC(=O)c1c[nH]c2c(C)cccc12	https://www.rcsb.org/structure/5ETB
5O4T	9KT	190.07	Cn1c(=O)c(=O)n(C)c2ccccc21	https://www.rcsb.org/structure/5O4T
5EV9	5SB	252.1	CC(=O)Nc1ccc(-c2cn[nH]c2)c2cccnc12	https://www.rcsb.org/structure/5EV9
5DYC	5GU	160.08	Bc1ccc2c(c1)NC(=O)CN2	https://www.rcsb.org/structure/5DYC
5DY7	5GT	216.05	O=C1CNc2ccc(C(F)(F)F)cc2N1	https://www.rcsb.org/structure/5DY7
5C89	4YT	241.12	CCc1nnc2cc(C)c3ccc(OC)cc3n12	https://www.rcsb.org/structure/5C89
5EVA	5S9	237.07	Cn1nccc1C(=O)Nc1ccc(F)cc1F	https://www.rcsb.org/structure/5EVA
5EPR	5QY	162.08	C[C@@H]1Nc2ccccc2NC1=O	https://www.rcsb.org/structure/5EPR
5EPS	5QX	162.08	CN1C(=O)CNc2ccccc21	https://www.rcsb.org/structure/5EPS
5C85	4YO	160.08	Bc1ccc2c(c1)NCC(=O)N2	https://www.rcsb.org/structure/5C85
5EWH	5SG	145.05	Oc1cccc2cnccc12	https://www.rcsb.org/structure/5EWH
5DYA	5GV	220.08	CC[C@H]1Nc2ccc(C(=O)O)cc2NC1=O	https://www.rcsb.org/structure/5DYA
5E3G	5JQ	186.02	O=C1NC2=NC(S)NC(=O)C2N1	https://www.rcsb.org/structure/5E3G
5C87	4YS	145.05	O=c1[nH]ccc2ccccc12	https://www.rcsb.org/structure/5C87
5T4U	12Q	159.07	Cn1c(=O)ccc2ccccc21	https://www.rcsb.org/structure/5T4U
5EWC	5SJ	234.1	CCOC(=O)c1ccc2c(c1)NC(=O)[C@@H](C)N2	https://www.rcsb.org/structure/5EWC
5EWV	5SN	197.1	Cc1cc2nnc(C)n2c2ccccc12	https://www.rcsb.org/structure/5EWV
5EWD	5SH	155.07	c1ccc(-c2ccncc2)cc1	https://www.rcsb.org/structure/5EWD
5EWW	5SK	255.15	CC[C@@H](C)Nc1nc2ccccc2n2c(C)nnc12	https://www.rcsb.org/structure/5EWW
5OWE	B0E	228.04	O=c1oc2cc(O)cc(O)c2c2ccccc12	https://www.rcsb.org/structure/5OWE
5D7X	XZ8	255.1	CC(=O)n1cc(-c2c(C)[nH][nH]c2=O)c2ccccc21	https://www.rcsb.org/structure/5D7X
5EQ1	BEA	189.04	Cc1cccc2sc3nncn3c12	https://www.rcsb.org/structure/5EQ1
5ETD	5RN	159.07	CC(=O)c1c[nH]c2ccccc12	https://www.rcsb.org/structure/5ETD
5OWB	AYW	164.05	Cc1cc(O)cc2c1C(=O)CO2	https://www.rcsb.org/structure/5OWB

ALK

PDB ID	Ligand Name	Ligand MW	Ligand-smiles	URL

7JYT	VRM	159.08	Cc1cc(-c2cccnc2)n[nH]1	https://www.rcsb.org/structure/7JYT
7JY4	W47	423.3	Cc1cc([nH]n1)c2cc(ccc2O[C@H]3C[C@@]3(CN)c4ccc(cc4F)F)C(F)(F)F	https://www.rcsb.org/structure/7JY4

DNA-gyrase

PDB ID	Ligand Name	Ligand MW	Ligand-smiles	URL
6KZV	E0F	389.4	CN(Cc1ccccc1NC(=O)C2=Cc3ccccc3NC2=O)C4CCCCC4	https://www.rcsb.org/structure/6KZV
6KZZ	E0R	337.11	CNc1cccc2cc(C(=O)Nc3ccc(C(=O)O)cc3)c(=O)[nH]c12	https://www.rcsb.org/structure/6KZZ

DPP-4

PDB ID	Ligand Name	Ligand MW	Ligand-smiles	URL
3CCC	7AC	296.7	Cn1cnc2c1c(c(c(c2)C#N)c3ccccc3Cl)CN	https://www.rcsb.org/structure/3CCC
3CCB	B2Y	183.1	NCc1ccccc1-c1ccccc1	https://www.rcsb.org/structure/3CCB

LPXC

PDB ID	Ligand Name	Ligand MW	Ligand-smiles	URL
7CIC	FZ0	234.1	c1ccc(cc(c1)OC(F)(F)F)NC(=O)CN	https://www.rcsb.org/structure/7CIC
7CI7	FY9	484.6	CS(=O)(=O)C[C@@H]([C@H](C(=O)N1CCC(CC1)Cc2ccc(cc2)C#Cc3ccc(cc3)CO)N)O	https://www.rcsb.org/structure/7CI7
7CID	FZ3	200.13	Cc1ccc(CCCn2ccnc2)cc1	https://www.rcsb.org/structure/7CID
7CI9	FYL	412.4	C[C@@H](c1nccn1Cc2cc(on2)C#Cc3ccc(cc3)OCC(CO)(CO)N)O	https://www.rcsb.org/structure/7CI9

M. tuberculosis InhA

PDB ID	Ligand Name	Ligand MW	Ligand-smiles	URL
6SQ5	LRW	226.12	C.C.FC(F)(F)c1ccccc1.O=CO.[HH]	https://www.rcsb.org/structure/6SQ5
6SQL	LTK	366.8	Cc1c2cc(ccc2sc1S(=O)(=O)Nc3cccc(c3)CN)Cl	https://www.rcsb.org/structure/6SQL

Notum Carboxylesterase

PDB ID	Ligand Name	Ligand MW	Ligand-smiles	URL
6ZVL	QR2	244.05	Cc1ccc(-c2n[nH]c(=O)o2)cc1C(F)(F)F	https://www.rcsb.org/structure/6ZVL
6ZUV	B1J	189.09	Cc1ccc(-n2cc(CO)nn2)cc1	https://www.rcsb.org/structure/6ZUV
6YV2	PUE	191	c1ccc(cc1)N2CC[C@H](C2)C(=O)O	https://www.rcsb.org/structure/6YV2
6YSK	PJK	273.1	Cc1ccc(N2CC[C@H](C(=O)O)C2)cc1C(F)(F)F	https://www.rcsb.org/structure/6YSK

PIM-1

PDB ID	Ligand Name	Ligand MW	Ligand-smiles	URL
3VBY	0FR	211.06	O=C(c1ccco1)c1c[nH]c2ccccc12	https://www.rcsb.org/structure/3VBY

1YXV	LI6	191.06	Cn1c(=O)c(O)c(O)c2ccccc21	https://www.rcsb.org/structure/1YXV
5KGD	6SL	195.08	c1cncc(-c2nc3ccccc3[nH]2)c1	https://www.rcsb.org/structure/5KGD
3UIX	Q17	294.3	c1ccc2c(c1)c(nc(n2)c3ccccc3O)NCCCN	https://www.rcsb.org/structure/3UIX
5KGE	6SN	187.11	Cc1ccc(-c2cc(N)n[nH]2)cc1C	https://www.rcsb.org/structure/5KGE
5N50	8MN	196.07	Cc1ccc(SCC(=O)NN)cc1	https://www.rcsb.org/structure/5N50
4MTA	2D2	202.06	Cc1oc(-c2ccccc2)cc1C(=O)O	https://www.rcsb.org/structure/4MTA
3VBV	0FK	188.06	NC(=O)c1ccc2cccc(O)c2n1	https://www.rcsb.org/structure/3VBV
4MBI	26K	271.3	CN(C)CCNc1ccn2c(n1)c(cn2)c3c[nH]nc3	https://www.rcsb.org/structure/4MBI
5N4X	8MT	166.05	Bc1cc(C(=O)NN)sc1B	https://www.rcsb.org/structure/5N4X
3VBX	0FO	174.05	Bc1ccc2oc(=O)cc(O)c2c1	https://www.rcsb.org/structure/3VBX
5N5L	8NZ	173.06	Bc1ccc2[nH]cc(C(=O)O)c2c1	https://www.rcsb.org/structure/5N5L
5N4Z	8MK	172.15	C.C.CC=O.Oc1ccccc1.[HH]	https://www.rcsb.org/structure/5N4Z
3JPV	1DR	234.08	O=Cc1c[nH]c2c1ccc1c3ccccc3[nH]c12	https://www.rcsb.org/structure/3JPV
5DHJ	5E5	210.09	Cc1n[nH]c2cnc(-c3cccnc3)cc12	https://www.rcsb.org/structure/5DHJ
3JYA	LWG	230.05	Cc1ccc(C)c2c1sc1c(=O)[nH]cnc12	https://www.rcsb.org/structure/3JYA
3C4E	C4E	209.1	c1ccc(Nc2c[nH]c3ncccc23)cc1	https://www.rcsb.org/structure/3C4E
3VBT	0F9	174.08	Cc1ccc(O)c(-c2cc[nH]n2)c1	https://www.rcsb.org/structure/3VBT
5NDT	8UB	160.06	Cc1nc2ccccc2[nH]c1=O	https://www.rcsb.org/structure/5NDT
5N4U	8MZ	232.04	Nc1nc(-c2ccc3[nH]c(=O)[nH]c3c2)cs1	https://www.rcsb.org/structure/5N4U
5N4V	8MW	250.08	Cc1c(C(=O)O)sc2c1C(C)N=C(C1CC1)N2	https://www.rcsb.org/structure/5N4V
3R00	UNJ	174.05	Bc1ccc2oc(C(=O)O)cc2c1	https://www.rcsb.org/structure/3R00
3VC4	0FS	277.04	CC1SC(=O)NC1=O.FC(F)(F)c1ccccc1	https://www.rcsb.org/structure/3VC4
1YXX	LI7	238.07	O=C1Nc2ccccc2/C1=N/c1ccc(O)cc1	https://www.rcsb.org/structure/1YXX
5N4R	8MQ	159.07	CC(=O)c1c[nH]c2ccccc12	https://www.rcsb.org/structure/5N4R
5KGG	6SO	194.6	c1cc2c(cc1Cl)c(c[nH]2)CCN	https://www.rcsb.org/structure/5KGG
2XIX	XIX	99.05	Nc1n[nH]c(N)n1	https://www.rcsb.org/structure/2XIX
2XIY	XIY	148.06	OCc1nc2ccccc2[nH]1	https://www.rcsb.org/structure/2XIY
4LL5	SK8	297.07	Fc1ccc(-c2nc3n(c2-c2ccncc2)CCS3)cc1	https://www.rcsb.org/structure/4LL5
5KGI	6SF	206	c1cc(c(cc1OCCN)Cl)Cl	https://www.rcsb.org/structure/5KGI
5N4N	8M8	206.06	Cc1c(C#N)sc(C2=NCNN2)c1C	https://www.rcsb.org/structure/5N4N
2XIZ	XIZ	159.13	C.CO.Cc1ccncc1.O	https://www.rcsb.org/structure/2XIZ
WRD-5				
PDB ID	Ligand Name	Ligand MW	Ligand-smiles	URL

6UHY	Q8G	244.12	O=C(O)c1ccc2c(c1)ncn2C1CCCCC1	https://www.rcsb.org/structure/6UHY
6UOZ	QF1	413.13	Bc1cc(C)c(O)c(S(=O)(=O)NC2=NC(S(C)(=O)=O)N(C3CCCC3)C2)c1	https://www.rcsb.org/structure/6UOZ

PDE-4

PDB ID	Ligand Name	Ligand MW	Ligand-smiles	URL
1Y2K	7DE	290.11	CCOC(=O)c1c(C)nn(-c2cccc([N+](=O)O)c2)c1C	https://www.rcsb.org/structure/1Y2K
1Y2B	DEE	168.09	CCOC(=O)c1c(C)n[nH]c1C	https://www.rcsb.org/structure/1Y2B

Carbonic Anhydrase II CAII

PDB ID	Ligand Name	Ligand MW	Ligand-smiles	URL
5FNI	YIH	244.05	Clc1ccc(OCc2n[n-]nn2)cc1Cl	https://www.rcsb.org/structure/5FNI
5FLS	6ZX	196.63	CC(=C/C(O)=O)\c1ccc(Cl)cc1	https://www.rcsb.org/structure/5FLS
5FNJ	YI6	180.2	CCOc1ccc(CC(O)=O)cc1	https://www.rcsb.org/structure/5FNJ
5FLT	VJJ	214.2	OC(=O)c1cccc(Oc2ccccc2)c1	https://www.rcsb.org/structure/5FLT
5FNK	5OO	217.1	OC(=O)/C=C/c1ccc(Cl)cc1Cl	https://www.rcsb.org/structure/5FNK
5FLO	J4K	210.6	Clc1ccc(OCc2[nH]nnn2)cc1	https://www.rcsb.org/structure/5FLO
5FLP	6J5	210.6	Clc1ccccc1OCc2[nH]nnn2	https://www.rcsb.org/structure/5FLP
5FNG	YIE	193.6	Clc1ccc(Cc2[n-]nnn2)cc1	https://www.rcsb.org/structure/5FNG
5FLQ	IO2	242.2	OC(=O)Cc1ccc(OCc2ccccc2)cc1	https://www.rcsb.org/structure/5FLQ
5FLR	XCZ	173.2	Cc1ccnc(n1)[S](N)(=O)=O	https://www.rcsb.org/structure/5FLR
5EH5	XCZ	173.2	Cc1ccnc(n1)[S](N)(=O)=O	https://www.rcsb.org/structure/5EH5
5EHV	5ON	312.3	OC(=O)Cc1cccc(COc2cccc(/C=C/C(O)=O)c2)c1	https://www.rcsb.org/structure/5EHV
5EH7	5O5	245	Clc1ccc(OCc2[nH]nnn2)cc1Cl	https://www.rcsb.org/structure/5EH7
5EHW	5OO	217	OC(=O)/C=C/c1ccc(Cl)cc1Cl	https://www.rcsb.org/structure/5EHW
5EH8	5O6	192.2	COc1ccc(cc1)\C(C)=C\C(O)=O	https://www.rcsb.org/structure/5EH8
5FNL	5ON	312.3	OC(=O)Cc1cccc(COc2cccc(/C=C/C(O)=O)c2)c1	https://www.rcsb.org/structure/5FNL
5FNM	5O6	192.2	COc1ccc(cc1)\C(C)=C\C(O)=O	https://www.rcsb.org/structure/5FNM

Adenosine A1 receptor

PDB ID	Ligand Name	Ligand MW	Ligand-smiles	URL
7LD3	XTD	449.7	Nc1scc(c2cc(cc(c2)C(F)(F)F)C(F)(F)F)c1C(=O)c3ccc(Cl)cc3	https://www.rcsb.org/structure/7LD3
6D9H	ADN	267.2	Nc1ncnc2n(cnc12)[C@@H]3O[C@H](CO)[C@@H](O)[C@H]3O	https://www.rcsb.org/structure/6D9H
7LD4	ADN	267.2	Nc1ncnc2n(cnc12)[C@@H]3O[C@H](CO)[C@@H](O)[C@H]3O	https://www.rcsb.org/structure/7LD4

Adenosine A2 Receptor

PDB ID	Ligand Name	Ligand MW	Ligand-smiles	URL
3REY	XAC	428.4	CCCN1C(=O)N(CCC)c2nc([nH]c2C1=O)c3ccc(OCC(=O)NCCN)cc3	https://www.rcsb.org/structure/3REY
3PWH	ZMA	337.3	Nc1nc(NCCc2ccc(O)cc2)nc3nc(nn13)c4occc4	https://www.rcsb.org/structure/3PWH
5G53	NEC	308.2	CCNC(=O)[C@H]1O[C@H]([C@H](O)[C@@H]1O)n2cnc3c(N)ncnc23	https://www.rcsb.org/structure/5G53
3RFM	CFF	194.1	Cn1cnc2N(C)C(=O)N(C)C(=O)c12	https://www.rcsb.org/structure/3RFM

Beta 1 Adrenergic Receptor

PDB ID	Ligand Name	Ligand MW	Ligand-smiles	URL
2Y01	Y00	301.3	C[C@H](CCc1ccc(cc1)O)NCCc2ccc(c(c2)O)O	https://www.rcsb.org/structure/2Y01
2Y02	WHJ	368.4	COc1ccc(C[C@@H](C)NC[C@H](O)c2ccc(O)c3NC(=O)C=Cc23)cc1	https://www.rcsb.org/structure/2Y02
2Y03	5FW	211.2	CC(C)NC[C@H](O)c1ccc(O)c(O)c1	https://www.rcsb.org/structure/2Y03
2Y04	68H	239.3	CC(C)(C)NC[C@H](O)c1ccc(O)c(CO)c1	https://www.rcsb.org/structure/2Y04
2YCW	CAU	298.3	CC(C)NC[C@H](O)COc1cccc2[nH]c3ccccc3c12	https://www.rcsb.org/structure/2YCW
2YCZ	I32	413.2	CC(C)(C)NC[C@H](O)COc1cccc2[nH]c(C#N)c(I)c12	https://www.rcsb.org/structure/2YCZ
3ZPQ	XF5	201.2	C1CN(CCN1)c2cccc3[nH]ccc23	https://www.rcsb.org/structure/3ZPQ
3ZPR	3WC	227.3	Cc1cc(nc2ccccc12)N3CCNCC3	https://www.rcsb.org/structure/3ZPR
4AMI	G89	363.4	CC(C)(Cc1c[nH]c2ccccc12)NC[C@H](O)COc3ccccc3C#N	https://www.rcsb.org/structure/4AMI
4AMJ	CVD	406.4	COc1ccccc1OCCNC[C@H](O)COc2cccc3[nH]c4ccccc4c23	https://www.rcsb.org/structure/4AMJ
4BVN	P32	287.3	CC(C)(C)NC[C@H](O)COc1cccc2N=C(Cc12)C#N	https://www.rcsb.org/structure/4BVN
5ABE	XQO	274.3	CCCCCN1[C@H](CO)[C@@H](O)[C@@H](O)[C@@H]1CC(=O)NC	https://www.rcsb.org/structure/5ABE
5F8U	P32	287.3	CC(C)(C)NC[C@H](O)COc1cccc2N=C(Cc12)C#N	https://www.rcsb.org/structure/5F8U

Beta 2 Adrenergic Receptor

PDB ID	Ligand Name	Ligand MW	Ligand-smiles	URL
4LDE	P0G	370.4	Cc1ccccc1CC(C)(C)NC[C@H](O)c2ccc(O)c3NC(=O)COc23	https://www.rcsb.org/structure/4LDE
4LDO	ALE	183.2	CNC[C@H](O)c1ccc(O)c(O)c1	https://www.rcsb.org/structure/4LDO
6N48	P0G	370.4	Cc1ccccc1CC(C)(C)NC[C@H](O)c2ccc(O)c3NC(=O)COc23	https://www.rcsb.org/structure/6N48
4LDL	XQC	317.3	CC(C)(Cc1ccc(O)cc1)NC[C@H](O)c2ccc(O)c(O)c2	https://www.rcsb.org/structure/4LDL
6MXT	K5Y	415.5	OCc1cc(ccc1O)[C@@H](O)CNCCCCCCOCCCCc2ccccc2	https://www.rcsb.org/structure/6MXT

CB1 Cannabinoid Receptor

PDB ID	Ligand Name	Ligand MW	Ligand-smiles	URL
7V3Z	9GF	376.5	CCCCCCC(C)(C)c1ccc([C@@H]2C[C@H](O)CC[C@H]2CCCO)c(O)c1	https://www.rcsb.org/structure/7V3Z
5XRA	8D3	449.4	CC1=CC[C@@H]2[C@@H](C1)c3c(cc(cc3OC2(C)C)C(C)(C)CCCCCCBr)O	https://www.rcsb.org/structure/5XRA

5XR8	8D0	445.6	CC(C)(CCCCCCN=C=S)c1cc(O)c2[C@@H]3C[C@H](CO)CC[C@H]3C(C)(C)Oc2c1	https://www.rcsb.org/structure/5XR8
Histamine H1 Receptor				
PDB ID	Ligand Name	Ligand MW	Ligand-smiles	URL
8X63	Y5E	285.3	COc1ccc(CN(CCN(C)C)c2ccccn2)cc1	https://www.rcsb.org/structure/8X63
8X64	Y5R	310.8	c1cc2c(nc1)C(=C3CCNCC3)c4ccc(cc4CC2)Cl	https://www.rcsb.org/structure/8X64
8X5Y	XB7	458.5	COc1ccc(CCN2CCC(CC2)Nc3nc4ccccc4n3Cc5ccc(F)cc5)cc1	https://www.rcsb.org/structure/8X5Y
MGlu-5 Receptor				
PDB ID	Ligand Name	Ligand MW	Ligand-smiles	URL
5CGC	51D	299.6	Fc1c(Cl)cc(cc1c2cc(ncn2)n3cccn3)C#N	https://www.rcsb.org/structure/5CGC
5CGD	51E	310.7	c1cc(ncc1F)c2cc(ncn2)c3cc(cc(c3)Cl)C#N	https://www.rcsb.org/structure/5CGD
BCL-xL BH3 domain Receptor				
PDB ID	Ligand Name	Ligand MW	Ligand-smiles	URL
6O0O	F3Q	710.81	Oc1ccc(cc1)N(C(=O)c2cc(n3CCCCc23)c4cc5OCOc5cc4C(=O)N6Cc7ccccc7C[C@H]6CN8CCOCC8)c9ccccc9	https://www.rcsb.org/structure/6O0O
6O0P	LBM	868.4	CC1(C)CCC(=C(C1)c2ccc(Cl)cc2)CN3CCN(CC3)c4ccc(C(=O)N[S](=O)(=O)c5ccc(NCC6CCOCC6)c(c5)[N+]([O-])=O)c(Oc7cnc8[nH]ccc8c7)c4	https://www.rcsb.org/structure/6O0P
6O0L	LBM	868.4	CC1(C)CCC(=C(C1)c2ccc(Cl)cc2)CN3CCN(CC3)c4ccc(C(=O)N[S](=O)(=O)c5ccc(NCC6CCOCC6)c(c5)[N+]([O-])=O)c(Oc7cnc8[nH]ccc8c7)c4	https://www.rcsb.org/structure/6O0L
Class A Beta-lactamase Receptor				
PDB ID	Ligand Name	Ligand MW	Ligand-smiles	URL
6OOF	R6Z	401.3	c1cc(cc(c1)NC(=O)c2cc(cc(c2)C(F)(F)F)c3[nH]nnn3)c4[nH]nnn4	https://www.rcsb.org/structure/6OOF
4UAA	3GK	373.2	FC(F)(F)c1cc2[nH]cnc2c(c1)C(=O)Nc3cccc(c3)c4n[nH]nn4	https://www.rcsb.org/structure/4UAA
4UA7	3GK	373.2	FC(F)(F)c1cc2[nH]cnc2c(c1)C(=O)Nc3cccc(c3)c4n[nH]nn4	https://www.rcsb.org/structure/4UA7
7U70	LW0	323.3	Fc1cccc(c1)C(=O)N[C@@H]2C[C@H](c3[nH]nnn3)c4ccccc24	https://www.rcsb.org/structure/7U70
6OOJ	J1X	399.3	FC(F)(F)c1cc(cc(c1)C(=O)Nc2cccc(c2)c3[nH]nnn3)n4cccn4	https://www.rcsb.org/structure/6OOJ
6OOK	MZV	410.3	FC(F)(F)c1cc(cc(c1)c2ccccn2)C(=O)Nc3cccc(c3)c4[nH]nnn4	https://www.rcsb.org/structure/6OOK
MDM2 Receptor				
PDB ID	Ligand Name	Ligand MW	Ligand-smiles	URL
7BIR	TUZ	558.4	CC(C)(O)c1cc(F)c2c(c1)C(=O)N(Cc3ccc(Cl)cn3)[C@@]2(OCC4(CC4)C(N)=O)c5ccc(Cl)cc5	https://www.rcsb.org/structure/7BIR

4HG7	NUT	581.4	COc1ccc(c(OC(C)C)c1)C2=N[C@H]([C@H](N2C(=O)N3CCNC(=O)C3)c4ccc(Cl)cc4)c5ccc(Cl)cc5	https://www.rcsb.org/structure/4HG7
7BMG	U3Z	516	CC(C)(O)c1ccc2c(c1)C(=O)N(Cc3ccc(cc3)C#C)[C@@]2(OCC4(CO)CC4)c5ccc(Cl)cc5	https://www.rcsb.org/structure/7BMG
7BIT	TV5	531.4	CC(C)(O)c1cc(F)c2c(c1)C(=O)N(Cc3ccc(Cl)cn3)[C@@]2(OCC4(O)CC4)c5ccc(Cl)cc5	https://www.rcsb.org/structure/7BIT
7BIV	TUW	535.9	CC(C)(c1cc2c(c(c1)F)[C@@](N(C2=O)Cc3ccc(cn3)C#N)(c4ccc(cc4)Cl)OCC5(CC5)CO)O	https://www.rcsb.org/structure/7BIV
7BJ6	TVK	545.4	CC(C)(O)c1cc(F)c2c(c1)C(=O)N(Cc3ccc(Cl)cn3)[C@@]2(OCC4(CO)CC4)c5ccc(Cl)cc5	https://www.rcsb.org/structure/7BJ6
KRAS_PDEd Receptor				
PDB ID	Ligand Name	Ligand MW	Ligand-smiles	URL
5ML8	V98	616.2	Clc1ccc(CN(C2CCCC2)[S](=O)(=O)c3ccc(cc3)[S](=O)(=O)N(CC4CCNCC4)Cc5ccccc5)cc1	https://www.rcsb.org/structure/5ML8
4JVF	17X	638.8	O=C(OC[C@H](C1CCNCC1)n2c3ccccc3nc2c4ccccc4)C5CCN(CC5)c6nc7ccccc7n6Cc8ccccc8	https://www.rcsb.org/structure/4JVF
5ML3	DL3	647.2	CNc1nccc(n1)CN(CC2CCNCC2)S(=O)(=O)c3ccc(cc3)S(=O)(=O)N(Cc4ccc(cc4)Cl)C5CCCC5	https://www.rcsb.org/structure/5ML3
5ML4	RRQ	689.2	CNc1cc(CN(CC2CCNCC2)[S](=O)(=O)c3ccc(cc3)[S](=O)(=O)N(Cc4ccc(Cl)cc4)C5CCCC5)ccc1C(O)=O	https://www.rcsb.org/structure/5ML4
4JVB	1M0	560.6	C=CC[C@H](COc1ccc(cc1)c2nc3ccccc3n2Cc4ccccc4)n5c6ccccc6nc5c7ccccc7	https://www.rcsb.org/structure/4JVB
5ML6	9GD	675.25	c1cc(ccc1CN(C2CCCC2)S(=O)(=O)c3ccc(cc3)S(=O)(=O)N(Cc4ccc(c(c4)N)C(=O)O)CC5CCNCC5)Cl	https://www.rcsb.org/structure/5ML6
4JV6	18F	284.3	C(n1c2ccccc2nc1c3ccccc3)c4ccccc4	https://www.rcsb.org/structure/4JV6
4JV8	1M1	298.3	N1[C@H](n2c3ccccc3nc2c4ccccc14)c5ccccn5	https://www.rcsb.org/structure/4JV8
5ML2	NH6	519	Clc1ccc(CN(C2CCCC2)[S](=O)(=O)c3ccc(cc3)[S](=O)(=O)NCc4ccccc4)cc1	https://www.rcsb.org/structure/5ML2
Tyrosine Phosphatase 1B (PTP1B) Receptor				
PDB ID	Ligand Name	Ligand MW	Ligand-smiles	URL
7FRF	6SU	229.2	COC(=O)c1cccc(N[S](C)(=O)=O)c1	https://www.rcsb.org/structure/7FRF
7FQT	JJY	215.2	O=C1CCCc2nc3CCCC(=O)c3cc12	https://www.rcsb.org/structure/7FQT
7FQU	JMV	186.5	OC(=O)COc1cccc(Cl)c1	https://www.rcsb.org/structure/7FQU
7FQN	JKA	235.3	C[S](=O)(=O)N1C[C@H](O)[C@H]2C[C@@H]1CC[C@H]2O	https://www.rcsb.org/structure/7FQN
7FQO	WUW	263.3	CO[C@H]1CC[C@@H]2C[C@H]1[C@H](CN2[S](C)(=O)=O)OC	https://www.rcsb.org/structure/7FQO
7FQP	JN1	238.2	O=C1C[N@@]2C[C@H](N1)c3ccccc3[S]2(=O)=O	https://www.rcsb.org/structure/7FQP
7FQQ	JKJ	249.3	COC[C@H]1N(C2CCC1(O)CC2)[S](C)(=O)=O	https://www.rcsb.org/structure/7FQQ
7FRO	JG4	150.2	[nH]1ccnc1c2sccc2	https://www.rcsb.org/structure/7FRO
7FRP	JL4	225.2	Cc1scc(n1)c2sc(cc2)C(O)=O	https://www.rcsb.org/structure/7FRP
7FRQ	JLG	211.26	OC(=O)c1csc(n1)c2sccc2	https://www.rcsb.org/structure/7FRQ
7FQM	JKG	219.3	Oc1ccc(CNCc2sccc2)cc1	https://www.rcsb.org/structure/7FQM

3EAX	LZP	646.6	O=C(CC(=O)c1cccc(OCc2ccccc2)c1)C(=O)N3CCN(CC3)C(=O)C(=O)CC(=O)c4cccc(OCc5ccccc5)c4	https://www.rcsb.org/structure/3EAX
7FRR	O1J	166.1	OC(=O)COCc1ccccc1	https://www.rcsb.org/structure/7FRR
7FRK	JFP	170.2	CCC(=O)Nc1scc(C)n1	https://www.rcsb.org/structure/7FRK
7FRL	JHD	167.2	COc1ccc(CN)cc1OC	https://www.rcsb.org/structure/7FRL
7FQZ	WV0	151.1	Fc1ccc2NC(=O)Cc2c1	https://www.rcsb.org/structure/7FQZ
7FRM	JGG	245.2	O=C(NCc1ccc(cc1)C#N)N2CCOCC2	https://www.rcsb.org/structure/7FRM
3EB1	LZQ	387.42	OC(=O)C(=O)CC(=O)c1cccc(c1)N(Cc2ccccc2)Cc3ccccc3	https://www.rcsb.org/structure/3EB1
7FRN	JO1	221.2	Cn1nccc1C(=O)NCc2sccc2	https://www.rcsb.org/structure/7FRN
7FRG	GV1	207.2	CN(C)c1ncnc2sc(C)c(C)c12	https://www.rcsb.org/structure/7FRG
7FRH	JGD	122.1	CN(C)c1ccncc1	https://www.rcsb.org/structure/7FRH
7FQV	JGD	122.1	CN(C)c1ccncc1	https://www.rcsb.org/structure/7FQV
7FRI	JHJ	243.2	COc1ccc(NC(=O)Nc2ccncc2)cc1	https://www.rcsb.org/structure/7FRI
7FQW	JG4	150.2	[nH]1ccnc1c2sccc2	https://www.rcsb.org/structure/7FQW
7FRJ	JFJ	155.6	CNCc1cccc(Cl)c1	https://www.rcsb.org/structure/7FRJ
7FQX	JHD	167.2	COc1ccc(CN)cc1OC	https://www.rcsb.org/structure/7FQX
7FQY	GRY	214.2	Cc1cc(C)nc(Nc2ccc(N)cc2)n1	https://www.rcsb.org/structure/7FQY
7FQR	JPD	243.2	CC[C@H](O)[C@@H]1N(C2CCC1(O)CC2)C(=O)OC	https://www.rcsb.org/structure/7FQR
7FQS	JPV	228.2	O[C@H]1CN[C@@H]2C[C@H]1c3[nH]c4ccccc4c3C2	https://www.rcsb.org/structure/7FQS
Proteasome β5 subunit (PSMB5) Receptor				
PDB ID	Ligand Name	Ligand MW	Ligand-smiles	URL
6QM7	410.4	229.2	c1cc(c(cc1NC(=O)N2CCCC2)c3ccn4c(cnc4n3)N5CCOCC5)F	https://www.rcsb.org/structure/6QM7

Supplementary Notes

Note 1: FMOPhore analysis per system

In the following section data is represented (per system) in the following order for figures format.

- **FP-score plots (quadrant matrix):** for apo-structure scanning and holo-complexes analysis per system. Normalization of Percentage of Interaction (PI%) (x-axis) and Normalization of Interaction Energy (ΔE) (y-axis). Vertical line intersecting the x-axis is the mean of percentage of interaction energy (PI mean) values, and horizontal line intersecting the y-axis is the mean of interaction energy (ΔE mean). Top-right red box ➔ Highly prioritized binding site residue. Top-left yellow box ➔ Binding site residue with highly favorable interaction energy but low frequency of interaction. Bottom-right green-box ➔ Binding site residue with high frequency of interaction but less binding affinity. Bottom-left blue box ➔ Least prioritized binding site residue.
- ***2D-FMOPhore heatmap****: The binding site residues on the bottom x-axis, and the percentage of interaction (%) on the top x-axis. PDB-IDs representing the ligands of the complex (PDB-D_Ligand-name_Chain-ID_Ligand-Number) on the y-axis (left side), and the color code of each cell corresponds to the interaction energy values y-axis (right side), (kcal/mol) between the function group on the ligands and the binding site residue.*
- ***PIEDA plots at least 2 PDB-ID per system: Bar-chart** showing Pair interaction energy decomposition analysis with interaction bond type and energy per function group versus binding site residues.*

Anaplastic Lymphoma Kinase (ALK)

FP-score plot

Apo-structure

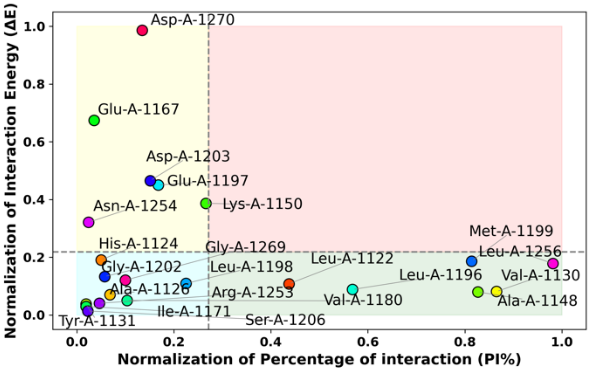

Holo-complexes

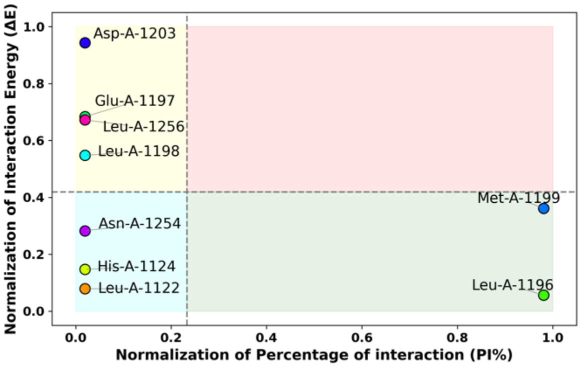

2D-FMOPhore heatmap

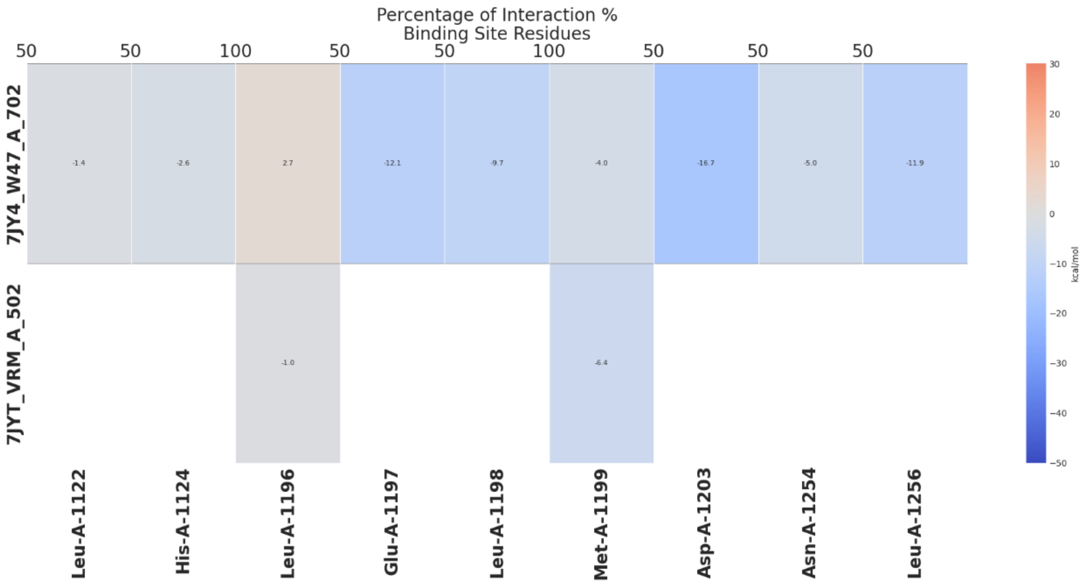

Aurora-A Kinase

FP-score plot

Apo-structure

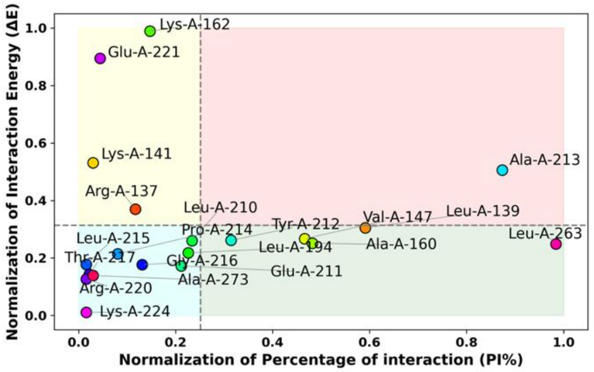

Holo-complexes

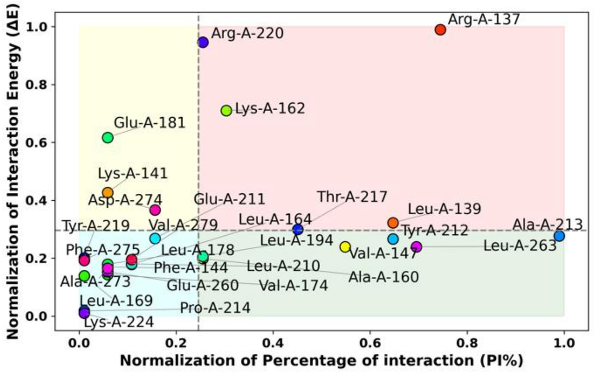

2D-FMOPhore heatmap

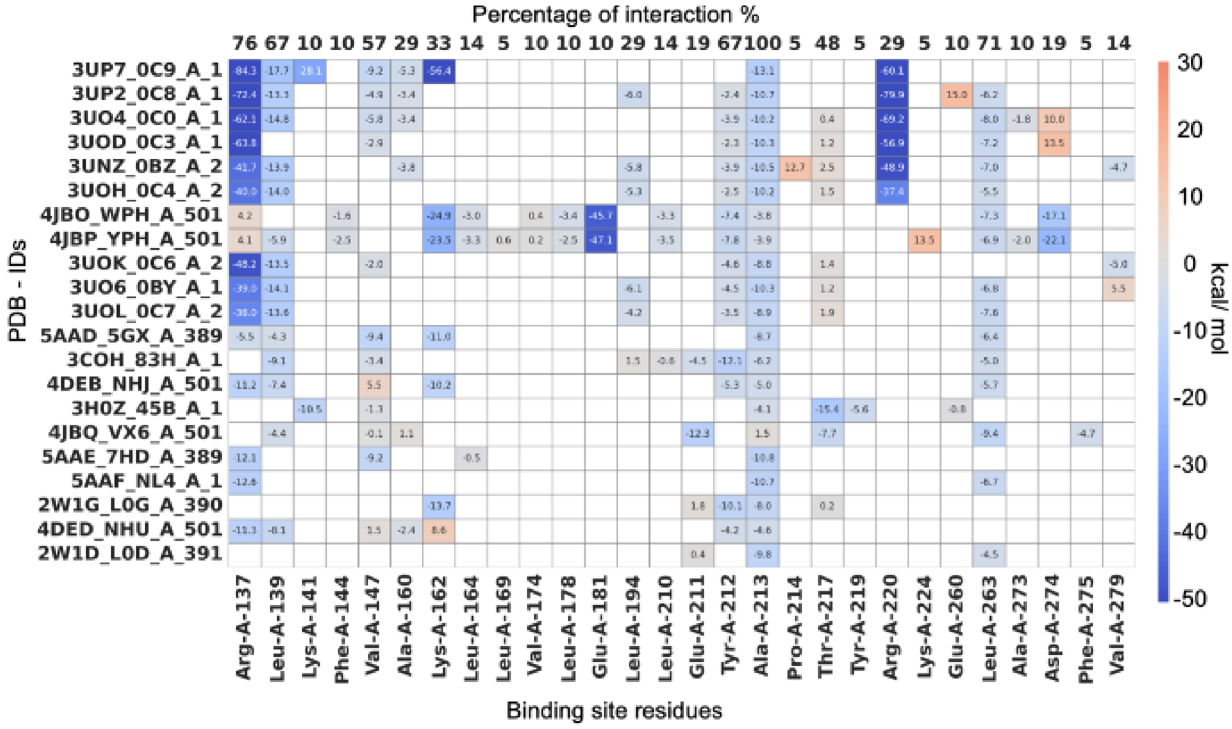

Bromodomain-containing protein (BRD-1)

FP-score plot

Apo-structure

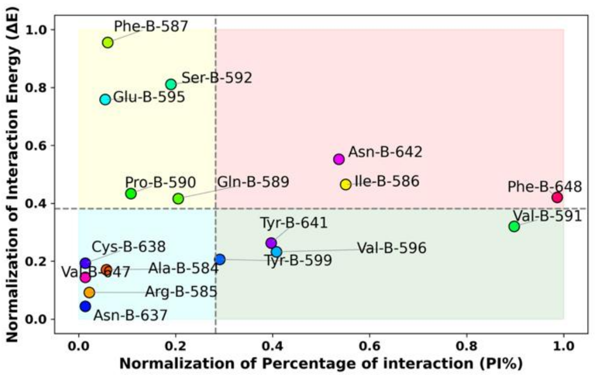

Holo-complexes

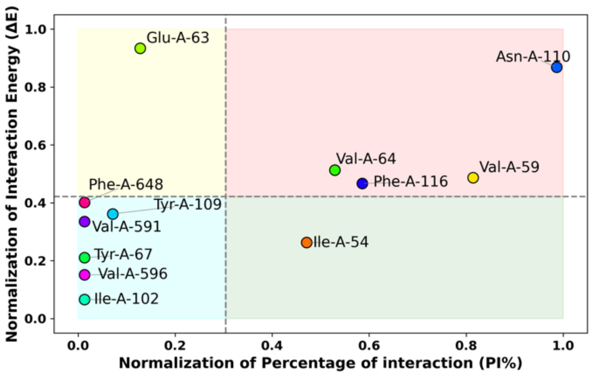

2D-FMOPhore heatmap

	Ile-A-54	Val-A-59	Glu-A-63	Val-A-64	Tyr-A-67	Ile-A-102	Tyr-A-109	Asn-A-110	Phe-A-116	Val-A-591	Val-A-596	Phe-A-648
	39	65	13	43	4	4	9	78	48	4	4	4
5PON_8RA_A_201	-0.4		-6.5	-6.0				-11.0				
5POB_8TD_A_201			-3.8					-11.9	-6.3			
5PO8_8SY_A_201		-5.3					-4.7	-11.0				
5POO_8TS_A_202	-0.2	-3.5		-4.0				-11.7				
5POJ_8RV_A_203	-0.1	-1.0	-13.4						-3.1			
5PO9_8T7_A_201	-1.9	-6.6		-7.0				-2.0				
5POE_8T1_A_203		-2.4						-8.0	-6.1			
5POH_8TP_A_203		1.0		-3.0				-9.6	-4.6			
5POT_8U4_A_203	-0.1	0.7		-2.1				-6.4	-5.3			
5PO7_8SV_A_201				-1.9				-10.3				
3RCW_MB3_A_1										-4.3	-1.5	-5.3
5PP0_8UD_A_204				-4.6				-2.6	-3.2			
5POC_8TG_A_203		0.2						-4.5	-5.9			
5POU_8U7_A_203	1.5	2.6		-0.3				-12.4	-0.3			
5POW_8UA_A_204		-0.6		-2.7				-5.1				
5POS_8U1_A_204		-0.7		-4.0					-3.3			
5AMF_TWL_A_143	-2.9	-5.0										
5PO0_8S7_A_205	-3.2								-3.9			
5POI_8RY_A_203								-6.6				
5POX_8UG_A_203		-1.1			-2.4	-0.2		-1.8				
5POQ_8TY_A_203		3.6						-8.7				
5POR_PW3_A_203		-0.3						-4.6				
5PO6_8SS_A_203	-0.5						1.4	-0.2	-5.1			

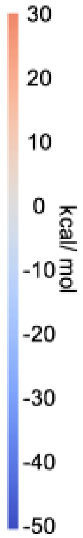

Bromodomain-containing protein (BRD-2)

FP-score plot

Apo-structure

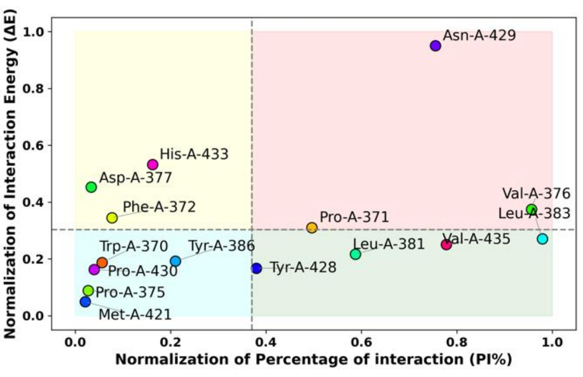

Holo-complexes

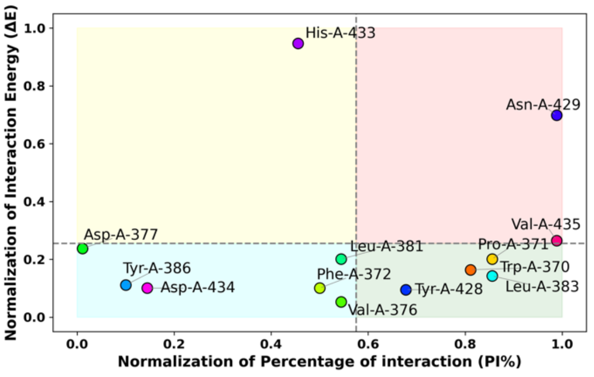

2D-FMOPhore heatmap

	Trp-A-370	Pro-A-371	Phe-A-372	Val-A-376	Asp-A-377	Leu-A-381	Leu-A-383	Tyr-A-386	Tyr-A-428	Asn-A-429	His-A-433	Asp-A-434	Val-A-435
	80	84	52	56	8	56	84	16	68	96	48	20	96
6E6J_HWV_A_501	-8.3	-2.6			-10.2	-9.5			-4.7	-18.5	-6.6		-5.6
5IG6_6B3_A_505		-4.3		-6.0		-8.1	-8.4	-3.5		-11.5	-23.6		
6VIY_QYY_A_501	-8.8	-2.8				-9.0	-5.0		-4.8	-18.9	-6.4		-5.8
3ONI_JQ1_A_1	-6.8	-3.8	-6.9	-3.8			-5.0		-4.3	-8.9	-7.3		-10.7
4UYG_73B_A_456	-8.0		-3.0	-4.6		-9.4	-2.6		-6.0	-13.1			-8.9
6FFE_D7Q_A_505	-5.0	-9.5	-2.8				-4.1	-7.8	-7.5	-15.3		6.0	-8.7
6K04_CQF_A_501	-7.0	-5.2	-5.8	-5.9		-4.3	-0.6	-3.6		-11.2	-0.6		-7.7
5UEW_87D_A_501	-4.8	-5.9			-8.7	-7.1				-12.1	-3.5		-8.4
6FFF_D7H_A_503	-8.0	-4.2	-6.8				-1.1	-3.1	-2.5	-11.3		-5.4	-7.5
5N2L_8J2_A_504	-7.6		-7.6	-4.1		-3.9	-0.5		-2.6	-9.5		-7.6	-5.7
7NQJ_UME_A_501	-6.2					-4.7	-5.7		-4.1	-12.1	-6.8		-7.8
6DBC_G3J_A_508	-6.2	-6.0	-5.9	-4.7			-1.2		-3.1	-10.9	-1.7		-7.5
6MOA_JW4_A_501	-7.1	-5.1	-7.5	-6.1			-1.3		-2.6	-5.2	-3.1		-8.7
5EK9_5P4_A_501	-5.6	-4.3	-4.7	-4.2		-6.4	-1.3		-2.7	-8.4		-0.7	-7.7
5U6V_7WY_A_501	-7.2	-6.6	-5.1	-4.6		-4.9				-7.3			-9.5
4MR5_1K0_A_506		-4.0				-5.1	-5.9		-4.9	-15.5	-4.2		-5.2
6I81_H7E_A_503	-6.3	-2.5	-6.3			-4.2	-3.7			-12.9			-8.6
4J1P_1K0_A_501		-4.0		-6.3		-6.3	-5.7			-16.8			-5.0
6DDJ_G7V_A_501	-7.1	-5.5	-6.3	-4.2			-0.7		-2.8	-10.6			-5.9
7NQI_UM8_A_501	-4.7						-5.4		-5.3	-12.0		-6.1	-8.5
5BT5_2LO_A_505	-6.9	-5.3	-3.9	-6.7			-1.3		-2.8	-5.3			-8.5
7NQ8_UM5_A_504	-5.7	-1.1					-6.0		-4.9	-11.0	-5.1		-5.9
5DW1_5GD_A_500		-2.7		-4.9		-2.7	-2.5		-0.5	-12.1	1.3		-4.6
5XHK_LDR_A_504		-2.9		-4.3			-3.8			-9.8			-4.0
5XHK_2ME_A_503	-2.1	-1.1											-2.2

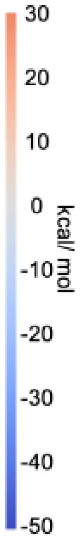

Bromodomain-containing protein (BRD-4)

FP-score plot

Apo-structure

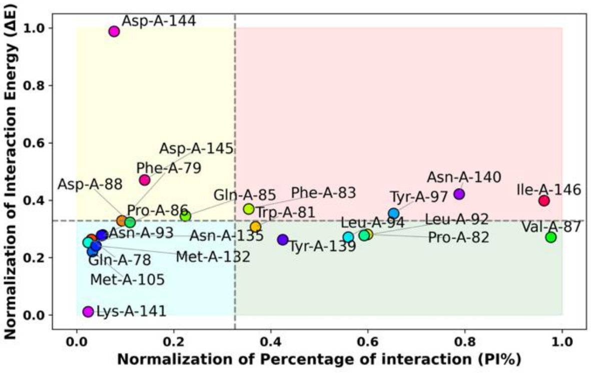

Holo-complexes

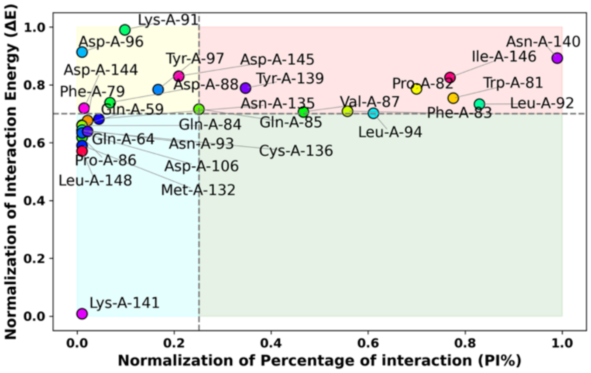

2D-FMOPhore heatmap

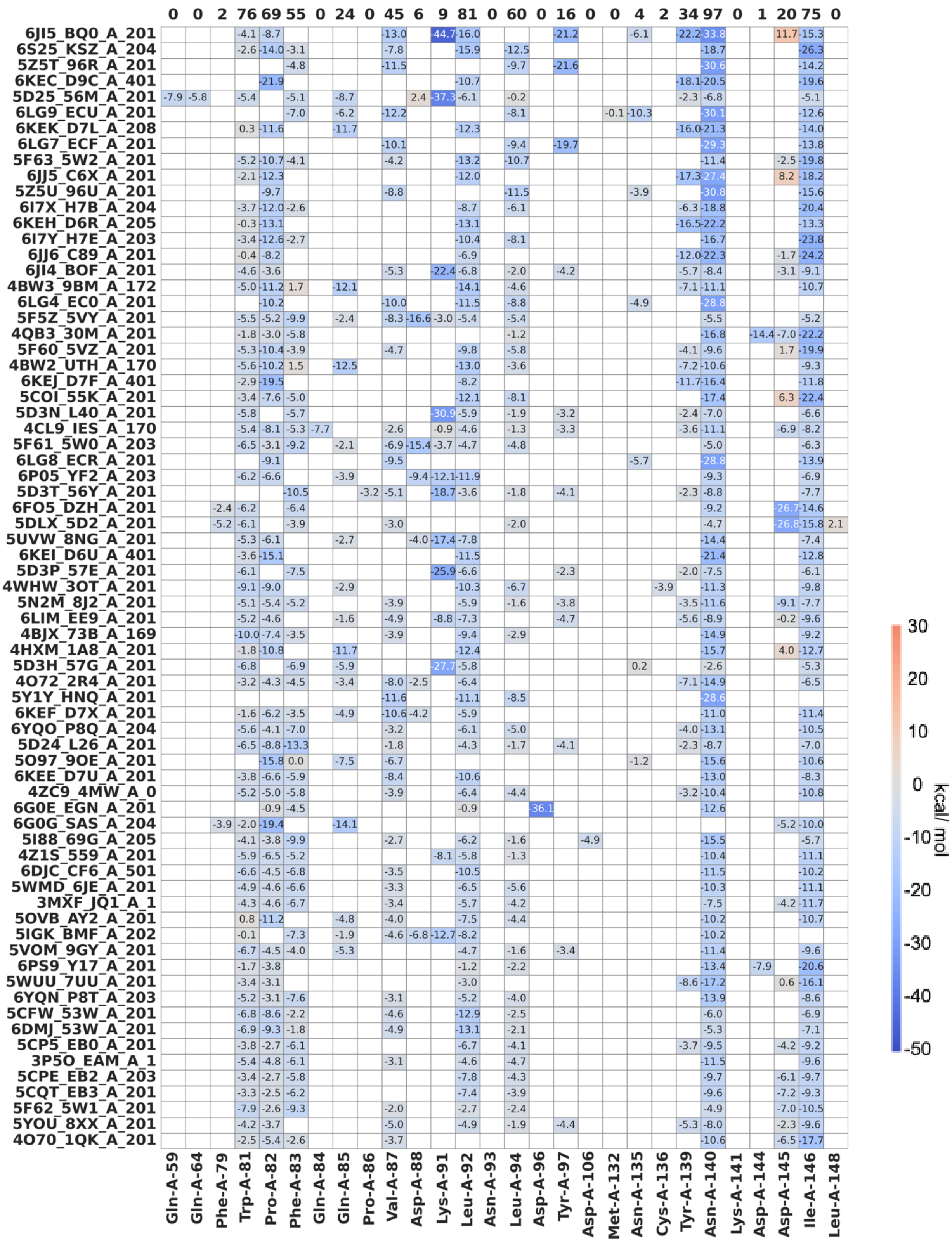

Continue next page

Continue from previous page

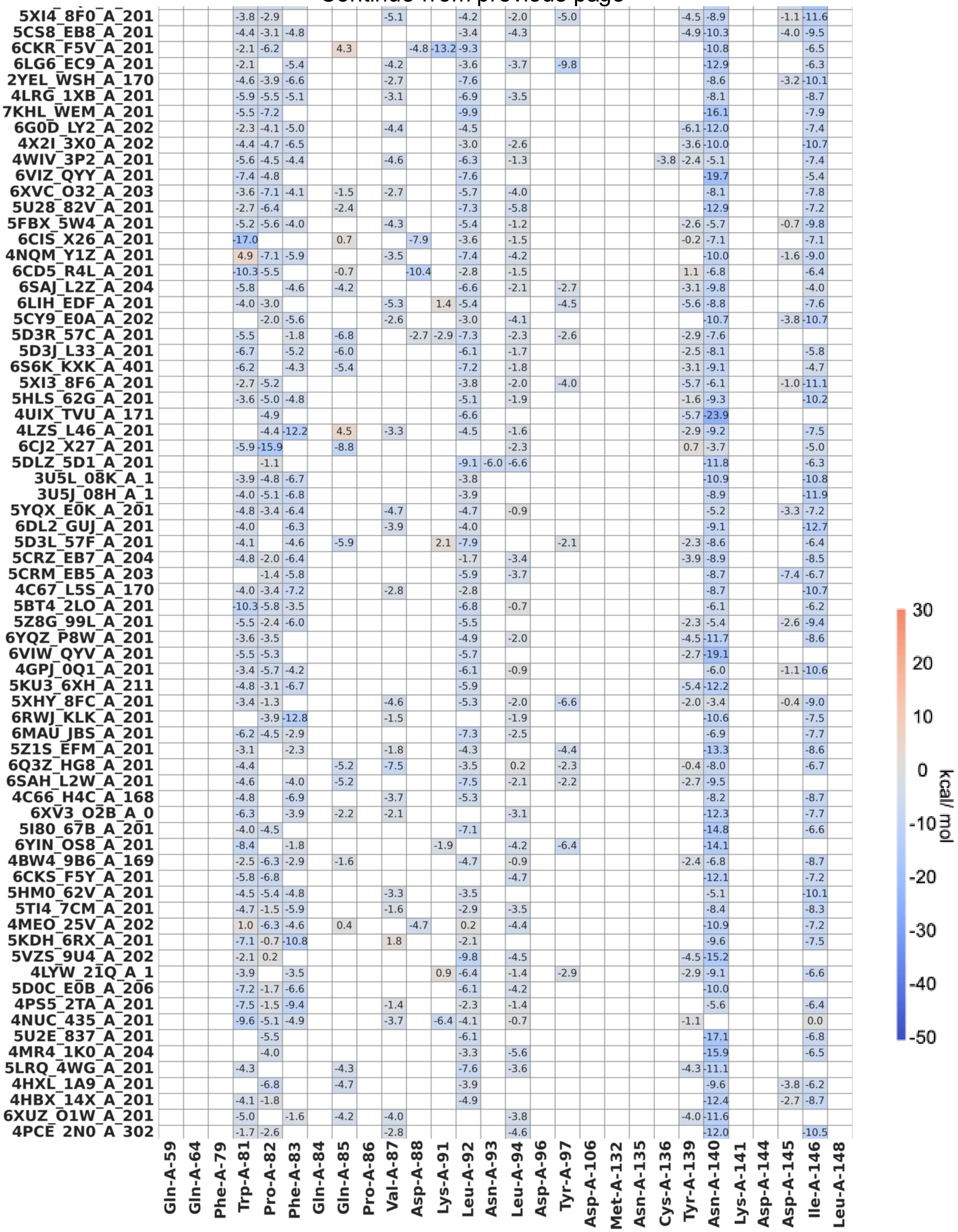

Continue next page

Continue from previous page

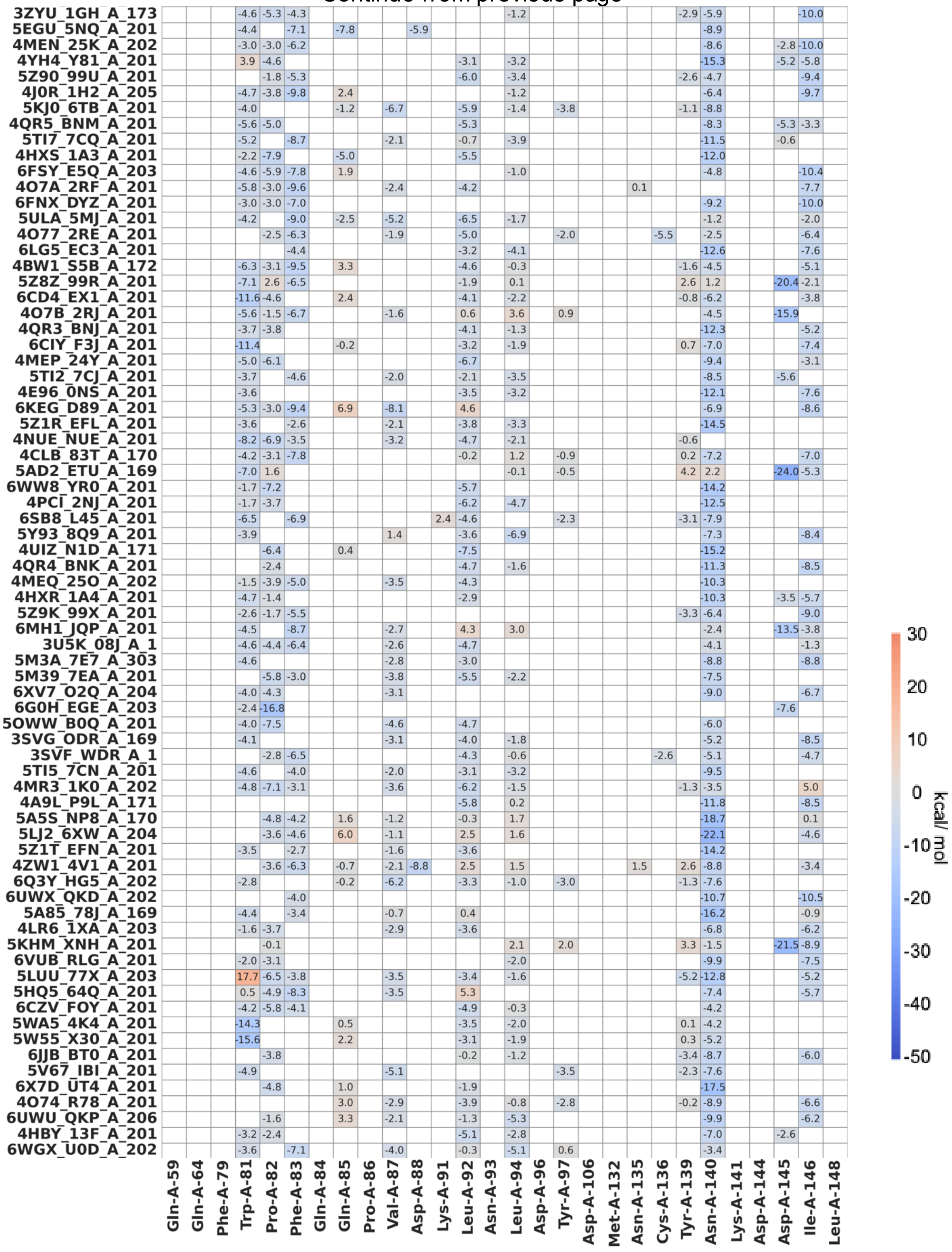

Continue next page

Continue from previous page

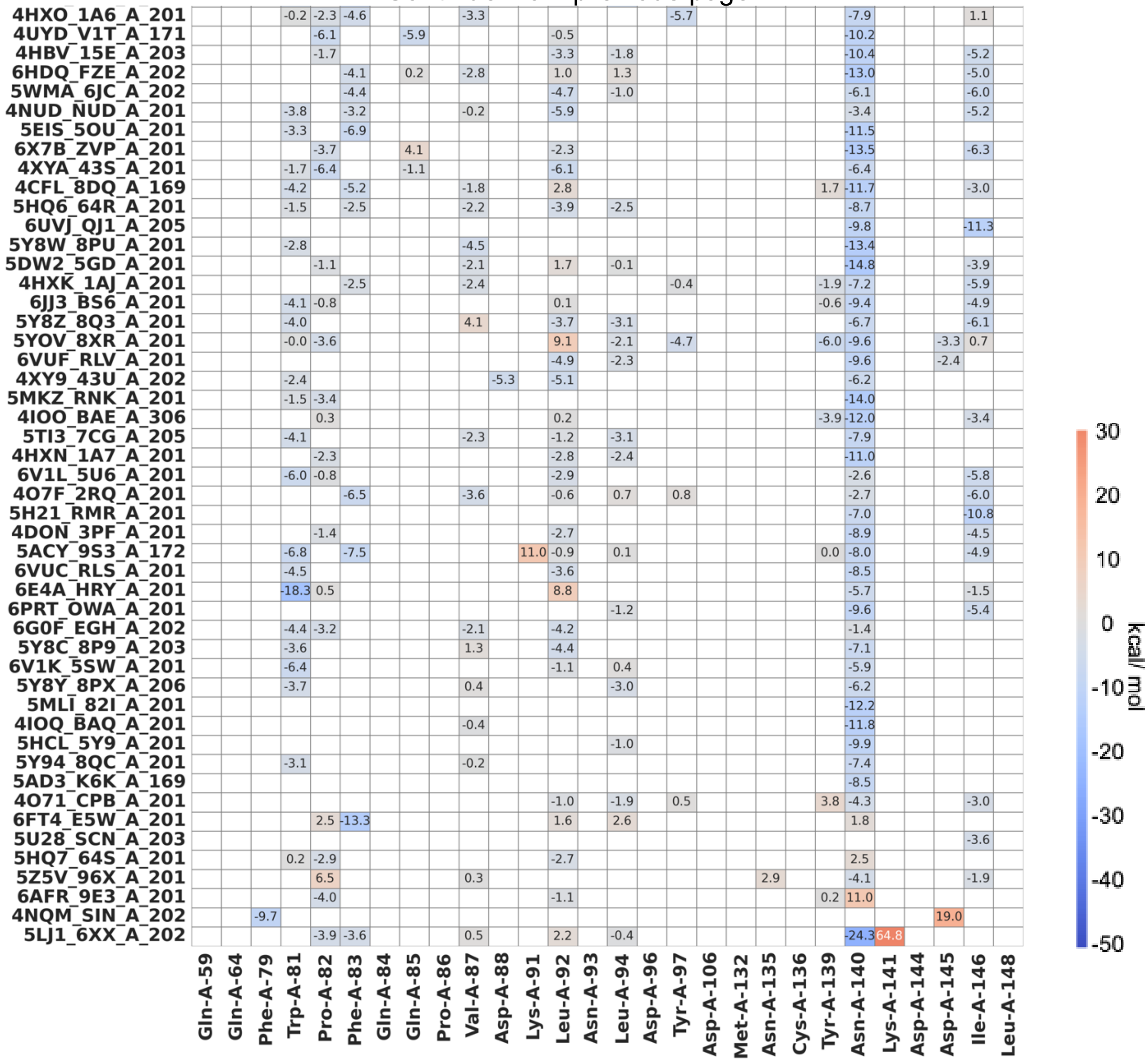

Bromodomain-containing protein (BRD-7)

FP-score plot

Apo-structure

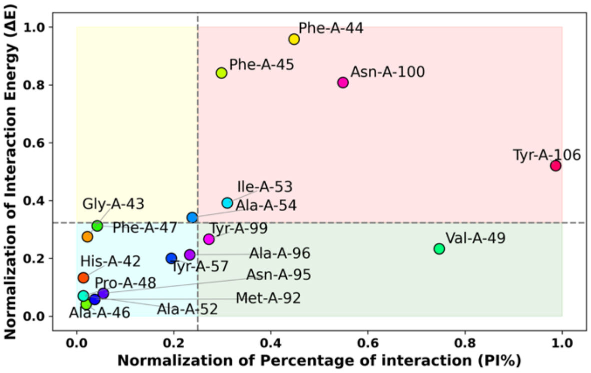

Holo-complexes

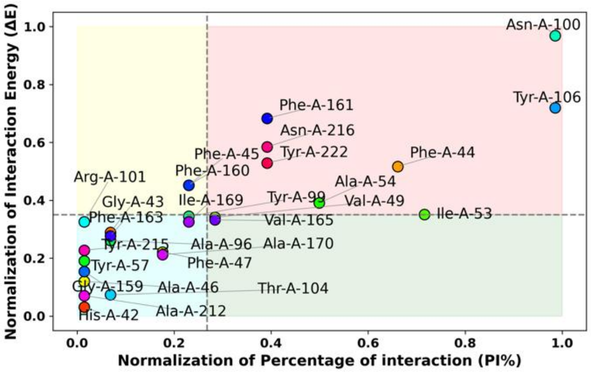

2D-FMOPhore heatmap

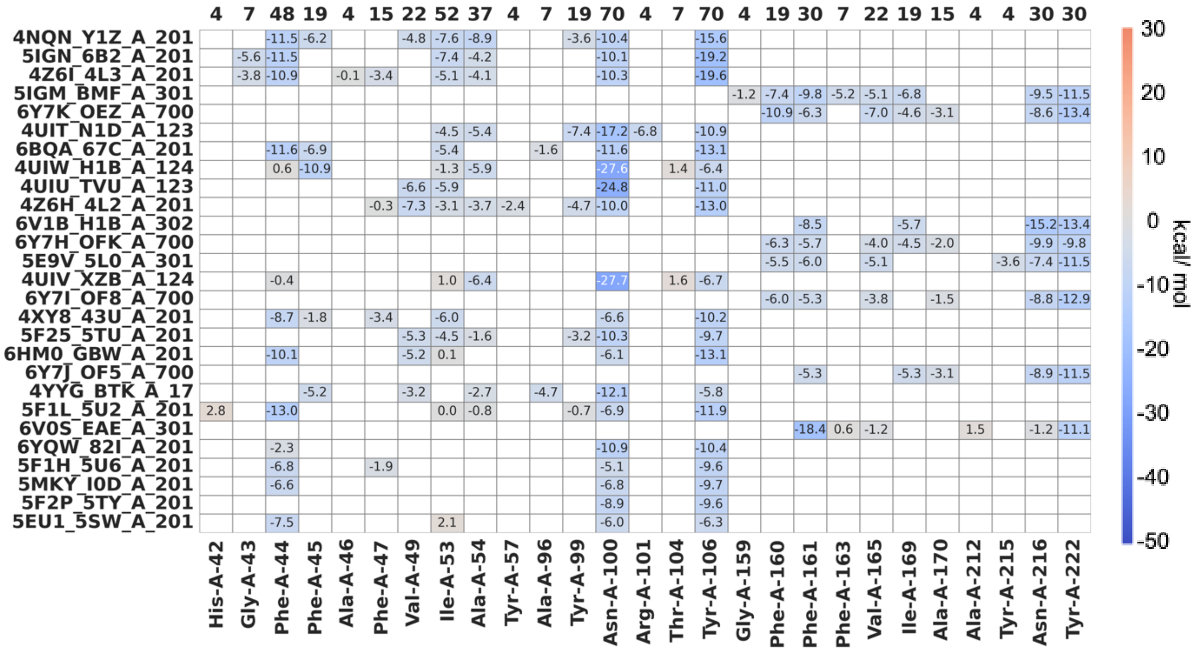

Bromodomain-containing protein (BRD-9)

FP-score plot

Apo-structure

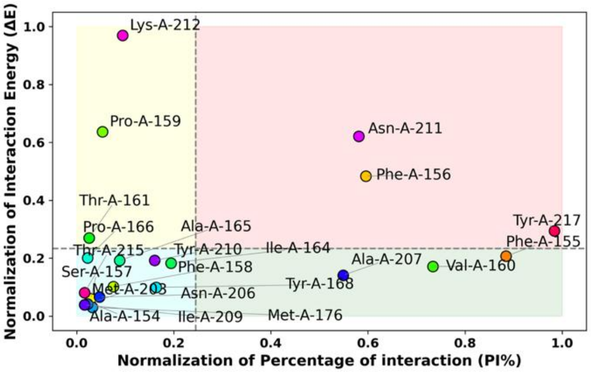

Holo-complexes

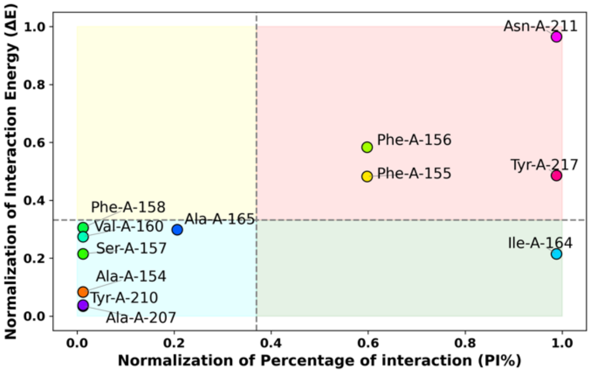

2D-FMOPhore heatmap

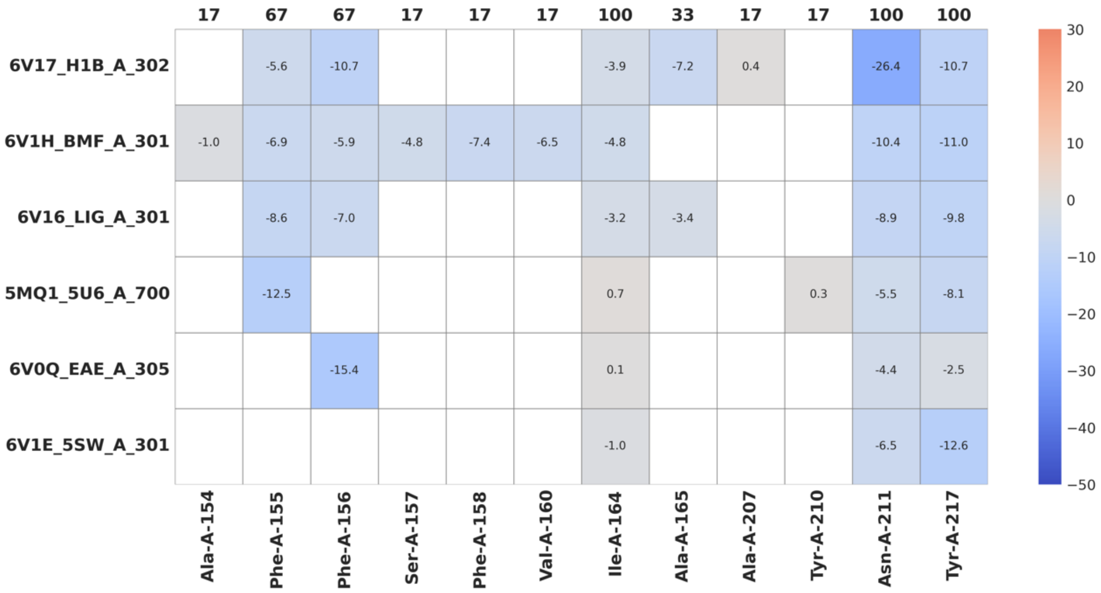

Bromodomain and plant homeodomain (PHD) finger containing protein 1 (BRPF1)

FP-score plot

Apo-structure

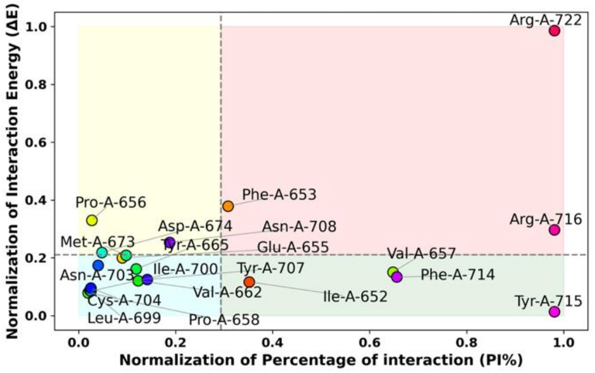

Holo-complexes

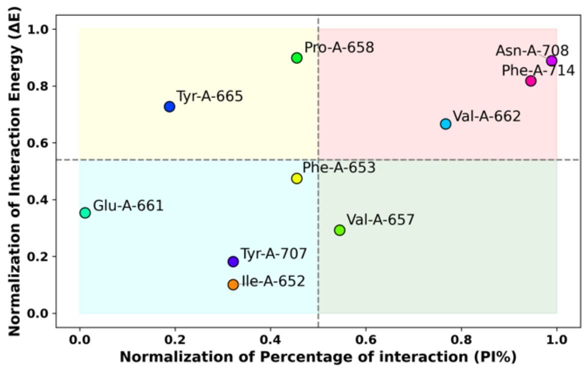

2D-FMOPhore heatmap

	Ile-A-652	Phe-A-653	Val-A-657	Pro-A-658	Glu-A-661	Val-A-662	Tyr-A-665	Tyr-A-707	Asn-A-708	Phe-A-714
	33	46	54	46	4	75	21	33	96	92
5DYA_5GV_A_802	-4.5	-3.6		-12.4		-10.1	-3.5		-12.3	-11.6
5C89_4YT_A_801		-5.6	-6.1			-4.3		-4.2	-9.4	-9.5
5EWW_5SK_A_801		-4.5	-6.2	-4.0				-3.4	-8.6	-10.4
5EWC_5SJ_A_801	-2.5	-8.2		-5.8		-3.5			-8.7	-8.0
5EV9_5SB_A_801	-0.4	-4.4	-4.0			-6.3		-4.2	-9.4	-7.9
5OWE_B0E_A_801	-2.5		-6.4			-3.9		-4.6	-9.4	-9.7
5ETD_5RN_A_801		-6.4	-5.3			-3.5		-3.7	-9.7	-7.3
5EWV_5SN_A_801		-5.2	-6.1			-2.4	-3.6		-8.5	-9.6
5ETB_5RO_A_801		-6.2	-5.2	-0.8		-3.7			-9.8	-7.8
5EPR_5QY_A_801	-1.0	-5.5	-4.2	-2.8		-2.0			-9.8	-7.9
5T4U_12Q_A_802			-5.4	-2.8		-1.9	-1.7	-3.7	-8.9	-7.3
5O4T_9KT_A_801				-2.0		-2.1	-5.2		-10.6	-8.6
5C87_4YS_A_802			-5.3				-10.7	-5.3		-5.2
5EPS_5QX_A_801	-1.5			-3.0		-2.3			-10.1	-7.8
5DYC_5GU_A_802					-7.0	-1.9			-8.4	-6.8
5EQ1_BEA_A_801				-2.8		-3.4			-7.7	-8.4
5EWD_5SH_A_801			-5.1	-2.8		-3.3			-3.9	-7.0
5D7X_XZ8_A_802		-7.9				-3.2		-3.3	-7.7	
5OWB_AYW_A_802	-1.4	-4.8	-5.4						-8.4	
5EWH_5SG_A_801	-2.4		-5.8						-4.4	-6.9
5E3G_5JQ_A_801									-11.2	-7.8
5DY7_5GT_A_801						-1.8			-8.2	-7.3
5C85_4YO_A_802						-1.8			-7.6	-6.2
5EVA_5S9_A_801				-3.3					-3.2	-7.7

Beta-secretase receptor

FP-score plot

Apo-structure

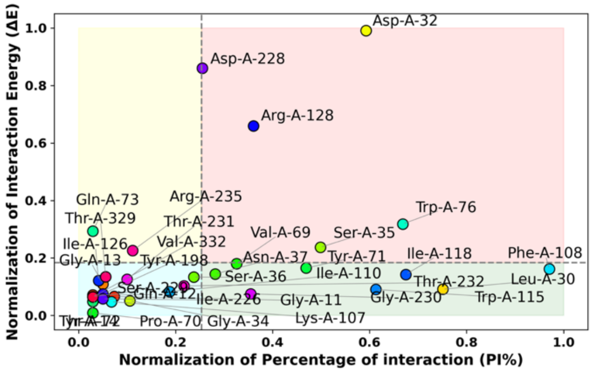

Holo-complexes

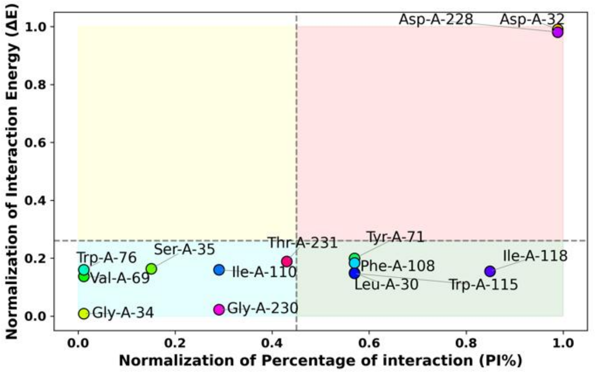

2D-FMOPhore heatmap

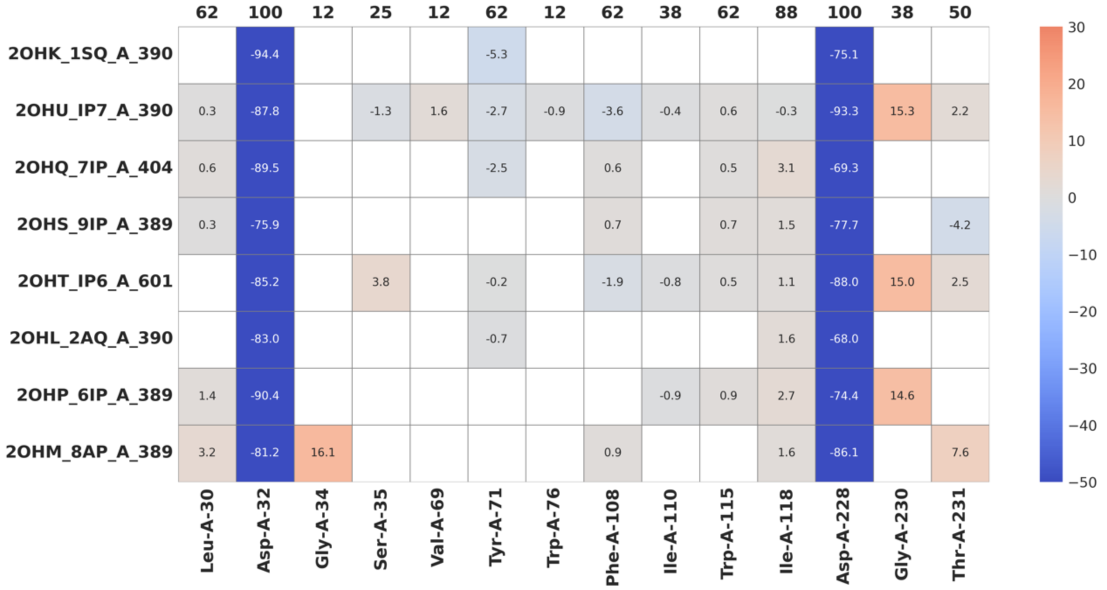

	Leu-A-30	Asp-A-32	Gly-A-34	Ser-A-35	Val-A-69	Tyr-A-71	Trp-A-76	Phe-A-108	Ile-A-110	Trp-A-115	Ile-A-118	Asp-A-228	Gly-A-230	Thr-A-231
	62	100	12	25	12	62	12	62	38	62	88	100	38	50
2OHK_1SQ_A_390		-94.4				-5.3						-75.1		
2OHU_IP7_A_390	0.3	-87.8		-1.3	1.6	-2.7	-0.9	-3.6	-0.4	0.6	-0.3	-93.3	15.3	2.2
2OHQ_7IP_A_404	0.6	-89.5				-2.5		0.6		0.5	3.1	-69.3		
2OHS_9IP_A_389	0.3	-75.9						0.7		0.7	1.5	-77.7		-4.2
2OHT_IP6_A_601		-85.2		3.8		-0.2		-1.9	-0.8	0.5	1.1	-88.0	15.0	2.5
2OHL_2AQ_A_390		-83.0				-0.7					1.6	-68.0		
2OHP_6IP_A_389	1.4	-90.4							-0.9	0.9	2.7	-74.4	14.6	
2OHM_8AP_A_389	3.2	-81.2	16.1					0.9			1.6	-86.1		7.6

Biotin carboxylase

PIEDA plots

Table –: Shows PIEDA plots for holo-complex analysis.

2W6O	2W6Z
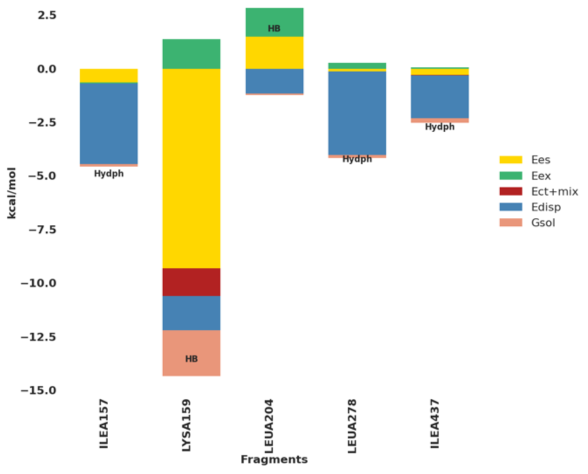	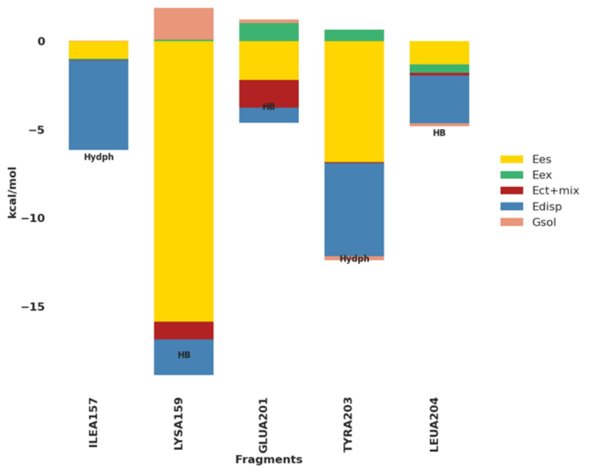

Carboxylesterase Notum

FP-score plot

Apo-structure

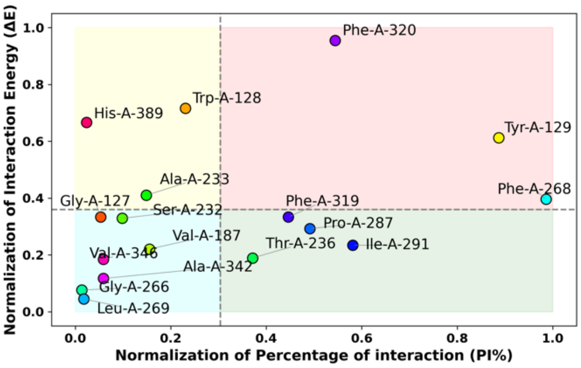

Holo-complexes

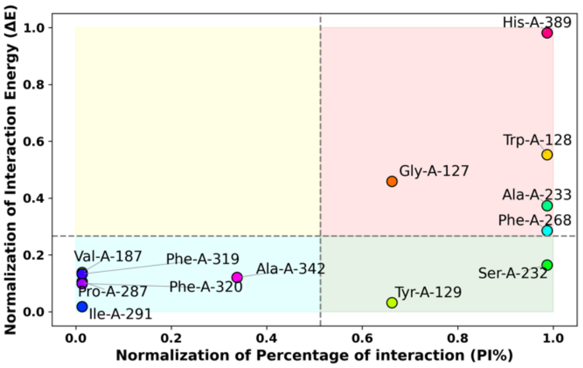

2D-FMOPhore heatmap

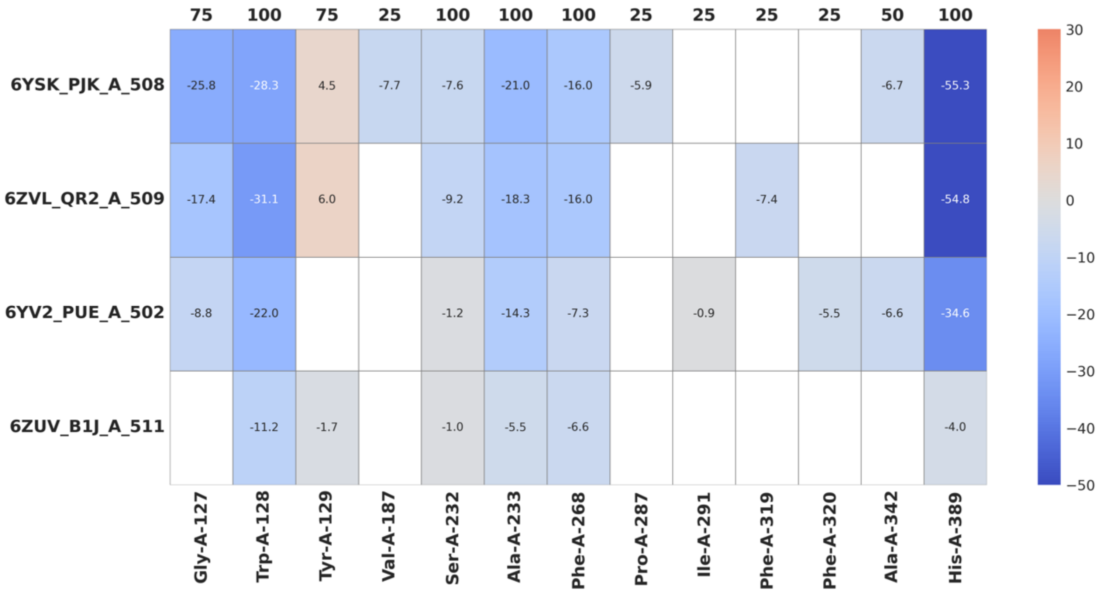

Cyclin-dependent Kinase-2 (CDK-2)

FP-score plot

Apo-structure

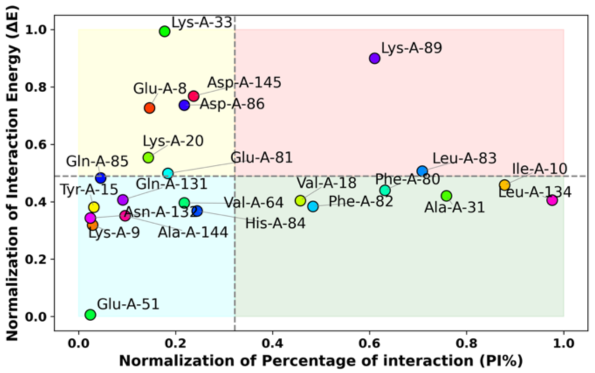

Holo-complexes

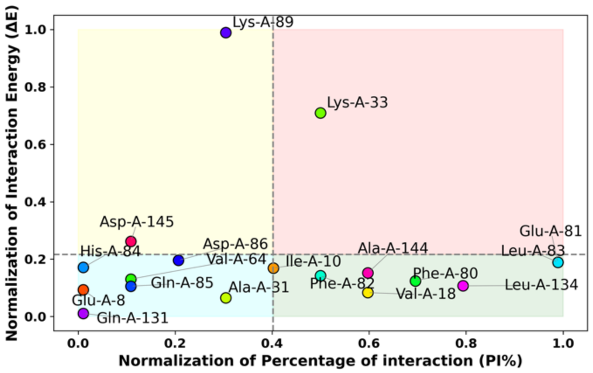

2D-FMOPhore heatmap

	Glu-A-8	Ile-A-10	Val-A-18	Ala-A-31	Lys-A-33	Val-A-64	Phe-A-80	Glu-A-81	Phe-A-82	Leu-A-83	His-A-84	Gln-A-85	Asp-A-86	Lys-A-89	Gln-A-131	Leu-A-134	Ala-A-144	Asp-A-145
	9	45	64	36	55	18	73	100	55	100	9	18	27	36	9	82	64	18
4FKQ_42K_A_301		-12.8			-63.4	-9.2	-8.6	17.1		-14.7			16.1	-37.1	2.0	-6.4	-11.2	
4FKG_4CK_A_300				-3.1			-5.0	9.5	-9.8	-9.0				-89.6		-4.2	-3.1	
4FKW_62K_A_301			-4.8		-22.5		-1.9	-1.3		-8.0		-6.9	-15.3	-16.7		-5.4		-3.8
2VU3_LZE_A_299			-3.4					-14.7	-9.5	1.3	-13.0					-7.0		-21.5
1VYW_292_A_300	-5.7	-12.8	-2.8	-1.6			-2.2	-2.7	-9.7	-5.2						-5.5		
4FKI_09K_A_301		-10.6	-2.5	-1.3	-3.1		-1.6	-3.3	-10.3	-4.9						-3.9	-0.7	
4FKS_46K_A_301			-1.6		11.7	2.4	1.8	-12.4		-3.1		-6.4	1.1	-33.4		-3.4	1.8	
1VYZ_N5B_A_300		-6.0	-1.7	-1.4			-1.6	-4.3	-9.5	-5.3						-4.3	-0.8	
4FKL_CK2_A_300		-3.1			-17.3			0.9		-3.4							-1.6	
4FKR_45K_A_301					-0.6		-3.7	0.9	-4.9	-6.9						-4.8	-0.8	
2VTA_LZ1_A_301			-2.7					-2.5		-4.6								

Dipeptidyl peptidase IV (DPP-4)

FP-score plot

Apo-structure

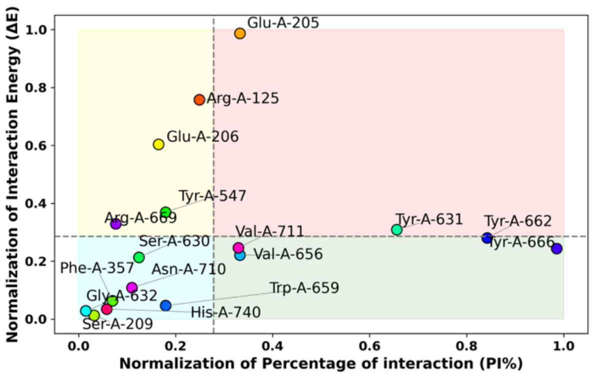

Holo-complexes

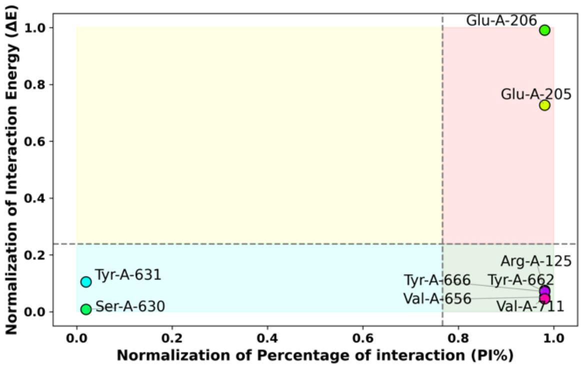

2D-FMOPhore heatmap

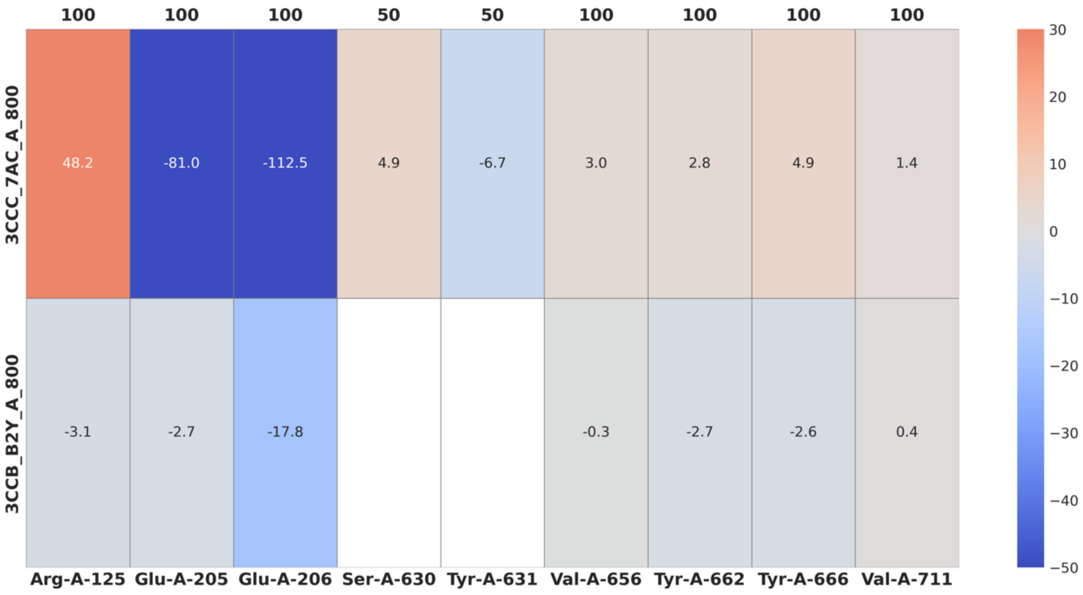

DNA-gyrase

FP-score plot

Apo-structure

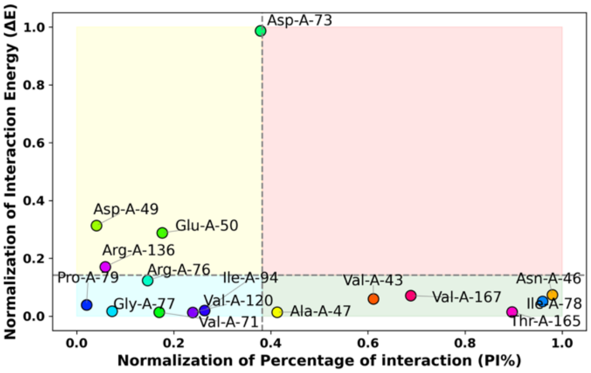

Holo-complexes

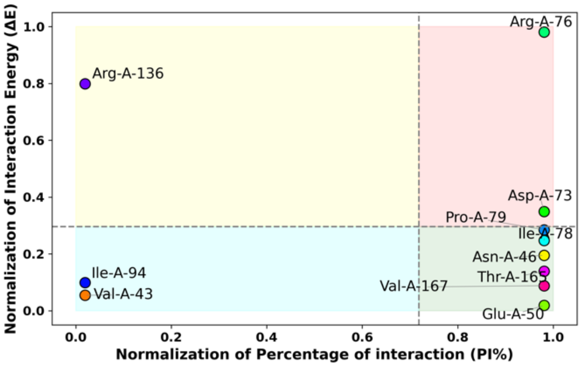

2D-FMOPhore heatmap

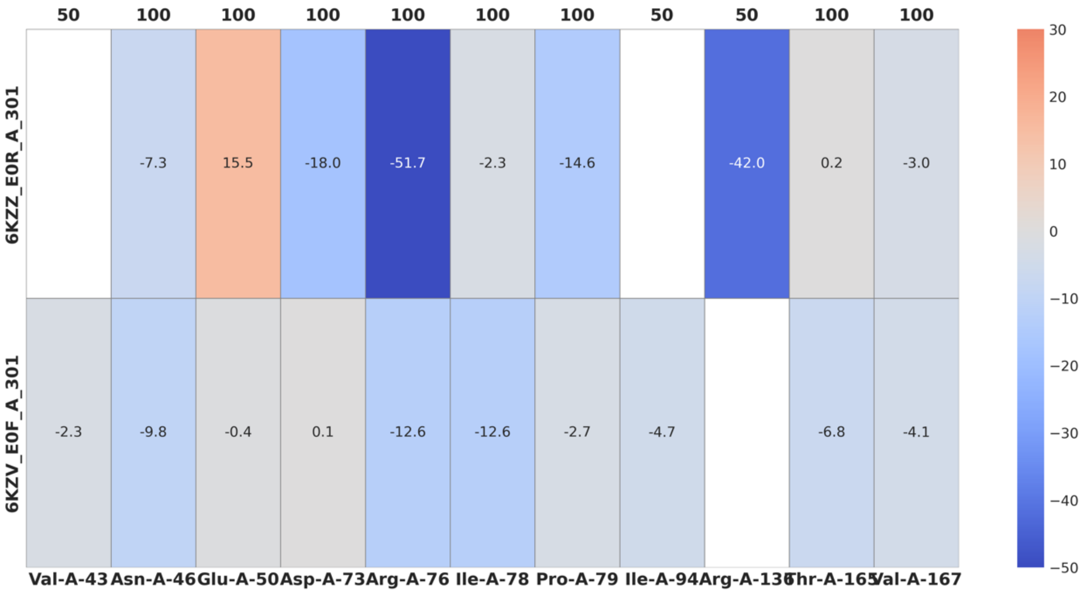

Tyrosine kinase EPHA2 (Ephrin type-A receptor 2)

FP-score plot

Apo-structure

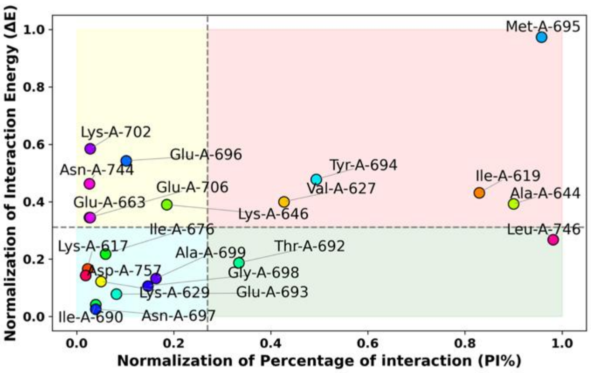

Holo-complexes

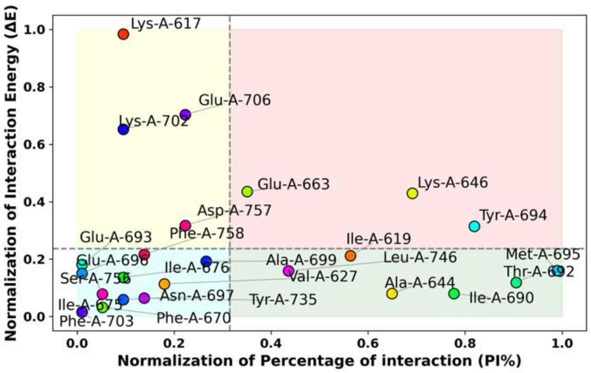

2D-FMOPhore heatmap

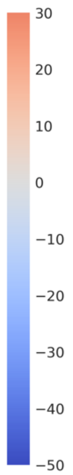

	Lys-A-617	Ile-A-619	Val-A-627	Ala-A-644	Lys-A-646	Glu-A-663	Phe-A-670	Ile-A-675	Ile-A-676	Ile-A-690	Thr-A-692	Glu-A-693	Tyr-A-694	Met-A-695	Glu-A-696	Asn-A-697	Ala-A-699	Lys-A-702	Phe-A-703	Glu-A-706	Tyr-A-735	Leu-A-746	Ser-A-756	Asp-A-757	Phe-A-758
	12	58	21	67	71	38	8	4	12	79	92	4	83	100	4	12	29	12	4	25	17	46	8	25	17
5NKD_91K_A_1	-60.8	-8.7		-2.7	-24.6					-4.4	-6.4		-6.6	-8.3											
5NKC_90T_A_1	-40.3	-12.6			-26.2	11.3				-4.4	-5.8		-19.0	-8.8											
5I9Z_627_A_1				-0.9	-21.5						1.5	-10.7	-15.1	-1.5						-39.1		-9.3	-0.8		
5IA1_ZZL_A_1		-7.5	-6.5	-3.5									-4.8	-9.4				-40.1				-8.0	-4.3	1.3	
6FNG_DWT_A_1			-3.7			-26.6			-7.9	-0.3	-6.8		-7.0	-6.5							-3.4			-8.6	-11.0
5IA5_GV0_A_1					-12.8	-7.9	-1.2		-3.4		-1.8			-1.6						-20.3	-0.6			-16.5	-12.0
5IA4_88Z_A_1			-3.6	-3.3	-12.1	-10.1	-1.4	-0.4	-3.7		-2.3		-6.8	-3.7							-0.3			-19.2	-10.6
6FNF_DXH_A_1			-5.6			-25.7				-0.6	-6.2			-7.0							-2.8	-5.1		-6.7	-12.9
5I9Y_1N1_A_1				-3.2	-18.8	2.6				-2.5	-6.1		-9.8	-6.6			-5.1	-10.2		-10.0					
5NK8_90Z_A_1		-6.3			-4.2					-1.7	-4.2		-11.8	-4.9		-3.1			-0.4	-28.7					
5NKH_8ZQ_A_1		-5.8			-2.2					-1.1	-4.3		-12.5	-2.1			-6.4			-30.8					
5NKA_91H_A_1	-14.6	-9.9			-6.2					-3.5	-5.0		-14.2	-7.2											
5NK2_8ZZ_A_1		-6.6		-2.2	-9.1	-5.9				-0.8	-5.3		-13.1	-3.1	-8.8							-5.6			
5IA2_L66_A_1			-5.7	-4.4	-0.5	-21.4				-1.5	-5.3			-9.3										-7.8	
5NK6_90W_A_1		-4.9		-2.5		-6.8				-2.1	-5.7		-8.1	-6.3			-11.4	-1.9				-5.6			
5I9X_DB8_A_1		-1.9		-3.4	2.2									-3.6			-2.5			-43.3					
5NKB_8ZT_A_1		-7.5		-3.2						-2.8	-5.7		-9.9	-5.7								-6.1			
5IA3_P17_A_1				-2.1	-15.0					-2.3	-3.2		-7.6	-4.8								-5.6			
5NKI_8ZW_A_1		-6.3		-2.5						-2.5	-5.7		-8.8	-5.6								-5.7			
5NKG_8ZK_A_1		-6.9		-1.9	-2.7					-0.7	-4.8		-11.2	-2.6								-5.5			
5NK7_8ZZ_A_1				-2.2	-5.4					-0.6	-5.0		-12.2	-2.5		1.6						-5.3			
5NJZ_8ZH_A_1				-2.1						-0.5	-5.4		-7.5	-3.0		-1.7	-5.5					-5.8			
5NK3_92Q_A_1		-1.3			1.0					0.6	-4.7		-11.2	-4.2			-9.4								
5NK4_90E_A_1		-2.3		-0.8	10.0					0.7	-5.1		-11.4	-2.9			-6.0								

Heat shock protein (HSP90)

FP-score plot

Apo-structure

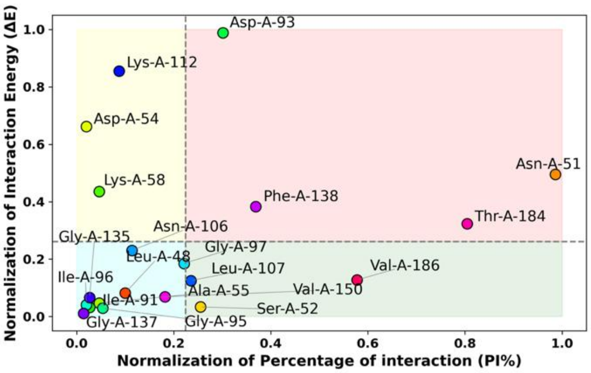

Holo-complexes

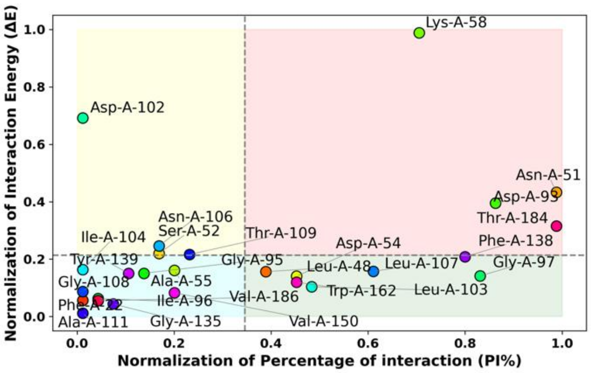

2D-FMOPhore heatmap

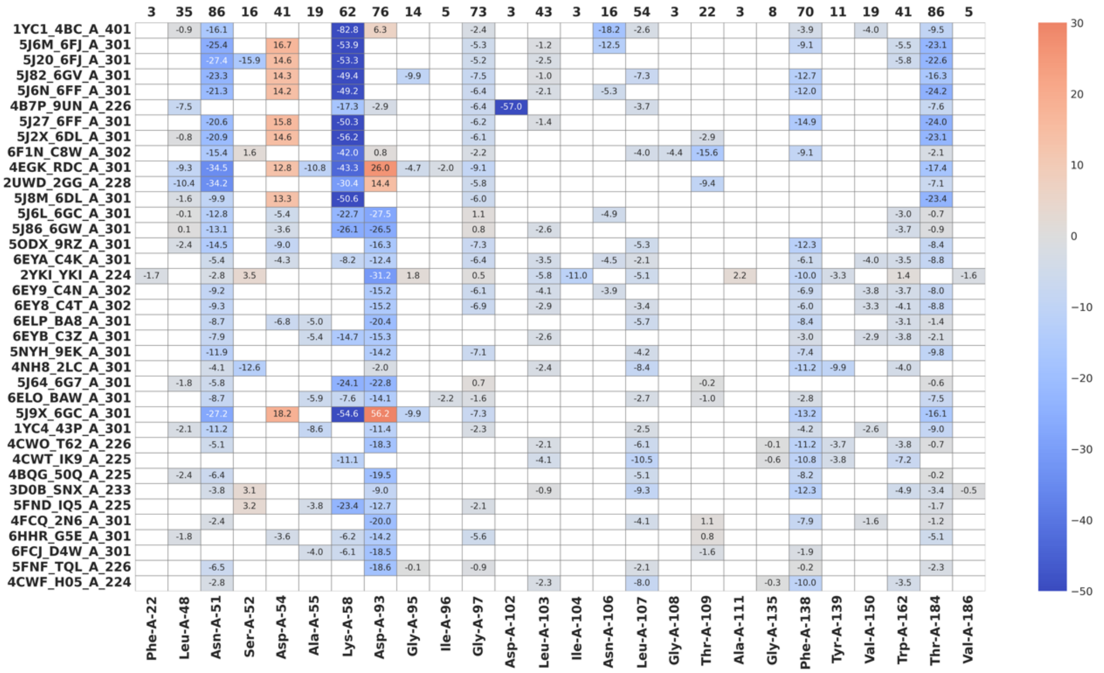

Hepatitis-C virus (HCV NS5b RNA polymerase)

FP-score plot

Apo-structure

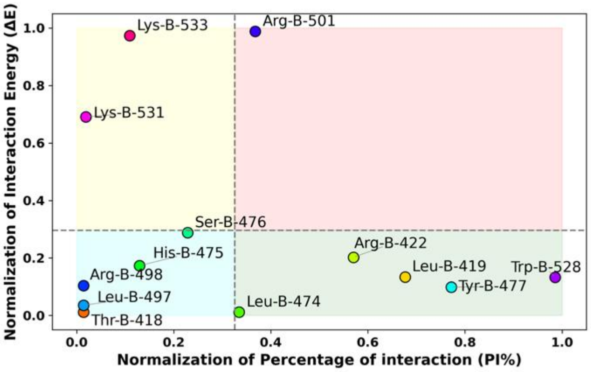

Holo-complexes

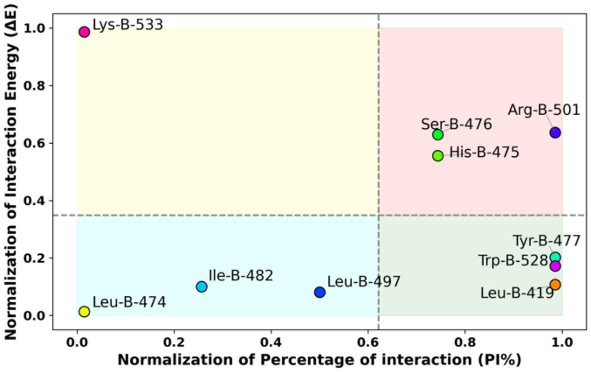

2D-FMOPhore heatmap

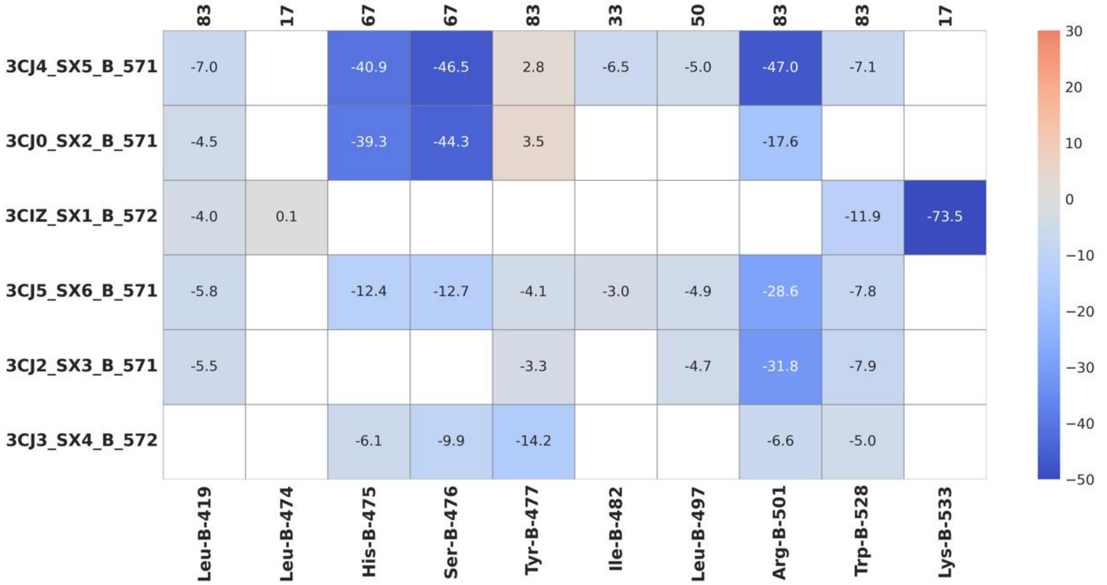

Human Immunodeficiency Virus protease (HIV-1)

FP-score plot

Apo-structure

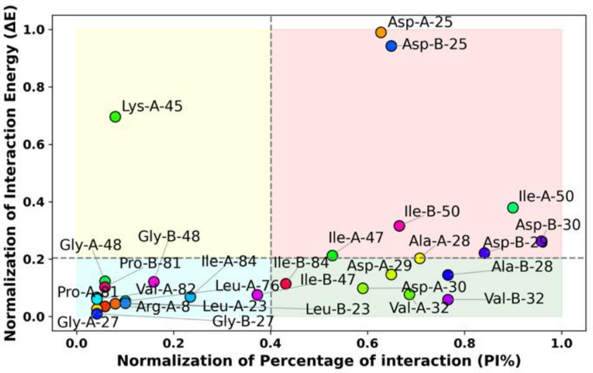

Holo-complexes

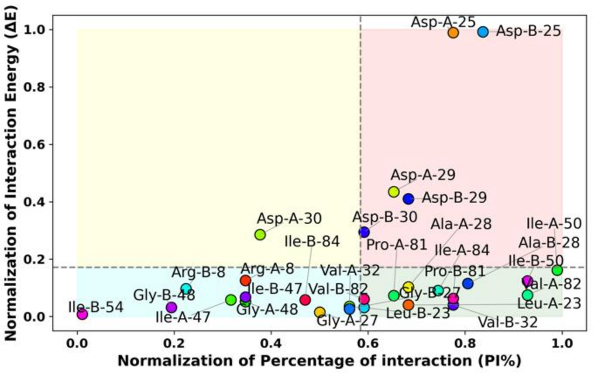

2D-FMOPhore heatmap

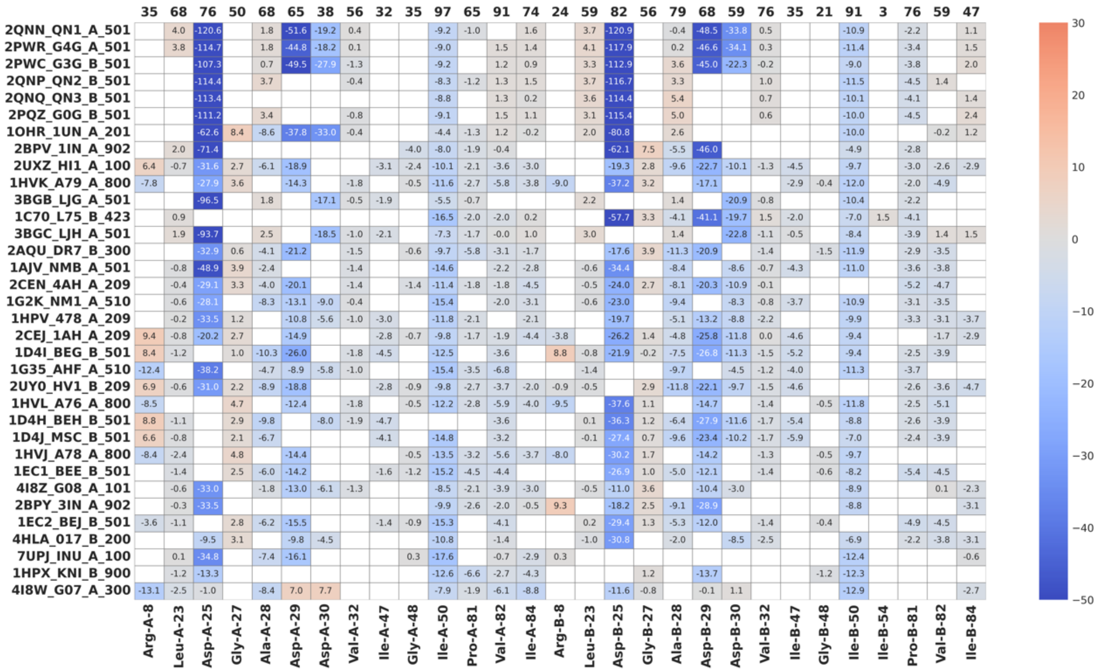

Janus Kinase family (JAK-1)

FP-score plot

Apo-structure

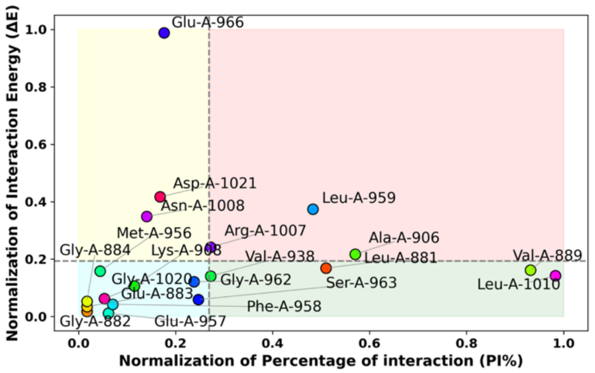

Holo-complexes

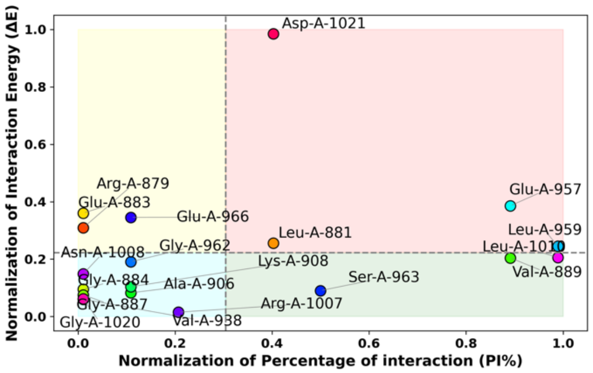

2D-FMOPhore heatmap

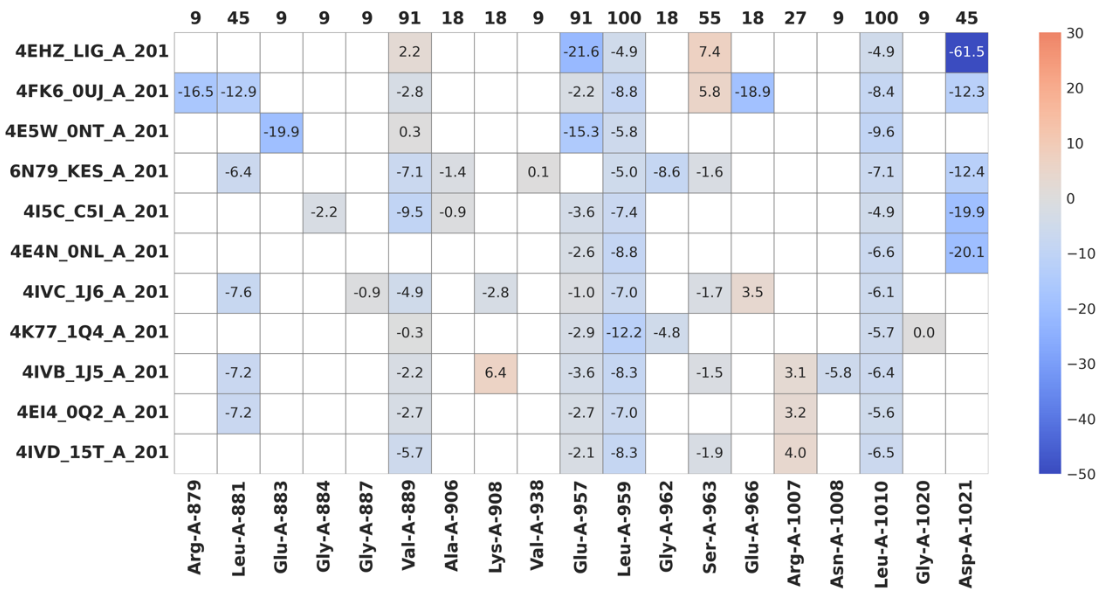

	Arg-A-879	Leu-A-881	Glu-A-883	Gly-A-884	Gly-A-887	Val-A-889	Ala-A-906	Lys-A-908	Val-A-938	Glu-A-957	Leu-A-959	Gly-A-962	Ser-A-963	Glu-A-966	Arg-A-1007	Asn-A-1008	Leu-A-1010	Gly-A-1020	Asp-A-1021
	9	45	9	9	9	91	18	18	9	91	100	18	55	18	27	9	100	9	45
4EHZ_LIG_A_201						2.2				-21.6	-4.9		7.4				-4.9		-61.5
4FK6_0UJ_A_201	-16.5	-12.9				-2.8				-2.2	-8.8		5.8	-18.9			-8.4		-12.3
4E5W_0NT_A_201			-19.9			0.3				-15.3	-5.8						-9.6		
6N79_KES_A_201		-6.4				-7.1	-1.4		0.1		-5.0	-8.6	-1.6				-7.1		-12.4
4I5C_C5I_A_201				-2.2		-9.5	-0.9			-3.6	-7.4						-4.9		-19.9
4E4N_0NL_A_201										-2.6	-8.8						-6.6		-20.1
4IVC_1J6_A_201		-7.6			-0.9	-4.9		-2.8		-1.0	-7.0		-1.7	3.5			-6.1		
4K77_1Q4_A_201						-0.3				-2.9	-12.2	-4.8					-5.7	0.0	
4IVB_1J5_A_201		-7.2				-2.2		6.4		-3.6	-8.3		-1.5		3.1	-5.8	-6.4		
4EI4_0Q2_A_201		-7.2				-2.7				-2.7	-7.0				3.2		-5.6		
4IVD_15T_A_201						-5.7				-2.1	-8.3		-1.9		4.0		-6.5		

Janus Kinase family (JAK-2)

FP-score plot

Apo-structure

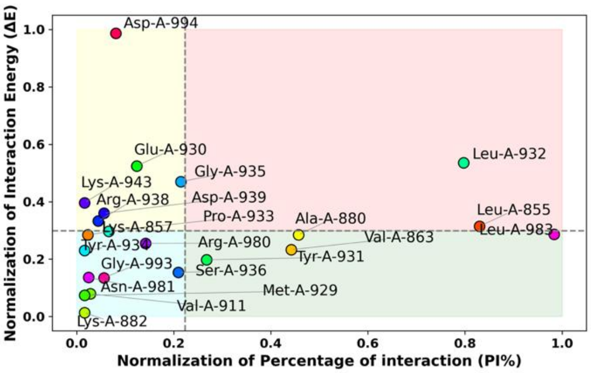

Holo-complexes

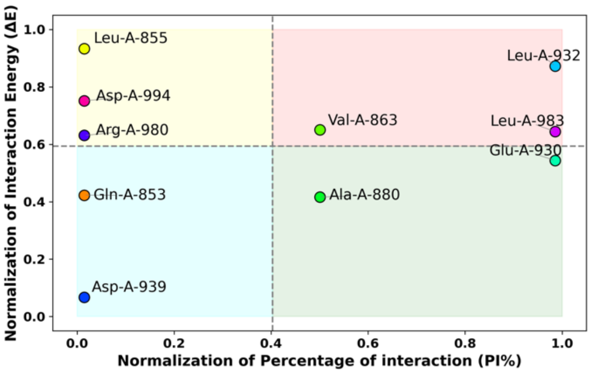

2D-FMOPhore heatmap

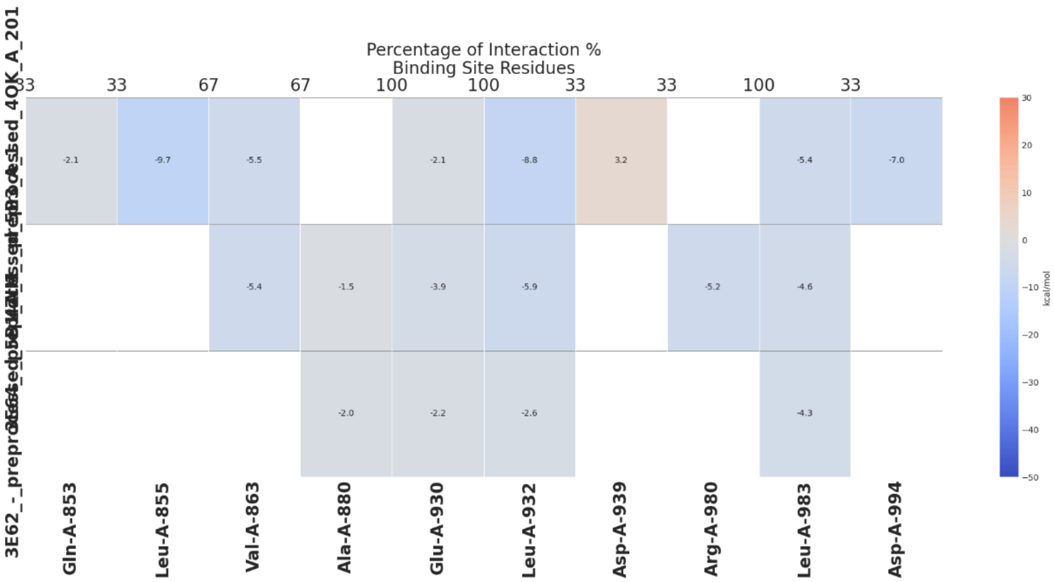

Janus Kinase family (JAK-3)

FP-score plot

Apo-structure

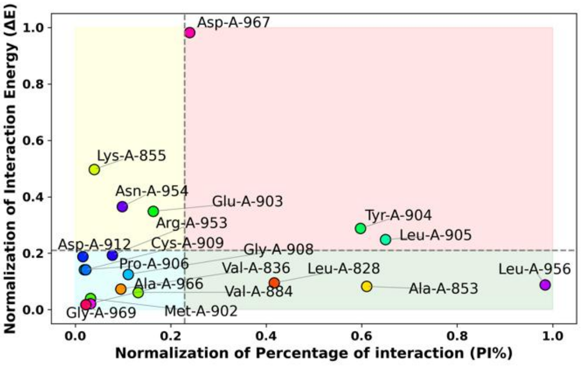

Holo-complexes

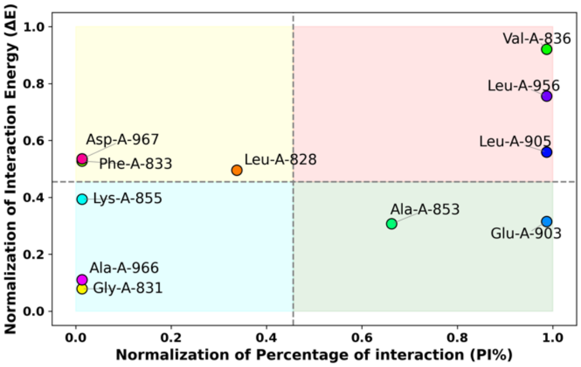

2D-FMOPhore heatmap

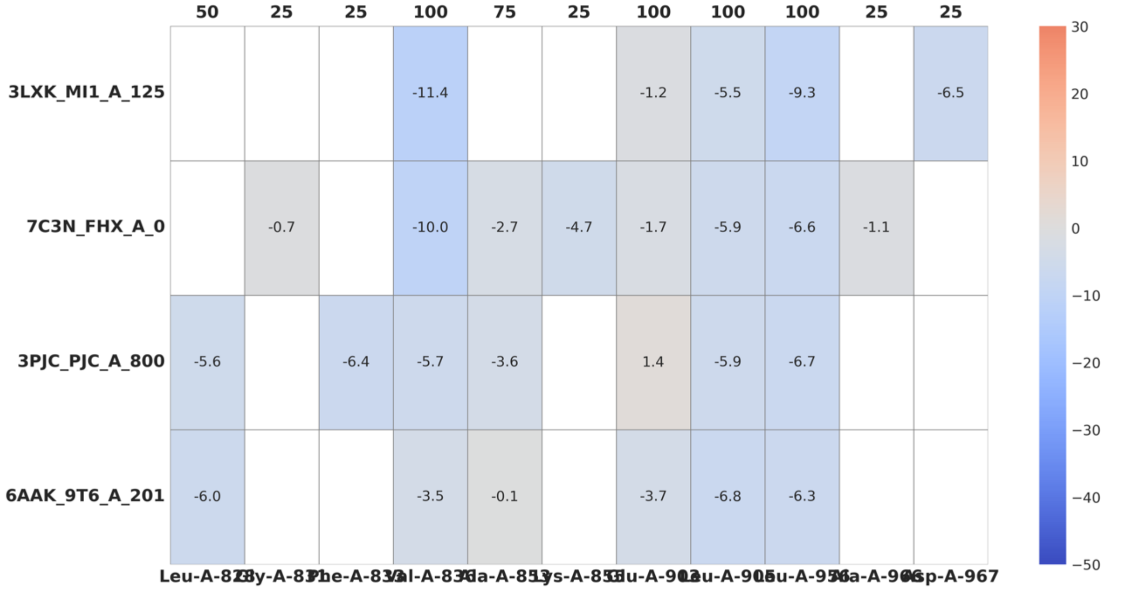

Main Protease Protein (Mpro)

FP-score plot

Apo-structure

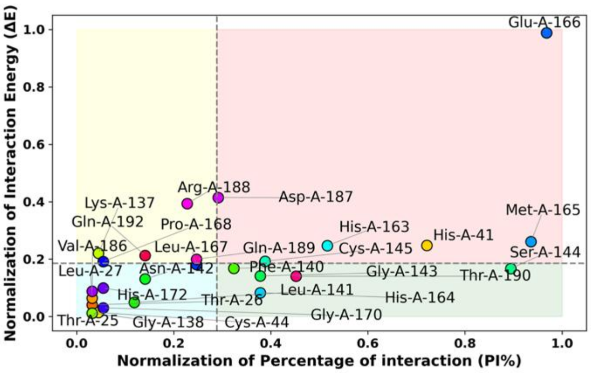

Holo-complexes

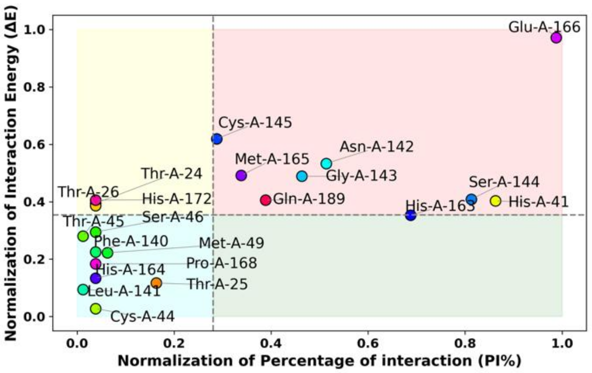

2D-FMOPhore heatmap

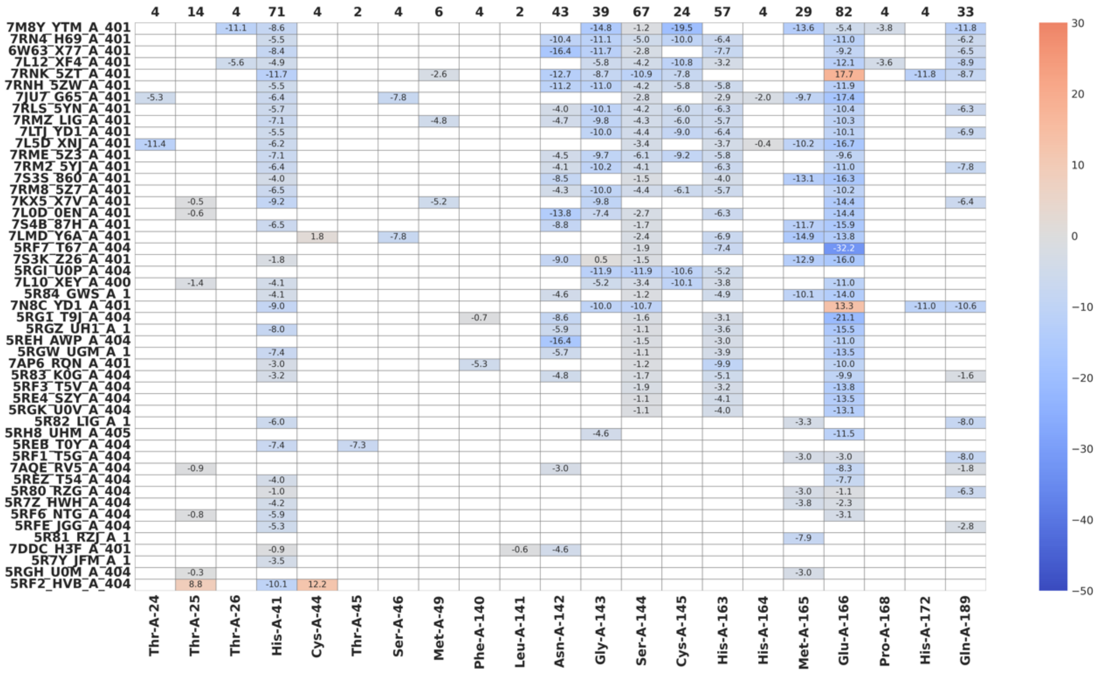

M. Tuberculosis-InhA

FP-score plot

Apo-structure

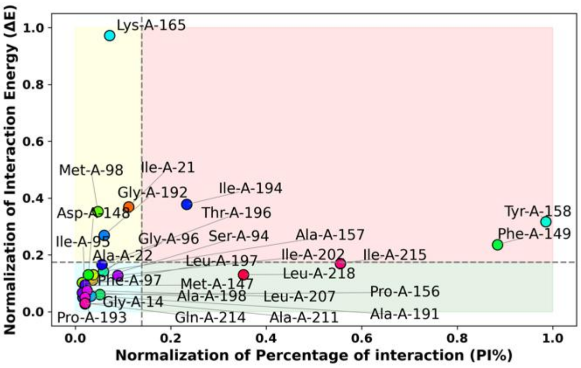

Holo-complexes

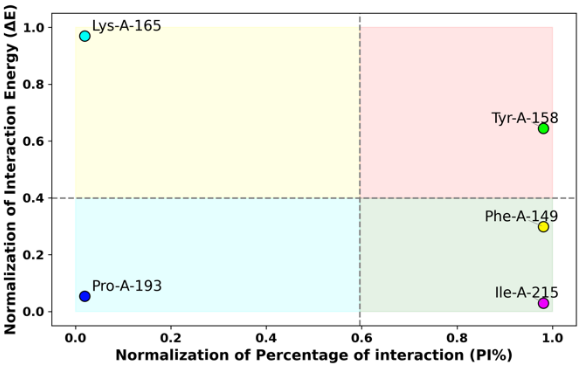

2D-FMOPhore heatmap

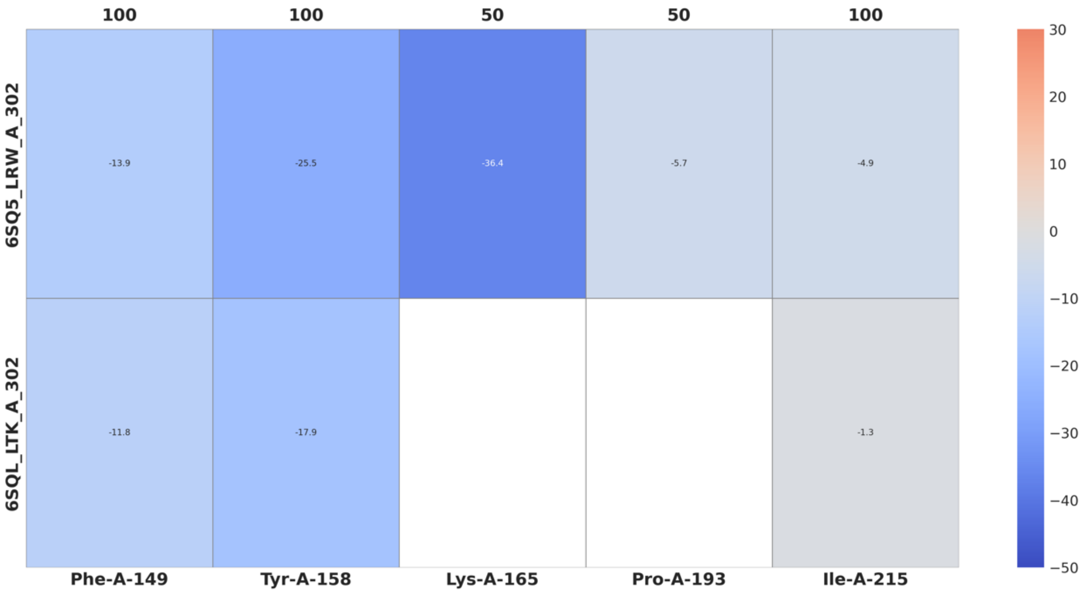

Phosphodiesterase 4 (PDE4)

FP-score plot

Apo-structure

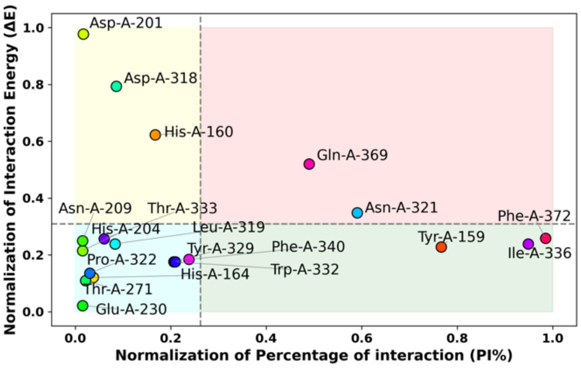

Holo-complexes

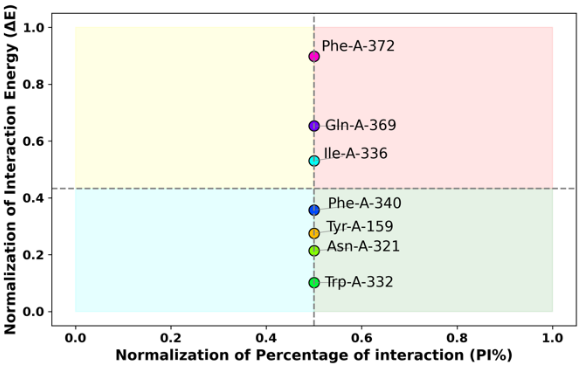

2D-FMOPhore heatmap

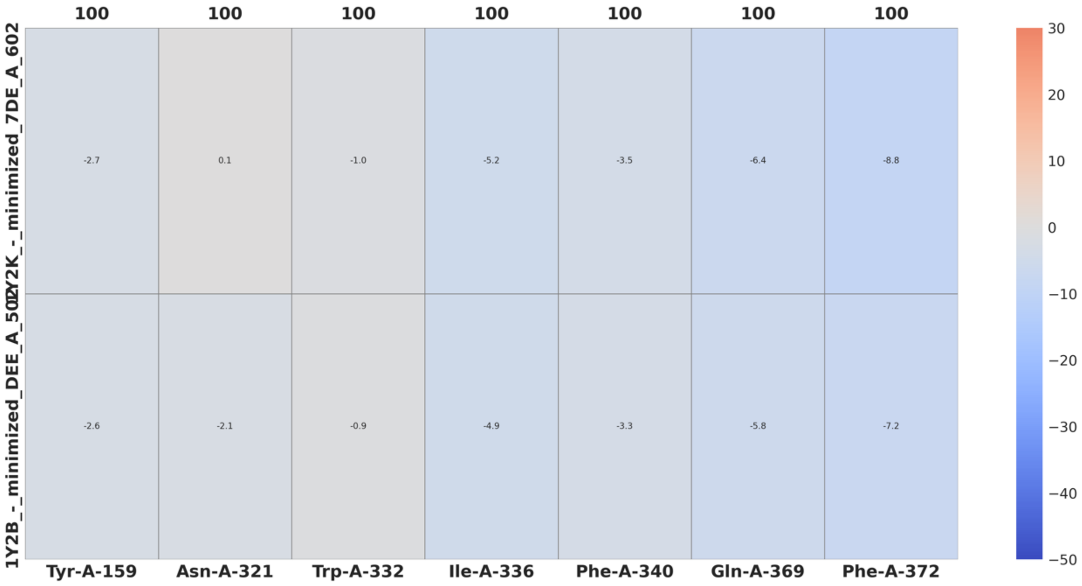

Papain-like protease (PL-pro)

FP-score plot

Apo-structure

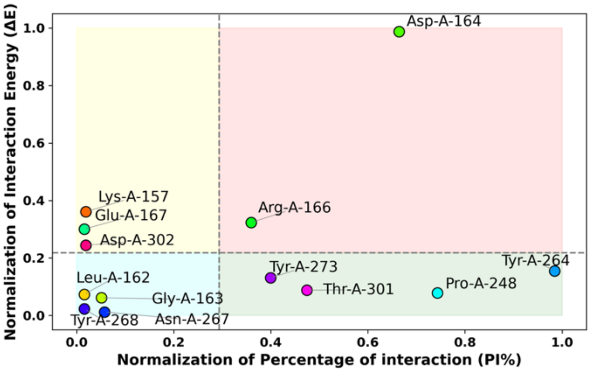

Holo-complexes

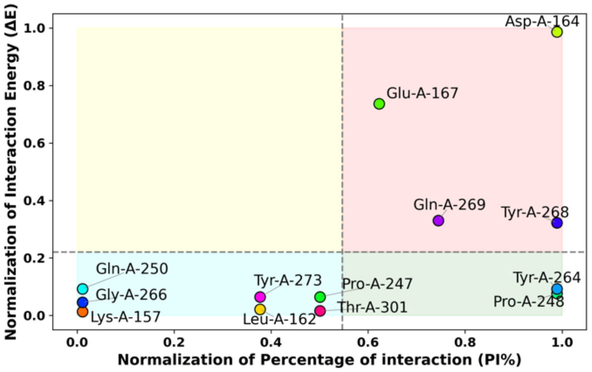

2D-FMOPhore heatmap

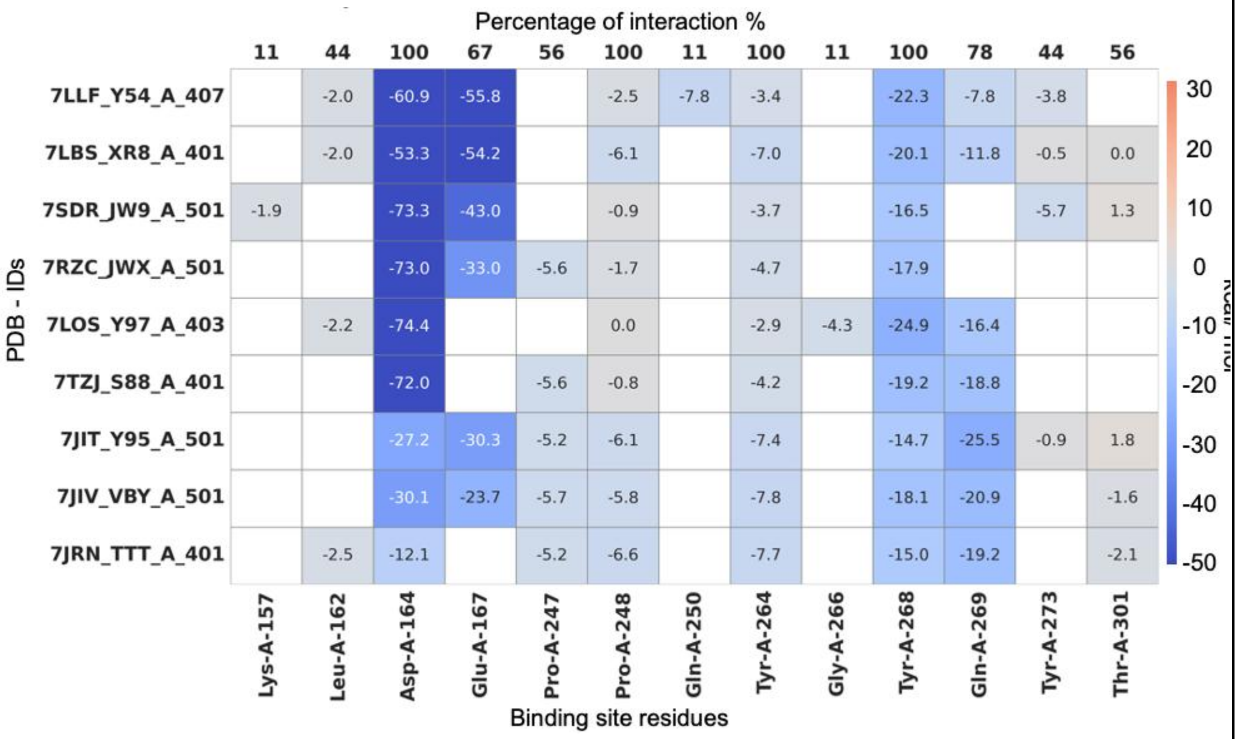

Proto-oncogene serine/threonine-protein kinase (Pim-1)

FP-score plot

Apo-structure

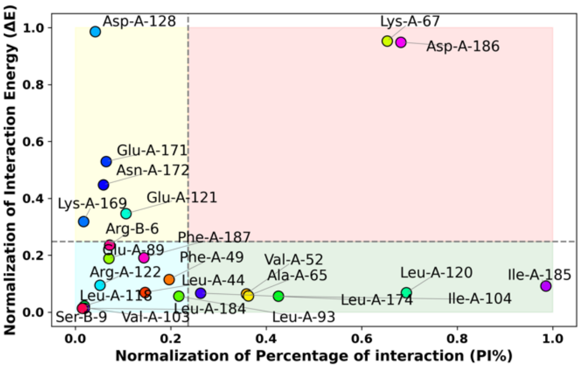

Holo-complexes

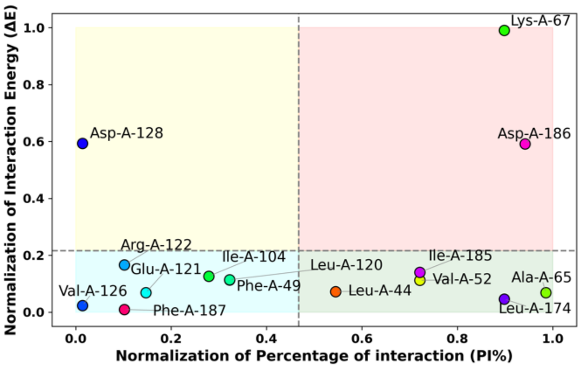

2D-FMOPhore heatmap

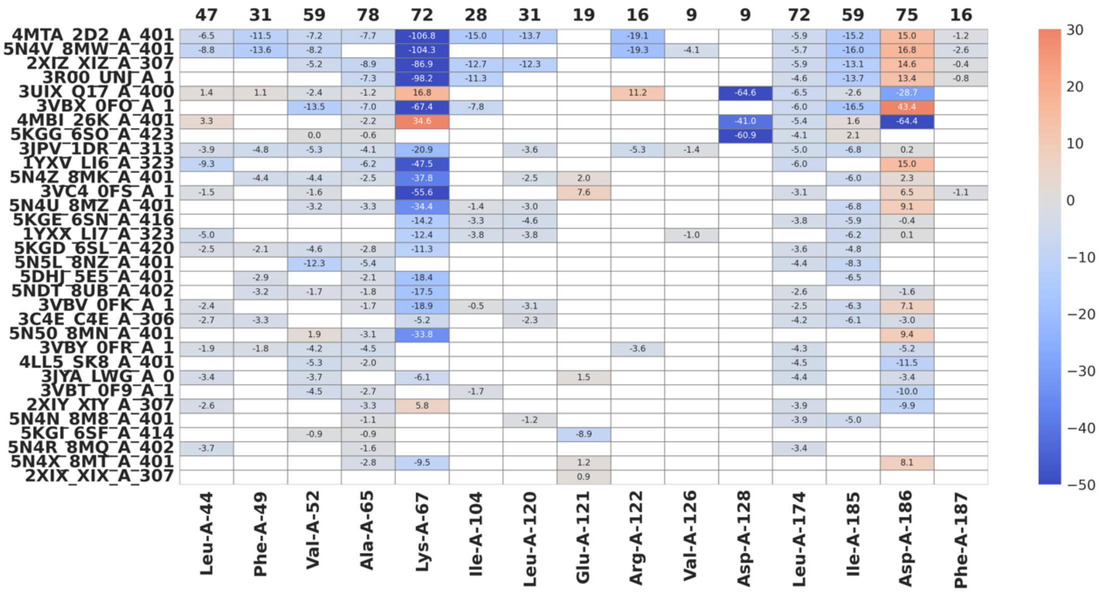

Peroxisome proliferator-activated receptor (PPARs) gamma

FP-score plot

Apo-structure

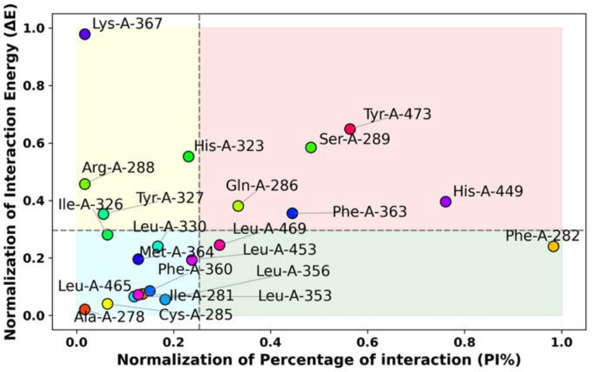

Holo-complexes

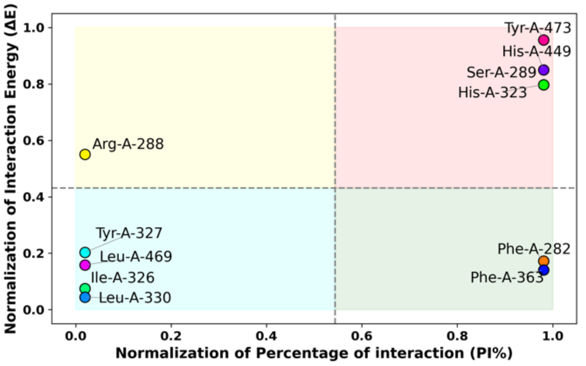

2D-FMOPhore heatmap

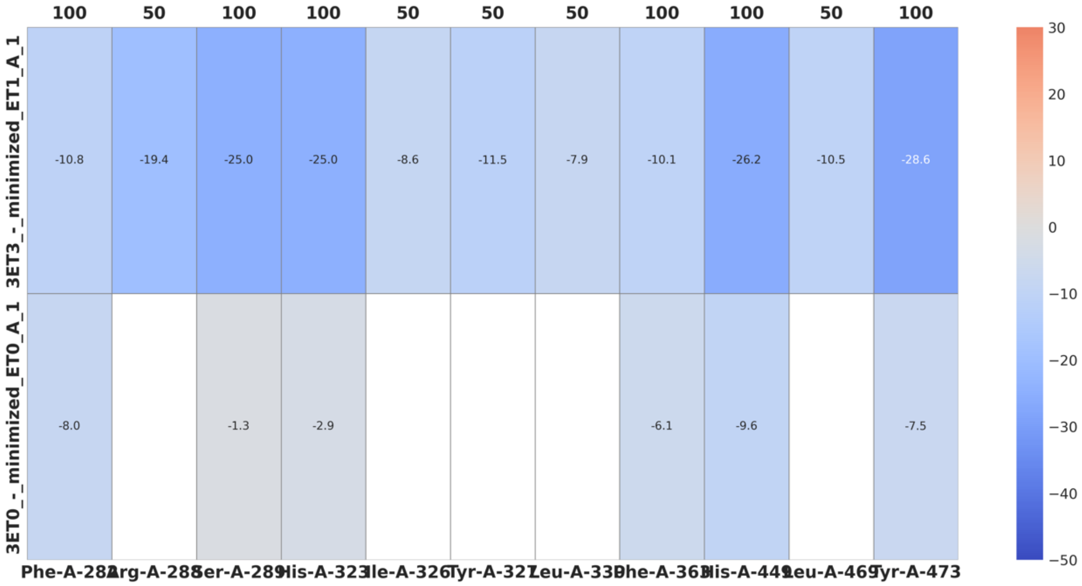

Phosphoinositide 3-kinase R (PI3KR)

FP-score plot

Apo-structure

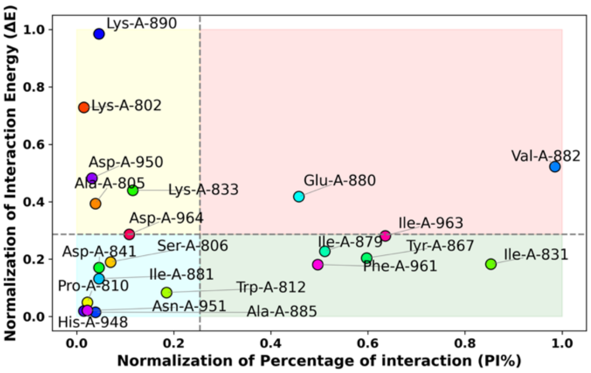

Holo-complexes

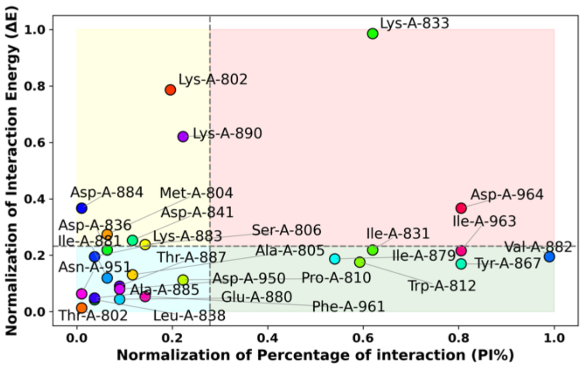

2D-FMOPhore heatmap

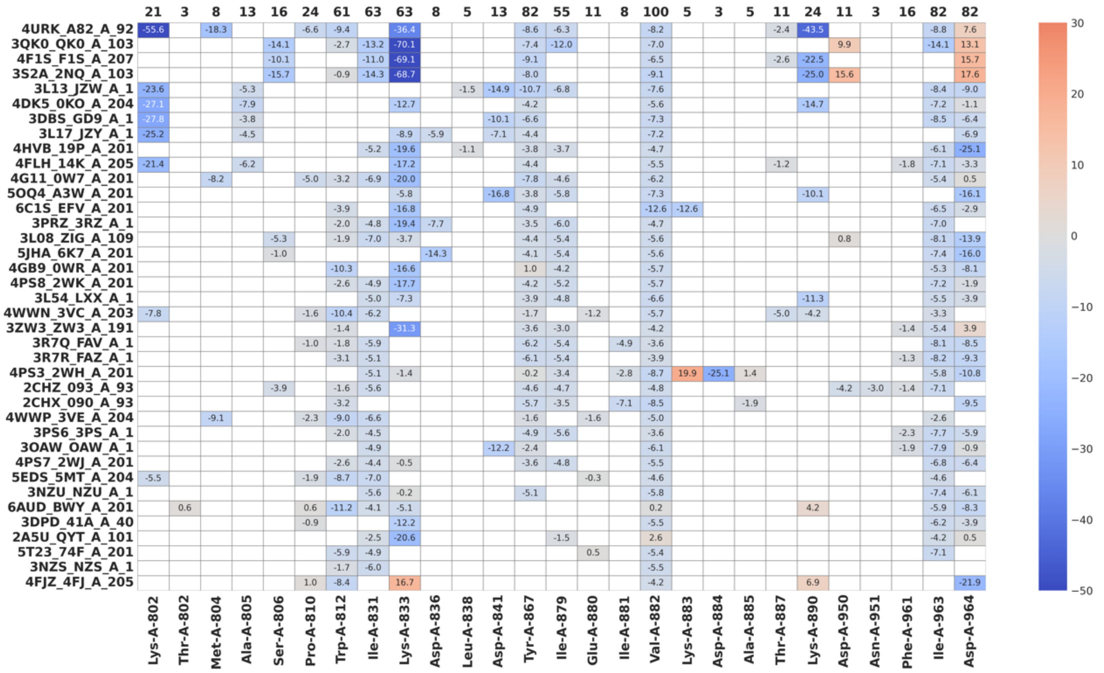

Protein Kinase B

FP-score plot

Apo-structure

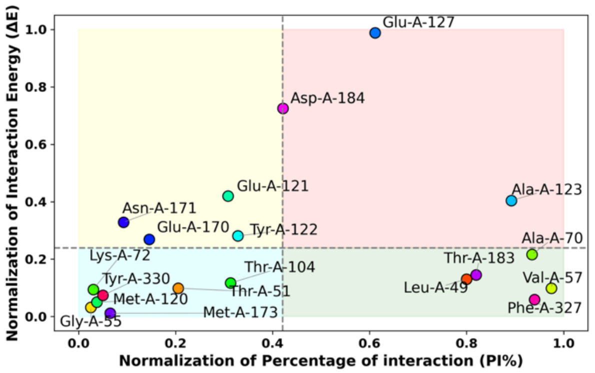

Holo-complexes

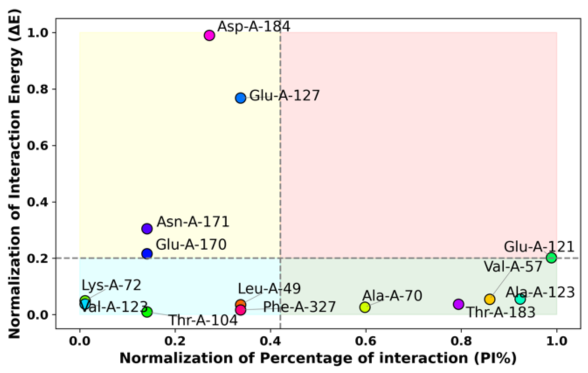

2D-FMOPhore heatmap

	Leu-A-49	Val-A-57	Ala-A-70	Lys-A-72	Thr-A-104	Glu-A-121	Ala-A-123	Val-A-123	Glu-A-127	Glu-A-170	Asn-A-171	Thr-A-183	Asp-A-184	Phe-A-327
	35	82	59	6	18	94	88	6	35	18	18	76	29	35
2UVZ_GVJ_A_351	0.4	-4.7				-18.9	-2.0				-31.1		-89.2	
2VNW_M01_A_351						-17.1	-3.1						-98.7	
2VO0_M03_A_354		-3.1	1.2			-16.8	-3.3					6.2	-100.3	
2uw6_GVO_A_351		-4.1	4.8			-20.7	-3.2					5.4	-79.6	
2VO6_M05_A_352		-4.2	2.7			-16.0	-2.3		-77.9			2.0		
2VNY_M02_A_351						-18.1	-1.5		-72.9			-0.6		
2UVY_GVI_A_351	0.2	-1.4				-16.4	-2.0		-68.4			-1.7		1.1
2uw7_GVP_A_351		-4.6	4.2			-14.3	-2.7		-63.5			2.1		0.3
2UW0_GVK_A_351		-5.7				-13.7	-2.1		-52.7					
2uw9_GVP_A_480		-5.8	2.5			-14.2	0.0		-52.4			-0.6		
2uw8_GVQ_A_351	4.3	-2.5	3.7								-29.9		-38.1	2.1
2VO3_M04_A_352		-4.0	1.7	-5.3	4.2	-16.5	-2.4			-22.1		-14.6		
2uw5_GVN_A_351		-4.9	3.8			-15.9	-2.4				-25.0	1.6		
2VO7_M05_A_352			2.6			-13.0		-4.1		-21.8		-3.2		
2uw4_L15_A_351	1.3	-2.9			0.9	-17.8	0.1			-18.4		-0.3		0.5
2uw3_GVG_A_351	-3.9	-4.8			-1.3	-3.2	-5.3					-2.5		-2.0
2UVX_GVH_A_351	-2.2	-2.3	-3.0			-0.7	-5.9					-4.0		-1.2

p38α MAP kinase

FP-score plot

Apo-structure

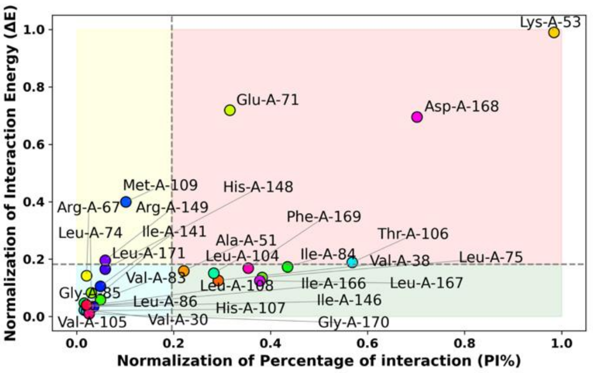

Holo-complexes

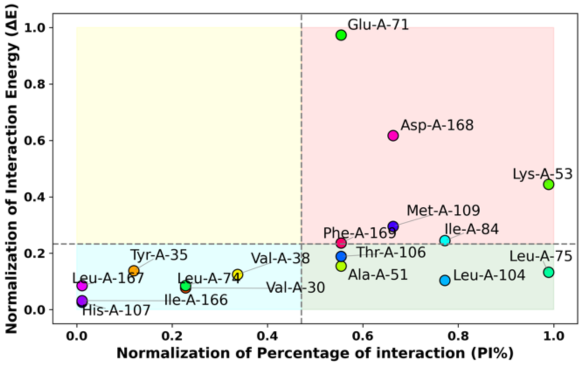

2D-FMOPhore heatmap

	Val-A-30	Tyr-A-35	Val-A-38	Ala-A-51	Lys-A-53	Glu-A-71	Leu-A-74	Leu-A-75	Ile-A-84	Leu-A-104	Thr-A-106	His-A-107	Met-A-109	Ile-A-166	Leu-A-167	Asp-A-168	Phe-A-169
	30	20	40	60	100	60	30	100	80	80	60	10	70	10	10	70	60
1WBN_L09_A_355	-1.8		-3.8	-5.2	-0.6	-19.5		-4.4	-8.6		-2.6		-8.1	-0.6		-10.9	-6.2
1WBT_WBT_A_355				-4.9	-10.7	-9.8	-2.1	-2.7	-7.1	-2.9	-6.5		-8.3			-12.0	-5.1
1W83_L11_A_355	-1.8		-4.1	-5.1	-0.9	-19.0	-2.6	-3.1	-7.3	-1.4			-7.0			-10.1	-8.1
1WBS_LI2_A_355				-4.3	-7.4	-12.0	-2.2	-2.7	-6.7	-2.0			-9.1			-13.2	-5.5
1W82_L10_A_356					8.6	-36.0		-4.2								-14.1	-8.3
1WBV_LI3_A_355					-5.6	-15.3		-4.2	-7.0	-2.1	-3.8					-12.2	-3.2
1WBW_LI4_A_355	-2.3	-4.6		-4.8	-10.0			-1.2	-3.0	-2.6	-4.6	-0.4	-10.5				
1W84_L12_A_356			-3.2	-4.6	-16.1			-1.5	-3.2	-3.3			-8.9		-2.6		
1WBO_2CH_A_355					-3.1			-1.0	-0.8	-2.3	-4.8					-22.6	
1W7H_3IP_A_355		-3.7	-2.9		-8.1			-1.2		-2.2	-3.0		-4.8				

Color scale: 30, 20, 10, 0, −10, −20, −30, −40, −50

2UDP-3-O-acyl-N-acetylglucosamine deacetylase (LpxC)

FP-score plot

Apo-structure

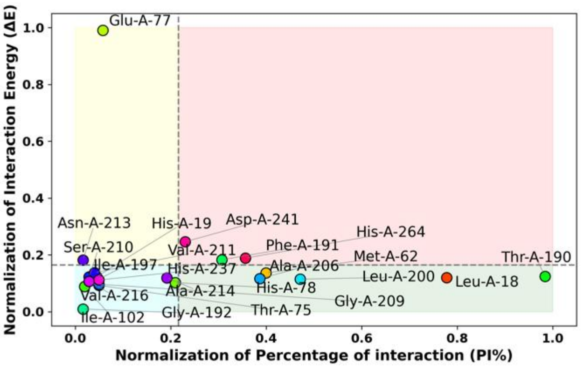

Holo-complexes

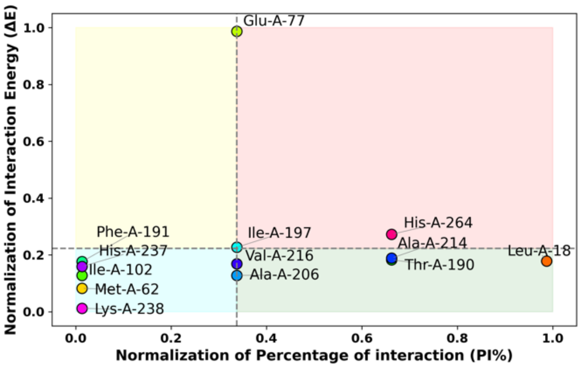

2D-FMOPhore heatmap

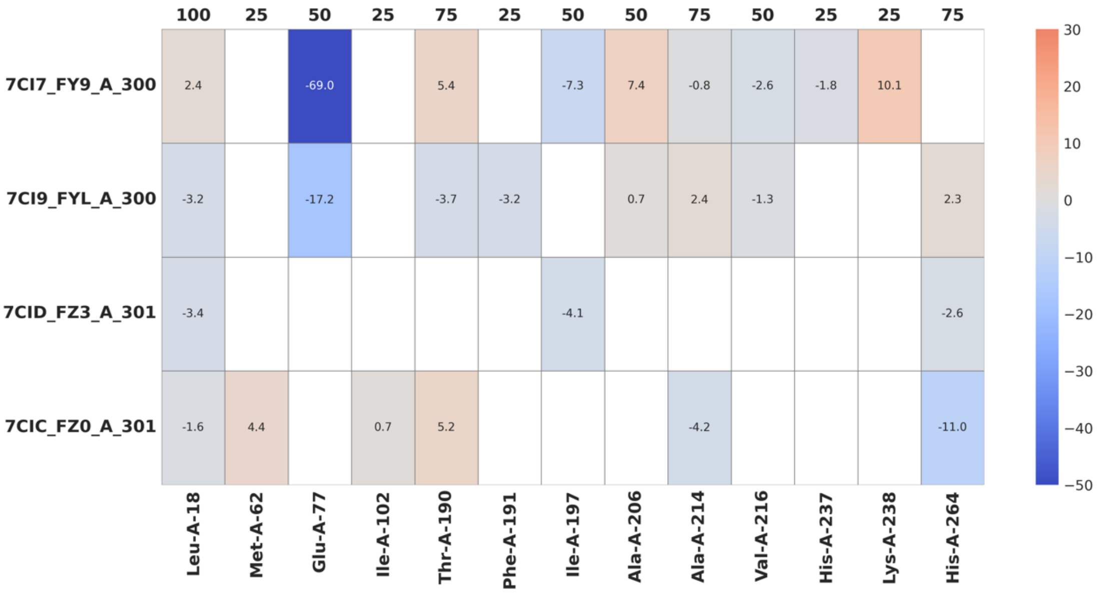

WD repeat-containing protein 5 (WDR5)

FP-score plot

Apo-structure

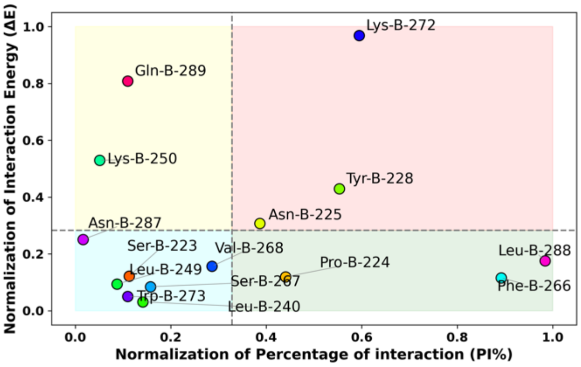

Holo-complexes

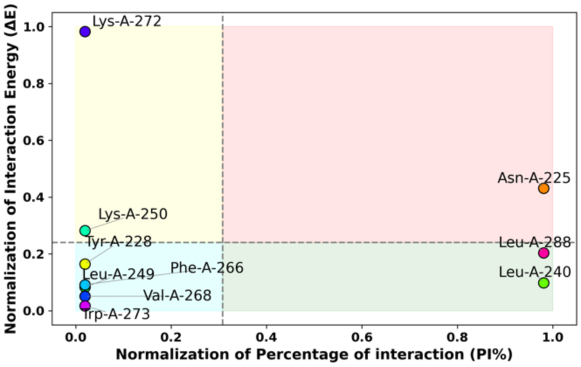

2D-FMOPhore heatmap

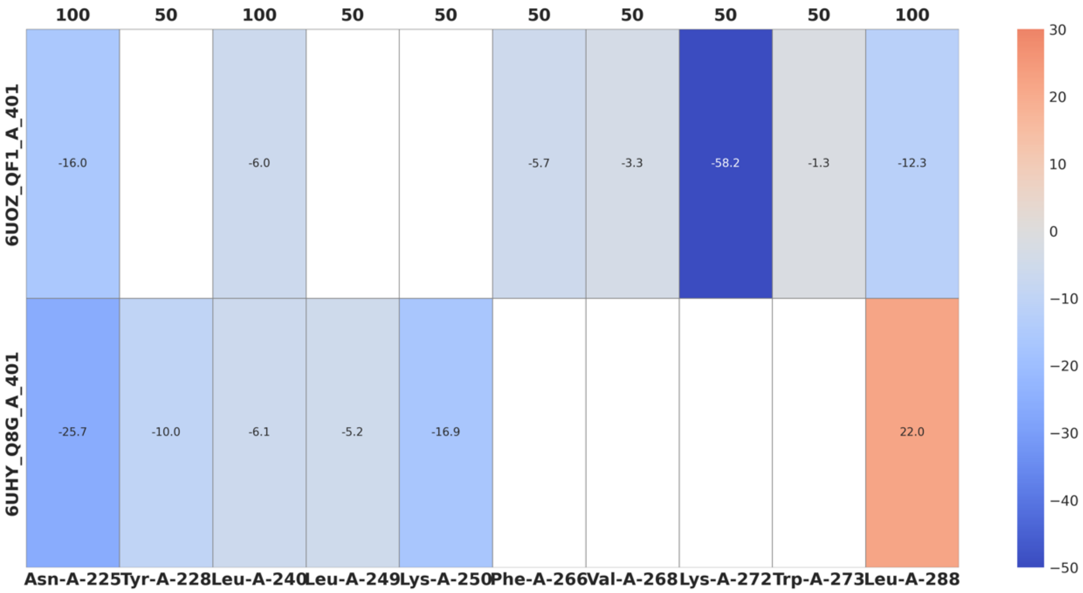

Tyrosine Phosphatase 1B (PTP1B)

FP-score plot

Apo-structure

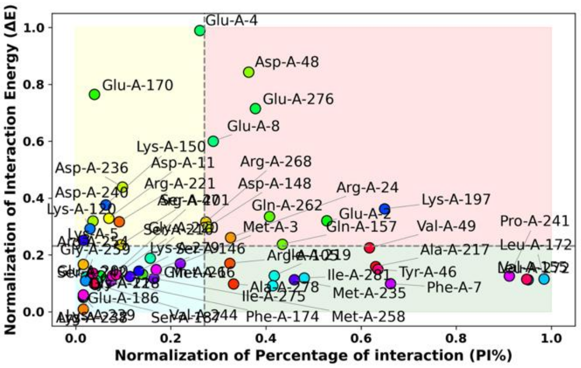

Holo-complexes

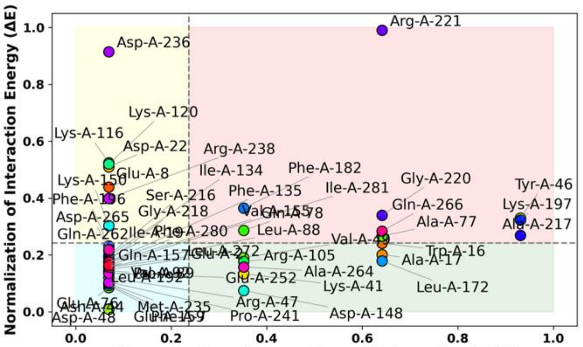

2D-FMOPhore heatmap

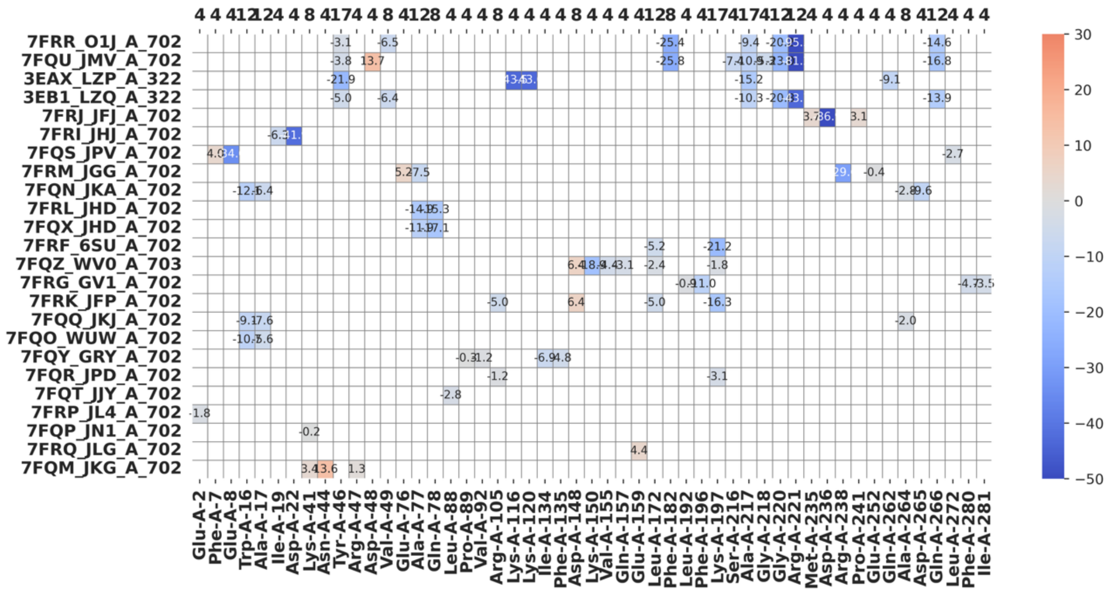

Metabotropic Glutamate Receptor 5 (mGlu5)

FP-score plot

Apo-structure

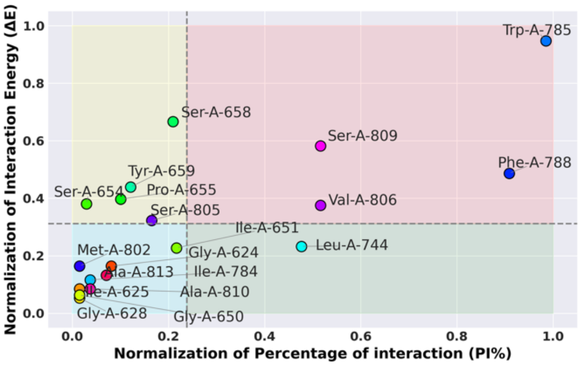

Holo-complexes

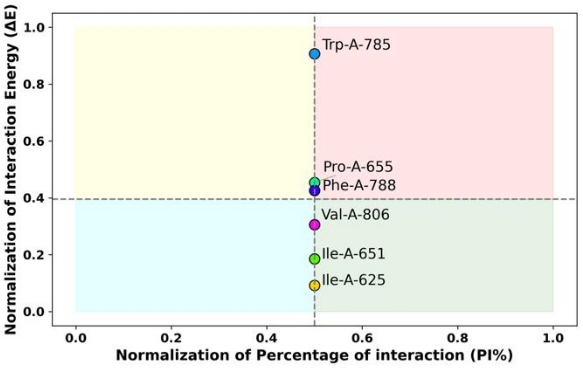

2D-FMOPhore heatmap

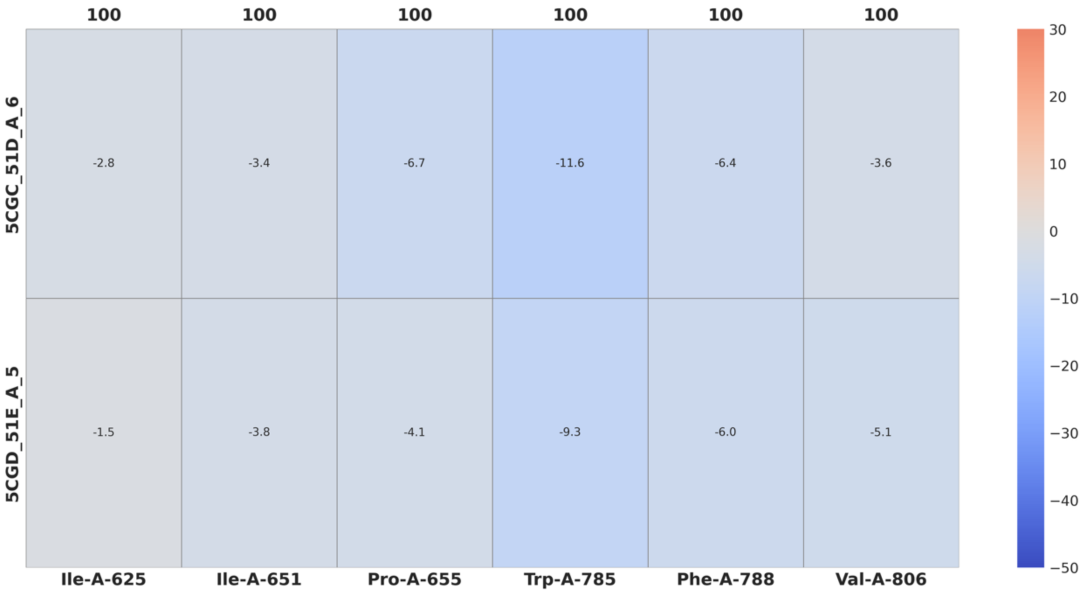

Mouse Double Minute 2 homolog (MDM2)

FP-score plot

Apo-structure

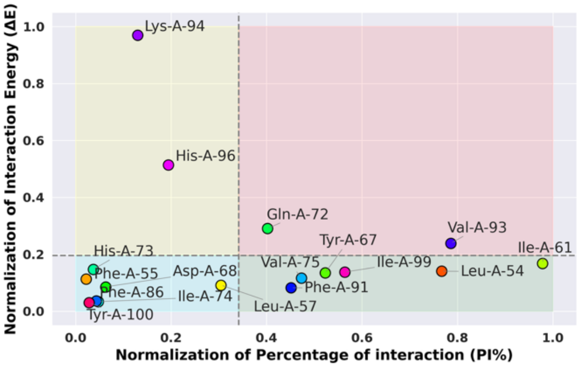

Holo-complexes

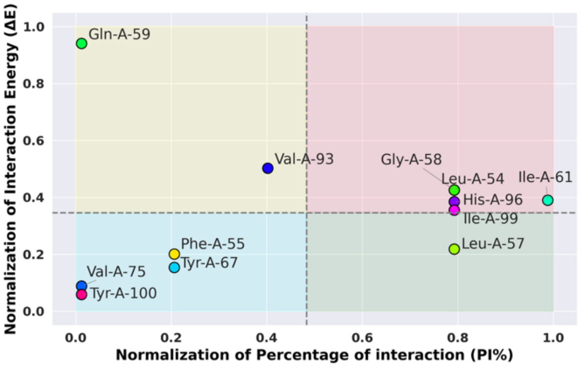

2D-FMOPhore heatmap

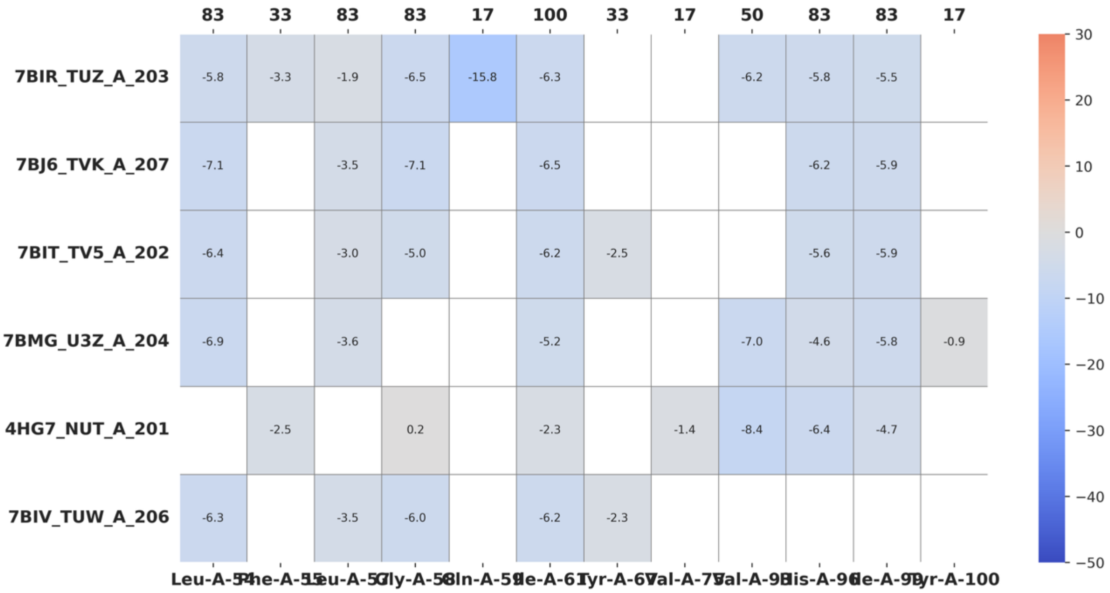

KRAS–Phosphodiesterase delta complex (KRAS_PDEδ)

FP-score plot

Apo-structure

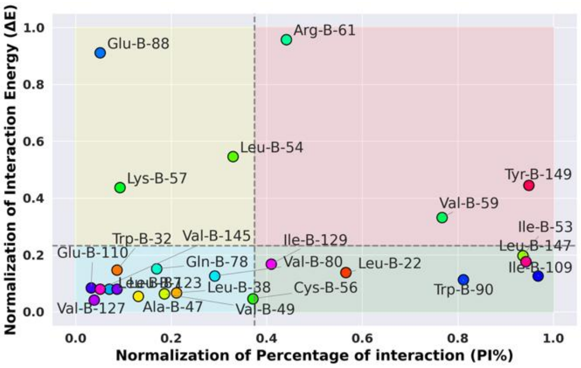

Holo-complexes

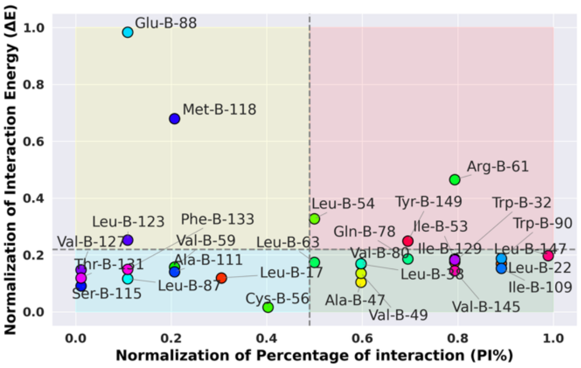

2D-FMOPhore heatmap

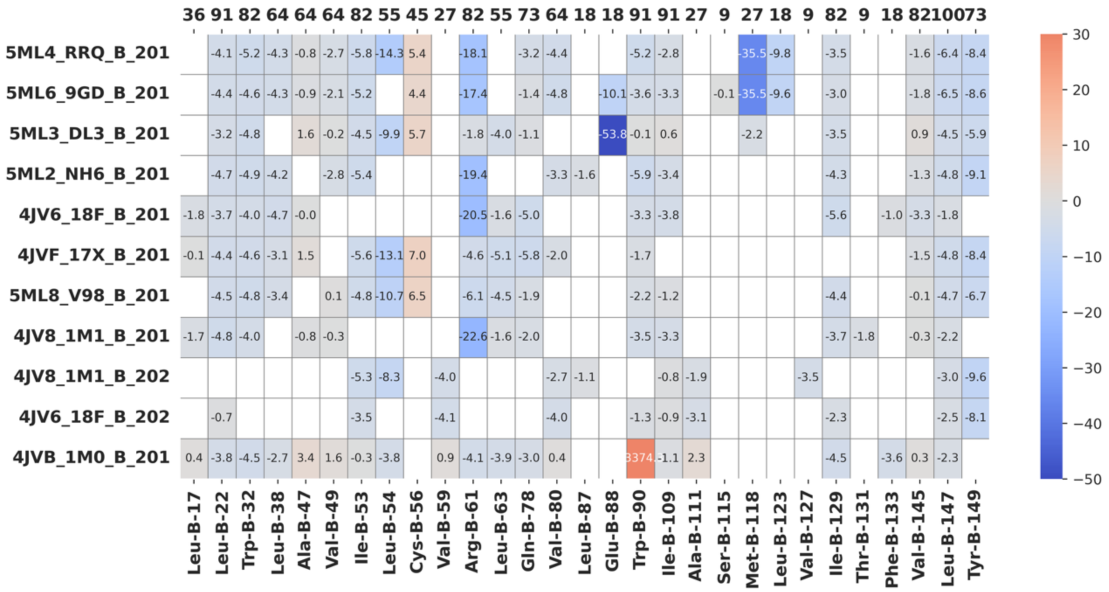

Human Carbonic Anhydrase II (hCAII)

FP-score plot

Apo-structure

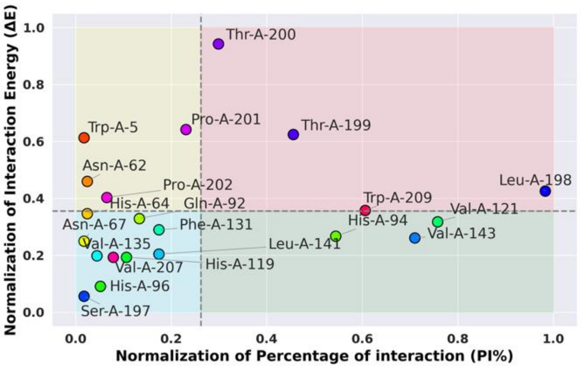

Holo-complexes

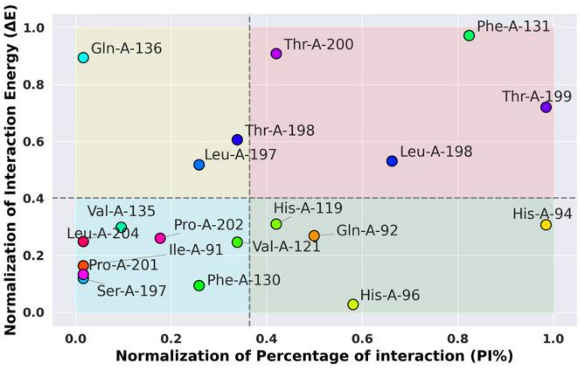

2D-FMOPhore heatmap

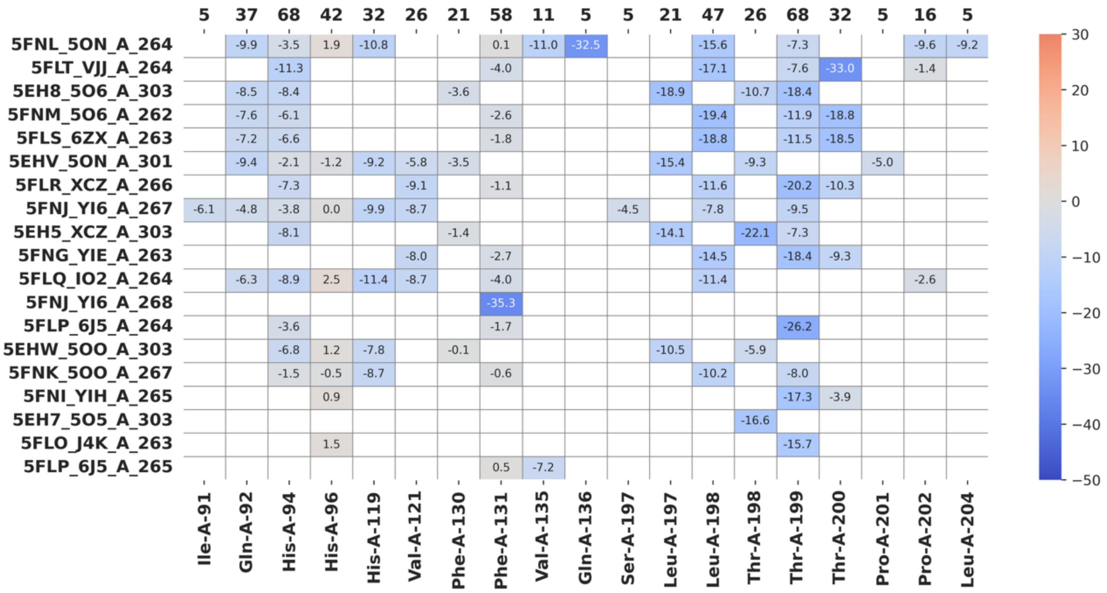

Histamine H1 Receptor (H1R)

FP-score plot

Apo-structure	Holo-complexes

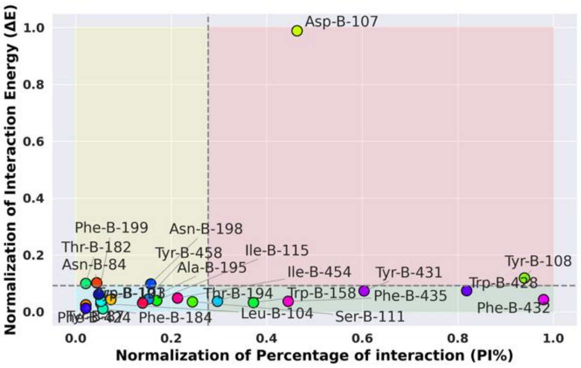

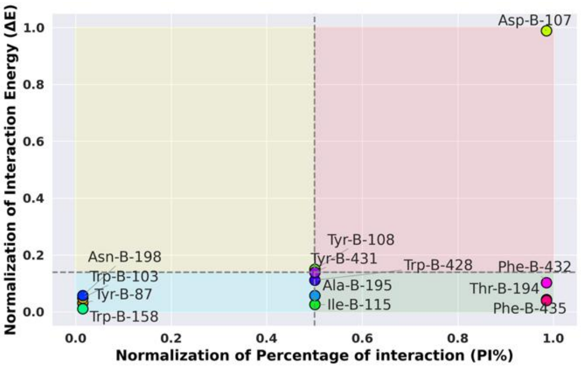

2D-FMOPhore heatmap

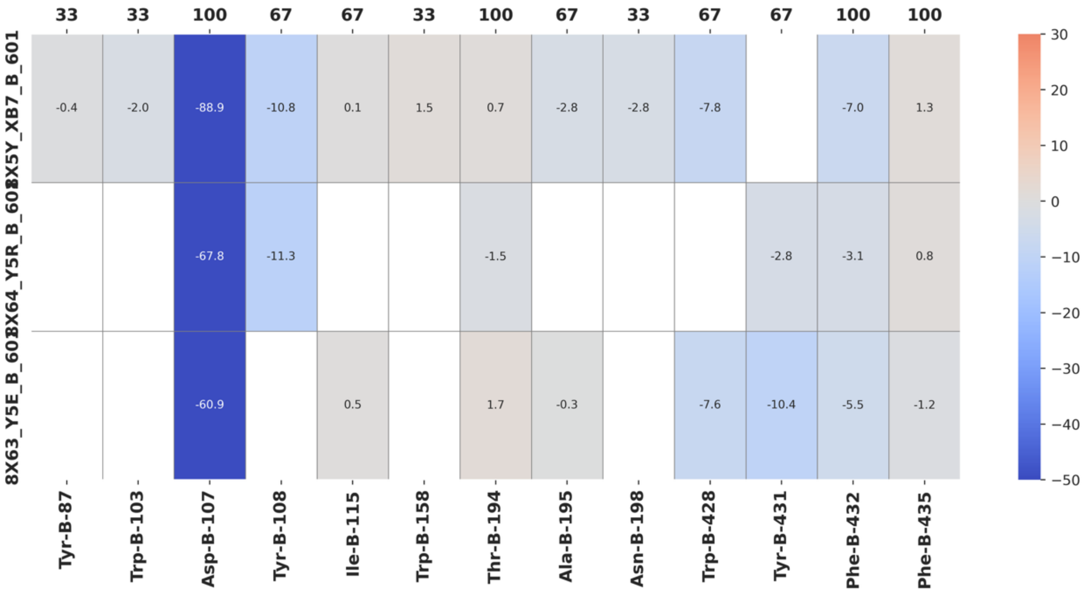

Class A Beta-Lactamase

FP-score plot

Apo-structure

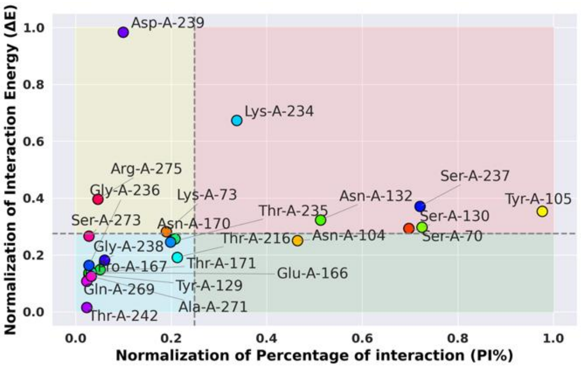

Holo-complexes

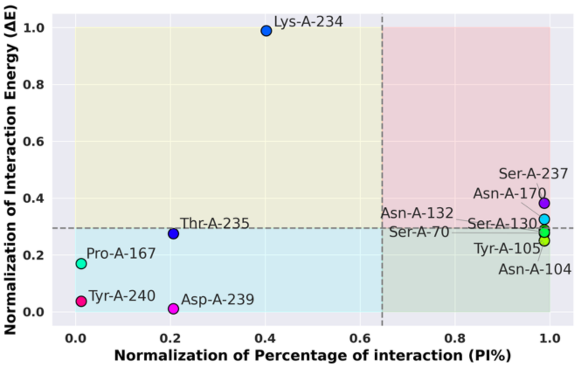

2D-FMOPhore heatmap

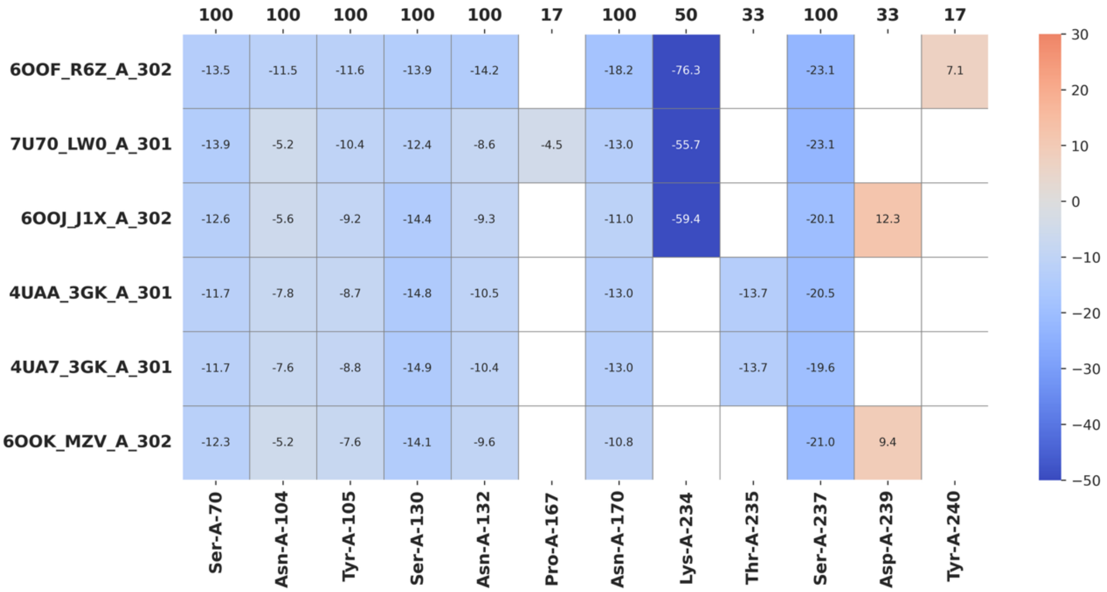

Cannabinoid Receptor 1 (CB1)

FP-score plot

Apo-structure

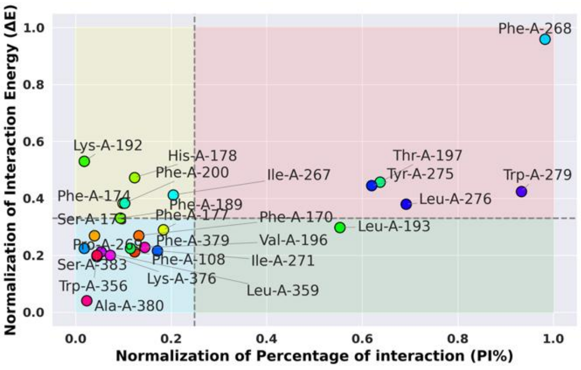

Holo-complexes

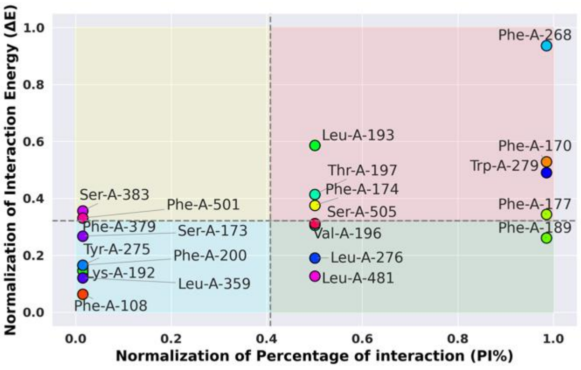

2D-FMOPhore heatmap

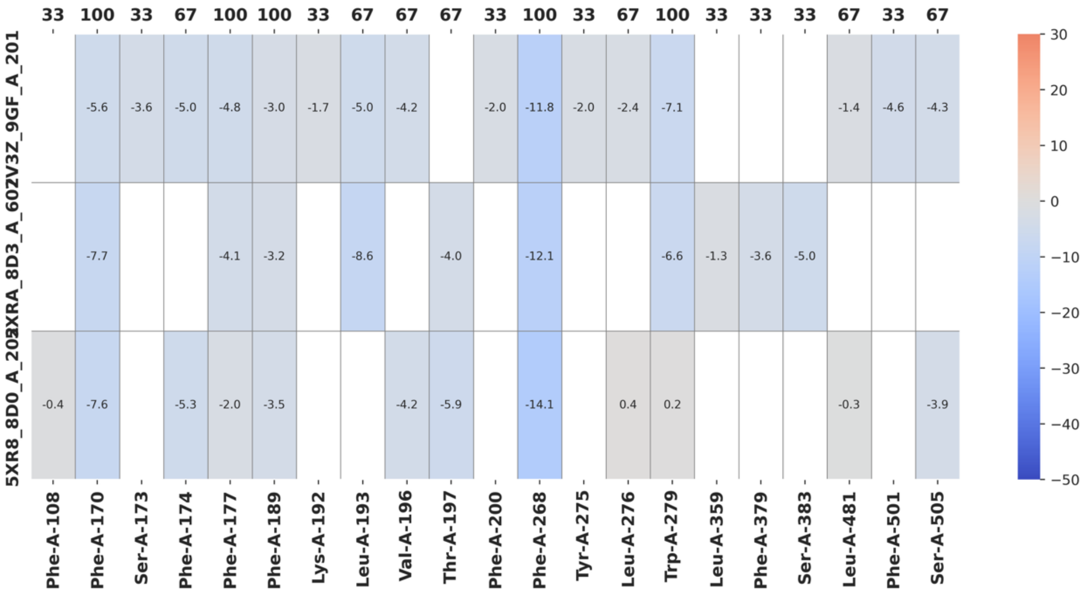

Beta-1 Adrenergic Receptor (β1AR)

FP-score plot

Apo-structure

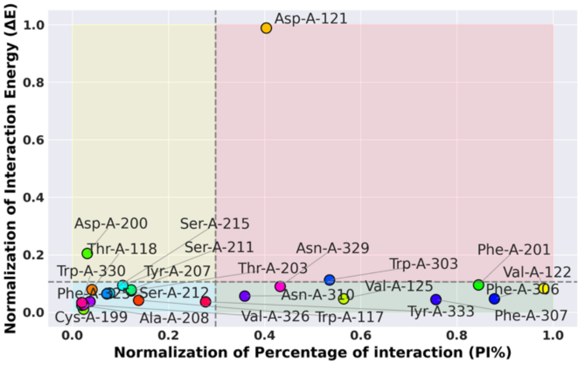

Holo-complexes

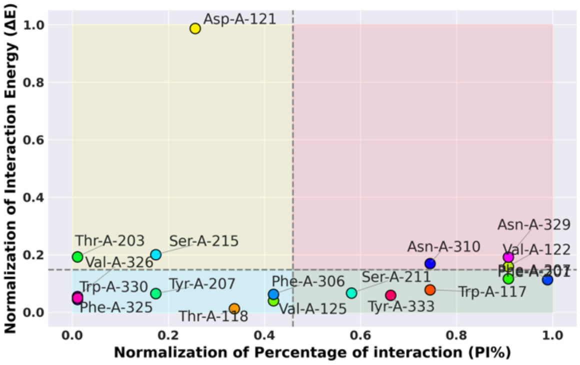

2D-FMOPhore heatmap

	Trp-A-117	Thr-A-118	Asp-A-121	Val-A-122	Val-A-125	Phe-A-201	Thr-A-203	Tyr-A-207	Ser-A-211	Ser-A-215	Phe-A-306	Phe-A-307	Asn-A-310	Phe-A-325	Val-A-326	Asn-A-329	Trp-A-330	Tyr-A-333
	77	38	31	92	46	92	8	23	62	23	46	100	77	8	8	92	8	69
2y02_WHJ_A_601	-2.7	-0.1	-50.9	-11.5	-2.2	-8.2			-4.3	-14.7	-2.7	-7.9	-12.3			-11.2		
2y01_Y00_A_601	-5.2		-75.9						1.1		0.6	-4.8	3.3				-3.0	
2y03_5FW_A_601	-3.0		-73.0	-2.3	4.8	1.1			0.8	3.2	-2.8	-5.2	4.9			-9.1		1.2
5f8u_P32_A_400	-1.8	1.1	-74.3	-1.4		3.7			8.9			-6.0	3.5			-9.1		
2ycw_CAU_A_400	-0.0			-3.6	4.6	-1.1		-4.2				-6.4				-5.9		0.1
2ycz_I32_A_400	-0.1			-0.3		-0.0			-0.5			-5.4	-0.2			-8.3		
2y04_68H_A_601	-1.1			-3.0		-4.1			4.4	4.9	-2.3	-4.7	3.9			-14.0		3.0
4ami_G90_A_359		1.3		-0.4	9.3	-2.9						-6.0	4.0	-2.6	-3.4	-9.4		-0.8
5abe_XTK_A_501	-1.6	0.1		-3.7	5.0	-2.9			6.6			-5.5	2.1			-4.6		-3.7
3zpq_XF5_A_359				-5.4		-0.5			3.1		-4.0	-5.7	4.1			-0.2		1.2
4amj_CVD_A_359	-2.5			-3.7	4.3	7.3		-4.1			-2.4	-5.7				-1.5		1.7
3zpr_3WC_A_500				-5.8		-1.0		-4.0				-2.4				19.8		-2.5

Color scale: 30, 20, 10, 0, −10, −20, −30, −40, −50

Beta-2 Adrenergic Receptor (β2AR)

FP-score plot

Apo-structure

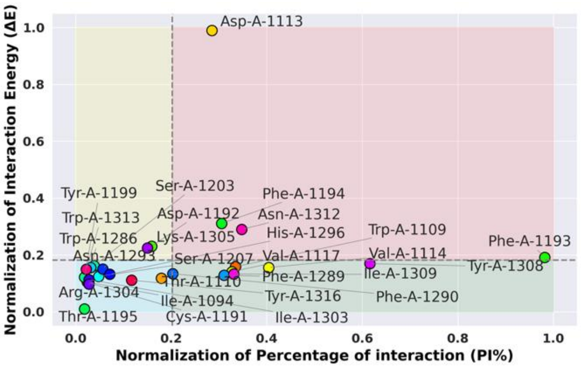

Holo-complexes

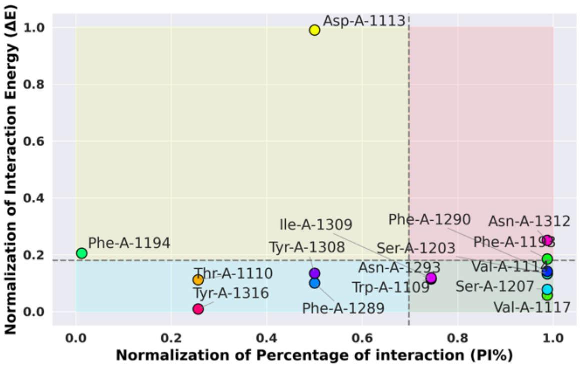

2D-FMOPhore heatmap

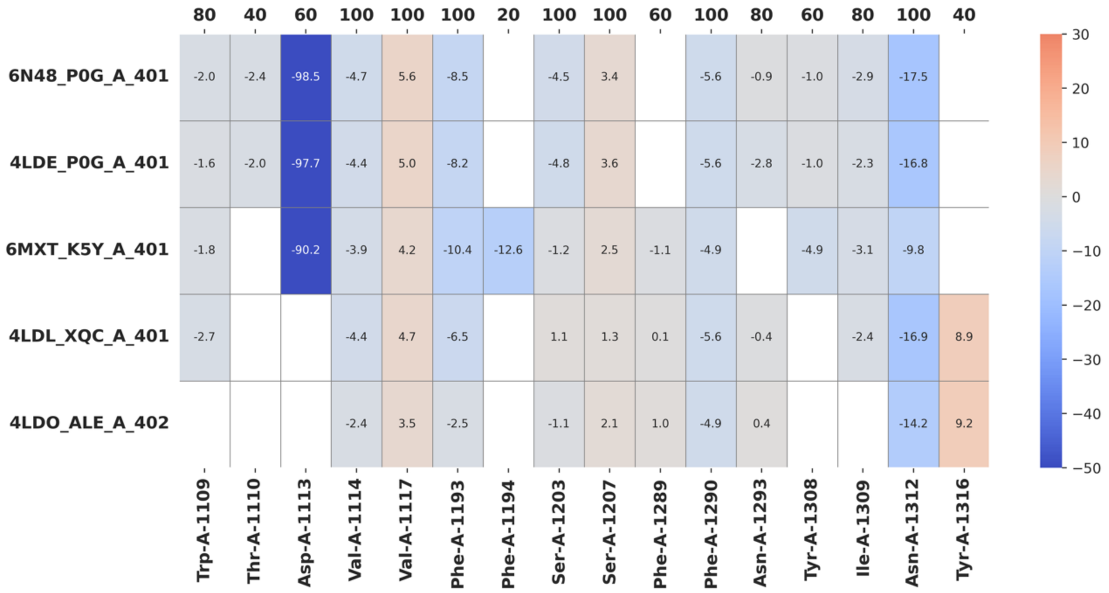

BCL-xL BH3 Domain Complex

FP-score plot

Apo-structure

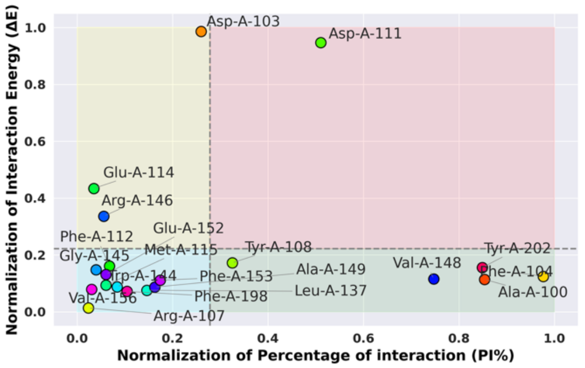

Holo-complexes

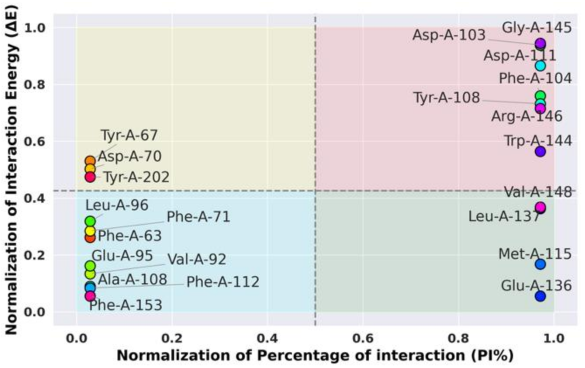

2D-FMOPhore heatmap

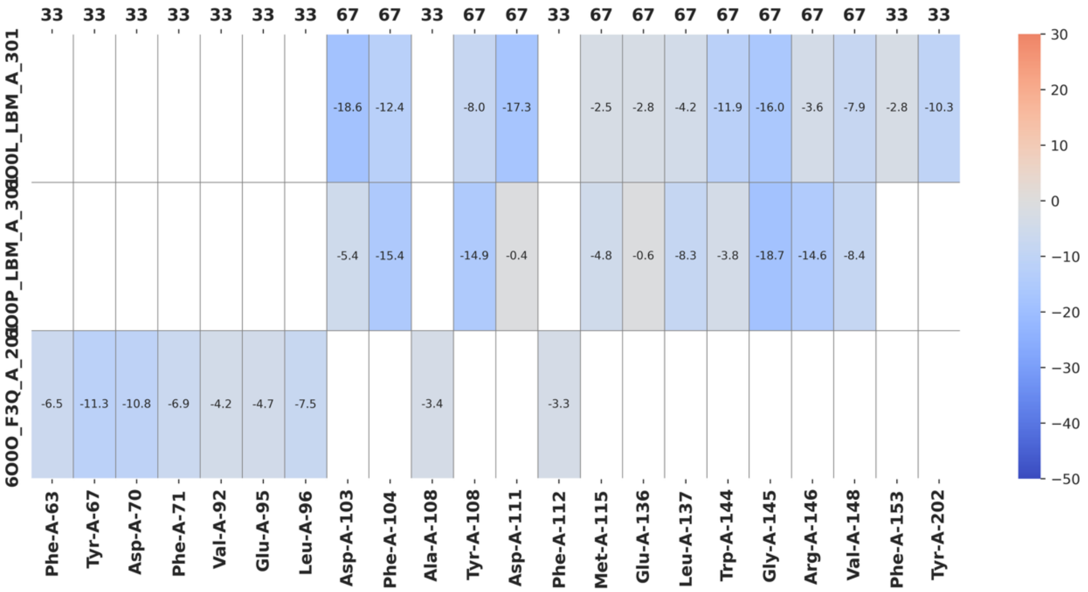

Adenosine A2A Receptor (A2AR)

FP-score plot

Apo-structure

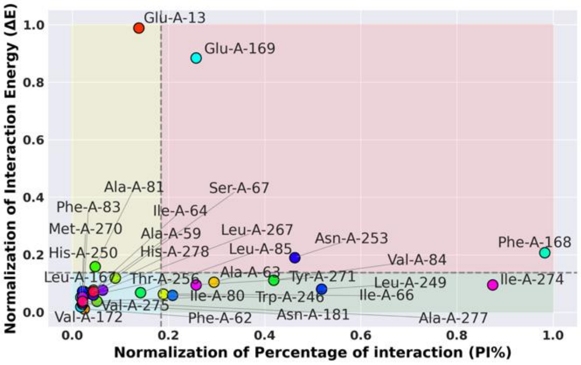

Holo-complexes

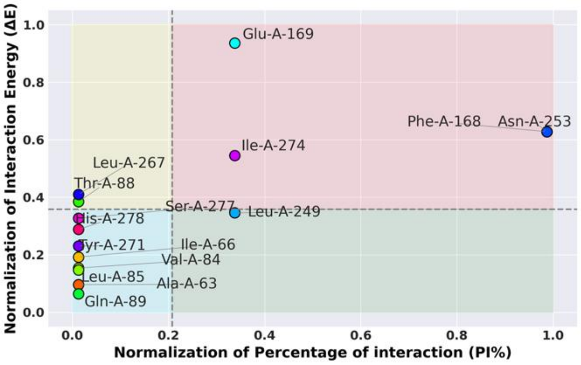

2D-FMOPhore heatmap

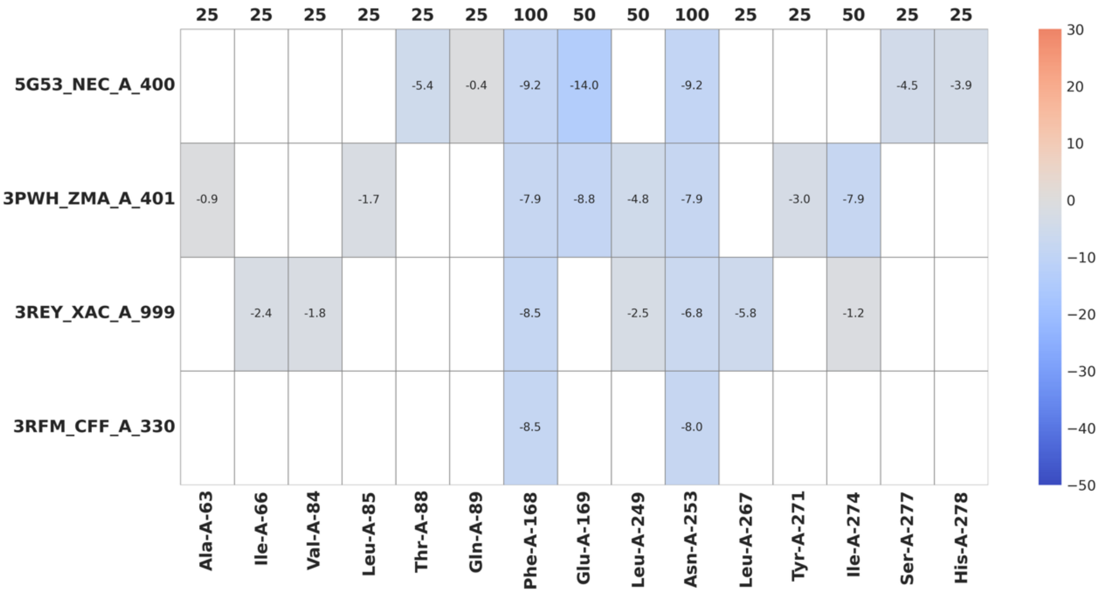

Adenosine A1 Receptor (A1R)

FP-score plot

Apo-structure	Holo-complexes
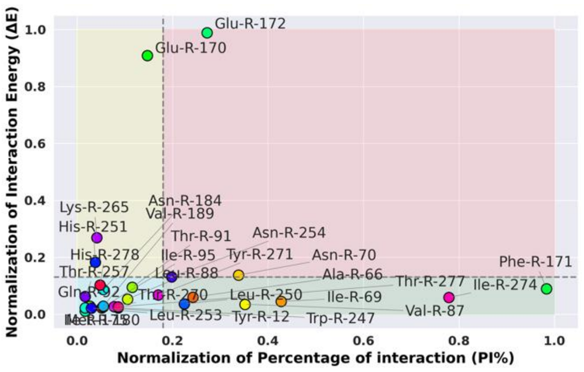	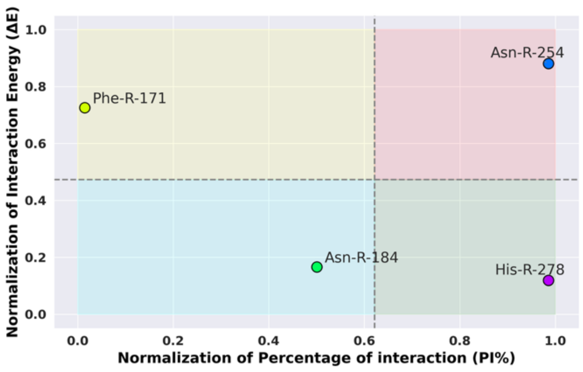

2D-FMOPhore heatmap

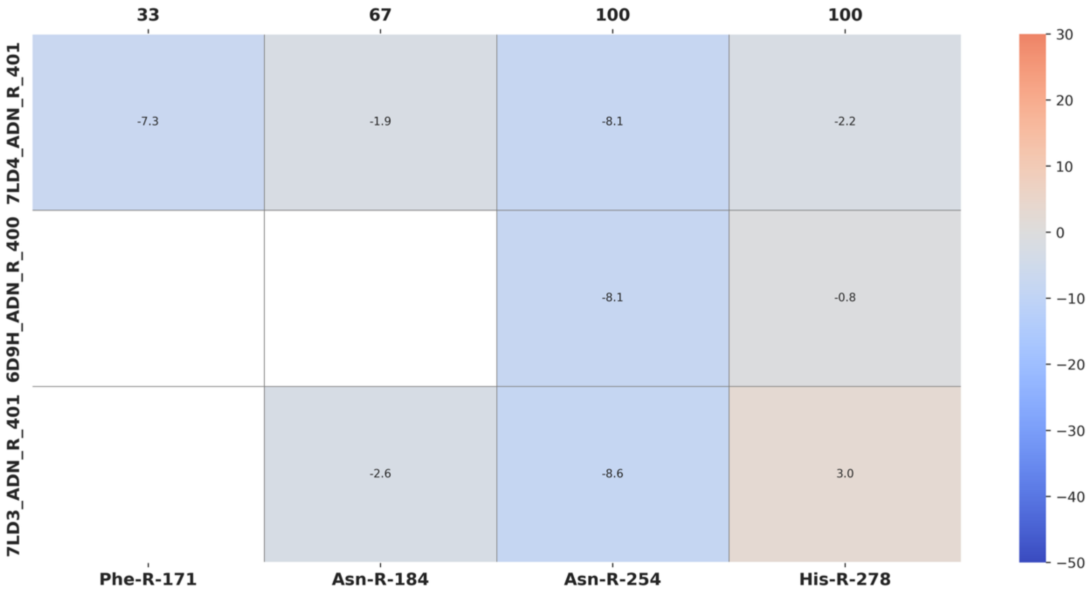

Proteasome β5 subunit (PSMB5)

FP-score plot	
Apo-structure	Holo-complexes
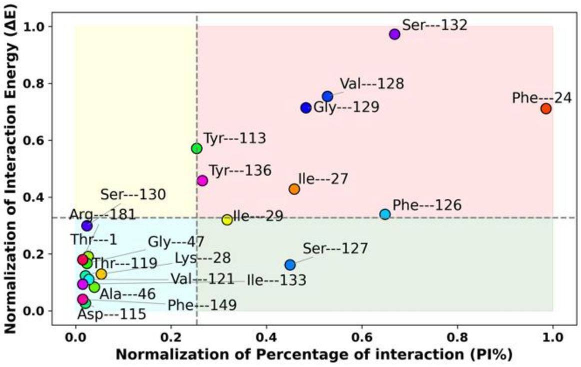	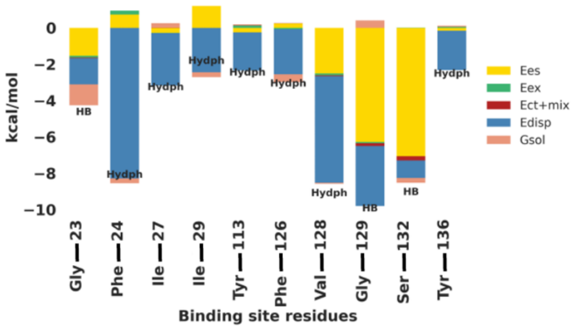

Note 2: Molecular dynamics simulations protocol for Dy-FMOPhore

To run *Dy-FMOPhore* protocol Supervised molecular dynamics (SuMD) (code availability: https://github.com/molecularmodelingsection/SuMD) was used to allow a whole ensemble sampling of different conformations of the binding site. All calculations were performed on a hybrid CPU/GPU cluster. SuMD simulations were carried out with the ACEMD engine, on a GPU cluster equipped with 16 GPUs (2 NVIDIA GTX 1080 per node on 8 GPUs).

System preparation

Protein structures for seven systems; M-pro, HIV, JAK-1, PI3K, PL-pro, HSP90 and Hepatitis-C, were prepared with the protein preparation wizard as implemented in Maestro,[4] for addition of hydrogen atoms and missing atoms in protein side chains were built according to the AMBER16 force field topology.[5]

Solvated system setup, equilibration, and production

Two types of simulations can be done for the *Dy-FMOPhore* protocol, holo-complex simulations and apo-structures simulations. In both simulations tleap was to assemble the system with and without ligand, used AMBER16SB as the force field for the proteins.[6] The systems were explicitly solvated by a cubic water box with cell borders placed at least 13 Å away from any protein or ligand atom using TIP3P as the water model.[7] To achieve charge neutrality, Na+ /Cl− counterions were added to the system, resulting in a final salt concentration of 0.15 M. The systems were then subjected to energy minimization for 1,000 steps using the conjugate-gradient method. Subsequently, a 50,000-step (100 ps) microcanonical ensemble (NVE) simulation was performed, maintaining a constant number of particles (N), constant volume (V), and constant energy (E). This was followed by a 1 ns simulation under isothermal-isobaric (NPT) conditions, with constant number of particles (N), constant pressure (P), and constant temperature (T). A time step of 2 fs was employed, with harmonic positional constraints applied to the protein and ligand atoms, gradually reduced using a scaling factor of 0.1. The system pressure was maintained at 1 atm using a Berendsen barostat, while temperature control

was achieved through a Langevin thermostat with a low damping constant of 1 ps. Bond lengths involving hydrogen atoms were constrained via the M-SHAKE algorithm. For the production molecular dynamics (MD) runs, simulations were conducted in an NVT ensemble (constant number of particles, constant volume, and constant temperature). Long-range Coulomb interactions were handled using the particle mesh Ewald (PME) method, with a mesh spacing of 1.0 Å.[8-11]

Clustering using DBSCAN.

Following the concatenation of SuMD trajectories, geometric clustering was performed using the DBSCAN algorithm.[12] The density-based clustering method enables the identification of distinct clusters representing similar conformations within the combined trajectory data. DBSCAN efficiently distinguishes densely populated binding site conformations with and without ligand bound, from sparse background noise. Each identified cluster comprises a collection of MD snapshots (frames) from the trajectory. *Dy-FMOPhore* analysis for the holo-complex system was conducted throughout the molecular dynamic trajectory, on 52 snapshots. While for the apo-structures, the concept is to have different binding site conformations therefore only few snapshots of different binding site conformations (cluster representatives), along with the initial static crystal structure conformations are scanned with the probes library using Docking Glide (as mentioned in the article; Apo-structure scenario) [13-15], and average *FP-score* is calculated between the different snapshots.

Dy-FMOPhore analysis of PDB-ID: 7S3S

Protein RMSD

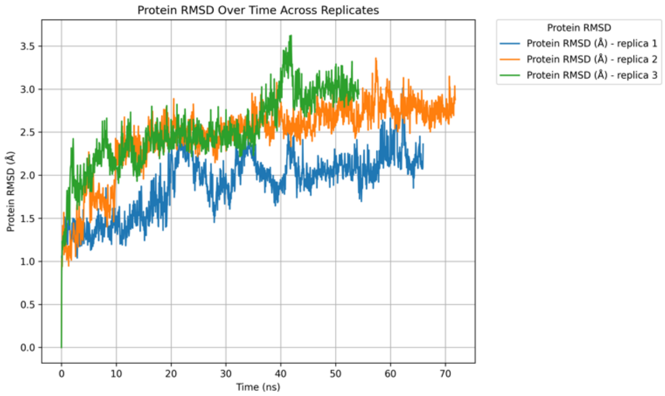

Protein RMSF

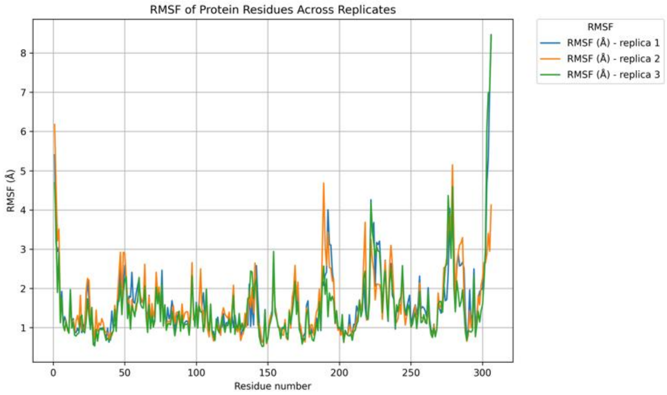

Ligand RMSD

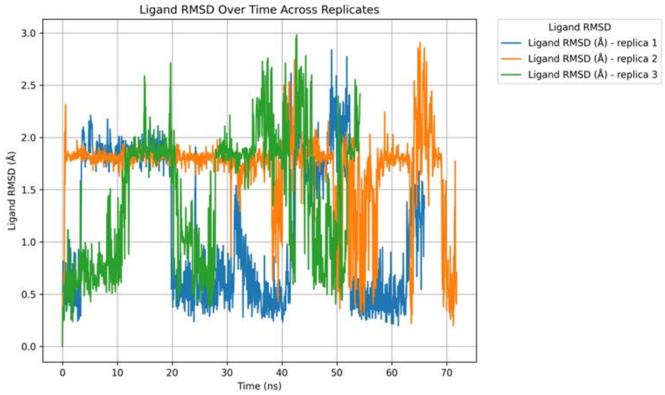

Figure S1: The 3 replicates of SuMD simulation for each Mpro PDB-ID 7S3S.

Simulation box dimensions	Total number of atoms	Total number of water molecules	Salt concentration	Lipid composition
X = 145.059<br>Y = 160.751<br>Z = 158.942	53572	16284	0.15M	0

Proteasome β5 subunit

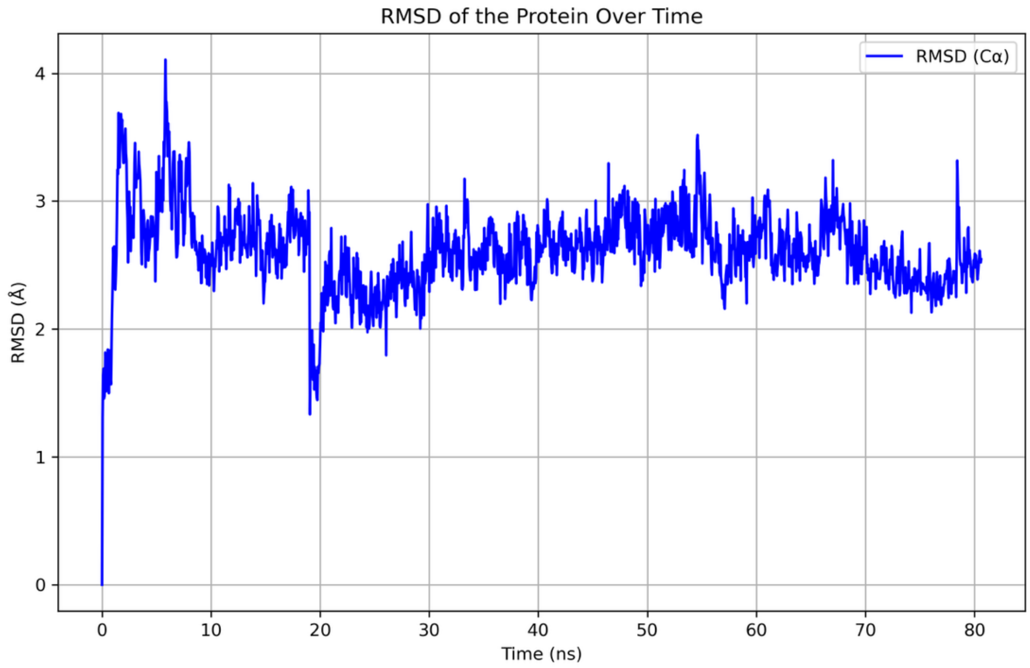

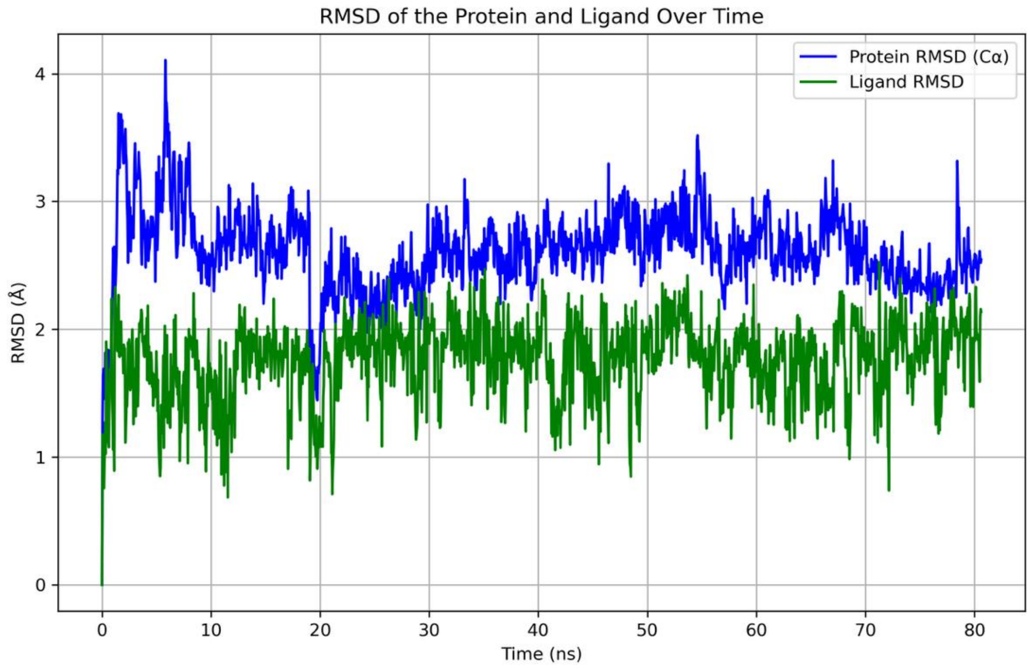

Protein RMSF

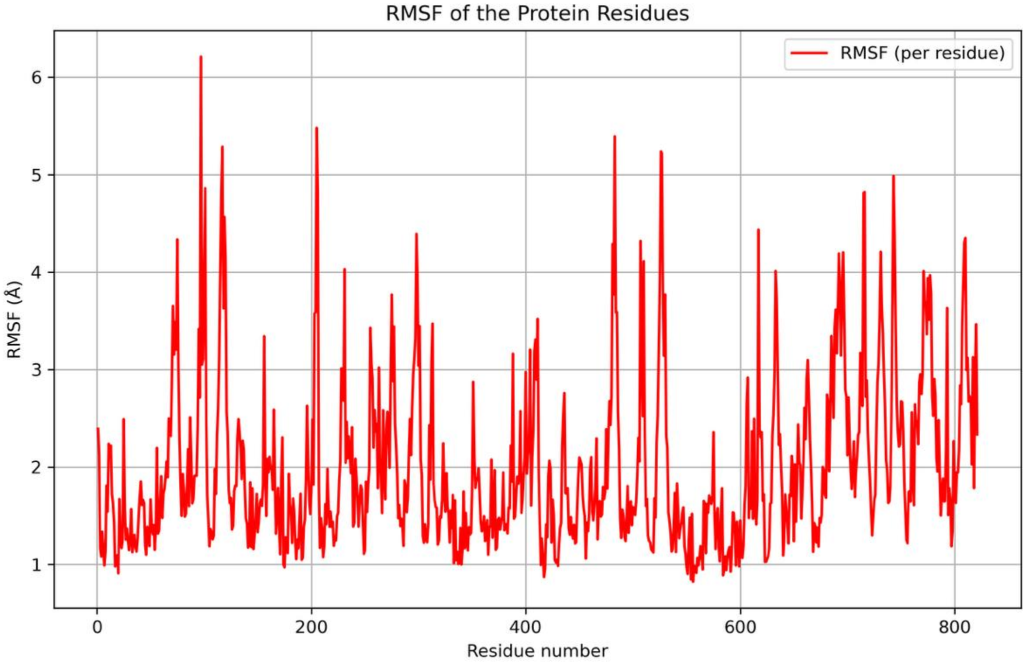

Figure S8: The SuMD simulation for each holo-structure of Proteasome β5 subunit in complex with compound 7a.

Simulation box dimensions	Total number of atoms	Total number of water molecules	Salt concentration	Lipid composition
X = 177.548<br>Y = 190.817<br>Z = 156.902	133722	39919	0.15M	0

Dy-FMOPhore analysis Systems

Hepatitis-C virus (HCV NS5b RNA polymerase)

Protein RMSD

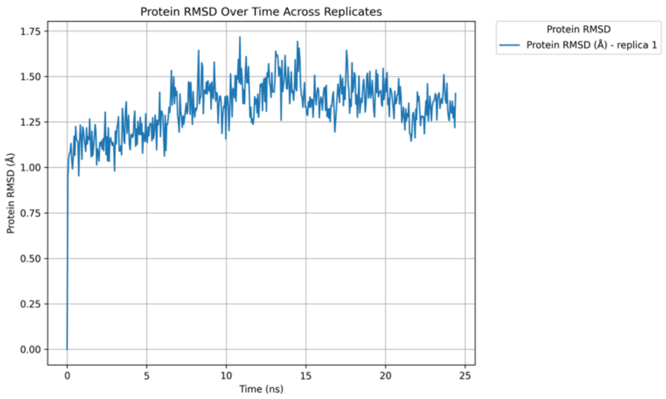

Protein RMSF

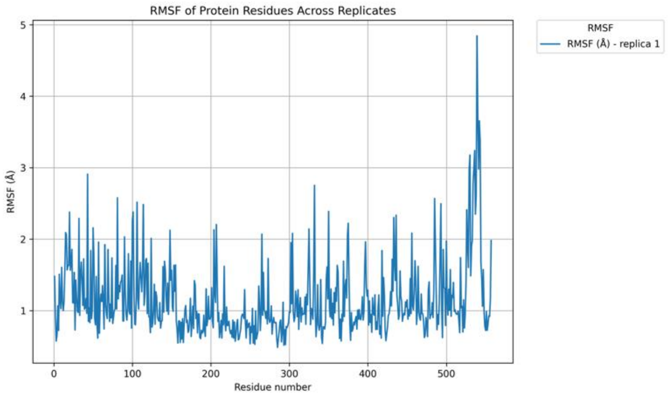

Figure S2: The SuMD simulation for each apo-structure of Hepatitis-C virus (HCV NS5b RNA polymerase).

Simulation box dimensions	Total number of atoms	Total number of water molecules	Salt concentration	Lipid composition
X = 159.447<br>Y = 160.872<br>Z = 165.321	82817	24613	0.15M	0

Human Immunodeficiency Virus protease (HIV-1)

Protein RMSD

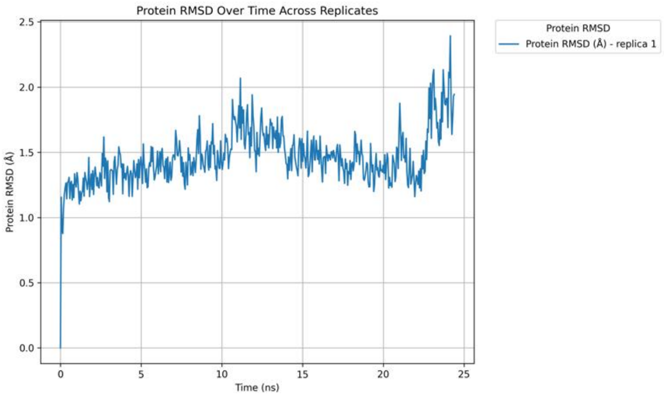

Protein RMSF

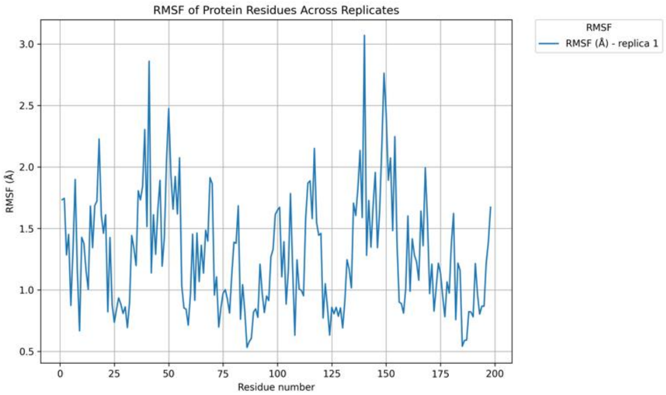

Figure S3: The SuMD simulation for each apo-structure of Human Immunodeficiency Virus protease (HIV-1).

Simulation box dimensions	Total number of atoms	Total number of water molecules	Salt concentration	Lipid composition
**X = 144.450**<br>**Y = 152.609**<br>**Z = 152.123**	**39318**	**11976**	**0.15M**	**0**

Heat shock protein (HSP90)

Protein RMSD

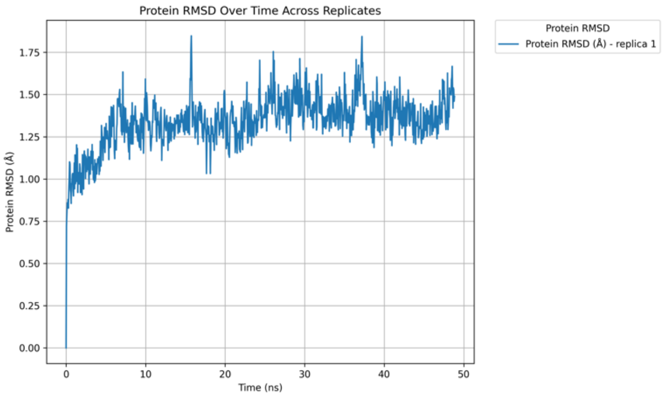

Protein RMSF

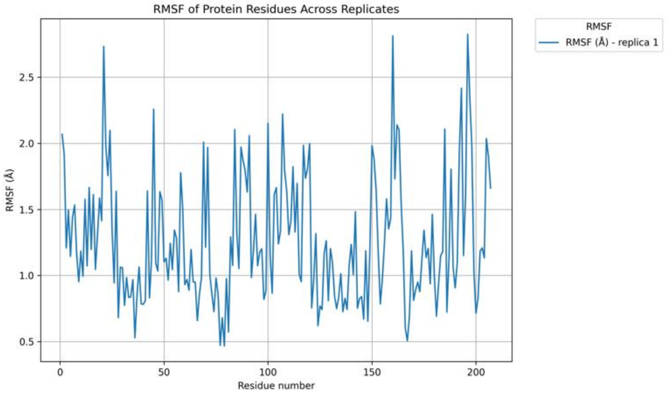

Figure S4: The SuMD simulation for each apo-structure of Heat shock protein (HSP90)

Simulation box dimensions	Total number of atoms	Total number of water molecules	Salt concentration	Lipid composition
**X = 147.021**<br>**Y = 145.799**<br>**Z = 150.926**	**34867**	**10449**	**0.15M**	**0**

Janus Kinase family (JAK-1)

Protein RMSD

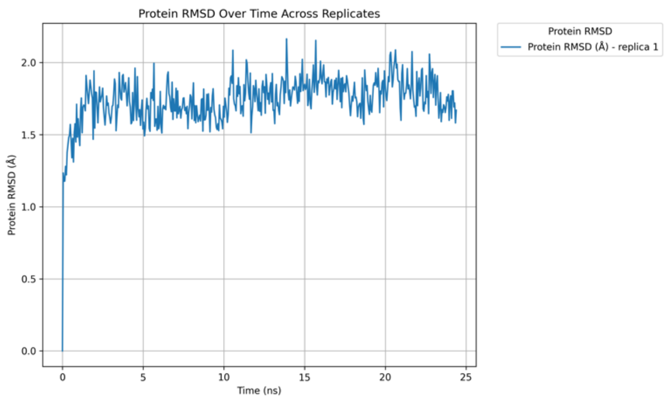

Protein RMSF

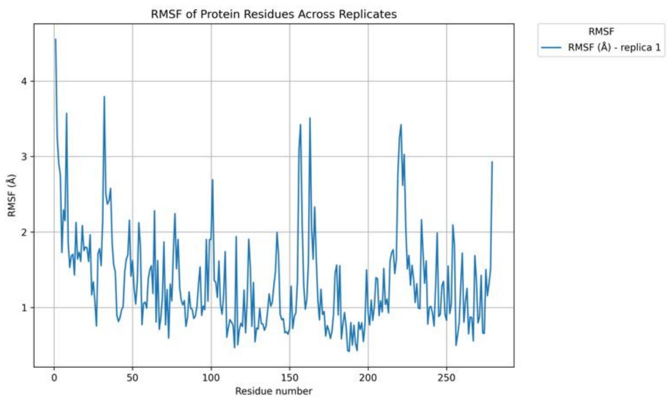

Figure S5: The SuMD simulation for each apo-structure of Janus Kinase family (JAK-1).

Simulation box dimensions	Total number of atoms	Total number of water molecules	Salt concentration	Lipid composition
X = 154.717<br>Y = 151.576<br>Z = 151.332	48594	14686	0.15M	0

Main Protease Protein (Mpro)

Protein RMSD

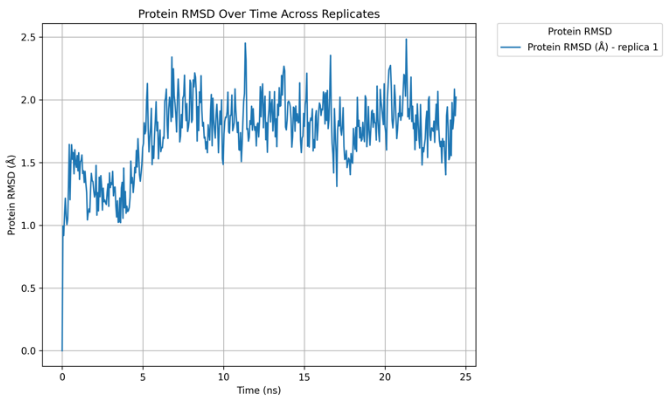

Protein RMSF

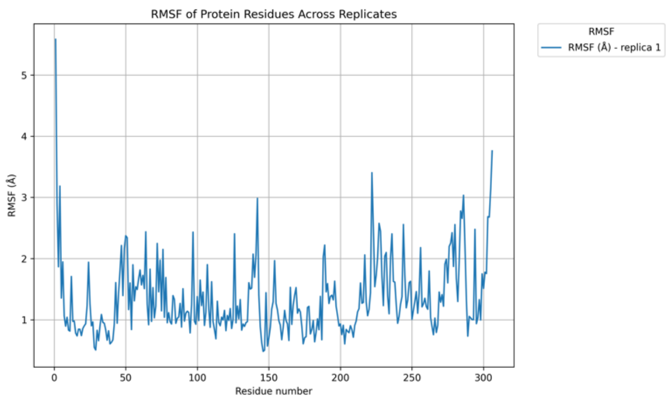

Figure S6: The SuMD simulation for each apo-structure of Main Protease Protein (Mpro).

Simulation box dimensions	Total number of atoms	Total number of water molecules	Salt concentration	Lipid composition
X = 145.054<br>Y = 159.859<br>Z = 159.388	53141	16151	0.15M	0

Phosphoinositide 3-kinase R (PI3KR)

Protein RMSD

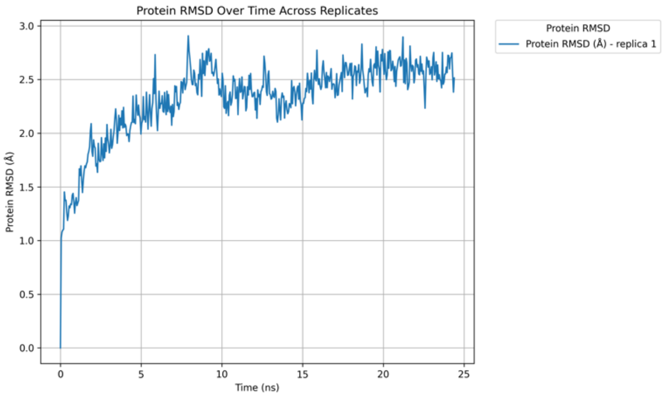

Protein RMSF

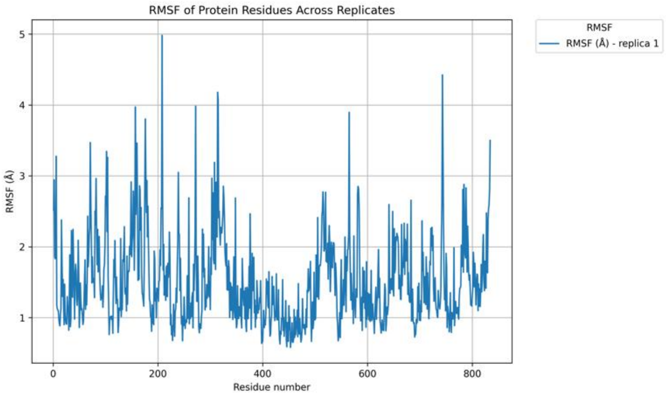

Figure S7: The SuMD simulation for each apo-structure of Phosphoinositide 3-kinase R (PI3KR).

Simulation box dimensions	Total number of atoms	Total number of water molecules	Salt concentration	Lipid composition
**X = 176.396**<br>**Y = 166.609**<br>**Z = 164.020**	**13222**	**33212**	**0.15M**	**0**

Papain-like protease (PL-pro)

Protein RMSD

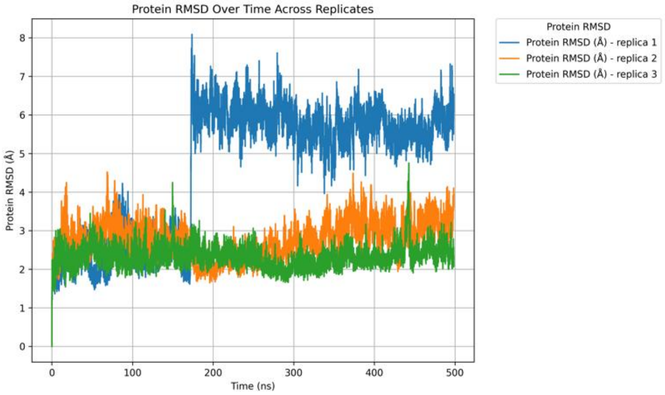

Protein RMSF

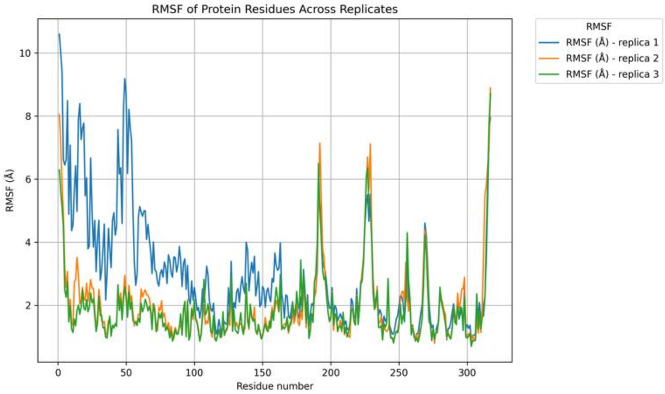

Figure S8: The SuMD simulation for each apo-structure of Papain-like protease (PL-pro).

Simulation box dimensions	Total number of atoms	Total number of water molecules	Salt concentration	Lipid composition
X = 161.294<br>Y = 159.651<br>Z = 170.823	89003	28000	0.15M	0

For animation and videos supplementary to the opening and closing of the P-loop binding site of the Pl-pr, please visit the supplementary section in https://onlinelibrary.wiley.com/doi/10.1002/jcc.27370 [16]

Supplementary Figures

Figure 1: FMOPhore analysis on two M-pro Holo-complex structures, PDB-IDs: 7N8C and 7S3S

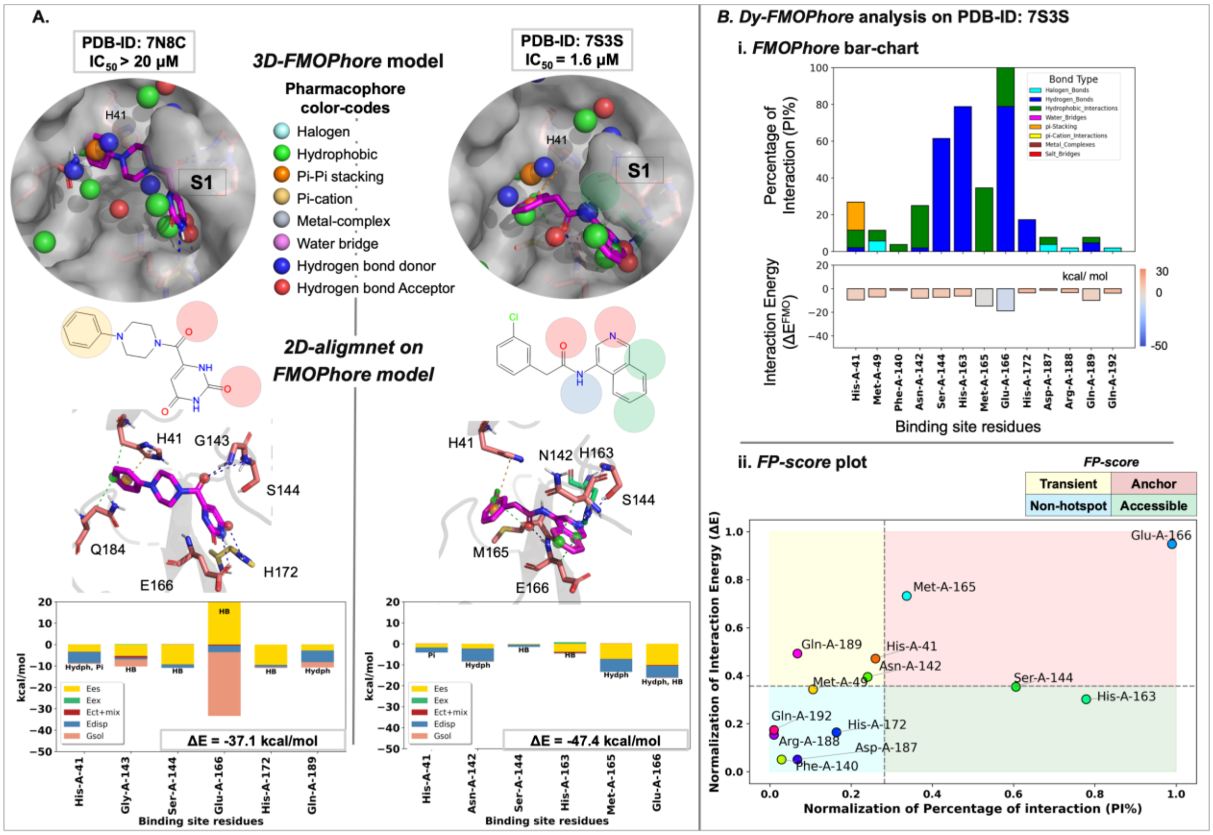

A. Bar-chart showing interaction bond type and energy per function group versus binding site residues. Binding site residues colored according to *FP-score* categories and the ligands colored magenta. *FP-score* prioritized *3D-FMOPhore* model shows overlapping of function groups with pharmacophoric features of the ligands. Binding site residues are colored according to *FP-score* categories by *FMOPhore* analysis on the holo-complexes.

B. *Dy-FMOPhore analysis on M-pro Holo-complex PDB-ID: 7S3S:*

   i. *FMOPhore* bar-chart showing the percentage of interaction between residues and ligand during complete Molecular dynamics trajectory, with interaction energy and type of bonds to be formed.

   ii. *FP-score* plot categorizing the binding site residues, normalization of Interaction energies on the y-axis, and normalization of percentage of interaction on the x-axis.

Figure 2: FMOPhore analysis of Biotin carboxylase

A. FP-score plot

i. Apo-structure | **ii. Holo-complexes**

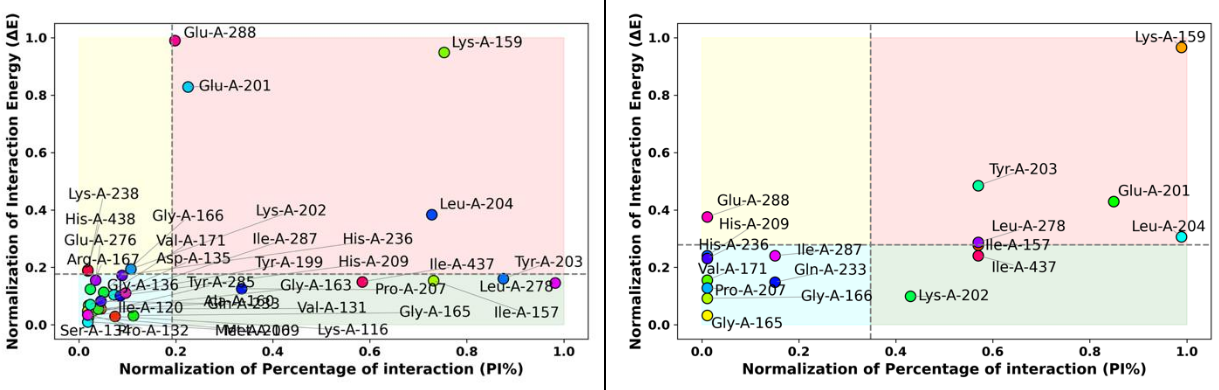

B. 2D-FMOPhore heatmap

	Ile-A-157 (62)	Lys-A-159 (100)	Gly-A-165 (12)	Gly-A-166 (12)	Val-A-171 (12)	Glu-A-201 (88)	Lys-A-202 (50)	Tyr-A-203 (62)	Leu-A-204 (100)	Pro-A-207 (12)	His-A-209 (12)	Gln-A-233 (25)	His-A-236 (12)	Leu-A-278 (62)	Ile-A-287 (25)	Glu-A-288 (12)	Ile-A-437 (62)
2W6N_OA2_A_446	-4.2	-24.4			-2.4	-6.1		-11.2	-6.4					-4.6			-5.0
2W71_L23_A_448		-27.0				-1.9	-0.7	-8.2	-6.8			-2.2		-6.1	-5.0		-4.2
2W6Q_OA5_A_447	-4.8	-19.0				-1.5	-0.6	-12.4	-5.2				-4.7				-2.4
2W6M_OA1_A_447	-3.6	-14.2				-10.7		-10.5	-7.0								-2.1
2W70_L22_A_447		-19.4	1.3	-0.5		-5.0	-0.6		-5.9					-5.0		-9.1	
2W6Z_L21_A_447	-6.1	-17.0				-3.4		-11.7	-4.8								
2W6P_OA4_A_447		-15.2				-2.0	-0.3		-4.6	-1.6	-5.0	-2.2		-6.4	-4.9		
2W6O_OA3_A_446	-4.6	-13.0							1.6					-3.9			-2.5

Color scale: 30, 20, 10, 0, −10, −20, −30, −40, −50

A. *FP-score* plot categorizing the binding site residues, normalization of Interaction energies on the y-axis, and normalization of percentage of interaction on the x-axis. i. Apo-structure. ii. Holo-complexes.

B. *2D-FMOPhore heatmap. The binding site residues on the bottom x-axis, and the percentage of interaction (%) on the top x-axis. PDB-IDs representing the ligands of the complex (PDB-D_Ligand-name_Chain-ID_Ligand-Number) on the y-axis (left side), and the color code of each cell corresponds to the interaction energy values y-axis (right side), (kcal/mol) between the function group on the ligands and the binding site residue.*

Figure 3: FMOPhore for Hotspot identification in the Proteasome β5 subunit

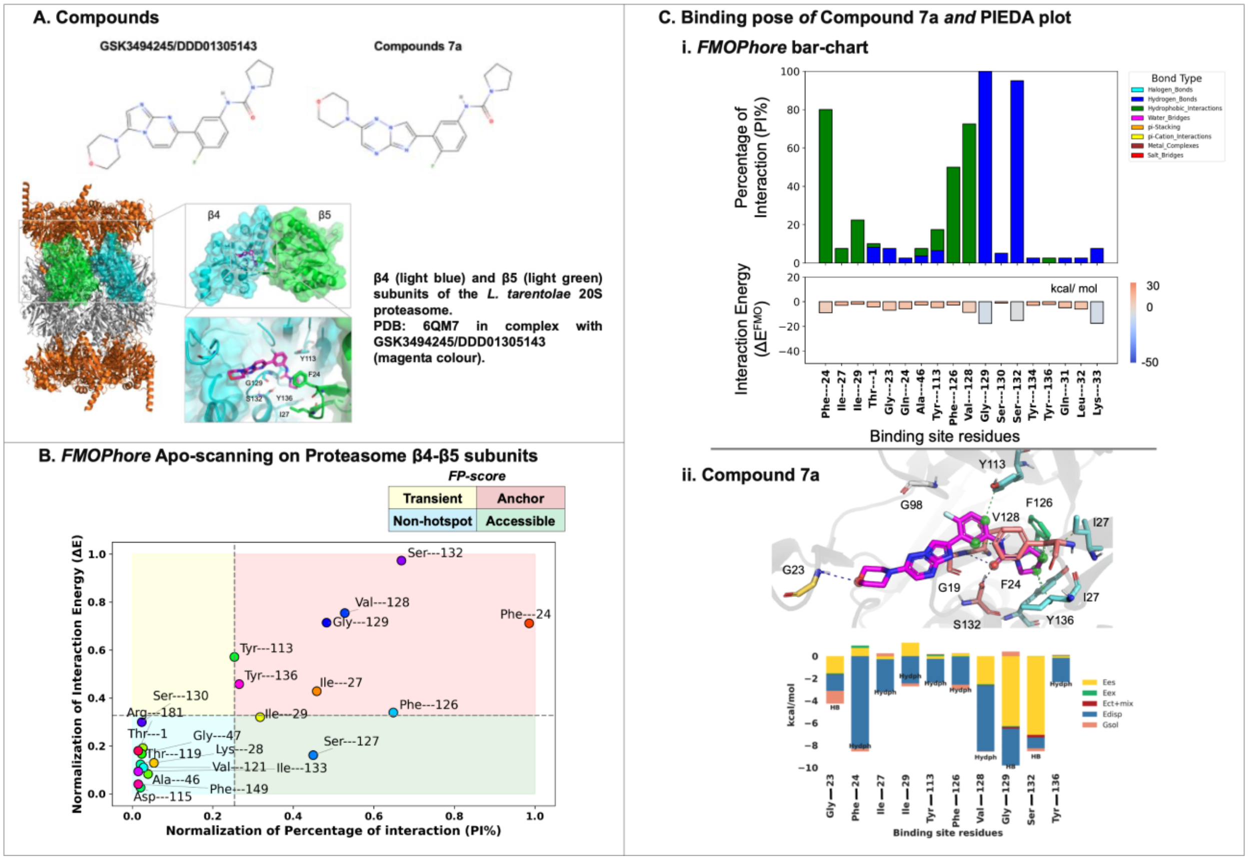

A. Compounds GSK3494245/DDD01305143 and Compound 7*a*. β4 (light green) and β5 (light blue) subunits of the *L. tarentolae* 20S proteasome. PDB: 6QM7 in complex with GSK3494245/DDD01305143 (magenta colour).
B. *FMOPhore* Apo-scanning on Proteasome β4-β5 subunits.
C. Binding pose of Compound 7a and PIEDA plot.
    i. *FMOPhore bar-chart*.
    ii. *Compound 7a*.

Figure 4: FMOPhore analysis of Janus Kinases

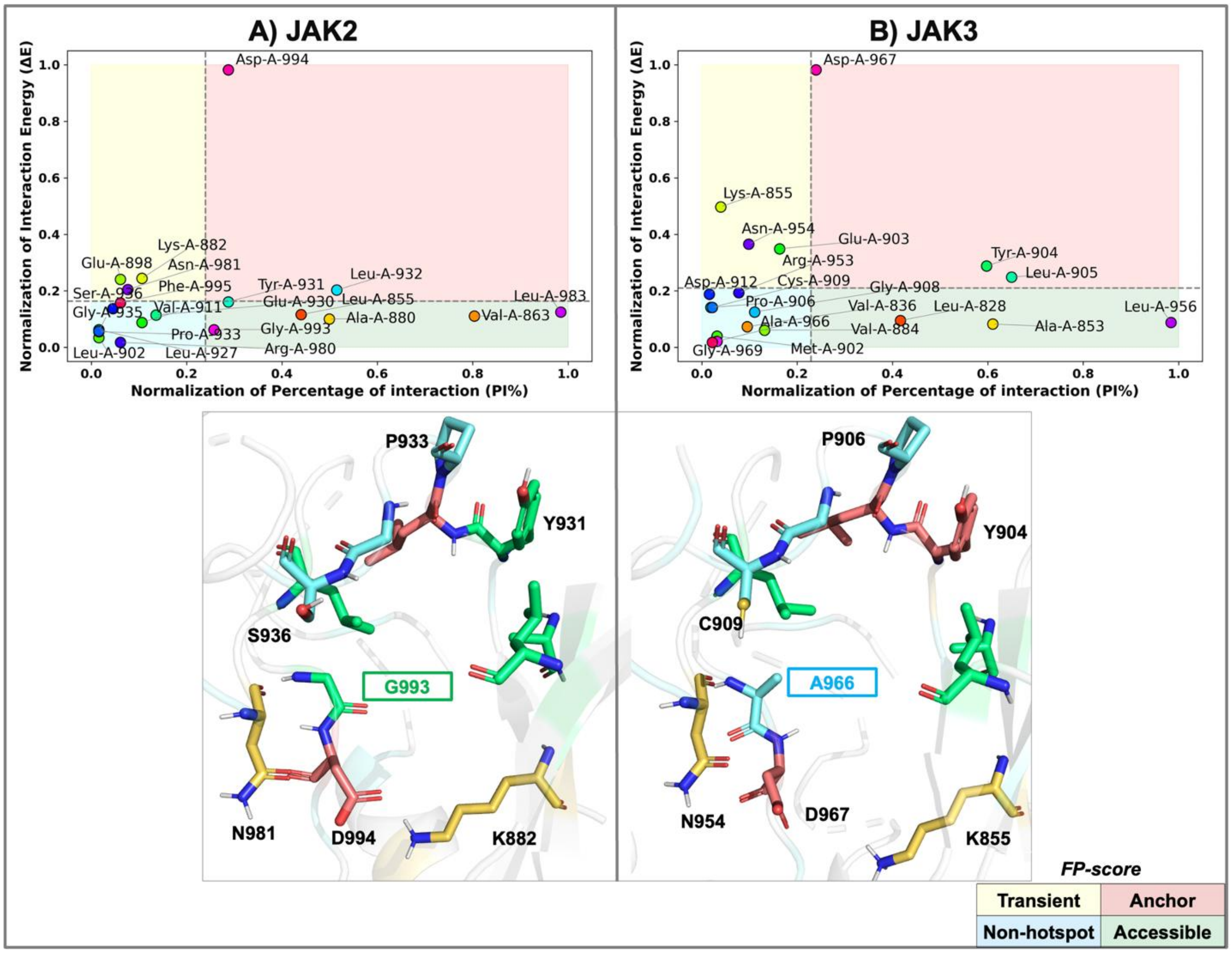

FP-score plot categorising the binding site residues, with normalisation of interaction energies on the y-axis, and normalisation of percentage of interaction on the x-axis. (A) JAK2 and (B) JAK3 for selectivity study, binding site residues coloured as per categorised by FMOPhore scan on apo structure.

Supplementary References

1. Kitaura, K., Ikeo, E., Asada, T., Nakano, T. and Uebayasi, M., 1999. Fragment molecular orbital method: an approximate computational method for large molecules. Chemical Physics Letters, 313(3–4), pp.701–706.
2. Heifetz, A., Chudyk, E.I., Gleave, L., Aldeghi, M., Cherezov, V., Fedorov, D.G. et al., 2015. The fragment molecular orbital method reveals new insight into the chemical nature of GPCR–ligand interactions. Journal of Chemical Information and Modeling, 56(1), pp.159–172.
3. Suenaga, M., 2005. Facio: new computational chemistry environment for PC GAMESS. Journal of Computational Chemistry Japan, 4, pp.25–32.
4. Sastry, G.M., Adzhigirey, M., Day, T., Annabhimoju, R. and Sherman, W., 2013. Protein and ligand preparation: parameters, protocols, and influence on virtual screening enrichments. Journal of Computer-Aided Molecular Design, 27(3), pp.221–234.
5. Peters, M.B., Yang, Y., Wang, B., Füsti-Molnár, L., Weaver, M.N. and Merz, K.M., 2010. Structural survey of zinc-containing proteins and development of the zinc AMBER force field (ZAFF). Journal of Chemical Theory and Computation, 6, pp.2935–2947.
6. Tian, C., Kasavajhala, K., Belfon, K.A.A., Raguette, L., Huang, H., Migues, A.N., Bickel, J., Wang, Y., Pincay, J., Wu, Q. and Simmerling, C., 2020. ff19SB: amino-acid-specific protein backbone parameters trained against quantum mechanics energy surfaces in solution. Journal of Chemical Theory and Computation, 16(1), pp.528–552.
7. Mark, P. and Nilsson, L., 2002. Structure and dynamics of liquid water with different long-range interaction truncation and temperature control methods in molecular dynamics simulations. Journal of Computational Chemistry, 23(13), pp.1211–1219.
8. Berendsen, H.J.C., Postma, J.P.M., Van Gunsteren, W.F., Dinola, A. and Haak, J.R., 1984. Molecular dynamics with coupling to an external bath. The Journal of Chemical Physics, 81(8), pp.3684–3690.

9. Elber, R., Ruymgaart, A.P. and Hess, B., 2011. SHAKE parallelization. The European Physical Journal Special Topics, 200(1), pp.211–223.
10. Kräutler, V., van Gunsteren, W.F. and Hünenberger, P.H., 2001. A fast SHAKE: algorithm to solve distance constraint equations for small molecules in molecular dynamics simulations. Journal of Computational Chemistry, 22, pp.501–508.
11. Essmann, U., Perera, L., Berkowitz, M.L., Darden, T., Lee, H. and Pedersen, L.G., 1995. A smooth particle mesh Ewald method. The Journal of Chemical Physics, 103(19), pp.8577–8593.
12. Pei, J., Tseng, V.S. and Goebel, R., 2013. Advances in knowledge discovery.
13. Schrödinger, 2021. Schrödinger Release 2023-1: LigPrep. Schrödinger, LLC, New York, NY.
14. Friesner, R.A., Murphy, R.B., Repasky, M.P., Frye, L.L., Greenwood, J.R., Halgren, T.A., Sanschagrin, P.C. and Mainz, D.T., 2006. Extra precision Glide: docking and scoring incorporating a model of hydrophobic enclosure for protein-ligand complexes. Journal of Medicinal Chemistry, 49, pp.6177–6196.
15. Aksoy, S. and Haralick, R.M., 2001. Feature normalization and likelihood-based similarity measures for image retrieval.
16. Ibrahim, P.E.G.F., Zuccotto, F., Zachariae, U., Gilbert, I. and Bodkin, M., 2024. Accurate prediction of dynamic protein-ligand binding using P-score ranking. Journal of Computational Chemistry, 45(20), pp.1762–1778.
