# Supplementary material for: FMOPhore for hotspot identification and efficient fragment-to-lead growth strategies
Source: Nat Commun. 2026 Apr 28;17:5813. doi: 10.1038/s41467-026-72384-x (PMC13328607; doi:10.1038/s41467-026-72384-x)
Supplement: Supplementary file 2 — Description of Additional Supplementary Files [file 41467_2026_72384_MOESM2_ESM.pdf]

Description of Additional Supplementary Files

Supplementary Dataset 1: Initial and final snapshots of the molecular dynamics trajectories generated in this study.

Supplementary Movie 1: Dynamic visualization of the Dy-FMOPhore analysis for the 7S3S Mpro protein system.
