# Supplementary material for: FMOPhore for hotspot identification and efficient fragment-to-lead growth strategies
Source: Nat Commun. 2026 Apr 28;17:5813. doi: 10.1038/s41467-026-72384-x (PMC13328607; doi:10.1038/s41467-026-72384-x)
Supplement: Supplementary file 6 — Transparent Peer Review file [file 41467_2026_72384_MOESM6_ESM.pdf]

nature portfolio

Peer Review File

FMOPhore for hotspot identification and efficient fragment-to-lead growth strategies

Corresponding Author: Dr Peter Ibrahim

This file contains all reviewer reports in order by version, followed by all author rebuttals in order by version.

Version 0:

Reviewer comments:

Reviewer #1

(Remarks to the Author)
In this manuscript, Ibrahim et al. present a novel computational approach, FMOPhore, for identifying protein-ligand binding hotspots and guiding fragment-to-lead optimization strategies. The method combines Fragment Molecular Orbital (FMO) calculations with pharmacophore modeling to assess interaction energy and residue accessibility, classifying residues into Anchor, Transient, Accessible, and Non-hotspot categories. The authors validate their method across 32 protein-ligand systems and introduce Dy-FMOPhore, which incorporates protein flexibility for improved hotspot detection. This approach represents a promising advancement in fragment-based drug discovery by addressing challenges in identifying key residues for ligand optimization. The ability to predict hotspots in both holo-complexes and apo-structures using probe libraries further enhances its applicability. However, while the manuscript offers valuable insights, several aspects need to be addressed to improve clarity, reproducibility, and applicability.

Overall Recommendation: Accept with Major Revisions - While the paper presents an innovative and useful tool for fragment-based drug design, certain methodological improvements and expanded validations are necessary to enhance its rigor and applicability.

Major Points:
1. Ligand Fragmentation Granularity
The current method's focus on whole-ligand interactions overlooks residue-level insights tied to specific ligand atoms. The current model deals with ligands as single fragments, which limits its ability to evaluate residue interactions with specific ligand atoms. For lead optimization, it is critical to assess interactions at the ligand's atomic level. Introducing atom-level ligand fragmentation could provide a more detailed view of interaction energy contributions, addressing gaps in lead growth strategies and enabling better growth strategies for lead compounds. The authors should consider adding this functionality and testing its effectiveness.
2. Methodological Details on FMO Calculations
The methodology lacks sufficient detail regarding the FMO calculation process and the integration of pharmacophoric features. Expanding this section would improve reproducibility by providing clear descriptions of the computational framework, input parameters, and any preprocessing steps applied.
Pharmacophores are typically defined at the functional group or atom level, whereas the FMO interaction energies are only calculated at the fragmented single residue level. This discrepancy raises questions about how single residue-level interaction energies are matched to smaller pharmacophore units defined at the functional group or atomic scale. The authors should provide a detailed explanation of the process used to map residue-level energies to these smaller-scale pharmacophoric features to ensure accuracy and applicability for lead optimization strategies.
3. Validation Dataset Scope
The validation dataset primarily focuses on mostly kinase or enzyme protein-ligand systems. Expanding the dataset to include more membrane proteins, intrinsically disordered proteins, or multi-domain systems would demonstrate broader applicability. Additional case studies could further strengthen the method's robustness and relevance to diverse drug targets.
4. Performance Benchmarking
Comparisons to other widely used computational methods, such as molecular dynamics-based approaches and docking algorithms, are insufficient. Including benchmarks against these tools would help clarify FMOPhore's advantages, limitations, and computational performance relative to existing methods.
5. Dynamic Mode Computational Costs

The dynamic approach, Dy-FMOPhore, accounts for protein flexibility, but the manuscript lacks details on computational efficiency and scalability. Given the high computational demand of QM-based methods, the authors should provide a clearer discussion on runtime, hardware requirements, and potential strategies for optimizing performance in larger systems.
6. Additional validation of the computational method
We further recommend demonstrating the correlation between quantitatively identified hotspot interaction energies calculated using FMO and known Kd values in ligand growing strategies. For instance, a statistical regression model could be constructed to analyze the relationship between the magnitude of interaction energies and Kd values, leveraging the known scaffolds and identified Kds used for validation. Such an analysis would provide additional quantitative support and further strengthen the framework's predictive power.
7. Suggested Experimental Validation
If feasible we suggest an experimental verification to further support the findings, which would help the manuscript reach rigour required by Nature Communications. For example, point mutation experiments targeting the hotspots identified by Dy-FMOPhore could be conducted. Measuring the resulting changes in ligand binding affinity would provide direct evidence for the functional importance of these hotspots and reinforce the computational predictions.

Minor Points:
1. Methodological Details
The description of the FMO calculation process is insufficient. To improve reproducibility, the authors should provide more details about input preparation, basis sets, computational parameters, and pharmacophore generation.
2. Probe Library Description
Table S.2, which describes the probe library used for apo-scanning, is missing. Including this data is essential to validate the library's diversity and relevance for modeling various interaction types. The authors should also clarify the rationale for selecting the specific probes and their relevance to fragment-based design.
3. False Positive Analysis
A deeper analysis of false-positive rates, particularly for low-interaction residues classified as non-hotspots, is necessary. Such cases could impact downstream fragment optimization, and addressing this issue would improve confidence in the method.
4. Optimization Protocol for Apo-Structures
The apo-structure scanning process relies on docking probes without a subsequent optimization step for assembled constructs. Including this optimization step could improve hotspot predictions by accounting for strain or conformational adjustments.

(Remarks on code availability)
The code is in GitHub and freely available under an open-source license.

We were not able to test the software because it requires complex other software packages preinstalled such as GAMESS.

Reviewer #2

(Remarks to the Author)
This is very nice work to apply quantum chemistry calculation to facilitate the identification of the protein binding site for drug discovery.

While there are many data for analyzing, the authors have done a very good job at presenting. However, there are many definitions that are unclear.
1. In Equation 1, I disagree with calling delta_E_FMO as binding interaction energy. It would be the binding energy for protein-ligand complexes, but there is no binding for apoprotein.
2. Which version of DFTB was used in this work? Is the dispersion correction included in the calculations?
3. The definition of each term in Equation 2 is not well-defined. What do "number of interactions" and "total number of interactions" mean? For example, is the total number of interactions between protein and all ligands in the series? The definition of these two terms should be clarified.
4. On Page 7, what are high, moderate, and low mean? Saying "low to moderately favorable interaction energy" is too subjective. Are there any ranges?
5. From all FP-score analysis, the authors provided the clarification of each Hotspot class. However, no clarification exists on how the residues are assigned to each category. For example, the residues are assigned to the non-hotspot category when the normalized interaction energy is less than 0.4, and the percentage of interaction is less than 30% in Figure 2B. However, the ranges of the non-hotspot residues in Figure 5B are less than ~0.22 for normalized interaction energy and ~42% PI.
6. What are static and dynamic FMOPhores?
7. What are 2D- and 3D-FMOPhores?
8. How is delta_E_FMO for each residue determined? The FMO interaction energy is for the interaction energy within the system. There is no explanation for how the FMO interaction energy of each residue is calculated.
9. What software is used for molecular visualization?
10. In terms of organization, I think section 3.1 should move to Section 2 as it's not a result or discussion.
11. For FP-score plots,

- are there any reasons for using different colors for different residues for FP-score plots?
- the residue's names are overlayed with each other. It's impossible to read.
12. Figure 7C, the figure of PDB-ID 2OHS is shown, but no discussion.
13. From the writing, sometimes the differences of LE =0.01 are significant, but sometimes they are not. What is your justification of significant changes of delta LEs?
14. In Section 3.4, I noticed that both static and dynamic FMOPhores were performed for some proteins, but some were not. Are there any reasons and/or criteria for choosing when to perform only static FMOPhore or both static and dynamic analysis?
15. In Figure 9, the orange and red colors are very difficult to distinguish. It would be good to separate the static and dynamic modes from each other.
16. Line 510-511, "However, for other systems such as HSP90 and Hepatitis-C, were more challenging yet, both reported better classification for both Hotspots and Non-hotspots." —> I can't find the discussion about the challenges and the results of these protein systems.
17. It would be good to provide the details of the MD simulation.
18. Fragment molecular orbital (FMO) method is quantum mechanics based fragmentation. Are there any specific reasons in calling it as "QM-FMO"?
19. In the discussion, the authors provided some background of previous studies. However, some sentences are unclear if they were based on the literature or from the presented work.

There are so many grammatical errors. Here are some examples,
• In the abstract, "FP-score accurately classify binding site residues" should be "FP-score accurately classifies binding site residues"
• Page 3 line 26, "Thermal Shift Analysis are" should be "Thermal Shift Analysis is".
• Page 4 line 39, "However, identification of hotspots and quantitatively determining" should be "However, identifying hotspots and quantitatively determining".
• Line 41, "complementary ligand has been" should be "complementary ligand have been".
• Line 53, "thirty-two" should be "32".
• Line 74, "For protein-ligand pharmacophore models generation" should be "For protein-ligand pharmacophore model generation"
• Line 105, 373, verb is missing.
• Page 7, there are many subject/verb agreement errors.
• Line 135-137, "Transient residues if targeted with a proper complimentary functional group can play a key role in increase 137 binding affinity while positively influencing the ligand efficiency." —> not sure what the authors are trying to explain.
• Line 189-190, "fifty different holo-complexes of noncovalent protein-ligands" should be "50 different holo-complexes of noncovalent protein-ligand complexes".
• Inconsistant use of the Oxford commas
• Line 460, "the carboxylic", should it be "the carbonyl"?

Missing references for
• GAMESS software
• FMO
• FMO2-DFTB
• Section 3.1

(Remarks on code availability)
The FMOPhore codes are available on GitHub repository. https://github.com/PeterEGFIbrahim/FMOPhore/tree/main

While I looked at the repository, I don't have a chance to install and running it. README file is provided with the details of installation, example commands, and expected outputs. However, there is no manual documentation or the example of the input files.

Reviewer #3

(Remarks to the Author)
I co-reviewed this manuscript with one of the reviewers who provided the listed reports. This is part of the Nature Communications initiative to facilitate training in peer review and to provide appropriate recognition for Early Career Researchers who co-review manuscripts.

(Remarks on code availability)

Reviewer #4

(Remarks to the Author)
I have carefully read the manuscript entitled "FMOPhore for hotspot identification and efficient fragment-to-lead growth strategies."

This is a methodological work focused on the development of a new algorithm, similar to many others, aimed at qualifying pharmacophores derived from ligand/protein complexes. From a scientific perspective, this work is technically sound but

lacks originality, relying on well-established techniques in the field of ligand/protein interactions to extract additional information.

Scientifically, the study does not address any significant unresolved challenges. In fact, I believe its level of originality is considerably lower than articles published in ACS journals such as _J. Chem. Inf. Model._ or _JCTC_. I do not believe this manuscript is suitable for publication in _Nature Communications_.

I regret not being able to provide a more positive assessment of this manuscript.

(Remarks on code availability)

Version 1:

Reviewer comments:

Reviewer #1

(Remarks to the Author)
I would like to thank the authors for their thorough and constructive responses to my previous comments. Overall, the revisions address several of the points I raised, and the manuscript is now clearer in

In particular, the additional methodological details regarding the FMO calculations and pharmacophore integration, as well as the expanded validation dataset covering a more diverse set of targets, have improved the transparency of the study. The clarification on computational costs and the inclusion of supplementary information (workflow, input files, and probe library description) are also useful additions. The inclusion of an experimental validation of a point mutation case study provides some further support for the applicability of the proposed framework.

Nevertheless, I remain concerned that the current evaluation relies primarily on retrospective benchmarking. While these results are informative, I believe that for the approach to demonstrate genuine novelty and practical impact, prospective experimental validation will be essential. Without such evidence, the robustness and translational relevance of the framework remain uncertain.

(Remarks on code availability)

Reviewer #2

(Remarks to the Author)
Thanks for your responses. Many of my questions have been addressed.
1. It's clear to me now how the FP-score quadrant is generated. I believe the explanation on using the mean values of interaction energy and PI% as a threshold should be included in the main manuscript, since this is the key to classifying the hotspot.

2. In the manuscript, it is mentioned that PCM was included in FMO/DFTB calculations. However, the supporting info shows that no solvation model is used. This conflict needs to be resolved.

3. The work on FMO/DFTB3 is not cited. This reference should be included. https://doi.org/10.1016/j.cplett.2015.07.022

Overall, this work can contribute to applying quantum mechanics in drug discovery, helping identify binding site hotspots.

(Remarks on code availability)

Reviewer #3

(Remarks to the Author)
I co-reviewed this manuscript with one of the reviewers who provided the listed reports. This is part of the Nature Communications initiative to facilitate training in peer review and to provide appropriate recognition for Early Career Researchers who co-review manuscripts.

(Remarks on code availability)
The code is in GitHub and freely available under an open-source license.
We were not able to test the software because it requires complex other software packages preinstalled such as GAMESS.

Reviewer #4

(Remarks to the Author)
I have no further comments to add.

(Remarks on code availability)
Why two conda deactivate commands in the shell-script ? (lines 16 and 17)

Version 3:

Reviewer comments:

Reviewer #1

(Remarks to the Author)
The reviewers have done a meaningful revision, and the paper can be recommended for publication.

(Remarks on code availability)

Reviewer #2

(Remarks to the Author)
This work presents FMOPhore, a computational tool for identifying protein binding-site hotspots to guide fragment-to-lead optimization. Calculating paired interaction energies between residues and ligand using the Fragment Molecular Orbital (FMO) method, FMOPhore uses the FP-score, which combines paired interaction energies and residue accessibility to classify binding-site residues into four categories. Tested on 46 protein targets and 840 complexes, FMOPhore reliably finds residues key to ligand potency, efficiency, and selectivity. FMOPhore uniquely integrates QM-derived interaction energies, pharmacophore models, and protein dynamics.

(Remarks on code availability)

Reviewer #3

(Remarks to the Author)
I co-reviewed this manuscript with one of the reviewers who provided the listed reports. This is part of the Nature Communications initiative to facilitate training in peer review and to provide appropriate recognition for Early Career Researchers who co-review manuscripts.

(Remarks on code availability)

Open Access This Peer Review File is licensed under a Creative Commons Attribution 4.0 International License, which

permits use, sharing, adaptation, distribution and reproduction in any medium or format, as long as you give appropriate credit to the original author(s) and the source, provide a link to the Creative Commons license, and indicate if changes were made.
In cases where reviewers are anonymous, credit should be given to 'Anonymous Referee' and the source.
The images or other third party material in this Peer Review File are included in the article's Creative Commons license, unless indicated otherwise in a credit line to the material. If material is not included in the article's Creative Commons license and your intended use is not permitted by statutory regulation or exceeds the permitted use, you will need to obtain permission directly from the copyright holder.
To view a copy of this license, visit https://creativecommons.org/licenses/by/4.0/

Dear reviewers,

Firstly, we wanted to take a moment to express our thanks for taking the time to review the paper. Your feedback and constructive comments are useful, and we believe have enhanced the quality of our report. We genuinely appreciate the effort you put into providing feedback.

Accordingly, we would like to reply to the mentioned feedback as per reviewer:

Reviewer: 1

---

1. Ligand Fragmentation Granularity

The current method's focus on whole-ligand interactions overlooks residue-level insights tied to specific ligand atoms. The current model deals with ligands as single fragments, which limits its ability to evaluate residue interactions with specific ligand atoms. For lead optimization, it is critical to assess interactions at the ligand's atomic level. Introducing atom-level ligand fragmentation could provide a more detailed view of interaction energy contributions, addressing gaps in lead growth strategies and enabling better growth strategies for lead compounds. The authors should consider adding this functionality and testing its effectiveness.

We agree with the reviewer's point of view regarding the need for a more granular approach to ligand fragmentation.

The *FMOPhore* algorithm presented in this paper primarily focuses on an earlier phase of the drug discovery pipeline by identifying binding site hotspots and defining fragment growth strategies. The core objective is to categorize key binding site residues using the *FP-score* equation and quadrant plot, rather than analysing ligand fragmentation at the atomic level.

Fragment Molecular Orbital (FMO) calculations can indeed be applied to lead-optimization phase, however, our current study emphasizes the binding site residues perspective, rather than ligand-centric interactions. This is done by using small probes

library ranging from minimum of 2 and a maximum of 8 heavy atom count, while scanning the binding site in the apo-structure scan.

We acknowledge that integrating atom-level ligand fragmentation could enhance the understanding of residue-specific interaction energies and refine lead optimization strategies. To address this, we are actively developing an extended version of *FMOPhore* that incorporates ligand fragmentation at the atomic level for lead optimization applications. This will be introduced in a further study (Part 2), using *FMOPhore* hotspots in generative design which is currently in preparation.

---

2. Methodological Details on FMO Calculations

The methodology lacks sufficient detail regarding the FMO calculation process and the integration of pharmacophoric features. Expanding this section would improve reproducibility by providing clear descriptions of the computational framework, input parameters, and any preprocessing steps applied.

Pharmacophores are typically defined at the functional group or atom level, whereas the FMO interaction energies are only calculated at the fragmented single residue level. This discrepancy raises questions about how single residue-level interaction energies are matched to smaller pharmacophore units defined at the functional group or atomic scale. The authors should provide a detailed explanation of the process used to map residue-level energies to these smaller-scale pharmacophoric features to ensure accuracy and applicability for lead optimization strategies.

We understand the valid point regarding the methodological integration of FMO interaction energies with pharmacophore features in the *FMOPhore* framework. We acknowledge that pharmacophore features are typically defined at the level of functional groups or individual atoms, whereas FMO calculations yield interaction energies at the granularity of fragmented protein residues. This conceptual disparity is a central challenge that *FMOPhore* is specifically designed to address.

In our methodology, the *FP-score* equation was introduced to bridge this scale gap. It integrates two orthogonal yet complementary descriptors:

1- **Pharmacophores frequency represented in Percentage of Interaction (PI%)**: This quantifies the prevalence of known pharmacophore (ligand) interactions across a residue (e.g., side chains or backbone), based on systematic enumeration of interaction fingerprints. It reflects the accessibility and functional relevance of the residue.

2- **FMO-Based Interaction Energy (ΔE)**: This measures the quantum-mechanical interaction energy between the ligand (or probe; in case of apo-structure scan) and an individual binding site residue. Although computed at the residue level, this energy is highly sensitive to the presence and type of pharmacophore features on the ligand interacting/ clashing with the binding site residue. For instance:

   - **A hydrogen bond donor** interacting with an acceptor residue will result in a pronounced electrostatic attraction (defined in the $\mathbf{\Delta E_{ij}^{es}}$ - term of FMO equation).
   - An interaction between **two donors or two acceptors** yields repulsive energy components ($\mathbf{\Delta E_{ij}^{ex}}$).
   - **Lipophilic interactions** produce significant dispersion (van der Waals) contributions, reflective of hydrophobic packing ($\mathbf{\Delta E_{ij}^{DI}}$).

By evaluating both **PI%** and **ΔE** independently and combining them, the ***FP-score*** captures both the geometrical and chemical plausibility (via PI%) and the energetic feasibility (via FMO) of a pharmacophore-driven interaction.

Additionally, we have now expanded the Supplementary Files 1.1 mentioned in the methodology section of the manuscript to include:

- Full details of the FMO input generation (fragmentation protocol, basis sets, and level of theory).
- Preprocessing steps such as protonation state standardization, residue capping, and ligand minimization.
- Explicit explanation of how pharmacophore fingerprints are generated and statistically mapped across the interface.

---

3. Validation Dataset Scope

The validation dataset primarily focuses on mostly kinase or enzyme protein-ligand systems. Expanding the dataset to include more membrane proteins, intrinsically disordered proteins, or multi-domain systems would demonstrate broader applicability. Additional case studies could further strengthen the method's robustness and relevance to diverse drug targets.

Thank you for highlighting this important point regarding the diversity and generalizability of the validation dataset.

We acknowledge that the current benchmark set focuses predominantly on well-structured enzyme and kinase targets. This selection was intentional in the early stages of development to ensure rigorous and interpretable validation under well-characterized structural and energetic contexts. These systems provide reliable co-crystal structures and well-annotated interaction data, which are essential for calibrating and evaluating novel scoring functions such as the *FP-score* used in *FMOPhore*.

However, in response to the need for broader applicability, we have significantly expanded the dataset. We have now incorporated 14 additional and diverse systems, including GPCRs (e.g., CB1 cannabinoid receptor, Beta-1 and Beta-2 adrenergic receptors, Histamine H1 receptor, Adenosine A1 and A2 receptors), protein–protein interaction systems (e.g., MDM2), oncogenic signaling components (e.g., KRAS-PDEδ complex), metabolic enzymes (e.g., human carbonic anhydrase II, class A beta-lactamase), and regulatory targets (e.g., BCL-xL BH3 domain, mGlu5, and Tyrosine Phosphatase 1B). This updated selection ensures a more balanced representation of different structural classes, including:

- A prospective validation on the Proteosome B5 subunit, validated against saturation with Mutagenesis study done by a collaborator *Altmann et al*. (https://www.biorxiv.org/content/10.1101/2025.07.25.666894v1).
- Membrane proteins (GPCRs)
- Metabolic and signalling enzymes

- Multi-domain and disordered targets
- Include targets with known disordered binding regions (e.g., MDM2–p53)

This broader scope allows us to capture the challenges associated with:

- Dynamic and ambiguous binding pockets (e.g., in PPI and disordered targets).
- Membrane-embedded environments (e.g., for GPCRs).
- Allosteric regulation and interdomain coupling (e.g., in multi-domain proteins).

---

4. Performance Benchmarking

Comparisons to other widely used computational methods, such as molecular dynamics-based approaches and docking algorithms, are insufficient. Including benchmarks against these tools would help clarify FMOPhore's advantages, limitations, and computational performance relative to existing methods.

We appreciate the reviewer's suggestion regarding performance benchmarking. However, we would like to clarify that *FMOPhore* is not intended to replace or directly compete with molecular dynamics (MD) simulations or docking algorithms. Instead, it serves a complementary and distinct role: *FMOPhore* is designed to identify and classify hotspot residues by integrating Fragment Molecular Orbital (FMO) energy decomposition with pharmacophore-based models, thereby guiding fragment-growing strategies in a structure-informed and chemically meaningful manner.

While MD and docking are primarily used to simulate ligand–receptor dynamics or predict binding poses, *FMOPhore* focuses on residue-level interaction profiling. It leverages quantum-informed interaction energies and pharmacophore patterns to highlight key interaction hotspots.

Furthermore, *FMOPhore* is integrated with molecular dynamics (MD) to create *Dynamic-FMOPhore*, a hybrid approach that captures hotspot evolution across simulation timeframes, providing a dynamic view of protein–ligand interactions. Similarly, when combined with docking, *FMOPhore* enhances static binding pose evaluations by offering

residue-specific insights into interaction strengths and pharmacophoric relevance.

---

5. Dynamic Mode Computational Costs

The dynamic approach, Dy-FMOPhore, accounts for protein flexibility, but the manuscript lacks details on computational efficiency and scalability. Given the high computational demand of QM-based methods, the authors should provide a clearer discussion on runtime, hardware requirements, and potential strategies for optimizing performance in larger systems.

We appreciate the reviewer's comment regarding the computational efficiency and scalability of the *Dy-FMOPhore* approach. All molecular dynamics (MD) simulation details, including system setup, hardware used, simulation duration, and trajectory sampling parameters, are clearly described in Supplementary Files and information 1.3. These details illustrate the practical feasibility of applying *Dy-FMOPhore* to medium and large-scale systems.

---

6. Additional validation of the computational method

We further recommend demonstrating the correlation between quantitatively identified hotspot interaction energies calculated using FMO and known Kd values in ligand growing strategies. For instance, a statistical regression model could be constructed to analyze the relationship between the magnitude of interaction energies and Kd values, leveraging the known scaffolds and identified Kds used for validation. Such an analysis would provide additional quantitative support and further strengthen the framework's predictive power.

We appreciate the reviewer's thoughtful suggestion. Indeed, establishing a quantitative correlation between FMO-derived interaction energies and experimentally determined Kd values would provide valuable validation of the method's predictive strength. This is a direction we are actively pursuing which aligns closely with our ongoing research.

However, we would like to clarify that the current manuscript (Part 1) is focused primarily on hotspot prediction and assessing the recall percentage between holo and apo scans of the binding site using the *static* and *Dynamic-FMOPhore* framework. This

foundational step is crucial for mapping interaction-relevant regions prior to ligand elaboration.

The proposed analysis involving fragment growing strategies and statistical modeling in correlation with the Kd values will be the core of our forthcoming follow-up study (Part 2), which will extend the *FMOPhore* methodology into a fully integrated fragment-to-lead optimisation pipeline.

---

7. Suggested Experimental Validation

If feasible we suggest an experimental verification to further support the findings, which would help the manuscript reach rigour required by Nature Communications. For example, point mutation experiments targeting the hotspots identified by Dy-FMOPhore could be conducted. Measuring the resulting changes in ligand binding affinity would provide direct evidence for the functional importance of these hotspots and reinforce the computational predictions.

We fully agree that such validation would further strengthen the rigor and impact of our findings. To this end, we would like to highlight that we have already undertaken a prospective validation of the *Dy-FMOPhore* algorithm using the proteasome β5 subunit (PSMB5) through point mutation study. This system was selected due to its biomedical relevance in drug resistance and its suitability for mutagenesis-based probing.

Specifically, we benchmarked *Dy-FMOPhore's* predictions of essential interaction hotspots against results from a saturation mutagenesis study conducted by our experimental collaborators (Altman et al.), recently made publicly available as a preprint: *Altmann et al*., 2025. bioRxiv.

https://www.biorxiv.org/content/10.1101/2025.07.25.666894v1

This study systematically introduced point mutations across the β5 subunit and assessed their effects on ligand binding affinity. *Dy-FMOPhore* not only accurately identified most experimentally validated functional hotspots, but also effectively distinguished non-hotspot residues. This dual capability underscores the method's potential to prioritise biologically relevant mutations while eliminating superfluous

mutational candidates early in the design cycle. The strong concordance between computational predictions and experimental observations provides compelling support for the robustness and predictive power of the *Dy-FMOPhore* framework.

We have included this case study in the revised manuscript (Section 3.5, Figure 8.) and will continue to expand such validations in future work to include other protein families and mechanisms of resistance.

---

8. Methodological Details

The description of the FMO calculation process is insufficient. To improve reproducibility, the authors should provide more details about input preparation, basis sets, computational parameters, and pharmacophore generation.

We appreciate the reviewer's suggestion regarding the need for further methodological clarity. To address this, we have expanded the Methods and Supplementary Information to clearly describe all computational steps.

Specifically, protein–ligand complexes are first prepared using Maestro's Protein Preparation Wizard (Schrödinger suite). The output .pdb file from this preparation is used as the direct input to the *FMOPhore* code.

Within the FMOPhore pipeline: The complex is automatically fragmented using a distance-based cutoff (default 5 Å from ligand atoms, user-adjustable).

Fragmentation is handled according to a well-established fragmentation strategy by Facio. The FMO calculations are performed at the DFTB (Density Functional Tight Binding) level using GAMESS, with all input settings generated automatically by the code.

Protein–ligand pharmacophoric features are extracted using PLIP (Protein-Ligand Interaction Profiler), which identifies hydrogen bonds, hydrophobic contacts, salt bridges, and π-interactions.

The *FP-score* is then computed by analysing the interaction energy per residue and quantifying the percentage of interaction (PI%) across ligands. The scoring matrix is created and plotted to categorize binding site residues into *Anchors, Transients, Accessibles*. or *non-hotspots*. We have included a methodological workflow and example input/output files in the Supplementary Files (Sections 1.1).

Additionally, all code, scripts, and templates used in this study are publicly available on our GitHub repository: https://github.com/PeterEGFIbrahim/FMOPhore

---

9. Probe Library Description

Table S.2, which describes the probe library used for apo-scanning, is missing. Including this data is essential to validate the library's diversity and relevance for modeling various interaction types. The authors should also clarify the rationale for selecting the specific probes and their relevance to fragment-based design.

We appreciate the reviewer's thoughtful suggestion. In response, we have now included the full list of probes in Table 1 of the revised manuscript, replacing the previously referenced Table S.2. Additionally, we have clarified the rationale behind the selection of these probes, emphasizing their ability to represent key interaction types commonly targeted in fragment-based drug design (e.g., hydrogen bonding, hydrophobic contacts, aromatic stacking). This update ensures the diversity and relevance of the apo-scanning probe library.

---

10. False Positive Analysis

A deeper analysis of false-positive rates, particularly for low-interaction residues classified as non-hotspots, is necessary. Such cases could impact downstream fragment optimization and addressing this issue would improve confidence in the method.

Understanding false positives is certainly significant but this publication introduces a quantitative approach to classify binding hotspots. In our exemplars we have focussed

on exemplifying how the *FMOPhore* approach can be used to rationalise the key drivers of binding. As time progresses and over many more examples than what we have provided here in this publication we expect to establish a deeper understanding of the success of the *FMOPhore* approach.

---

11. Optimization Protocol for Apo-Structures

The apo-structure scanning process relies on docking probes without a subsequent optimization step for assembled constructs. Including this optimization step could improve hotspot predictions by accounting for strain or conformational adjustments.

In the apo-structure scanning process, the docking protocol is carried out without the use of constraints, allowing the probes to explore the binding site freely. For each probe, up to 50 poses are generated, ensuring a broad conformational sampling of potential binding modes within the apo pocket.

Regarding protein conformational flexibility, this is addressed within the *Dy-FMOPhore* module, which performs a dynamic scan of multiple protein conformations extracted from the molecular dynamics (MD) trajectory.

This enables the algorithm to capture transient and flexible hotspots that are not always observable in a single static structure. Therefore, while no explicit optimization of the assembled constructs is performed post-docking, both ligand and protein flexibility are systematically incorporated into the *Dy-FMOPhore* pipeline through comprehensive sampling.

Reviewers: 2 and 3

---

1. **In Equation 1, I disagree with calling delta_E_FMO as binding interaction energy. It would be the binding energy for protein-ligand complexes, but there is no binding for apoprotein.**

We appreciate the reviewer’s thoughtful observation and agree that the term “binding interaction energy” may be misleading. To address this, we have revised the terminology

in the manuscript to refer to ΔE^{FMO} as "interaction energy" rather than "binding interaction energy" throughout the text.

- For holo-complexes, ΔE^{FMO} represents the protein-ligand interaction energy between the ligand and individual protein residues, which can be interpreted as residue-level contributions to binding.
- However, in case apo-structure scenario, the probes library used for scanning the binding site after docking. Therefore, it is still an interaction energy between the probes (ligands) and the binding site residues.

---

2. **Which version of DFTB was used in this work? Is the dispersion correction included in the calculations?**

We used DFTB3 (third-order Density Functional Tight Binding) with the 3ob-3-1 parameter set, as implemented in GAMESS. This version enhances the description of charge transfer interactions and includes corrections for dispersion and solvation effects (PCM water model).

Yes, dispersion correction is included in the calculations using the UFF model (DISP=UFF). This is explicitly specified in the GAMESS input files via the following keywords:

This can be found in the input files for GAMESS calculations with the following flags:

```
$DFTB
  SCC=.TRUE. DFTB3=.TRUE. DAMPXH=.TRUE. DAMPEX=4.00 DISP=UFF
 $END
```

These settings ensure a balanced treatment of non-covalent interactions, which are essential for accurately identifying interaction hotspots in the FMOPhore workflow.

---

3. **The definition of each term in Equation 2 is not well-defined. What do "number of interactions" and "total number of interactions" mean? For example, is the total number of interactions between protein and all ligands in the series? The definition of these two terms should be clarified.**

Thank you for your valuable comment the following section has been rephrased and written in the revised version for more clarification on the terms and the Percentage of interaction equation (PI).

FMOPhore quantify protein-ligand binding interactions using PLIP which identifies various bond types including hydrophobic interactions, hydrogen bonds (both donors and acceptors), salt bridges, π–cation interactions, π–π stacking, water bridges, halogen bonds, and metal complexes. These interaction types are used to generate protein-ligand (PL) fingerprints that describe how each residue in the binding site interacts with ligands. The proportion of ligands that form interactions with a given residue is then calculated and expressed as the Percentage of Interaction (PI%), providing a quantitative measure of residue accessibility. This is formalized in Equation 2:

$$\boldsymbol{PI}\ (\%) = \frac{\boldsymbol{Frequency\ of\ Ligand\ interactions\ per\ residue}}{\boldsymbol{Total\ number\ of\ interactions}} * \mathbf{100}$$

Equation 2: PI (%); Percentage of interaction with a binding site residue. Frequency of Ligands interactions per residue is the number of ligands that interact with the residue. Total number of interaction opportunities is the total number of ligands scanned for that protein.

The percentage of interaction with a binding site residue is calculated as the proportion of protein-ligand interactions observed for a specific residue within the binding site, relative to the total number of sampling points (or ligands) analysed. This metric quantifies how frequently a particular residue interacts with ligands during the sampling process, providing insight into the residue's significance in ligand binding. A higher percentage indicates that the residue plays a more prominent role in ligand interactions across the dataset.

FMOPhore is based on a novel scoring function, that I developed named *FP-score* that classifies binding site residues in two classes: 1) Hotspot residues (further detailed into three categories; Anchor, Transient, and Accessible) and 2) non-hotspot residues.

4. **On Page 7, what are high, moderate, and low mean? Saying “low to moderately favorable interaction energy” is too subjective. Are there any ranges?**

Thank you for the valuable feedback. We agree that terms like "high," "moderate," and "low" are subjective without specific numerical ranges. To provide more clarity, we define these interaction energy ranges based on Fragment Molecular Orbital (FMO) calculations, which typically express interaction energy (ΔE^{FMO}) in kcal/mol.

**Category**	**Interaction Energy (ΔE^{FMO}) Range (kcal/mol)**	**Description**
**Highly Favorable (High)**	≤ -5.0 kcal/mol	Strong and stable interactions, typically forming hydrogen bonds or strong electrostatic interactions.
**Moderately Favorable (Moderate)**	-5.0 to -2.5 kcal/mol	Weaker interactions, such as hydrophobic contacts or weak electrostatic attractions.
**Low Favorability (Low)**	-2.5 to 0 kcal/mol	Minimal interaction energy, suggesting weak binding, can be broken easily with a small distortion in the binding conformation.
**Unfavorable**	> 0 kcal/mol	Destabilizing interactions, indicating repulsion or steric clashes.

- Anchor-category residues exhibit a high percentage of interaction and highly favorable interaction energy (≤ -5.0 kcal/mol).
- Transient-category residues have a low percentage of interaction but still contribute highly favorable interaction energy (≤ -5.0 kcal/mol).
- Accessible-category residues show a high percentage of interaction, but their interaction energy is in the low to moderately favorable range (-5.0 to -2.5 kcal/mol).
- Non-hotspot class residues exhibit low interaction percentages and low to unfavorable interaction energy (-2.5 to >0 kcal/mol).

---

5. **From all FP-score analysis, the authors provided the clarification of each Hotspot class. However, no clarification exists on how the residues are assigned to each category. For example, the residues are assigned to the non-hotspot category when the normalized interaction energy is less than**

0.4, and the percentage of interaction is less than 30% in Figure 2B. However, the ranges of the non-hotspot residues in Figure 5B are less than ~0.22 for normalized interaction energy and ~42% PI.

Thank you for your valuable comment. The classification of binding site residues into categories is based on relative comparisons between residues using the FP-score equation, rather than predefined threshold values. This process is performed automatically, without human intervention, by leveraging the system-specific mean values of two descriptors: the percentage of interaction (PI%) and the interaction energy (ΔE).

In the *FP-score* plots (Figure 1), the vertical line intersecting the x-axis represents the mean percentage of interaction (PI% mean), while the horizontal line intersecting the y-axis represents the mean interaction energy (ΔE mean).

Each case determines its own mean values of interaction energy and accessibility, based on the relative distribution of the binding site residues within that specific dataset. This explains the variation in threshold values observed in all figures, as they are dataset-dependent rather than predefined fixed ranges.

$$\boldsymbol{FP_{score}} = \left(\frac{\left(\mathbf{1} + \boldsymbol{max}\left(\boldsymbol{E^{FMO}}\right)\right) - \left(\boldsymbol{E_a^{FMO}}\right)}{\left(\mathbf{1} + \boldsymbol{max}\left(\boldsymbol{E^{FMO}}\right)\right) - \left(\boldsymbol{min}\left(\boldsymbol{E^{FMO}}\right) - \mathbf{1}\right)}\right) * \left(\frac{(\boldsymbol{PI}) - (\boldsymbol{min}(\boldsymbol{PI}) - \mathbf{1})}{\left(\mathbf{1} + \boldsymbol{max}(\boldsymbol{PI})\right) - (\boldsymbol{min}(\boldsymbol{PI}) - \mathbf{1})}\right)$$

The *FP-score* equation is inspired from the rescaling normalization equation where the range of all descriptors should be normalized so that each descriptor contributes proportionately to a final score. The normalization formula is a statistics formula that can transform a data set so that all its variations fall between zero and one. This can be helpful when comparing two or more data sets with different scales. Using the normalization formula allows for the representation of data points as values ranging from zero to one, where the smallest data point is assigned a normalized value of zero and the largest data point is assigned a normalized value of one. The remaining data points are assigned decimal values falling between these two extremes, relative to their positions within the overall range of the dataset.

In the case of the *FP-score* equation, based on the relative concept, that it is only be used for comparing the binding site residues to each other. On the interaction energy

calculation part (left-hand side of the equation after the = sign). Since all binding site residues should be considered with normalization values between 0 and 1 (but not 0 nor 1), where 0 means that there is no interaction energy, and 1 is referred as the ultimate interaction energy, yet FMO only consider non-covalent interactions in the *FP-score*. Therefore, in the denominator all the energy values fall in the range; “1+ energy values - 1”, to allow all values to fall within the range of 0 and 1, yet not equal to 0 nor 1, and since the range increased in the denominator so the comparison between the binding site residues should be to the maximum value which is the 1+*max(*ΔE*).* On the right-hand side of the equation (after the = sign), each binding site residue is evaluated according to its accessibility to form interaction fingerprint with docked or crystalized ligands. in comparison to the total number of sampling (docked ligands) points.

---

6. What are static and dynamic FMOPhore?

FMOPhore is an algorithm designed to identify key binding site residues (hotspots) based on two scenarios: (1) holo-complex analysis of multiple ligands bound to the same binding site or (2) apo-scanning the binding site using a small library of probes with diverse pharmacophoric features.

Since proteins are inherently dynamic, a single binding site conformation may not fully capture the flexibility of the binding pocket, including its opening and closing motions, which influence the volume of the pocket and accessibility of its binding site residues. A ***Static FMOPhore*** module refers to scanning a single conformation of the binding site (i.e., a single snapshot obtained either from a molecular dynamics (MD) trajectory or directly from a crystallized structure). However, this approach can be less accurate, as it does not account for the structural flexibility of proteins, particularly the opening or closing of cryptic pockets, which can significantly alter the accessibility and chemical characteristics of binding site residues.

To address this limitation, we introduced the ***Dynamic FMOPhore (Dy-FMOPhore)*** module, which scans multiple binding site conformations derived from an MD trajectory and computes the average *FP-score* across all snapshots. This approach improves

accuracy in replicating the structure-activity relationship (SAR) of different fragment-growing strategies, as discussed in Section 3.3.

---

7. **What are 2D- and 3D-FMOPhores?**

FMOPhore outputs can be visualized in two complementary formats: 2D-FMOPhore heatmaps and 3D-FMOPhore models.

The ***2D-FMOPhore*** **heatmap** (e.g., Figure 2A) provides a visual summary of the interaction landscape, allowing scientists to assess the relative strength and frequency of interactions between individual ligands and specific binding site residues. This representation highlights key hotspots based on energetic favourability and interaction prevalence, enabling data-driven prioritization of fragment-growing strategies.

The ***3D-FMOPhore*** **model** (e.g., Figure 2C) offers a three-dimensional pharmacophore-like representation derived from quantum-informed residue interactions. It captures the spatial arrangement of key interaction features, effectively serving as a 3D pharmacophoric fingerprint of the binding site. This model can be directly used to screen or design molecules that optimally target the identified hotspot regions, facilitating rational lead optimization.

---

8. **How is delta_E_FMO for each residue determined? The FMO interaction energy is for the interaction energy within the system. There is no explanation for how the FMO interaction energy of each residue is calculated.**

We appreciate the reviewer's observation. The ΔE^{FMO} for each residue in our framework is computed as traditional quantum mechanical pairwise interaction energy, represented in the strength of the pharmacophore-derived protein–ligand interaction fingerprint.

Specifically, we quantify the contribution of each residue based on: The frequency, strength, and type of pharmacophore interactions (e.g., hydrogen bonds, π-stacking, hydrophobic contacts).

Derived from dynamic sampling and mapped to a per-residue level using *FMOPhore's* normalized scoring function (*FP-score*). This method allows us to capture not just static proximity but functionally relevant, energetically weighted interactions.

---

9. What software is used for molecular visualization?

All molecular visualizations presented in the manuscript were generated using PyMOL (The PyMOL Molecular Graphics System, Version 2.5, Schrödinger, LLC). PyMOL was used to render protein-ligand complexes, highlight interaction hotspots, and prepare all structural figures.

---

10. In terms of organization, I think section 3.1 should move to Section 2 as it's not a result or discussion.

Thank you for the suggestion. We have revised the manuscript accordingly and moved Section 3.1 to Section 2 to better align with the organizational flow, as recommended.

---

11. For FP-score plots,
- are there any reasons for using different colors for different residues for FP-score plots?
- the residue's names are overlayed with each other. It's impossible to read.

The *FP-score* plots are generated using standard Python visualization packages—specifically, matplotlib, seaborn, and adjustText. The variation in point colours is an automatic mapping provided by the seaborn.color_palette("hsv", ...) function, which assigns distinct colours to each unique fragment for visual separation only. These colours are not intended to encode specific residue-level information.

The meaningful classification is instead represented by the shaded background areas on the plot, which correspond to different *FP-score* categories:

- Red (Anchor): High frequency and high energy contributions (core binding hotspots),

- Yellow (Transient): High energy but moderate frequency (context-dependent interactions),
- Green (Accessible): High frequency but lower energy (peripheral or flexible residues),
- Cyan (Non-hotspots): Low frequency and energy (generally non-essential interactions).

Although residue labels may overlap in some plots due to the number of residues involved, the shaded areas remain the key interpretable regions for identifying functionally relevant sites.

---

12. Figure 7C, the figure of PDB-ID 2OHS is shown, but no discussion.

We thank the reviewer for pointing this out. Indeed, Figure 7C (PDB ID: 2OHS) was inadvertently included without a corresponding discussion in the main text. This has now been corrected in the revised manuscript by removing the figure to maintain consistency and clarity.

---

13. From the writing, sometimes the differences of LE =0.01 are significant, but sometimes they are not. What is your justification of significant changes of delta LEs?

We appreciate the reviewer's observation in regards of the LE. The apparent interpretation of ΔLE values arises from the sensitivity of the ligand efficiency (LE) metric to changes in molecular weight. Small variations in LE (e.g., 0.01) can appear significant or negligible depending on the molecular size and context. As fragment elaboration progresses, the added molecular weight disproportionately affects LE.

---

14. In Section 3.4, I noticed that both static and dynamic FMOPhores were performed for some proteins, but some were not. Are there any reasons and/or criteria for choosing when to perform only static FMOPhore or both static and dynamic analysis?

We appreciate the reviewer's observation regarding the use of static versus dynamic *FMOPhore* analyses. Our rationale for selecting systems for dynamic *FMOPhore* evaluation was based on the initial performance of static scans. Specifically, we selected seven protein systems where static *FMOPhore* predictions appeared suboptimal, either missing known binding hotspots or providing lower-than-expected *FP-scores* for key residues.

These cases were chosen to assess whether incorporating conformational sampling through MD (i.e., *Dy-FMOPhore*) would enhance the predictive power. Indeed, for most of these selected systems, the dynamic approach led to improved hotspot detection and *FP-score* enrichment, supporting the complementary value of conformational flexibility.

That said, we found that static *FMOPhore* scans alone are generally robust and computationally more efficient, making them a practical default for large-scale screening. *Dynamic-FMOPhore* is recommended in cases where high-resolution prediction is required or where structural flexibility is suspected to play a significant role in ligand recognition. We have added clarification on this decision criterion in Section 3.7 of the revised manuscript.

---

15. **In Figure 9, the orange and red colors are very difficult to distinguish. It would be good to separate the static and dynamic modes from each other.**

We thank the reviewer for this helpful suggestion regarding the clarity of Figure 9.

In response, we have updated the figure to visually separate the static and dynamic FMOPhore results more clearly. Specifically, we addressed the colour overlap issue by separating the static and dynamic mode scanning plots. This ensures that each mode is now distinctly represented and easily interpretable, even in printed or grayscale formats. The revised version of Figure 10 has been included in the updated manuscript.

---

16. **Line 510-511, "However, for other systems such as HSP90 and Hepatitis-C, were more challenging yet, both reported better classification for both**

Hotspots and Non-hotspots." —> I can't find the discussion about the challenges and the results of these protein systems.

Thank you for your comment. This has been corrected in the revised manuscript.

---

17. **It would be good to provide the details of the MD simulation.**

Thank you for the comment. All details of the MD simulations are provided in Supplementary Files 1.4 - 1.7.

---

18. **Fragment molecular orbital (FMO) method is quantum mechanics-based fragmentation. Are there any specific reasons in calling it as "QM-FMO"?**

We acknowledge that the Fragment Molecular Orbital (FMO) method is the standard term used in computational chemistry and quantum chemistry literature. However, we have occasionally referred to it as "QM-FMO", to ensure clarity for audiences who may be unfamiliar with FMO. The aim of our paper is to reach a broad audience across multiple disciplines, including medicinal and computational chemists with different scientific backgrounds. "QM-FMO" helps emphasize that it is a quantum mechanics-based fragmentation approach and distinguish it from other fragmentation-based methods.

However, we agree with the reviewer that using only the term 'FMO' simplifies the terminology, improves clarity, and aligns with its widespread usage in the literature, that is why we have corrected that to FMO only in the revised version.

---

19. **In the discussion, the authors provided some background of previous studies. However, some sentences are unclear if they were based on the literature or from the presented work.**

We appreciate the reviewer's comment. To improve clarity and avoid any ambiguity, we have revised the relevant discussion sections to explicitly distinguish between references to previous literature and findings from our current work. All external studies are now clearly cited, and statements based on our presented results are explicitly

indicated as such. We trust this addresses the concern regarding the distinction between cited literature and our contributions.

---

20. **Grammatical errors:**
    - **In the abstract, "FP-score accurately classify binding site residues" should be "FP-score accurately classifies binding site residues"**
    - **Page 3 line 26, "Thermal Shift Analysis are" should be "Thermal Shift Analysis is".**
    - **Page 4 line 39, "However, identification of hotspots and quantitatively determining" should be "However, identifying hotspots and quantitatively determining".**
    - **Line 41, "complementary ligand has been" should be "complementary ligand have been".**
    - **Line 53, "thirty-two" should be "32".**
    - **Line 74, "For protein-ligand pharmacophore models generation" should be "For protein-ligand pharmacophore model generation"**
    - **Line 105, 373, verb is missing.**
    - **Page 7, there are many subject/verb agreement errors.**
    - **Line 135-137, "Transient residues if targeted with a proper complimentary functional group can play a key role in increase 137 binding affinity while positively influencing the ligand efficiency." —> not sure what the authors are trying to explain.**

Although *Transient-category* residues may have low accessibility, their key chemical features are characterized by their ability to form direct electrostatic interactions, which can result in highly favourable interactions. However, achieving these interactions can be challenging, either due to the limited available volume surrounding the chemical feature or because of high flexibility caused by long, fluctuating side chains (such as Arg or Lys residues). Nevertheless, if an interaction is successfully established using a properly complementary functional group on the ligand, it can form a strong and stable interaction.

- **Line 189-190, "fifty different holo-complexes of noncovalent protein-ligands" should be "50 different holo-complexes of noncovalent protein-ligand complexes".**
- **Inconsistent use of the Oxford commas**
- **Line 460, "the carboxylic", should it be "the carbonyl"?**
- **Missing references for**
    - **GAMESS software**
    - **FMO**
    - **FMO2-DFTB**
    - **Section 3.1**

Thank you for all the valuable comments listed above. These have been addressed and can be tracked in the trackable version.

21. Code availability.

The code is openly available on GitHub and can be easily installed via pip install for user convenience.

Additionally, any installation support or assistance with the usage of the package can be provided by the developer, Peter E.G.F. Ibrahim. A link to the GitHub repository for installation instructions has been added to the revised manuscript. https://github.com/PeterEGFIbrahim/FMOPhore

Reviewer: 4

We thank the reviewer for taking the time to read and evaluate our manuscript and for acknowledging the technical soundness of the work. We respectfully disagree with the assessment that *FMOPhore* lacks originality or that it does not address significant unresolved challenges in the field.

While *FMOPhore* builds upon established concepts in protein–ligand interaction analysis, its novelty lies in its unique integration of Fragment Molecular Orbital (FMO) - derived energy decomposition with pharmacophore modelling through a novel scoring function, the *FP-score*. This scoring function quantitatively and qualitatively classifies binding site residues into four distinct categories - Hotspots, Anchors, Transient, and Non-hotspots - generating a structured, mechanistically informed hotspot map. To our knowledge, no other published method provides this level of systematic residue classification, coupled with quantum mechanically validated energetic contributions.

This classification offers a unique value to medicinal chemists, directly guiding molecular design by identifying which regions of the binding site should be stabilised, targeted for interactions, or deprioritised. Importantly, *Dy-FMOPhore* also incorporates protein binding site flexibility, enabling hotspot detection that reflects the dynamic nature of protein–ligand interactions, thereby improving biological realism compared to static models.

The utility of *FMOPhore* and the *FP-score* was further demonstrated in a prospective validation study involving the proteasome, where mutagenesis experiments confirmed our computational predictions. The tool successfully distinguished hotspots from non-hotspots in agreement with experimental saturation mutagenesis data, underscoring its predictive accuracy and practical relevance. This capability directly addresses a persistent challenge in fragment-based drug discovery — bridging in silico hotspot detection with practical applications such as drug resistance studies.

We respectfully submit that this integration of FMO-based energetic mapping, dynamic protein flexibility, residue classification via *FP-score*, and experimental validation represents a methodological advance not currently available in the literature, including in J. Chem. Inf. Model. or JCTC. We believe this combination of physical rigor, interpretability, experimental concordance, and direct medicinal chemistry applicability supports the manuscript's suitability for Nature Communications.

Once again, thank you for your time, support, and valuable insights and we hope the significant changes made now fall into line with your expected standards for publication.

Warm regards,

The authors.

Dear reviewers,

Firstly, we wanted to take a moment to express our thanks for taking the time to review the paper. Your feedback and constructive comments are useful, and we believe have enhanced the quality of our report. We genuinely appreciate the effort you put into providing feedback.

Accordingly, we would like to reply to the mentioned feedback as per reviewer:

Reviewer: 1

---

1. Prospective experimental validation

I would like to thank the authors for their thorough and constructive responses to my previous comments. Overall, the revisions address several of the points I raised, and the manuscript is now clearer in. In particular, the additional methodological details regarding the FMO calculations and pharmacophore integration, as well as the expanded validation dataset covering a more diverse set of targets, have improved the transparency of the study. The clarification on computational costs and the inclusion of supplementary information (workflow, input files, and probe library description) are also useful additions. The inclusion of an experimental validation of a point mutation case study provides some further support for the applicability of the proposed framework.

Nevertheless, I remain concerned that the current evaluation relies primarily on retrospective benchmarking. While these results are informative, I believe that for the approach to demonstrate genuine novelty and practical impact, prospective experimental validation will be essential. Without such evidence, the robustness and translational relevance of the framework remain uncertain.

We thank the reviewer for this insightful comment and agree that prospective validation is essential for demonstrating both novelty and translational relevance.

We would like to emphasize that the saturation mutagenesis study performed on the Proteasome β5 subunit mentioned in the manuscript with our experimental collaborators constitutes a genuine prospective validation: the experiments were explicitly designed

and executed to test our framework by prospectively identifying resistance-linked hotspots.

We believe this work directly addresses the reviewer's request for "prospective validation case study." Coordinating this prospective study with our collaborators was also a key reason for the longer response time to the first-stage reviews, as we were awaiting the appearance of their manuscript on bioRxiv (https://www.biorxiv.org/content/10.1101/2025.07.25.666894v1) ; our revised submission was filed the following days to ensure the reviewer had access to this prospective evidence.

We are also actively trying to pursue a second prospective example with our group leads. However, as ongoing projects often involve intellectual property considerations, there may be limits on what can be shared within the current timeline. Nonetheless, we regard prospective validation as central to our approach and remain committed to expanding this aspect in future work.

Reviewers: 2

Thanks for your responses. Many of my questions have been addressed. It's clear to me now how the FP-score quadrant is generated. I believe the explanation on using the mean values of interaction energy and PI% as a threshold should be included in the main manuscript, since this is the key to classifying the hotspot.

1. **In the manuscript, it is mentioned that PCM was included in FMO/DFTB calculations. However, the supporting info shows that no solvation model is used. This conflict needs to be resolved**

We thank the reviewer for pointing out this point. This has been corrected in the Supplementary Information to clearly state that the FMO/DFTB3 calculations were performed with the 3ob-3-1 parameter set in GAMESS, which incorporates corrections for dispersion and solvation effects using the Polarizable continuum model (PCM) water model.

---

2. **The work on FMO/DFTB3 is not cited. This reference should be included. https://doi.org/10.1016/j.cplett.2015.07.022**

We thank the reviewer for this valuable comment. The suggested reference has now been added, and the section has been corrected in the revised version for improved clarity.

Reviewers: 3

---

3. **The code is in GitHub and freely available under an open-source license. We were not able to test the software because it requires complex other software packages preinstalled such as GAMESS.**

We thank the reviewer for this comment and fully understand the difficulty associated with installing and configuring GAMESS. To support the community, we provide direct contact and assistance with the developer to all users to facilitate installation and running of the code, as outlined on our GitHub page. A link to the repository with installation instructions is included in the revised manuscript.

https://github.com/PeterEGFIbrahim/FMOPhore - The code is openly available on GitHub and can be easily installed via pip install for user convenience.

Reviewer: 4

---

1. **Why two conda deactivate commands in the shell-script? (lines 16 and 17).**

We thank the reviewer for this observation. The first conda deactivate command is used to deactivate the active "***FMOPhore_env***" environment after running the code, while the second conda deactivate command fully deactivates conda itself, returning the shell to its base state.

Once again, thank you for your time, support, and valuable insights and we hope the significant changes made now fall into line with your expected standards for publication.

Warm regards,

The authors.

Dear reviewers,

Firstly, we wanted to take a moment to express our thanks for taking the time to review the paper. Your feedback and constructive comments are useful, and we believe have enhanced the quality of our report. We genuinely appreciate the effort you put into providing feedback.

Accordingly, we would like to reply to the mentioned feedback as per reviewer:

Reviewer: 1

---

1. Prospective experimental validation

I would like to thank the authors for their thorough and constructive responses to my previous comments. Overall, the revisions address several of the points I raised, and the manuscript is now clearer in. In particular, the additional methodological details regarding the FMO calculations and pharmacophore integration, as well as the expanded validation dataset covering a more diverse set of targets, have improved the transparency of the study. The clarification on computational costs and the inclusion of supplementary information (workflow, input files, and probe library description) are also useful additions. The inclusion of an experimental validation of a point mutation case study provides some further support for the applicability of the proposed framework.

Nevertheless, I remain concerned that the current evaluation relies primarily on retrospective benchmarking. While these results are informative, I believe that for

the approach to demonstrate genuine novelty and practical impact, prospective experimental validation will be essential. Without such evidence, the robustness and translational relevance of the framework remain uncertain.

We agree that future experimental tests of the FMOPhore approach will be informative in this regard but also believe that further experimental tests are beyond the scope of our current study. As stated above, comparison of FMOPhore predictions with the empirical data generated by Altmann et al, (https://www.biorxiv.org/content/10.1101/2025.07.25.666894v1) indeed "provides some further support for the applicability of the proposed framework".

We've now adjusted the text here to further emphasise the power of comparing our FMOPhore predictions with saturation mutagenesis data, since such datasets are not typically available for priority drug targets. The adjusted text now reads as follows: "A recent mutagenesis scanning study (Altmann et al.) 59, presented an excellent opportunity to compare our results with empirical data for all possible mutations around the compound 7a drug-binding pocket. Remarkably, the saturation mutagenesis study also identified Gly-98, Tyr-113, Val-128, Ser-132, and Tyr-136 as compound 7a resistance hotspots".

Reviewers: 2

Thanks for your responses. Many of my questions have been addressed. It's clear to me now how the FP-score quadrant is generated. I believe the explanation on using the mean values of interaction energy and PI% as a threshold should be included in the main manuscript, since this is the key to classifying the hotspot.

1. **In the manuscript, it is mentioned that PCM was included in FMO/DFTB calculations. However, the supporting info shows that no solvation model is used. This conflict needs to be resolved**

We thank the reviewer for pointing out this point. This has been corrected in the Supplementary Information to clearly state that the FMO/DFTB3 calculations were performed with the 3ob-3-1 parameter set in GAMESS, which incorporates corrections for dispersion and solvation effects using the Polarizable continuum model (PCM) water model.

---

2. **The work on FMO/DFTB3 is not cited. This reference should be included. https://doi.org/10.1016/j.cplett.2015.07.022**

We thank the reviewer for this valuable comment. The suggested reference has now been added, and the section has been corrected in the revised version for improved clarity.

Reviewers: 3

---

3. **The code is in GitHub and freely available under an open-source license. We were not able to test the software because it requires complex other software packages preinstalled such as GAMESS.**

We thank the reviewer for this comment and fully understand the difficulty associated with installing and configuring GAMESS.

https://github.com/DDU-CDD/fmophore - The code is available on GitHub for reviewing. With the following key: This key for GIT-HUB access is for reviewing purposes only and cannot be shared further.

github_pat_11BZ6A65I0ZpEQ0k5hLqZg_IBl9KA6bv00H8XwBabUM3nSDCDPD
qzgEvjMFNMrbSjr3BXPSZDAhu4UIDrx

Reviewer: 4

---

1. **Why two conda deactivate commands in the shell-script? (lines 16 and 17).**

We thank the reviewer for this observation. The first conda deactivate command is used to deactivate the active "***FMOPhore_env***" environment after running the code, while the second conda deactivate command fully deactivates conda itself, returning the shell to its base state.

Once again, thank you for your time, support, and valuable insights and we hope the significant changes made now fall into line with your expected standards for publication.

Warm regards, The authors.
